# RNA-Seq of the Caribbean reef-building coral *Orbicella faveolata* (Scleractinia-Merulinidae) under bleaching and disease stress expands models of coral innate immunity

**DOI:** 10.7717/peerj.1616

**Published:** 2016-02-15

**Authors:** David A. Anderson, Marcus E. Walz, Ernesto Weil, Peter Tonellato, Matthew C. Smith

**Affiliations:** 1 Department of Pathology and Immunology, Washington University in St. Louis, St. Louis, Missouri, United States of America; 2 Department of Marine Sciences, University of Puerto Rico at Mayagüez, Mayagüez, Puerto Rico, United States of America; 3 Joseph J. Zilber School of Public Health, University of Wisconsin-Milwaukee, Milwaukee, Wisconsin, United States of America; 4 Department of Biomedical Informatics, Harvard Medical School, Harvard University, Boston, Massachusetts, United States of America; 5 School of Freshwater Sciences, University of Wisconsin-Milwaukee, Milwaukee, Wisconsin, United States of America

**Keywords:** RNA-seq, Disease, Cnidaria, Coral, Innate immunity

## Abstract

Climate change-driven coral disease outbreaks have led to widespread declines in coral populations. Early work on coral genomics established that corals have a complex innate immune system, and whole-transcriptome gene expression studies have revealed mechanisms by which the coral immune system responds to stress and disease. The present investigation expands bioinformatic data available to study coral molecular physiology through the assembly and annotation of a reference transcriptome of the Caribbean reef-building coral, *Orbicella faveolata*. Samples were collected during a warm water thermal anomaly, coral bleaching event and Caribbean yellow band disease outbreak in 2010 in Puerto Rico. Multiplex sequencing of RNA on the Illumina GAIIx platform and de novo transcriptome assembly by Trinity produced 70,745,177 raw short-sequence reads and 32,463 *O. faveolata* transcripts, respectively. The reference transcriptome was annotated with gene ontologies, mapped to KEGG pathways, and a predicted proteome of 20,488 sequences was generated. Protein families and signaling pathways that are essential in the regulation of innate immunity across Phyla were investigated in-depth. Results were used to develop models of evolutionarily conserved Wnt, Notch, Rig-like receptor, Nod-like receptor, and Dicer signaling. *O. faveolata* is a coral species that has been studied widely under climate-driven stress and disease, and the present investigation provides new data on the genes that putatively regulate its immune system.

## Introduction

Coral reefs are experiencing a dramatic decline in coral cover and reef biodiversity, reports of which have been documented as early as the 1970s (Alemu & Clement, 2014; Altizer et al., 2013; Antonius, 1973; Bastidas et al., 2012; Bruno et al., 2007; Ducklow & Mitchell, 1979; Glynn, Peters & Muscatine, 1985; Glynn, 1983; Harvell et al., 1999; Tracy et al., 2015; Weil & Rogers, 2011; Weil, Smith & Gil-Agudelo, 2006). Climate change-driven stress can lead to disease outbreaks by shifting coral-associated microbial communities from symbiont and commensal-dominated to pathogen-dominated (Bourne et al., 2009; Rosenberg et al., 2007), and the molecular and cellular responses of coral to disease and environmental stress are well established (Ocampo et al., 2015; Palmer & Traylor-Knowles, 2012; Pinzón et al., 2015; Weiss et al., 2013). The basic model of coral immune responses to disease involves the migration of pluripotent immunocytes, also known as amoebocytes, to physical wounds and disease lesions; the production of cytotoxic reactive oxygen species; the production of antioxidants to reduce self-harm; the accumulation of melanin as a barrier to pathogen invasion; and the production of antimicrobial compounds to regulate commensal microbiota (Mydlarz et al., 2008; Palmer, Modi & Mydlarz, 2009; Tchernov et al., 2011; Vidal-Dupiol et al., 2011; Weis, 2008). Failure to overcome an infection leads to the manifestation of lesions and tissue mortality that are associated with molecular and cellular signatures of apoptosis (Ainsworth et al., 2007; Anderson & Gilchrist, 2008).

Next Generation Sequencing (NGS) technologies promise to reveal the genetic mechanisms that control the coral immune system on a whole-genome and whole-transcriptome scale. The versatility of NGS allows for the analysis of samples collected in situ and can thus be used to study physiological responses to natural disease and climate stress events. Several investigations have used NGS to identify putative immunity genes that are differentially expressed under stress and disease (Barshis et al., 2013; Burge et al., 2013; Libro, Kaluziak & Vollmer, 2013; Ocampo et al., 2015; Palumbi et al., 2014; Pinzón et al., 2015; Traylor-Knowles & Palumbi, 2014; Weiss et al., 2013). *Orbicella faveolata* is an important Caribbean and Atlantic reef-building coral. It has experienced recent population declines and is classified as a threatened species under the federal endangered species act (NOAA, 2014). In particular, this species has been severely impacted by coral bleaching and Caribbean Yellow Band Disease (CYBD) across its geographic range (Borger & Colley, 2010; Bruckner, 2012; Bruckner & Hill, 2009; Weil, Cróquer & Urreiztieta, 2009a; Weil et al., 2009b; Weil & Rogers, 2011). To better understand the biological mechanisms of this decline, transcriptomics has been used to define changes in gene expression of this coral and commensal microbiota in response to environmental stress during larval development, the establishment of symbiosis, and the maintenance of homeostasis (Aranda et al., 2012; Borger & Colley, 2010; Closek et al., 2014; Cróquer et al., 2013; Desalvo et al., 2008; Kimes et al., 2010; Pinzón et al., 2015; Roder et al., 2014; Schwarz et al., 2008; Sunagawa et al., 2009; Voolstra et al., 2009).

Most recently, Pinzón et al. (2015) used NGS to track temporal changes in gene expression of *O. faveolata* through a warm water thermal anomaly and bleaching event in 2010 in Puerto Rico. The present RNA-Seq-based investigation sampled Caribbean Yellow Band-Diseased (CYBD), bleached and asymptomatic colonies of *O. faveolata* during the same event. A reference transcriptome was assembled, annotated, and translated into a predicted proteome. Protein families and signaling pathways that were represented in the transcriptome but that have not been studied previously in the context of coral immune responses to stress and disease were selected for in-depth analysis. Phylogenetic analysis uncovered novel homologues of the Wnt protein family in the *O. faveolata* transcriptome, the signaling pathway of which is involved in immune cell differentiation and migration. Domain architectures for novel *O. faveolata* Dicer-like proteins, function in small RNA expression and antiviral immunity, are compared to putative homologues conserved across phyla. Finally, coral-specific Nod-like receptor, Rig-like receptor and Notch signaling pathways are illustrated to support future research on the study intracellular pathogen sensing and wound healing in corals. The results of this work expand current bioinformatic resources available for *O. faveolata* and present an in-depth analysis of evolutionarily conserved gene sets involved in the regulation of coral innate immunity.

## Methods

### Sample collection

**Figure 1:**
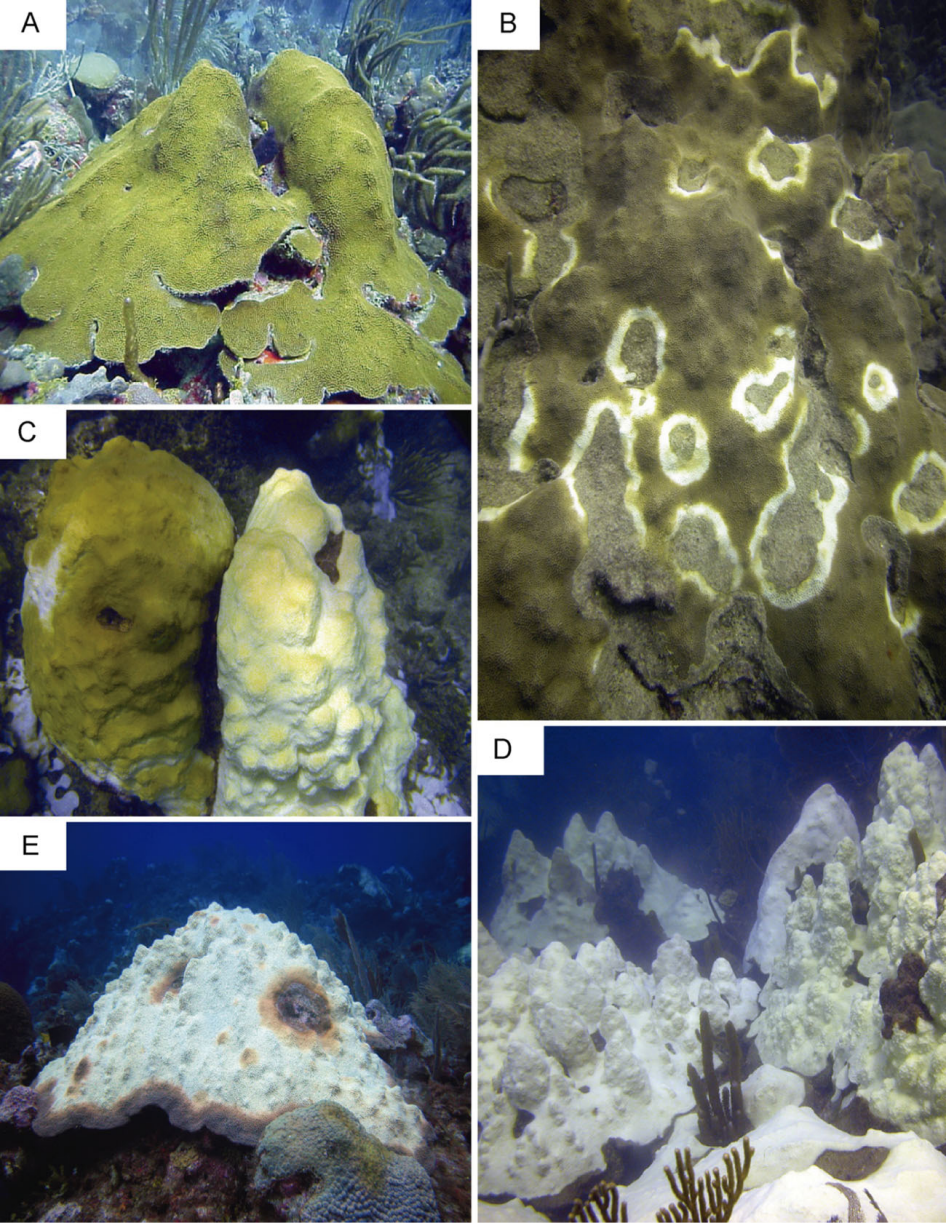
Representative images of colonies sampled in the present study. (A) Asymptomatic. (B) Caribbean yellow band-diseased. (C) Partially bleached colonies. (D) Completely bleached colonies. (E) Caribbean yellow band-diseased and bleached. Photos by E. Weil.

A concurrent thermal anomaly, coral bleaching event and Caribbean yellow band disease outbreak occurred in 2010 in Puerto Rico. This event provided a unique opportunity to sample colonies of *O. faveolata* affected by multiple environmental stressors that are known to induce innate immune responses (Mydlarz et al., 2009; Pinzón et al., 2015). Six samples (approximately 25 cm^2^) from four colonies were collected on a single dive at 10 m depth in October 2010 on Media Luna reef in La Parguera, Puerto Rico (17°56.091 N, 67°02.577 W). Samples were collected under a permit issued by the Department of Natural Resources of Puerto Rico to the Department of Marine Sciences at the University of Puerto Rico at Mayaguez. Reefs in this region experienced ten degree-heating weeks at the time of sample collection. Degree-heating week is a remote sensing metric that estimates accumulated thermal stress in corals during sea surface temperature anomalies (Gleeson & Strong, 1995), and is reported by the National Oceanic and Atmospheric Administration. Five different health conditions were sampled: bleached (sample 1) and asymptomatic tissue (sample 2) of a partially bleached colony; asymptomatic tissue (sample 3) and lesion tissue (samples 4 and 5) from a CYBD-affected colonies; and tissue from a completely asymptomatic colony (sample 6) (Supplemental Information 1). The conditions represented by samples 3 and 4 have not been used for NGS by any previously reported investigation. Photographic examples of each disease condition are presented in Fig. 1. Within one hour of collection and storage at ambient temperature in seawater, tissue samples were transported to the Department of Marine Sciences on Isla Magueyes, flash frozen in liquid nitrogen, photographed while on dry ice, and stored at −80 °C. It was assumed that colonies sampled were non-clonal given their large distances of separation (>10 m) and low clonal levels (3.5%) previously documented for the same species on the same reef (Severance & Karl, 2006).

### RNA extraction, sequencing, and de novo transcriptome assembly

The area of each tissue sample was estimated from photographs scaled with millimeter precision using ImageJ software (Schneider, Rasband & Eliceiri, 2012). The ratio of the sample area to volume of Trizol (Life Technologies, CA, USA) was the same for each sample: 2.0 mL of Trizol was added to every 1.00 cm^2^ of sample tissue in 50 ml capped tubes. Tissue was homogenized by vigorous shaking until skeletons were completely denuded. A neutralization reaction occurs between the calcium carbonate skeleton of the coral and the acidic Trizol, so 1 to 5 µl of 6 M hydrochloric acid were added to each sample to minimize DNA contamination of the aqueous phase on the addition of chloroform. For each sample, 700 µl of the aqueous phase was loaded onto and spun through a single RNeasy column, and an on-column DNA digestion step was performed using DNase according to the manufacturer’s protocol (Qiagen, Velno, Netherlands). RNA was eluted from the column with nuclease-free water, and RNA quality and concentration were verified by 2% denaturing agarose gel electrophoresis and a NanoDrop spectrophotometer (Thermo Scientific, Waltham, MA, USA), respectively.

Total RNA was sent for mRNA multiplex sequencing on the Illumina GAIIx platform (Illumina Inc, San Diego, CA, USA) according to standard protocols of the genomic core facility at the Scripps Research Institute in Ft. Pierce, Florida, USA. All samples were sequenced in multiplex on two lanes of an Illumina flow cell. Raw sequence reads from each sample are available on the NCBI Short Read Archive under Bioproject PRJNA236103 and accession numbers for each sample are reported in Supplemental Information 1. From 70,745,177 raw sequence reads of 72 bases in length, adaptor sequences and low-quality bases were trimmed and clipped using cutadapt (Martin, 2011), which resulted in 59,114,519 reads with a mean length of 67 bases (standard deviation of 5 bases). Raw reads were trimmed and clipped to optimize sequence quality based on results of RNA-SeQC analysis (DeLuca et al., 2012). The quality of the data used for sequence assembly before and after processing is reported as a supplementary figure (Supplemental Information 2). The Trinity software suite was chosen for de novo assembly of the *O. faveolata* transcriptome using 59,114,519 processed sequence reads and default parameters (Grabherr et al., 2011; Haas et al., 2013).

### Generation of the *Orbicella faveolata* reference transcriptome

To identify coral host and microbial sequences in the metatranscriptome, a series BLASTn alignments were conducted in parallel. For the first series, transcripts assembled by Trinity were aligned to transcripts previously reported for the photosynthetic endosymbiont, *Symbiodinium spp*. (Bayer et al., 2012; Shoguchi et al., 2013). Hits with an e-value less than 1E-3 were removed. For the second series, transcripts assembled by Trinity were aligned to *O. faveolata* expression sequence tags (EST) (E-value cutoff of 1E-6), *Acropora digitifera* mRNA sequences (E-value cutoff of 1E-3) (Shinzato et al., 2011), *Nematostella vectensis* mRNA sequences (E-value cutoff of 1E-3) (Putnam et al., 2007), and *Hydra magnipapillata* mRNA sequences (E-value cutoff of 1E-3) (Chapman et al., 2010). Transcripts with hits lower than the cutoff were assigned as *O. faveolata* in origin. Sequences downloaded for this analysis from their respective sources are provided as a supplementary file (Supplemental Information 3). Sequences that had significant hits to both *Symbiodinium* and Cnidarian sequences in both parallel analyses underwent a second-round of filtering using the classifier for metagenomic sequences (CLaMS). The complete genome of *N. vectensis* and whole transcriptome of *Symbiodinium* were used as training sets (Pati et al., 2011). Sequences that were binned as *Symbiodinium* only or both *Symbiodinium* and Cnidarian in origin were removed, and sequences that were binned as Cnidarian only were retained in the *O. faveolata* reference transcriptome. The resulting *O. faveolata*-specific reference transcriptome is provided as a compressed supplementary file (Supplemental Information 4a). A predicted proteome was generated using TransDecoder, a package within the Trinity software suite (Haas et al., 2013). The predicted proteome contains 20,488 sequences and is provided as a supplementary file (Supplemental Information 4b).

### Transcriptome annotation and pathway analysis

To assess sequence accuracy of the de novo reference transcriptome, assembled transcripts were aligned to *O. faveolata* Expressed Sequence Tags (ESTs) in NCBI. *O. faveolata* ESTs have been submitted to NCBI by various sources. The sequences downloaded and used here are provided as a supplementary file (Supplemental Information 5). Transcriptome annotation was conducted using a combination of methods. Gene ontologies and EMBL/InterProScan protein motifs were assigned using BLASTx alignments on the BLAST2GO platform, which extracts annotations from best hits to non-redundant protein sequences with a maximum e-value of 1E-3 (Boratyn et al., 2013; Conesa et al., 2005; Quevillon et al., 2005). Taxonomic identities of best-hit sequences were extracted for a *post-hoc* assessment of sequence contamination in the *O. faveolata*-specific reference transcriptome. Transcripts were also annotated with KEGG Orthologues (KO) using default settings for the KEGG Automatic Annotation Server (KAAS) with a minimum BLAST score of 60 (Moriya et al., 2007). To determine the completeness of the *O. faveolata* transcriptome, KEGG KAAS annotations were conducted in parallel with mRNA sequences from six other Cnidarians: *N. vectensis*, *H. vulgaris*, *Porites astreoides*, *A. millepora*, *A. digitifera*, and *P. damicornis* (Chapman et al., 2010; Hemmrich et al., 2012; Kenkel, Meyer & Matz, 2013; Moya et al., 2012; Pinzón et al., 2015; Putnam et al., 2007; Shinzato et al., 2011; Traylor-Knowles et al., 2011). The reference transcriptome reported by Pinzón et al. (2015) was also annotated with KEGG KAAS in parallel for direct comparisons to the present data set.

### Identification and analysis of putative immunity genes, proteins, and pathways

Correct open reading frames and domain architectures for predicted protein sequences were verified by hmmscan in the HMMER web server (Finn, Clements & Eddy, 2011). For phylogenetic analysis of predicted Wnt protein sequences, assignment to a specific Wnt family member was based on phylogenetic tree construction and clustering with previously described Wnt proteins for *N. vectensis* (Kusserow et al., 2005). Whole-length sequences were aligned by MUSCLE (Edgar, 2004), conserved regions were curated using Gblocks (Talavera & Castresana, 2007), maximum likelihood phylogenies were estimated using PhyML with 100 bootstraps and the Dayhoff substitution model (Dayhoff, Schwartz & Orcutt, 1978), and trees were constructed using TreeDyn (Chevenet et al., 2006). This pipeline was executed using the Phylogeny.fr platform (Dereeper et al., 2008). Immune signaling pathways were constructed by mapping assigned KO terms to KEGG pathway maps. Pathways were modified to illustrate the presence or absence of essential signaling components in the *O. faveolata* transcriptomes reported here and by Pinzón et al. (2015). Components of the miRNA and siRNA pathway gene list were selected based components reported to be conserved across invertebrates, including Cnidarians (Ding & Voinnet, 2007; Moran et al., 2013).

## Results and Discussion

The present investigation expands the bioinformatic resources available for the study of *Orbicella faveolata* and its physiological responses to stress and disease. The reference transcriptome generated here was annotated with gene ontologies, KEGG orthologies, and KEGG pathways. Immune cell development, migration, and intracellular microbial sensing pathways were emphasized to highlight aspects of the coral immune system that remain poorly characterized to date. Phylogenetic and domain architecture analyses revealed several new members of the Wnt and Dicer-like protein families, and pathway analysis revealed significant coverage of gene sets for Notch, Nod-like receptor, and Rig-like receptor pathways. Together, the results of this work provide new bioinformatic data for *O. faveolata* and an in-depth analysis of evolutionarily conserved aspects of the coral innate immune system.

### Transcriptome assembly and quality

**Figure 2:**
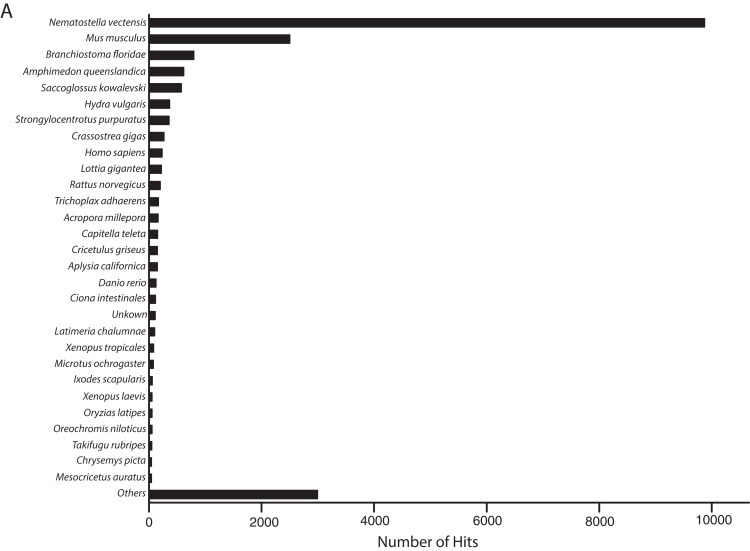
Frequency distribution of taxonomic identities of best hits to *O. faveolata* transcripts. BLAST2GO analysis results showing taxonomic identities of best BLASTx hits to *O. faveolata* transcripts from SwissProt non-redundant protein sequence database.

The metatranscriptome sequences assembled by Trinity included 35,967 and 47,760 transcripts that were identified by BLASTn alignments as *O. faveolata* and *Symbiodinium* in origin. However, 3504 transcripts were identified as both coral and *Symbiodinium* by CLaMS analyses. Those sequences were removed thus producing an *O. faveolata*-specific transcriptome that contains 32,463 sequences. BLASTx alignment of these sequences to non-redundant protein sequences in SwissProt by BLAST2GO revealed most frequent hits to *N. vectensis* followed by other metazoans (Fig. 2). This provided a *post hoc* confirmation that contaminating sequences were successfully removed from the *O. faveolata* reference transcriptome. The size of the reference transcriptome is similar to previous studies that have used the Illumina GAII platform, which report between 33,000 and 48,000 unique coral transcripts (Barshis et al., 2013; Libro, Kaluziak & Vollmer, 2013). The GC content of the reference transcriptome is 44%, which is comparable to previous reports for corals (Sabourault et al., 2009; Soza-Ried et al., 2010; Vidal-Dupiol et al., 2013). Sequence accuracy was high with reference transcriptome sequences sharing 96% identity with corresponding *O. faveolata* ESTs in NCBI (Supplemental Information 6). The N50 was 1736 bp, which is comparable to recent studies that produced de novo coral transcriptomes (Barshis et al., 2013; Burge et al., 2013; Lehnert, Burriesci & Pringle, 2012; Libro, Kaluziak & Vollmer, 2013; Moya et al., 2012; Pinzón et al., 2015; Pooyaei Mehr et al., 2013; Shinzato, Inoue & Kusakabe, 2014; Sun et al., 2012). This value is low compared to the most recent sequencing efforts for corals, but this is likely an indication of the low number of total sequence reads rather than sequence quality. High quality of short reads is demonstrated by RNA-SeQC results, which compare the raw sequence reads to trimmed and clipped sequences used as the input for assembly by Trinity (Supplemental Informations 1 and 2). Collectively, these results demonstrate that the methods used were sufficient to assemble an accurate de novo reference transcriptome for *O. faveolata*.

### Transcriptome annotation and novel sequences

**Figure 3:**
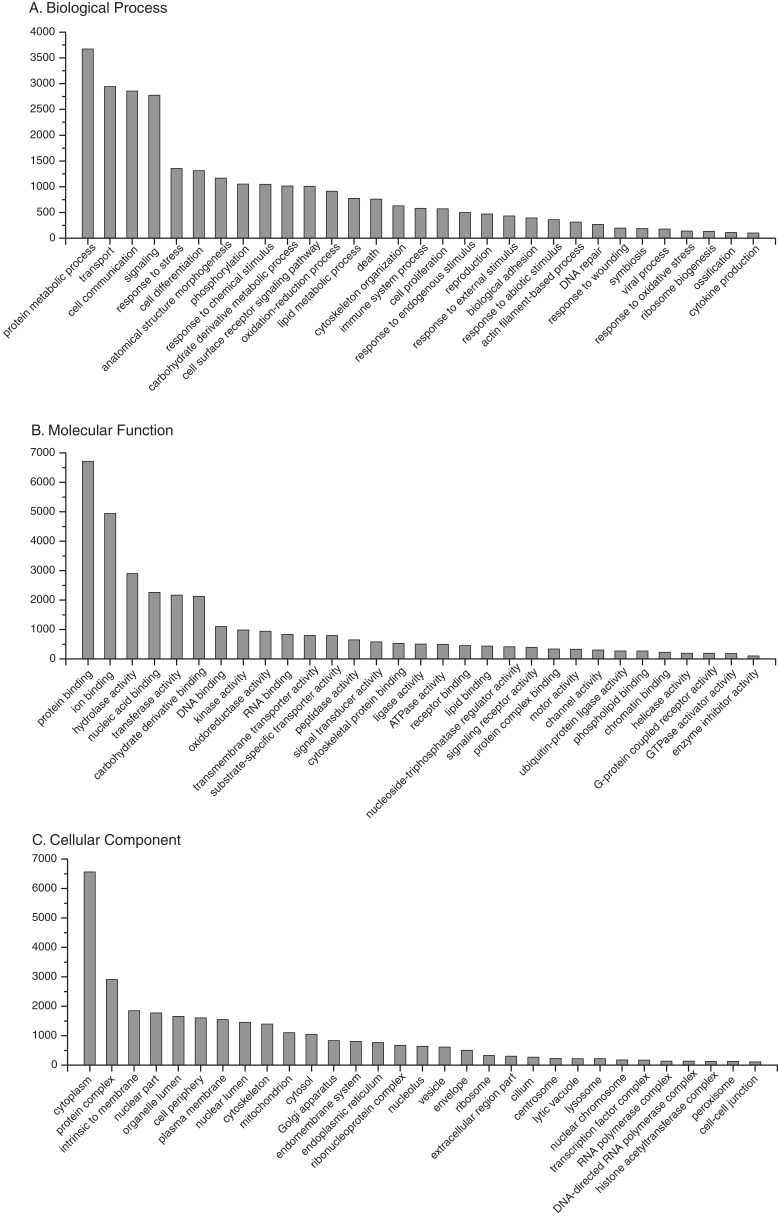
Frequency distribution of selected Gene Ontology (GO) terms annotated to *O. faveolata* transcripts. Results of BLAST2GO annotation of *O. faveolata* transcripts with GO terms. (A) Biological process GO terms. (B) Molecular function GO terms. (C) Cellular component GO terms.

BLAST2GO annotation assigned Gene Ontology (GO) terms and protein domain identities to 20,913 (64%) sequences with a maximum E-value of 1E-3 (Supplemental Information 7). A summary of the most abundant gene ontology terms for biological process, molecular function and cellular component is presented in Fig. 3. As expected, there was an abundance of transcripts associated with essential processes, such as metabolism, transcription, translation and protein complexes. In addition, there are transcripts associated with processes related to the physiological state of stress and disease, such as response to oxidative stress, death, immune system process, symbiosis, and response to wounding. Complete KO term and KEGG pathway annotations are provided as supplementary files (Supplemental Informations 8 and 9, respectively). To assess transcriptome completeness, a number of other Cnidarian transcriptomes were annotated in parallel for direct comparisons to the present data set (Table 1). Representation of metabolic and protein complex pathways for the present reference transcriptome was similar or slightly lower than data sets for other Cnidarian species. The results indicate that limited sequencing depth may underrepresent the full repertoire of possible *O. faveolata* transcripts. However, the present data set has a number of sequences associated with immune system-related pathways that are on par with or exceed the other data sets for Cnidarians (Table 1).

**Table 1:** KAAS pathway analysis of the *O. faveolata* reference transcriptome. The following transcript sequence resources were annotated in parallel using the KEEG KAAS sever and compared to the reference transcriptome reported in the present investigation for *O. faveolata*: NCBI Refseq mRNA sequences for *N. vectensis* and *H. magnipapillata* (Putnam et al., 2007; Chapman et al., 2010); reference transcriptome sequences for *A. millepora*, *P. astreoides*, *A. digitifera,* and *P. damincornis* (Moya et al., 2012; Kenkel, Meyer & Matz, 2013; Shinzato et al., 2011; Traylor-Knowles et al., 2011); and reference transcriptome sequences reported previously for *O. faveolata* (Pinzón et al., 2015). The number of transcripts annotated to each pathway is reported, and the number of transcripts that are unique to the independent *O. faveolata* transcriptomes is reported in the column labeled as unique.

Metabolism	*N. vectensis*	*H. magnipapillata*	*P. astreoides*	*A. millepora*	*A. digitifera*	*P. damicornis*	*O. faveolata*			
							Present	Unique	Pinzón et al. (2015)	Unique
Glycolysis & Gluconeogenesis	28	28	29	31	31	32	28	1	**31**	3
Pentose Phosphate	18	16	17	19	18	17	18	0	**19**	1
Citrate Cycle	23	22	21	23	21	23	20	0	**23**	3
Biosynthesis of Amino Acids	55	42	44	51	49	52	49	1	**53**	5
Valine, Leucine and Isoleucine Degradation	35	34	31	36	38	35	35	1	**36**	2
Purine Metabolism	101	85	89	106	102	85	95	6	**111**	21
Fatty Acid Metabolism	27	22	23	26	27	28	24	1	**26**	3
Pyrimidine Metabolism	69	65	63	73	68	50	63	2	**72**	11
**Protein Complexes**										
Spliceosome	100	98	93	103	99	94	96	9	**101**	13
Ribosome	112	102	97	87	113	82	100	2	**115**	17
Protein Export	20	20	17	20	19	14	20	0	**21**	1
Oxidative Phosphorylation	81	62	76	78	81	56	85	11	76	2
RNA Degradation	53	52	41	56	52	42	45	1	**54**	10
Ubiquitin Proteolysis	84	78	76	95	89	78	84	9	**94**	18
**Stress and Immunity**										
MAPK	70	77	73	90	88	76	**110**	28	95	12
Ras	56	57	64	76	74	67	**88**	20	78	9
Wnt	51	45	50	58	55	52	53	5	**60**	11
Notch	15	14	19	18	20	17	**22**	5	18	1
Phagosome	45	43	46	47	49	45	**53**	9	49	5
Peroxisome	53	39	41	54	53	47	46	2	**55**	11
Toll-like Receptor	22	22	20	27	23	23	27	3	**28**	4
Rig-like Receptor	19	18	18	19	19	19	**23**	2	22	3
Bacterial Invasion	32	30	35	36	37	34	**43**	8	36	1
Autophagy	15	15	12	15	15	13	13	1	**14**	2
Apoptosis	21	24	21	27	23	24	27	3	**30**	5
p53	26	28	21	32	28	26	28	6	**29**	7
Nod-like Receptor	13	13	18	19	17	18	19	3	19	3
NF-kB	17	18	17	29	20	25	**26**	8	24	6
PI3K-Akt	72	78	86	97	92	88	99	16	**101**	17
Complement	2	4	13	9	7	10	**13**	7	9	3
Cytosolic DNA sensing	19	16	20	24	19	16	21	3	**23**	5
Leukocyte Migration	31	28	32	32	30	29	**38**	10	30	2

**Figure 4:**
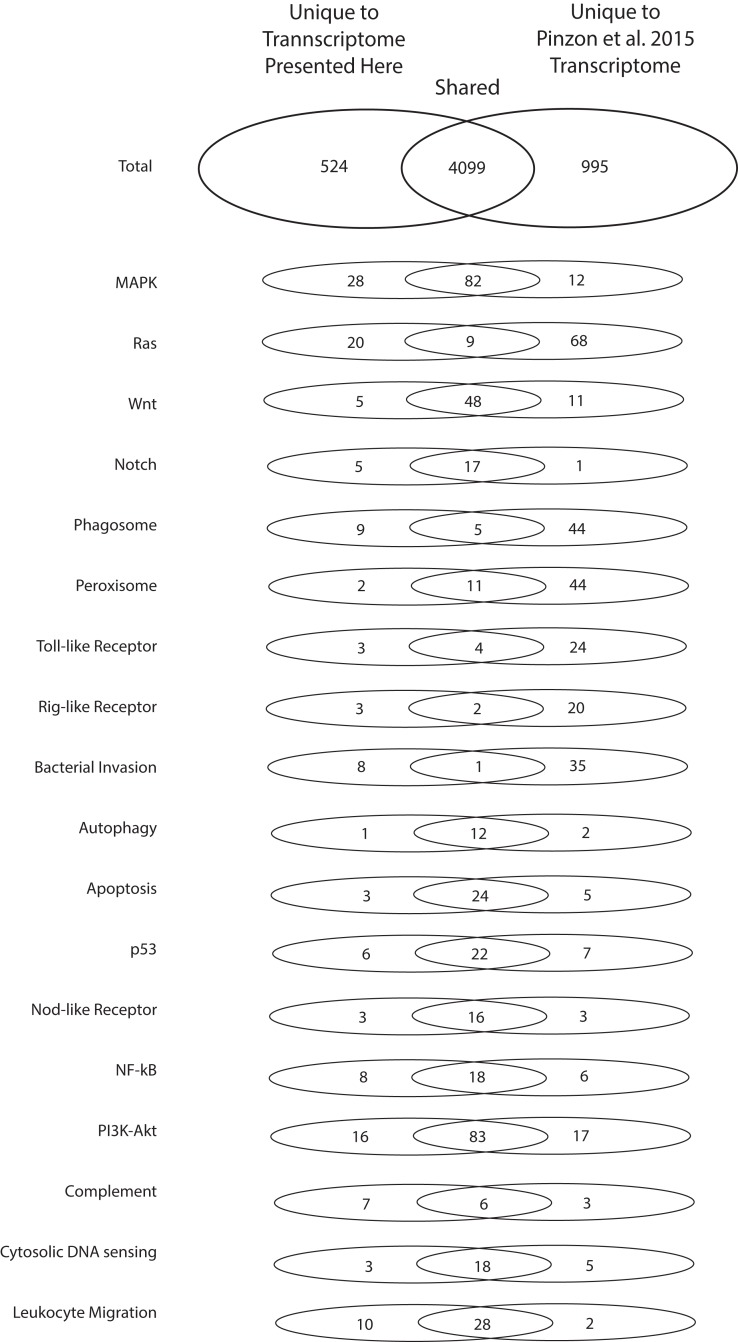
Numbers of unique and shared sequences between the present and (Pinzón et al., 2015) *O. faveolata* transcriptomes for select immunity-related KEGG pathways. Results from KEGG KAAS parallel annotation of the present transcriptome and the (Pinzón et al., 2015) transcriptome for *O. faveolata.* The number of non-redundant annotations mapped to select KEGG pathways for each transcriptome are shown.

To highlight the unique contributions of the present investigation to the bioinformatic data available for *O. faveolata*, an in-depth comparison to the (Pinzón et al., 2015) *O. faveolata* transcriptome is presented. The 70,745,177 raw sequence reads from which the present reference transcriptome is derived is small in comparison to the 387,512,512 raw sequence reads reported by Pinzón et al. (2015). When annotated in parallel, we estimate that the two projects share 5,618 unique KO terms (79% of total). The present study contributes 524 unique KO terms (7% of total) and the latter contributes 995 unique KO terms (14% of total). Greater sequencing depth for the (Pinzón et al., 2015) reveals better coverage of KEGG pathways used conventionally to assess transcriptome completeness (e.g. metabolic and protein complex pathways) (Table 1). However, the present reference transcriptome had a greater number of transcripts mapped to the following stress and immunity-related pathways: MAPK, Ras, Notch, phagosome, Rig-i-like, bacterial invasion, nuclear factor kappa beta, complement, and leukocyte migration Pathways (Fig. 4). Therefore, the transcriptome presented here contributes a considerable number of sequences not reported previously that are associated with evolutionarily conserved pathways of the innate immune system.

### The coral innate immune system

Models of coral immune response to disease are generally characterized by inflammation that involves the production of antimicrobial peptides and reactive oxygen species, the production of antioxidants to reduce self-harm, the migration of phagocytic cells to sites of infection, and the accumulation of melanin to prevent the spread of infection (Mydlarz et al., 2008; Palmer, Modi & Mydlarz, 2009; Tchernov et al., 2011; Vidal-Dupiol et al., 2011; Weis, 2008). In the arms race between invading pathogens and the coral host, a breakdown of host homeostasis leads to the activation of apoptosis and ultimately tissue mortality (Weis, 2008). Signaling pathways that control the coral immune system can be organized into 4 levels: (1) pathogen sensing by pattern recognition receptors, (2) downstream signaling cascades, (3) activation of inflammatory cytokine expression, and (4) effector mechanisms to lead to survival or death (Palmer & Traylor-Knowles, 2012). The present investigation provides new data for the study of these pathways in *O. faveolata* by revealing components of evolutionarily conserved immune signaling pathways that regulate immune cell development, migration and host-microbe interactions.

### Wnt and Notch pathways in immune cell development, migration and communication

Gene families, such as Wnt and Notch, have been studied with respect to genome evolution and larval development in Cnidarians (Käsbauer et al., 2007; Kusserow et al., 2005; Marlow et al., 2012; Miller, Ball & Technau, 2005; Radtke, Fasnacht & MacDonald, 2010). It is also known that these genes are important regulators of the immune system through the control of immune cell differentiation, migration and communication (Duncan et al., 2005; Radtke, Fasnacht & MacDonald, 2010; Staal, Luis & Tiemessen, 2008). However, few studies have explored hypotheses about the role of Wnt and Notch in coral immune responses to disease. If one considers that pluripotent, phagocytic immune cells (i.e. coral amoebocytes) are at the front lines of wound healing and pathogen removal, it is likely that these genes regulate coral immune responses disease and stress. Therefore, pathways that have dual roles in the regulation of development and innate immunity should be researched further. To that end, we report novel members of the Wnt-like protein family and a complete gene set for the Notch pathway in the *O. faveolata* transcriptome.

**Figure 5:**
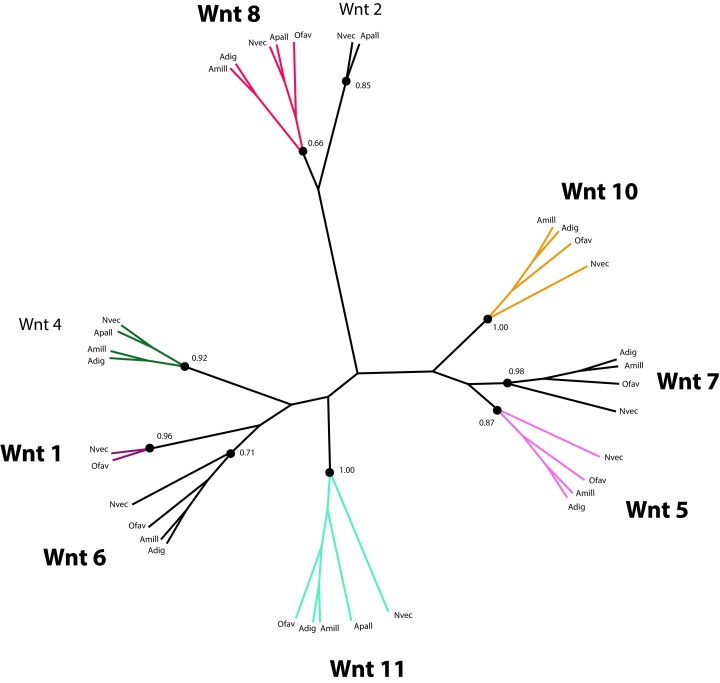
Phylogenetic analysis of Wnt-like protein sequences in *O. faveolata* transcriptome. Predicted protein sequences from *N. vectensis* (Nvec), *O. faveolata* (Ofav), *A. pallida* (Apall), *A. digitifera* (Adig), and *A. millepora* (Supplemental Information 11) were used for maximum likelihood phylogeny estimation with 100 bootstraps.

Wnt proteins are extracellular ligands of transmembrane receptors, Frizzled (FZD) and low-density Lipoprotein Receptor (LRP), that transduce signals to control the expression of target genes required for development in a β-catenin-dependent and independent manner (Gordon & Nusse, 2006; Logan & Nusse, 2004). Components of the Wnt pathway were first reported for Cnidarians in the species *Hydra vulgaris* (Hobmayer et al., 2000), from which time their role in Cnidarian larval development has been studied extensively (de Jong et al., 2006; Guder et al., 2006; Kortschak et al., 2003; Kusserow et al., 2005; Miller, Ball & Technau, 2005; Randall & Szmant, 2009; Technau et al., 2005a). Therefore, it is not surprising that a set of genes putatively involved in Wnt signaling is present in the *O. faveolata* transcriptomes (Supplemental Information 10). However, the role of Wnt-signaling in coral amoebocyte development during migration and differentiation has not been investigated. A number of Wnt-like predicted protein sequences were identified by annotation of the reference transcriptomes reported here and by Pinzón et al. (2015). The parallel annotation of other Cnidarian species identified Wnt-like sequences in the transcriptomes of the corals *Acropora millepora* (Kortschak et al., 2003; Moya et al., 2012) and *Acropora digitifera* (Shinzato et al., 2011), and the sea anemone, *Aiptasia pallida* (Lehnert, Burriesci & Pringle, 2012). Twelve Wnt proteins were originally identified in the *N. vectensis* genome (Kusserow et al., 2005). These sequences were included in the phylogenetic analysis of Wnt sequences to guide homology prediction for Wnt sequences in the *O. faveolata* transcriptome (Supplemental Information 11). Seven unique *O. faveolata* transcripts are reported here as putative homologues of the following *N. vectensis* Wnt proteins: Wnt1, Wnt5, Wnt6, Wnt7, Wnt8, Wnt10, and Wnt11 (Fig. 5). The extent to which Wnt protein expression is restricted to specific developmental life stages or environmental stimuli is not well understood in corals. Therefore, the proteins described here may only represent a subset of the complete repertoire encoded in the genome of this species. A complete genome sequence or a reference transcriptome derived from a more diverse set of tissue types and environmental conditions is required for an exhaustive assessment of *O. faveolata* Wnt-like genes. The present study is the first to characterize *O. faveolata* Wnt-like protein sequences. This new data can be used in future mechanistic studies on the role that Wnt protein family plays in the coral innate immune system.

**Figure 6:**
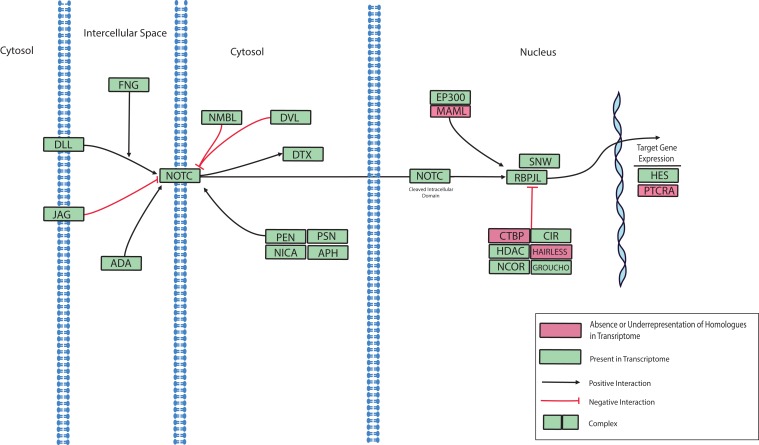
Modified *O. faveolata* Notch signaling pathway from KEGG. Delta-like ligand (DLL), protein jagged (JAG), disintegrin and metalloproteinase domain-containing protein 17 (ADA), O-fucosylpeptide 3-beta-N-acetylglucosaminyltransferase (FNG), neurogenic locus Notch homolog protein (NOTC), numb-like protein (NMBL), segment polarity protein dishevelled homolog (DVL), E3 ubiquitin-protein ligase DTX1 (DTX), presenilin enhancer protein 2 (PEN), nicastrin (NICA), presenilin-1 (PSN), anterior pharynx defective 1 (APH), E1A/CREB-binding protein (EP300), recombining binding protein suppressor of hairless (RBPJL), SNW domain-containing protein 1 (SNW), C-terminal binding protein (CTBP), hairless (HAIRLESS), nuclear receptor co-repressor 2 (NCOR), histone deacetylase 1 or 2 (HDAC), CBF1 interacting corepressor (CIR), groucho (GROUCHO), hairy and enhancer of split 1 (HES), pre T-cell antigen receptor alpha (PTCRA).

Notch signaling is involved in the regulation of cell identity, proliferation, differentiation and apoptosis (Gazave et al., 2009). Notch proteins are transmembrane receptors that detect delta-like ligands expressed on the surface of adjacent cells. On engagement of its ligand, the intracellular domain of Notch is cleaved and translocates to the nucleus where it serves as a transcription factor. Notch receptors have been described previously for various Cnidarians, including *Nematostella*, *Hydra*, and *Acropora* (Dunlap et al., 2013; Käsbauer et al., 2007; Marlow et al., 2012; Münder et al., 2010; Technau et al., 2005a). Inhibition of the Notch pathway was recently shown to disrupt wound healing in *N. vectensis* thus establishing a role for Notch in the regulation of innate immunity (DuBuc, Traylor-Knowles & Martindale, 2014). A role of Notch signaling in coral immune responses to disease is also supported by an RNA-Seq investigation that reported the differential expression of a Notch-like gene in response to acute exposure to bacterial Pathogen Associated Molecular Patterns (PAMPs) (Weiss et al., 2013). A putative Notch signaling pathway based on the presence or absence of pathway components in the *O. faveolata* transcriptome is presented in Fig. 6. This data can be used to investigate the role of Notch signaling in wound healing and immune responses to disease in *O. faveolata*.

### Intracellular host-microbe interactions

Signaling through innate immune pathways is activated on detection of extracellular PAMPs by Pattern Recognition Receptors (PRR). PRR-induced signals are transmitted by cytosolic kinases, such Mitogen Activated Protein Kinase (MAPK). MAPKs make up a highly conserved family of protein kinases that regulate immune signaling pathways (Chen et al., 2001; Schwarz et al., 2008). Signals transmitted by kinases activate pro-inflammatory gene expression by transcription factors that regulate effector responses, such as Nuclear Factor Kappa Beta (NF-κB) and Jun (Palmer & Traylor-Knowles, 2012). A complete prototypical inflammatory signaling cascade has been documented in *Hydra* in the context of host-associated microbiota. In this model, PAMP recognition by PRRs, such as Toll-Like Receptors (TLRs), activates MAPK signaling cascades through MyD88-dependent phosphorylation of Jun-Kinase (JNK). JNK in turn activates Jun and leads to the expression of pro-survival factors, such as Bcl-2 (Franzenburg et al., 2012). In *O. faveolata*, the expression of genes associated with this pathway has been detected under conditions of environmental stress (Schwarz et al., 2008; Voolstra et al., 2009). Mechanistic studies to confirm a functional role for these genes in *O. faveolata* immune responses to disease can now be conducted with the use of full-length gene sequences reported here and by Pinzón et al. (2015).

Host cells can also detect PAMPs associated with intracellular pathogens by Nod-Like Receptors (NLRs) and Rig-Like Receptors (RLRs) (MacKay, Wang & Kurt-Jones, 2014; Ting et al., 2008; Yoneyama & Fujita, 2007). NLRs detect components of bacterial cell walls and RLRs detect cytosolic viral nucleic acids. Various bacterial pathogens of corals, including the causative agents of CYBD, invade the host cytoplasm (Cervino et al., 2008; Cervino et al., 2004; Kushmaro et al., 2001). Therefore, NLRs should be investigated in coral immune responses to infectious disease. Recent investigations have also demonstrated the important roles that viruses play in pathogenesis and symbiosis (Atad et al., 2012; Barr et al., 2013; Davy et al., 2006; Soffer et al., 2013; Weynberg et al., 2015; Wilson et al., 2005). Therefore, RLRs, and the related dicer-like protein family, should be investigated to elucidate their role in the control of host interactions with symbiotic, commensal and pathogenic viruses. To support future studies along these lines, the present investigation identifies components of NLR, RLR and Dicer signaling pathways present in the *O. faveolata* transcriptome.

### Nod-like receptors and intracellular bacterial detection

**Figure 7:**
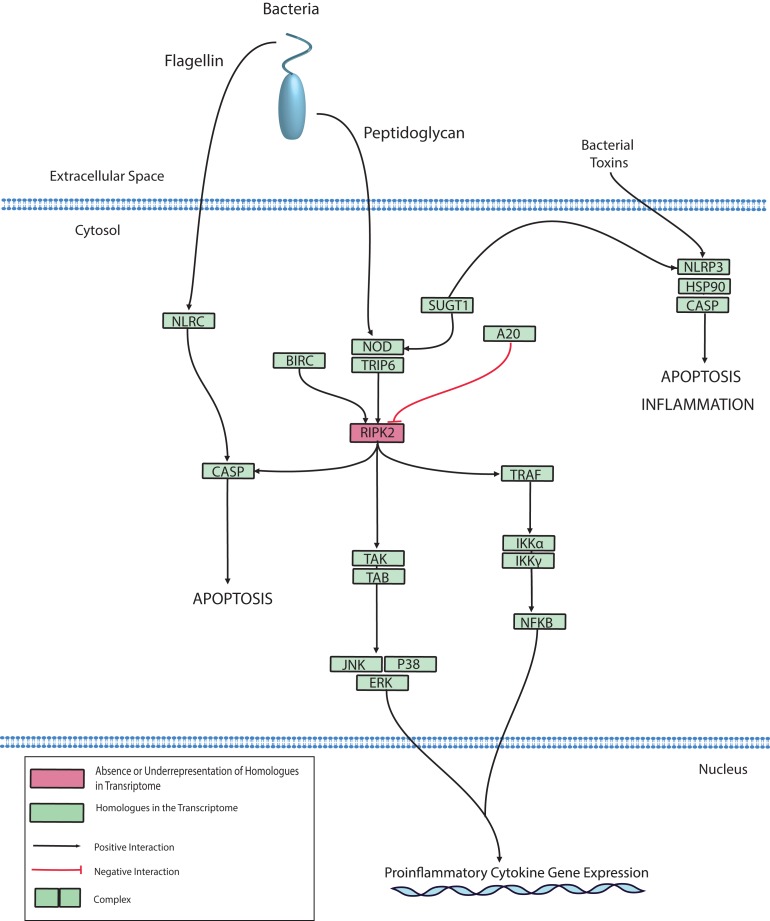
Modified *O. faveolata* NLR signaling pathway from KEGG. NLR family CARD domain-containing protein 4 (NLRC), NACHT, LRR and PYD domains-containing protein 3 (NLRP3), suppressor of G2 allele of SKP1 (SUGT1), receptor-interacting serine/threonine-protein kinase 2 (RIPK2), mitogen-activated protein kinase kinase kinase 7 (TAK), TAK1-binding protein 1 (TAB), c-Jun N-terminal kinase (JNK), mitogen-activated protein kinase 1 or 3 (ERK), p38 MAP kinase (p38), inhibitor of nuclear factor kappa-B kinase subunit gamma (IKKγ), inhibitor of nuclear factor kappa-B kinase subunit alpha (IKKα), nuclear factor kappa beta (Nfκβ), TNF receptor-associated factor 6 (TRAF), Caspase (CASP), baculoviral IAP repeat-containing protein (BIRC), tumor necrosis factor alpha-induced protein 3 (A20).

The NLR protein family has conserved roles in regulating innate immunity through the recognition of intracellular bacteria by Leucine-Rich Repeat (LRR) domains. LRR domains are also hallmarks of membrane-bound TLRs, however, NLRs are located exclusively in the cytoplasm (Ting et al., 2008). After pathogen recognition, NLRs can promote caspase-mediated cell death through the macromolecular assembly of the inflammasome, which converts inactive procaspase to active caspase by proteolysis. Caspase-mediated cell death has been reported in corals and Cnidarians and has been described in depth by multiple reviews (Cikala et al., 1999; Lasi et al., 2010; Tchernov et al., 2011; Weis, 2008). Alternatively, NLRs can mediate inflammation through the aforementioned MAPK, JNK, or NFκB pathways. Genes homologous to NLRs have been identified in basal metazoans including *Hydra*, *Nematostella, Acropora*, *Amphimedon* (Augustin, Fraune & Bosch, 2010; Bosch, 2012; Hamada et al., 2013; Yuen, Bayes & Degnan, 2014). In the *Hydra* model system, there is evidence to support a role for NLR in bacterial detection and caspase activation (Bosch et al., 2011; Lange et al., 2011). Therefore, it is not surprising that we also identified NLR homologues in the *O. faveolata* transcriptomes. A putative *O. faveolata* NLR signaling pathway is constructed to guide future investigations on coral immune responses to intracellular bacterial pathogens (Fig. 7).

### Detection of viral nucleic acids

To date, interactions between the coral host and associated microbes have largely focused on coral-fungal and coral-bacterial interactions (Kim et al., 2000; Kvennefors et al., 2008; Sutherland, Porter & Torres, 2004). However, viruses have emerged as important members of the coral holobiont and can have beneficial or harmful affects on the coral host (Van Oppen, Leong & Gates, 2009, Weynberg et al., 2015). While bacteriophages have been demonstrated to eradicate coral pathogens and prevent infection (Barr et al., 2013; Efrony et al., 2007), herpes-like viruses have been associated with virulent coral diseases (Soffer et al., 2013; Thurber et al., 2008). Therefore, recognition of viruses and regulation of their activities by the coral immune system is hypothesized to be essential in maintaining homeostasis.

**Figure 8:**
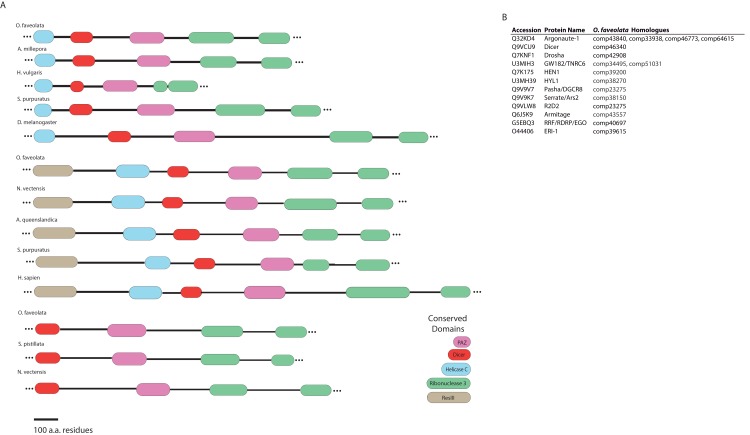
Domain architecture of dicer-like protein sequences derived from the *O. faveolata* transcriptome. (A) Hmmscan analysis of protein domain architecture for *O. faveolata* predicted protein sequences. Dicer-like protein with similar domain architectures are also shown (Supplemental Information 12) (B) miRNA and siRNA pathway components present in the *O. faveolata* transcriptome.

**Figure 9:**
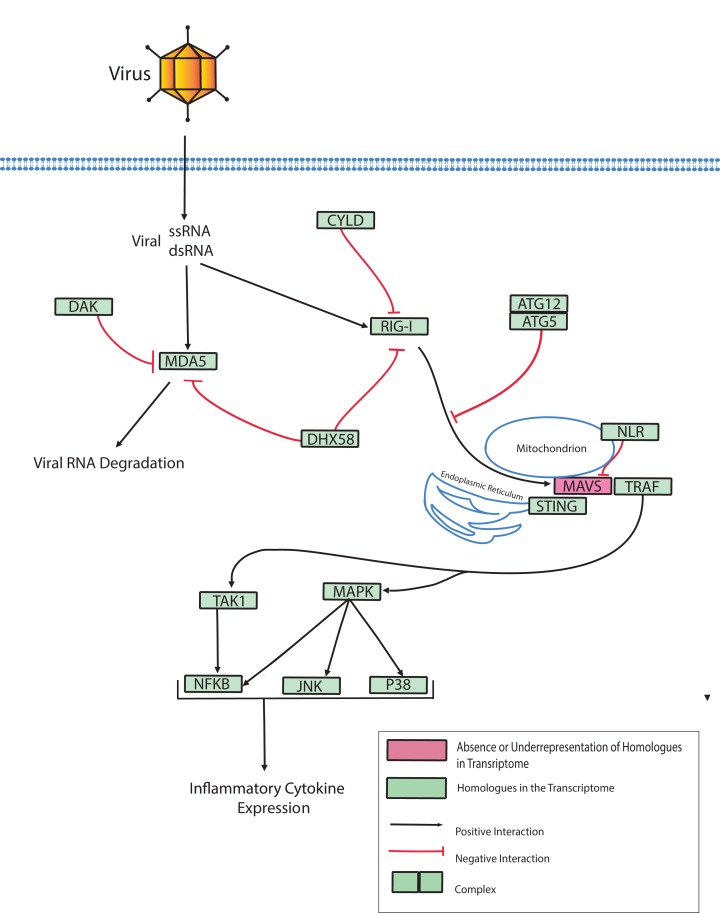
Modified *O. faveolata* RLR pathway from KEGG. Ubiquitin thioesterase CYLD (CYLD), ATP-dependent RNA helicase DDX58 (RIG-I), interferon-induced helicase C domain-containing protein 1 (MDA5), dihydroxyacetone kinase (DAK), ATP-dependent RNA helicase DHX58 (DHX58), autophagy-related protein 5 (ATG5), autophagy-related protein (ATG12), Nod-like receptor (NLR), mitochondrial antiviral-signaling protein (MAVS), transmembrane protein 173 (STING), TNF receptor-associated factor 3 (TRAF), mitogen-activated protein kinase kinase kinase 7 (TAK), TAK1-binding protein 1 (TAB), c-Jun N-terminal kinase (JNK), mitogen-activated protein kinase 1 or 3 (ERK), p38 MAP kinase (p38), nuclear factor kappa beta (NFKB), mitogen-activated protein kinase kinase kinase 1 (MAPK).

Dicer-like proteins, which share protein domains with RLRs, are essential members of the Micro RNA (miRNA) and Small Interfering RNA (siRNA) pathways. They have only recently gained attention in corals, and they may have conserved roles in antiviral immunity (Liew et al., 2014; MacKay, Wang & Kurt-Jones, 2014; Moran et al., 2013). The present study identifies full-length Dicer-like proteins and the essential components for siRNA and miRNA signaling in the *O. faveolata* transcriptomes (Fig. 8). The dicer-like protein sequences used in this study are provided in a supplementary file (Supplemental Information 12). RLRs have evolutionarily-conserved roles in recognition of viral nucleic acids (Loo & Gale, 2011; Mukherjee, Korithoski & Kolaczkowski, 2014; Zou et al., 2009). Homologues of RLR-like genes have been reported for *Nematostella* in studies of RLR evolution from basal metazoans to chordates (Zou et al., 2009), but their function has not been investigated in Cnidarians. The *O. faveolata* reference transcriptome has many of the evolutionarily conserved components required for RLR signaling, and a putative pathway is presented in Fig. 9 to guide future research on coral immune responses to viral infection.

## Conclusions

The present investigation expands the bioinformatic resources available for the Caribbean reef-building coral, *O. faveolata.* Putative immunity genes in the coral transcriptome were identified by annotation with gene ontologies and KEGG orthologies. It is well established that Wnt-like proteins are important in coral larval development, but their role in immune cell development and migration remains largely uninvestigated. Phylogenetic analysis of Wnt-like predicted proteins sequences revealed seven novel family members in the *O. faveolata* transcriptome, which can be used to guide future research on their function in coral innate immunity. Components of the Notch, Nod-like, Dicer-like, and Rig-like signaling pathways reveal possible mechanisms for host cell communication, wound healing, intracellular bacterial and viral recognition. The results of the present investigation provide new data to advance the field of coral innate immunity, which has the ultimate goal of understanding the biological mechanisms that confer resistance or susceptibility of corals to climate-driven stress events and disease outbreaks.

## Supplemental Information

10.7717/peerj.1616/supp-1 Supplemental Information 1 *O. faveolata s*amples collected and their conditions.

10.7717/peerj.1616/supp-2 Supplemental Information 2 RNA-SeQC results for raw and processed sequence reads used for transcriptome assembly.

10.7717/peerj.1616/supp-3 Supplemental Information 3 Details on the sources of all transcriptome sequences used for analyses in the present investigation.

10.7717/peerj.1616/supp-4 Supplemental Information 4 *O. faveolata* transcriptome from the present investigation.

10.7717/peerj.1616/supp-5 Supplemental Information 5 Predicted protein sequences derived from the *O. faveolata* transcriptome reported in the present investigation.

10.7717/peerj.1616/supp-6 Supplemental Information 6 *O. faveolata* EST sequences downloaded form NCBI and used in the present investigation.

10.7717/peerj.1616/supp-7 Supplemental Information 7 Analysis of sequence accuracy for a sample of sequences from the transcriptome reported in the present study by comparison to *O. faveolata* ESTs in NCBI.

10.7717/peerj.1616/supp-8 Supplemental Information 8 Full BLAST2GO annotation of the *O. faveolata* transcriptome reported in the present investigation with Gene Ontology terms and InterPro scan accession numbers.

10.7717/peerj.1616/supp-9 Supplemental Information 9 Full KEGG KAAS mapping of *O. faveolata* transcripts from the present investigation to KEGG pathway maps.

10.7717/peerj.1616/supp-10 Supplemental Information 10 Full KEGG KAAS Annotation of the *O. faveolata* transcriptome reported in the present investigation.

10.7717/peerj.1616/supp-11 Supplemental Information 11 Components of the Wnt signaling pathway present in the *O. faveolata* transcriptome reported in the present investigation. *O. faveolata* components were identified by BLASTp alignments to human components of the KEGG Wnt pathway.

10.7717/peerj.1616/supp-12 Supplemental Information 12 All Wnt sequences used in the Wnt phylogenetic analysis.

10.7717/peerj.1616/supp-13 Supplemental Information 13 All sequences used in the Dicer protein domain architecture analysis.

## Additional Information and Declarations

### Field Study Permissions

The following information was supplied relating to field study approvals (i.e., approving body and any reference numbers): Samples were collected under a general collecting permit issued by the Department of Natural Resources of Puerto Rico to the Department of Marine Sciences at the University of Puerto Rico at Mayaguez.

### DNA Deposition

The following information was supplied regarding the deposition of DNA sequences: NCBI Short sequence read archive BioProject PRJNA236103. Assembled transcriptome and annotations provided as Supplemental Information.
