# Supplementary material for: RNA-Seq of the Caribbean reef-building coral Orbicella faveolata (Scleractinia-Merulinidae) under bleaching and disease stress expands models of coral innate immunity
Source: PeerJ. 2016 Feb 15;4:e1616. doi: 10.7717/peerj.1616 (PMC4768675; doi:10.7717/peerj.1616)

Pooled Raw Reads | Processed Reads for Assembly

%GC Content Across Reads

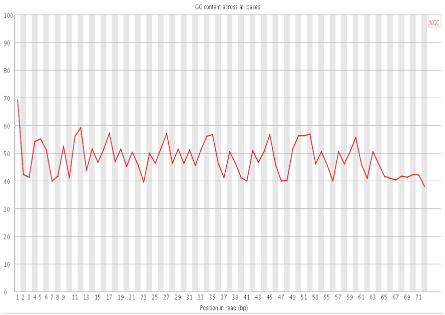

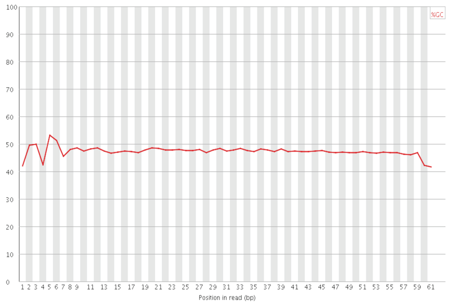

Quality Scores Per Base

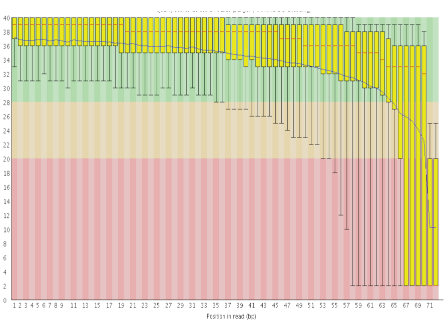

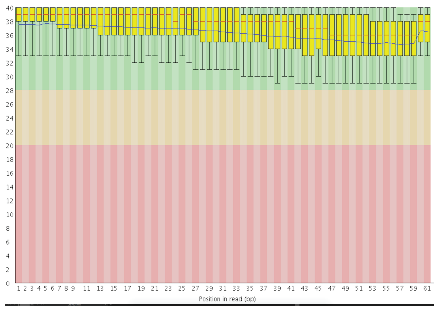

%GC Per Read Frequency Distrubution

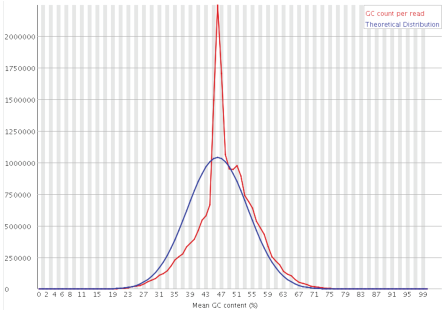

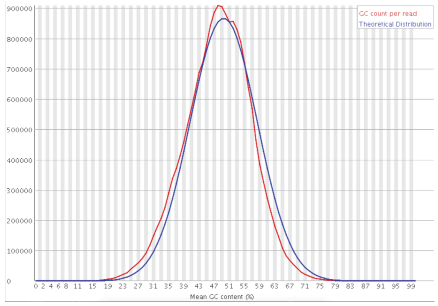

Supplement: Supplemental Information 2 [file peerj-04-1616-s002.pdf]
