# Supplementary figures and images for: RNA-Seq of the Caribbean reef-building coral Orbicella faveolata (Scleractinia-Merulinidae) under bleaching and disease stress expands models of coral innate immunity (part 2 of 2)
Source: PeerJ. 2016 Feb 15;4:e1616. doi: 10.7717/peerj.1616 (PMC4768675; doi:10.7717/peerj.1616)

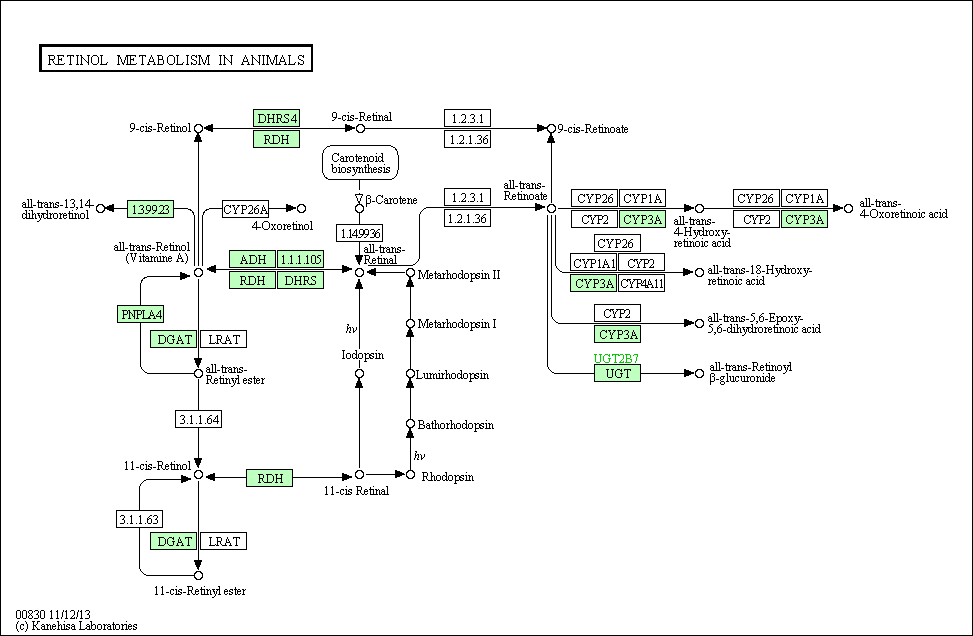

Supplement: Supplemental Information 9 [file peerj-04-1616-s009.gz › map/map00830.png]

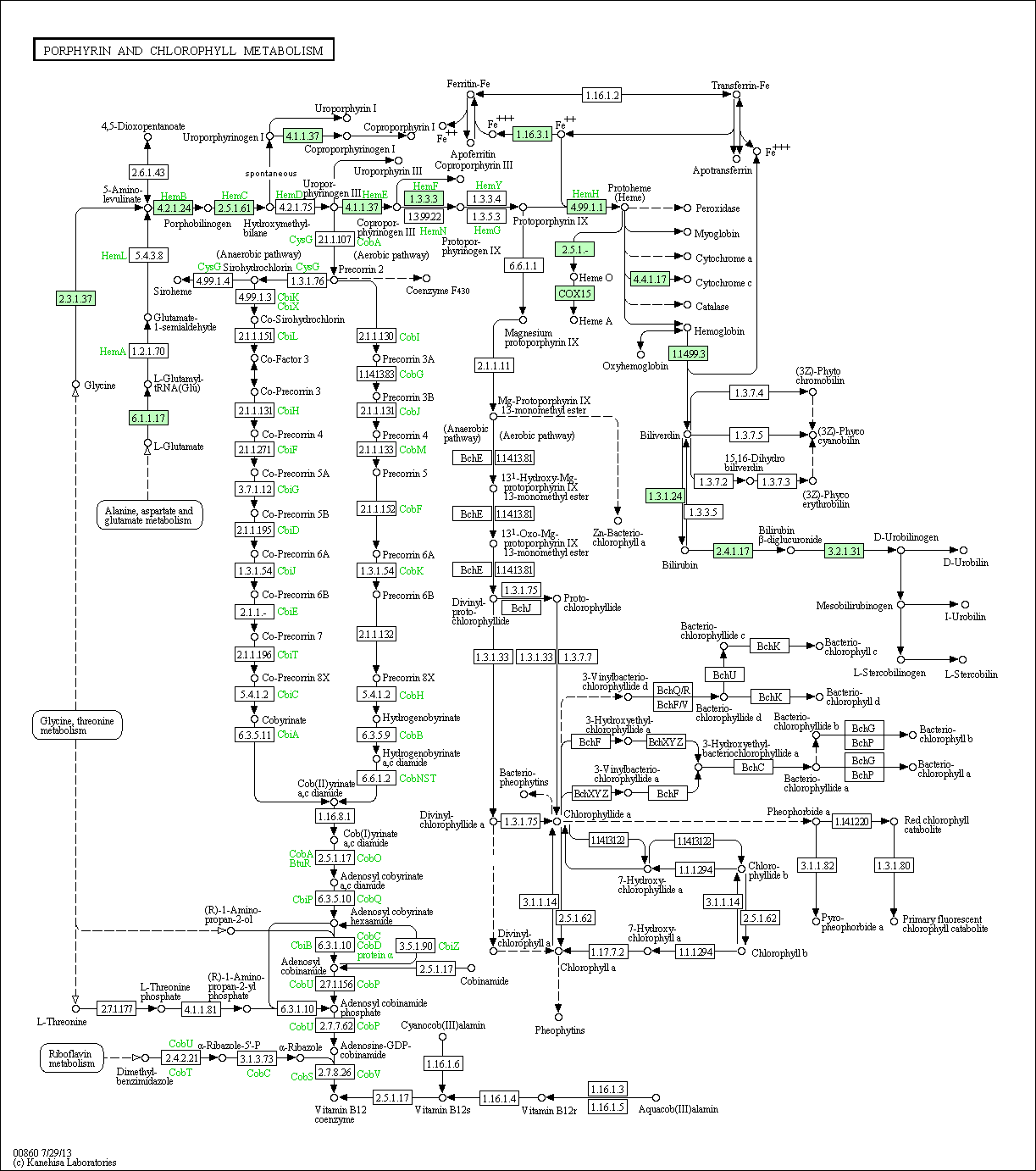

Supplement: Supplemental Information 9 [file peerj-04-1616-s009.gz › map/map00860.png]

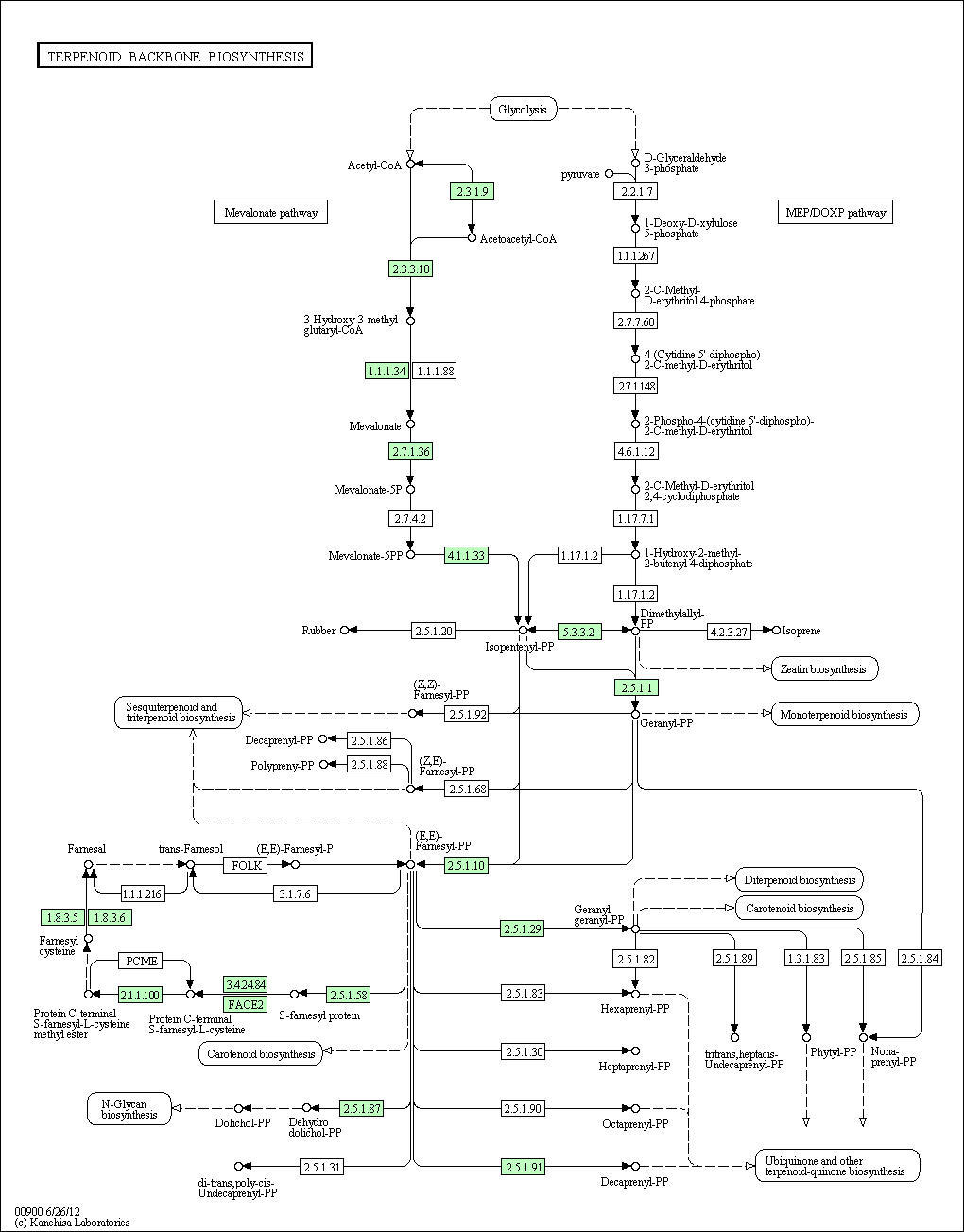

Supplement: Supplemental Information 9 [file peerj-04-1616-s009.gz › map/map00900.png]

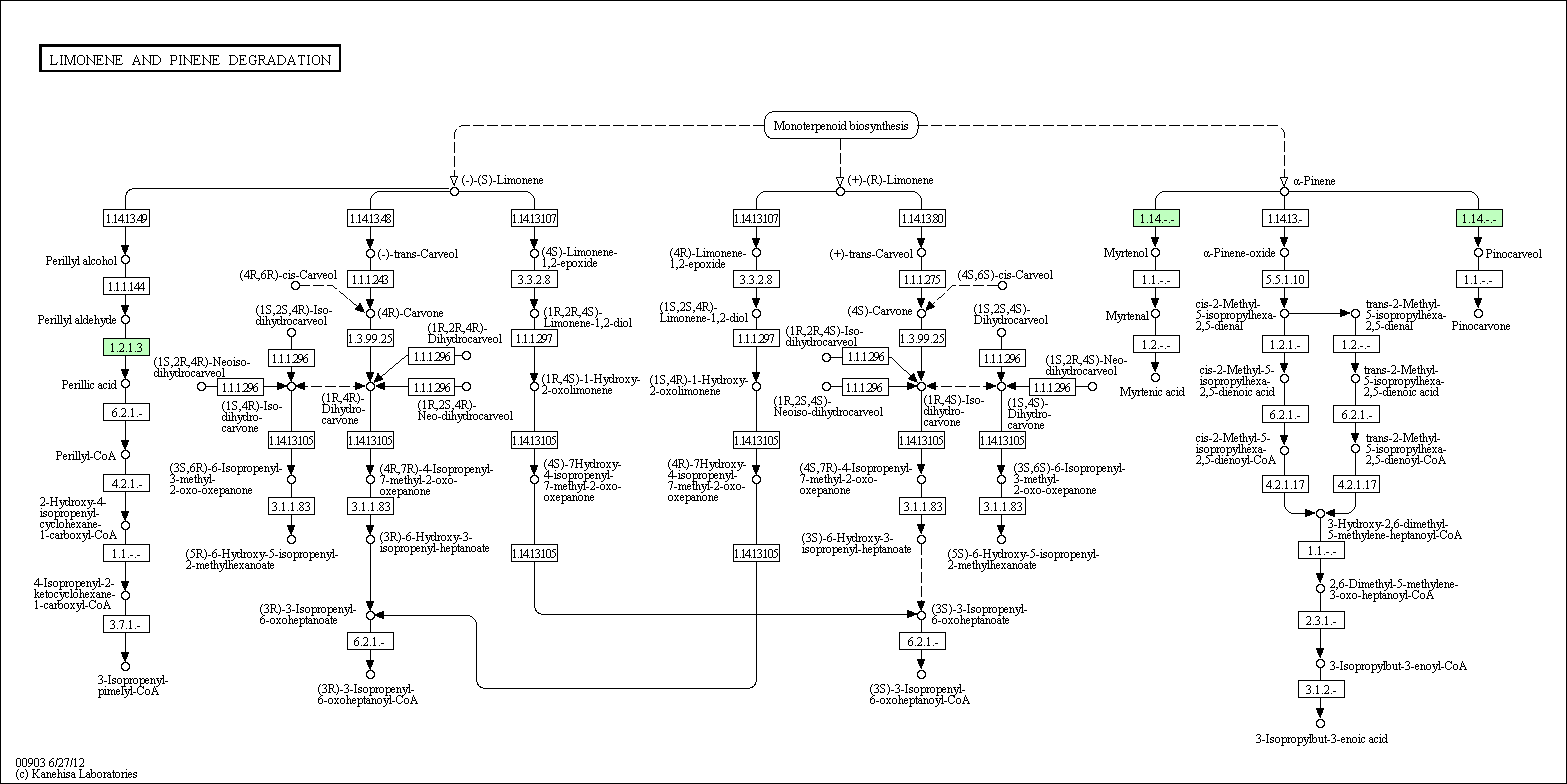

Supplement: Supplemental Information 9 [file peerj-04-1616-s009.gz › map/map00903.png]

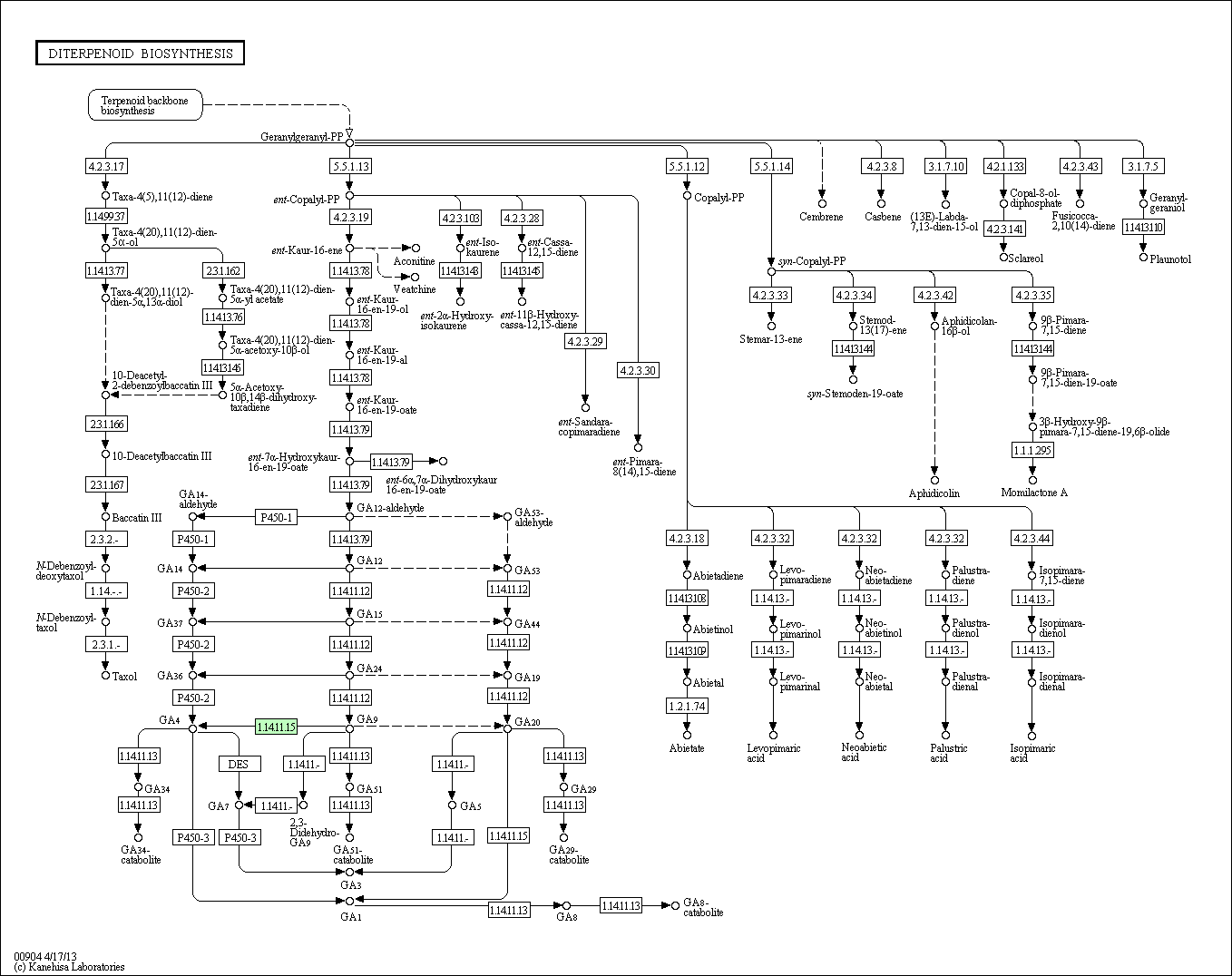

Supplement: Supplemental Information 9 [file peerj-04-1616-s009.gz › map/map00904.png]

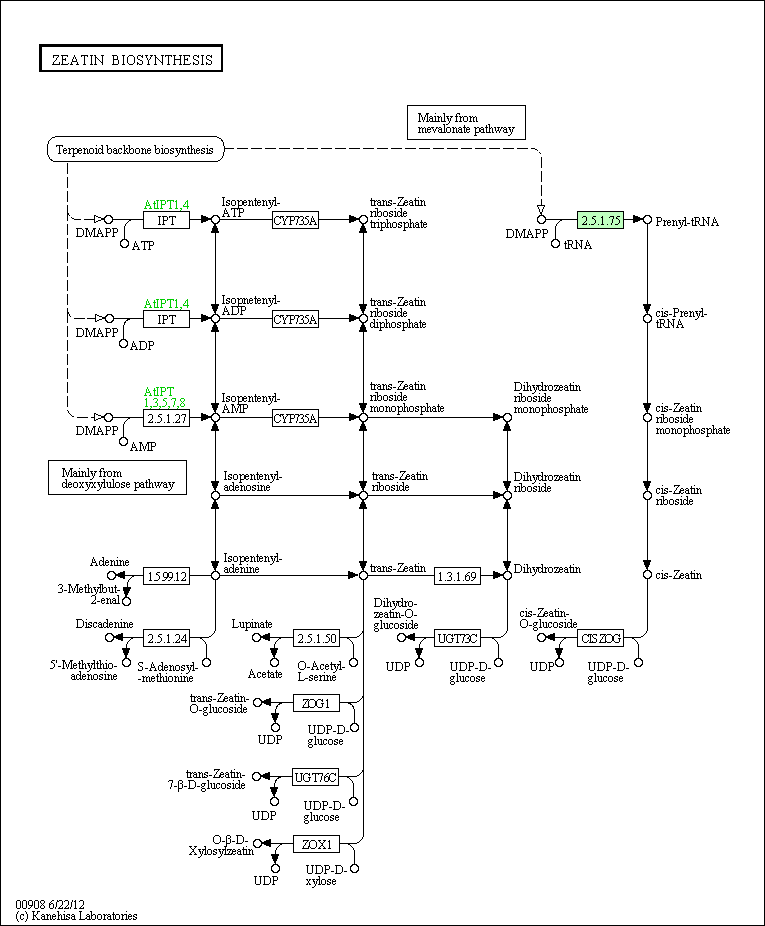

Supplement: Supplemental Information 9 [file peerj-04-1616-s009.gz › map/map00908.png]

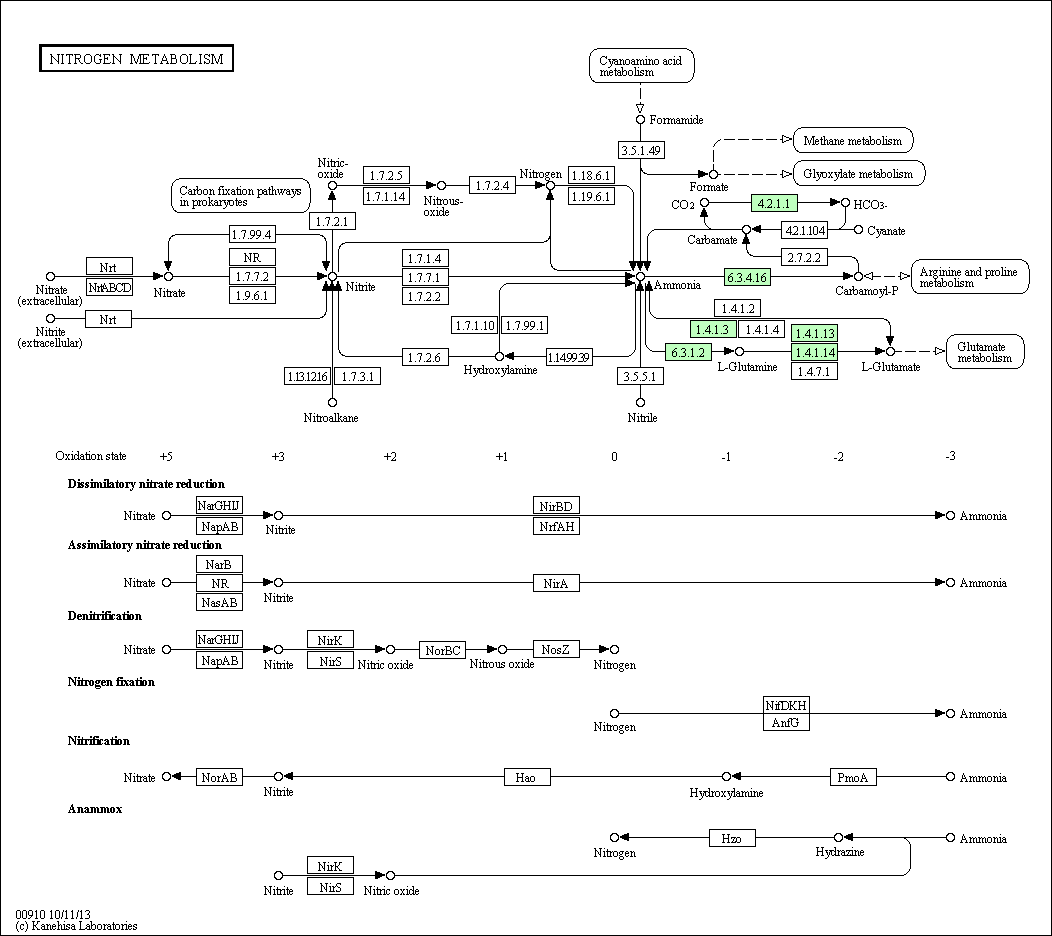

Supplement: Supplemental Information 9 [file peerj-04-1616-s009.gz › map/map00910.png]

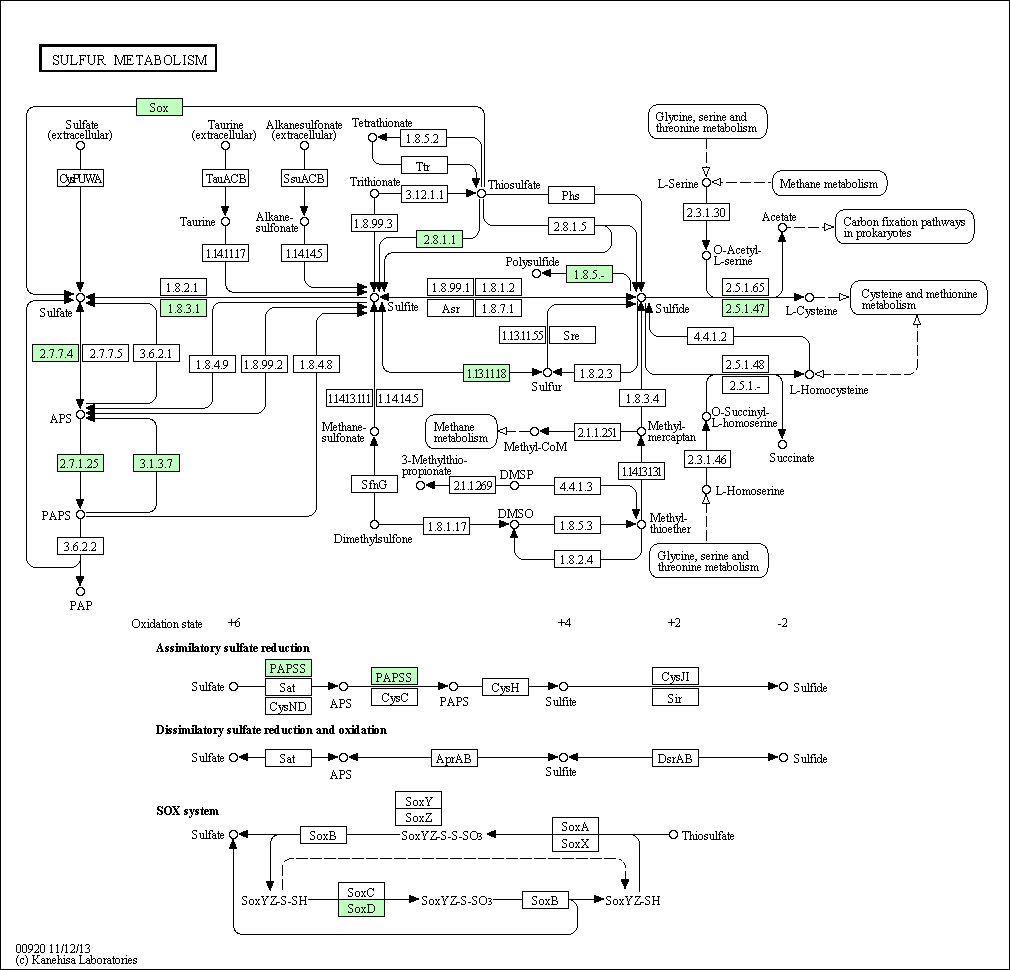

Supplement: Supplemental Information 9 [file peerj-04-1616-s009.gz › map/map00920.png]

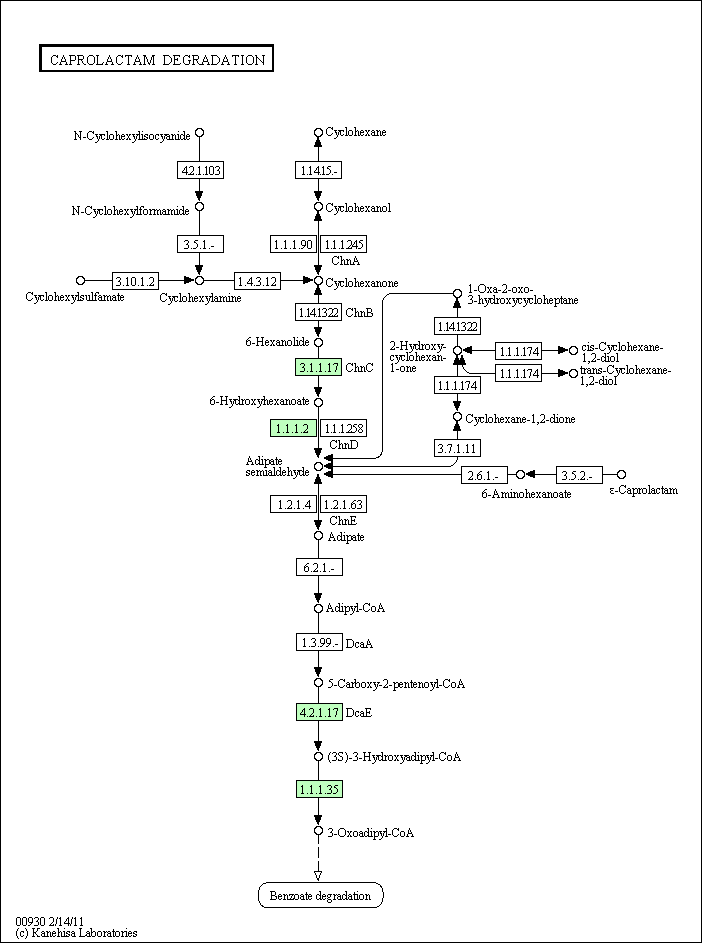

Supplement: Supplemental Information 9 [file peerj-04-1616-s009.gz › map/map00930.png]

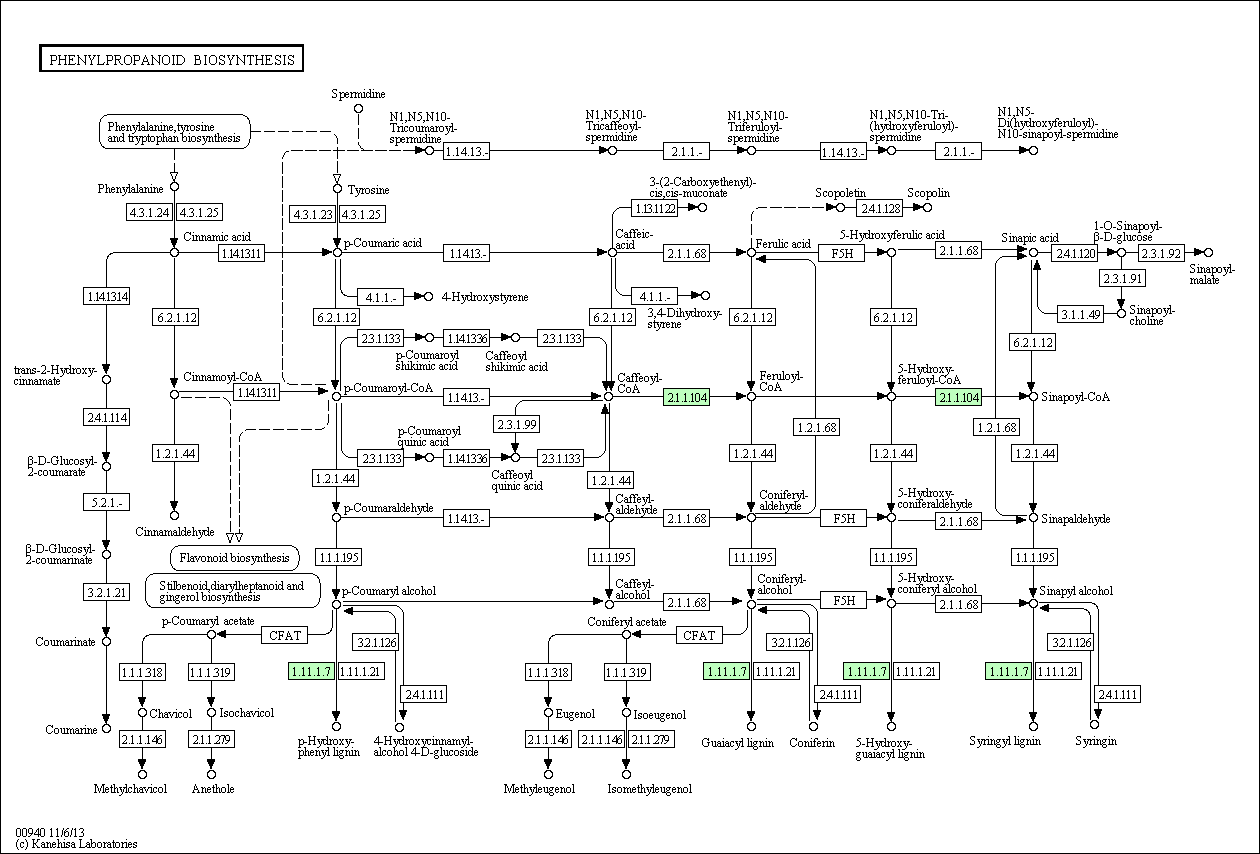

Supplement: Supplemental Information 9 [file peerj-04-1616-s009.gz › map/map00940.png]

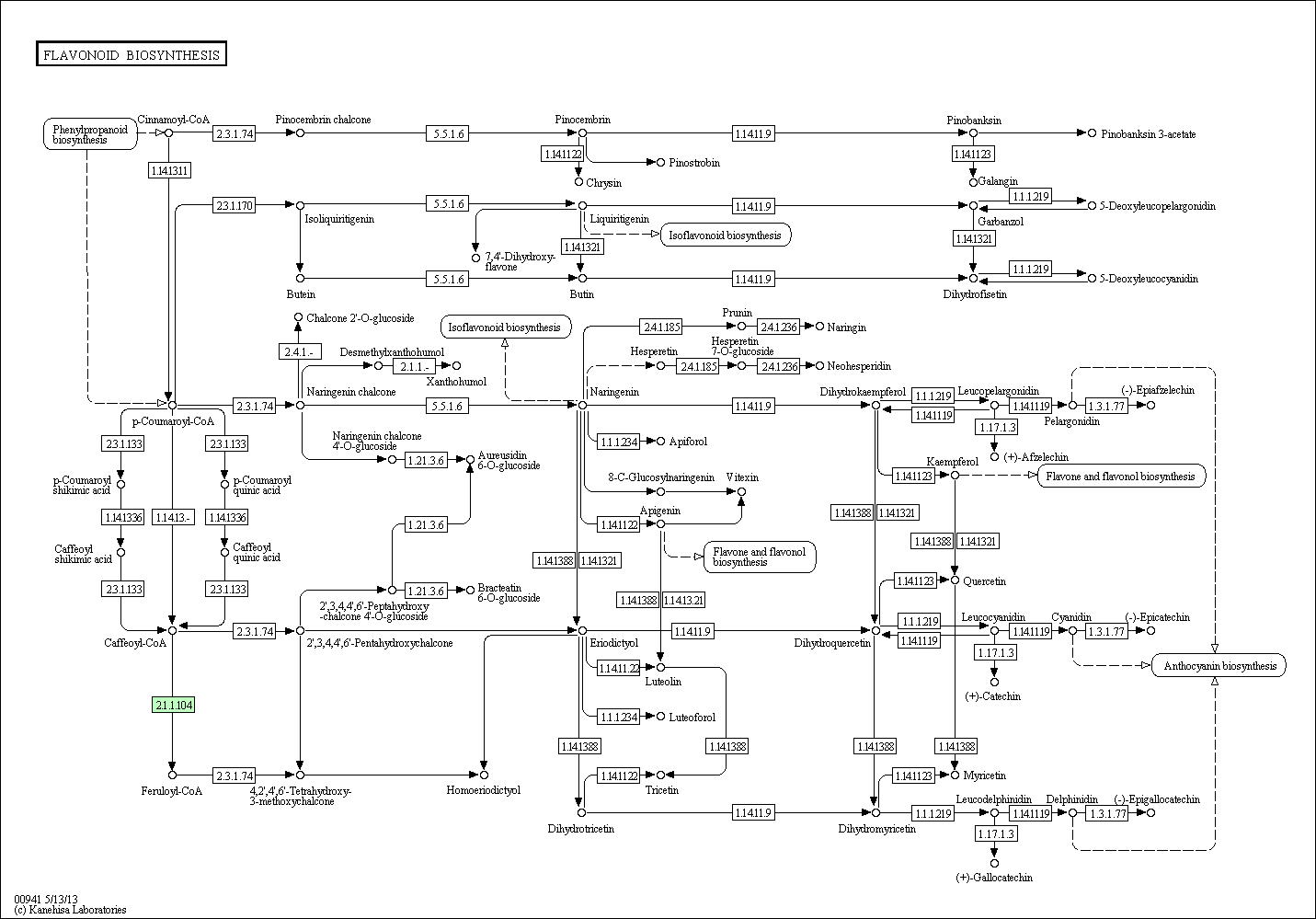

Supplement: Supplemental Information 9 [file peerj-04-1616-s009.gz › map/map00941.png]

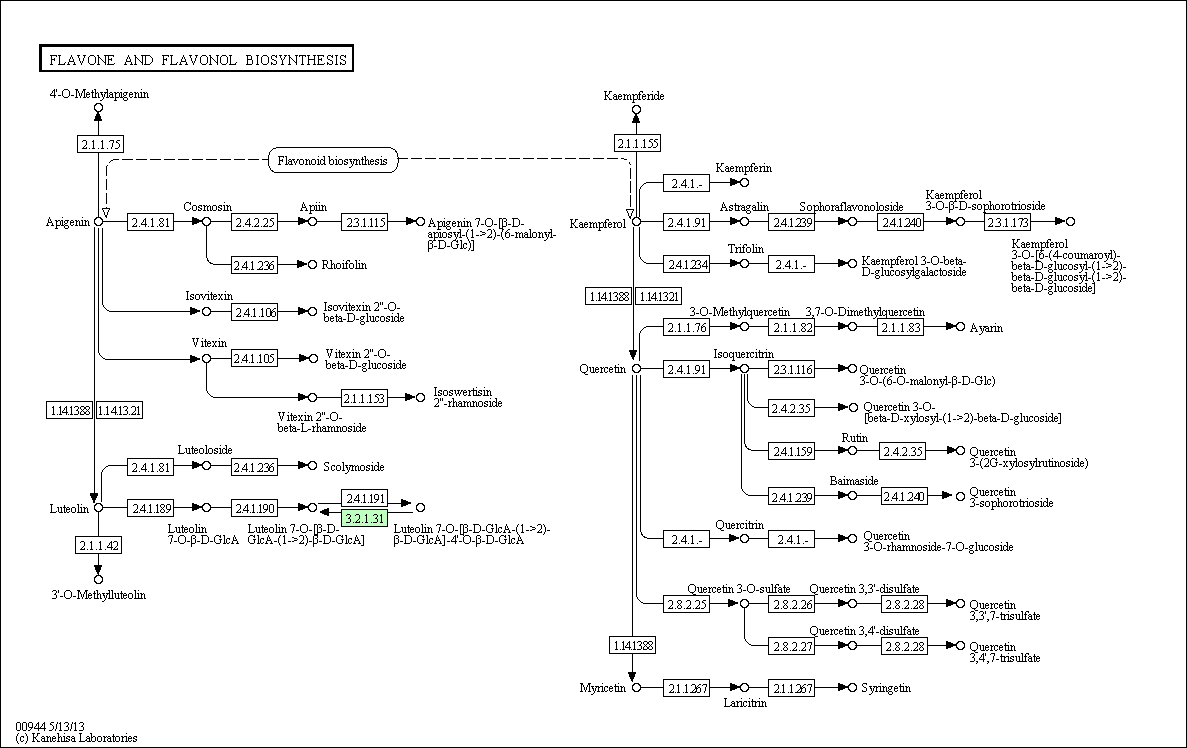

Supplement: Supplemental Information 9 [file peerj-04-1616-s009.gz › map/map00944.png]

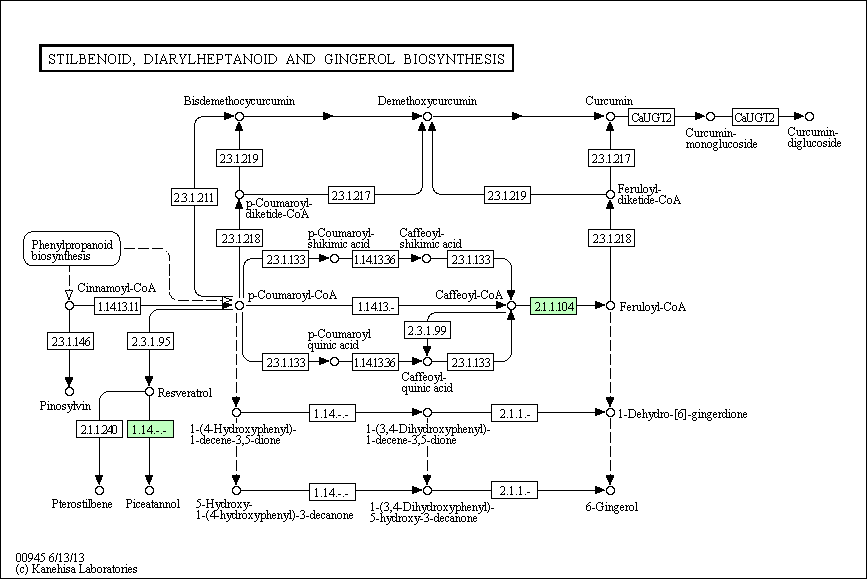

Supplement: Supplemental Information 9 [file peerj-04-1616-s009.gz › map/map00945.png]

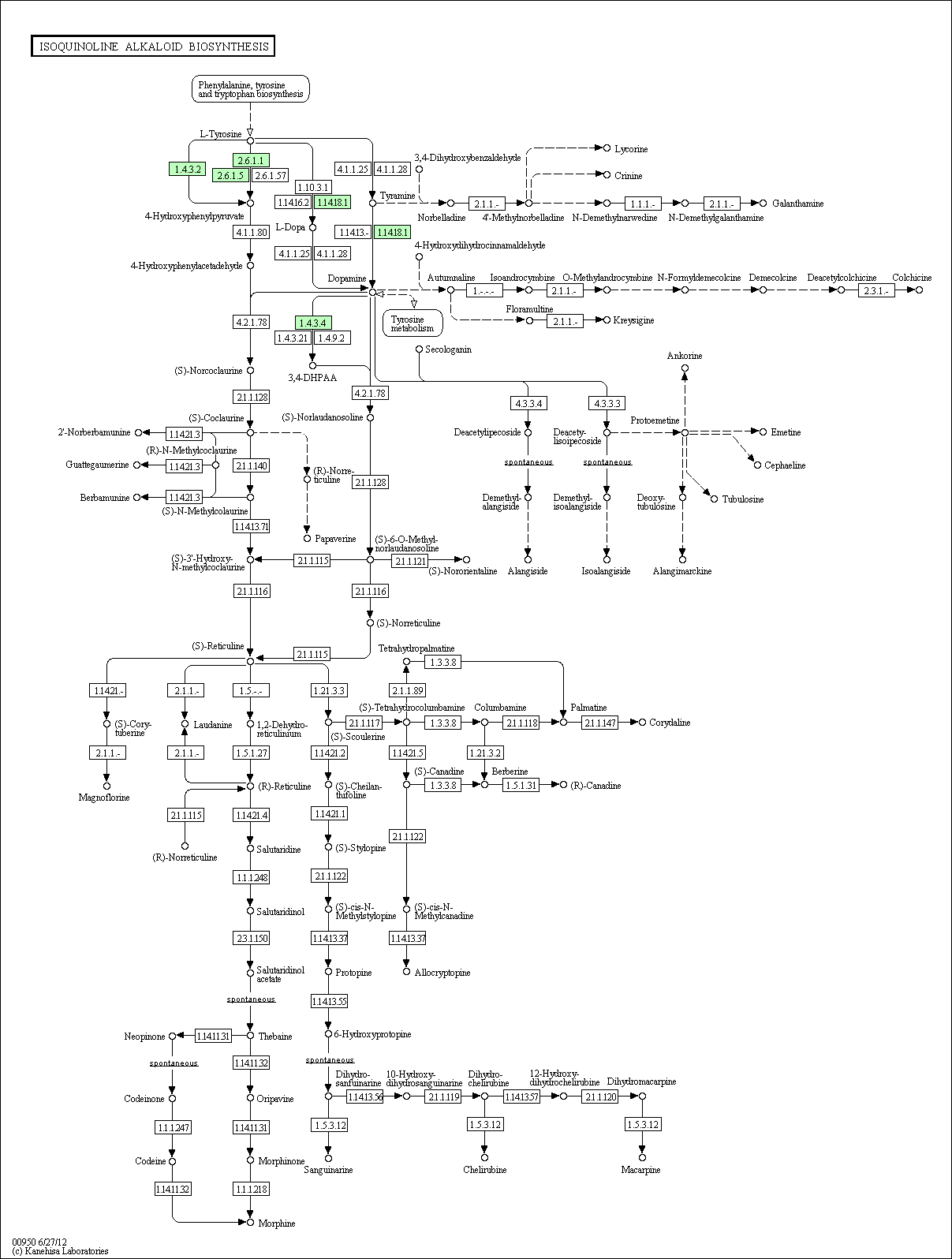

Supplement: Supplemental Information 9 [file peerj-04-1616-s009.gz › map/map00950.png]

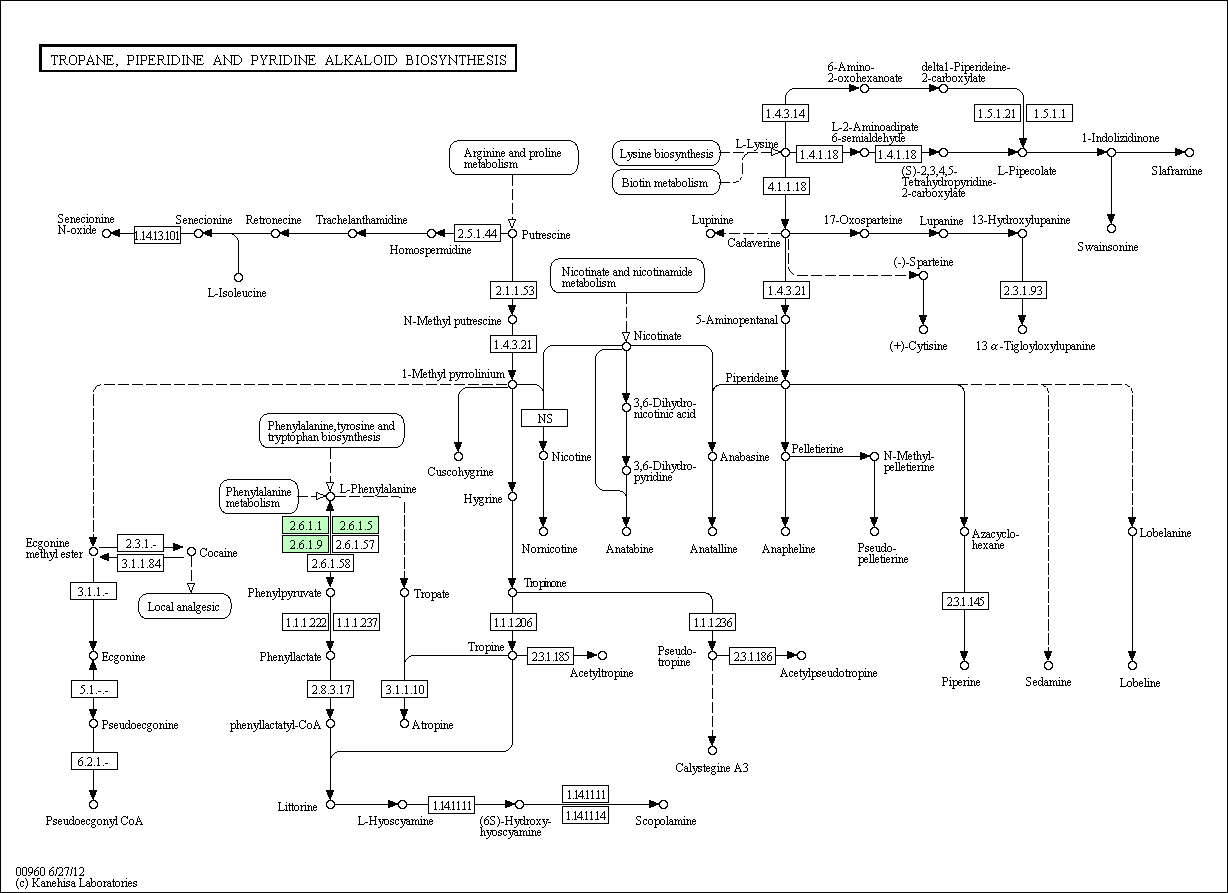

Supplement: Supplemental Information 9 [file peerj-04-1616-s009.gz › map/map00960.png]

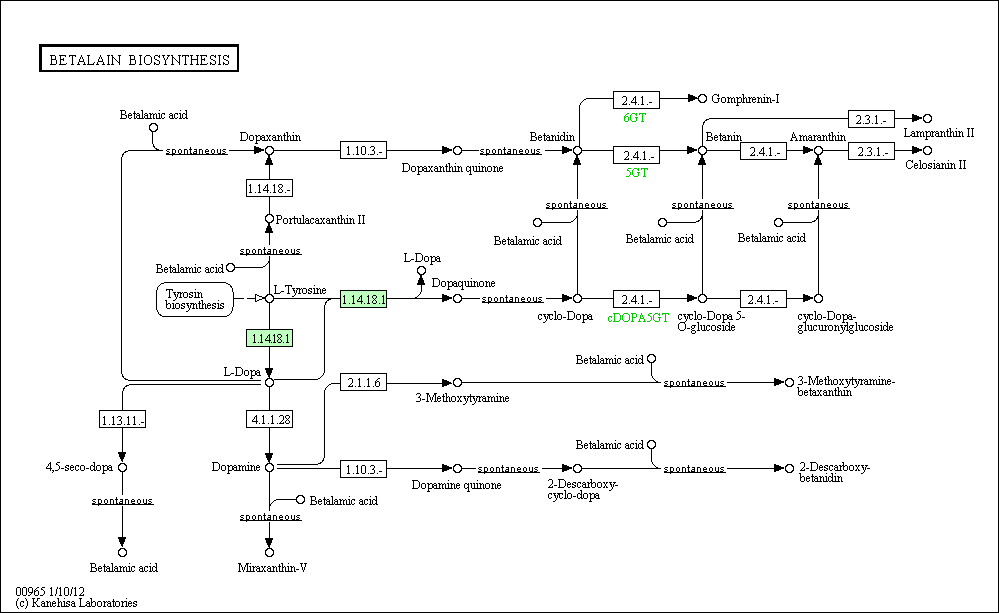

Supplement: Supplemental Information 9 [file peerj-04-1616-s009.gz › map/map00965.png]

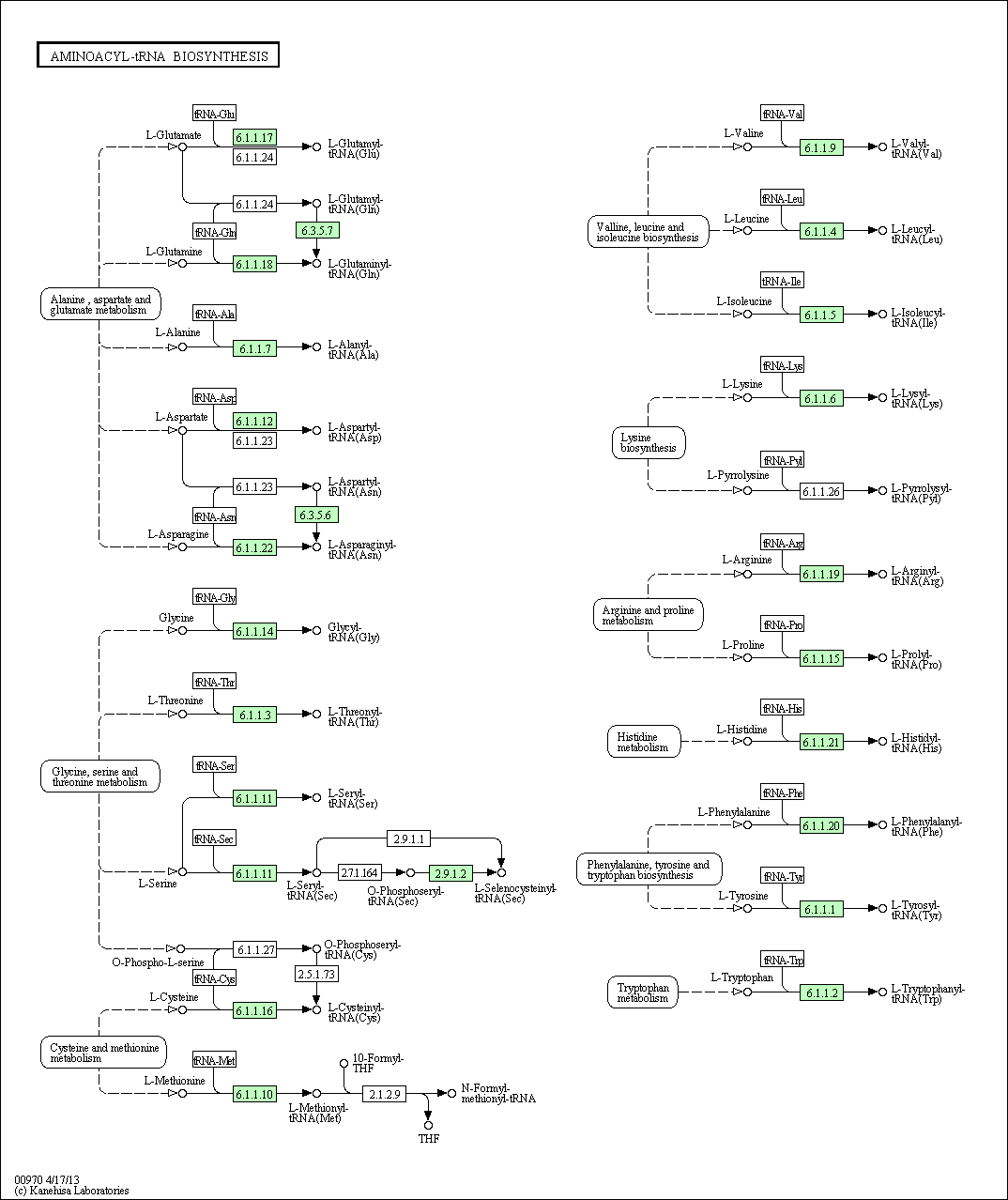

Supplement: Supplemental Information 9 [file peerj-04-1616-s009.gz › map/map00970.png]

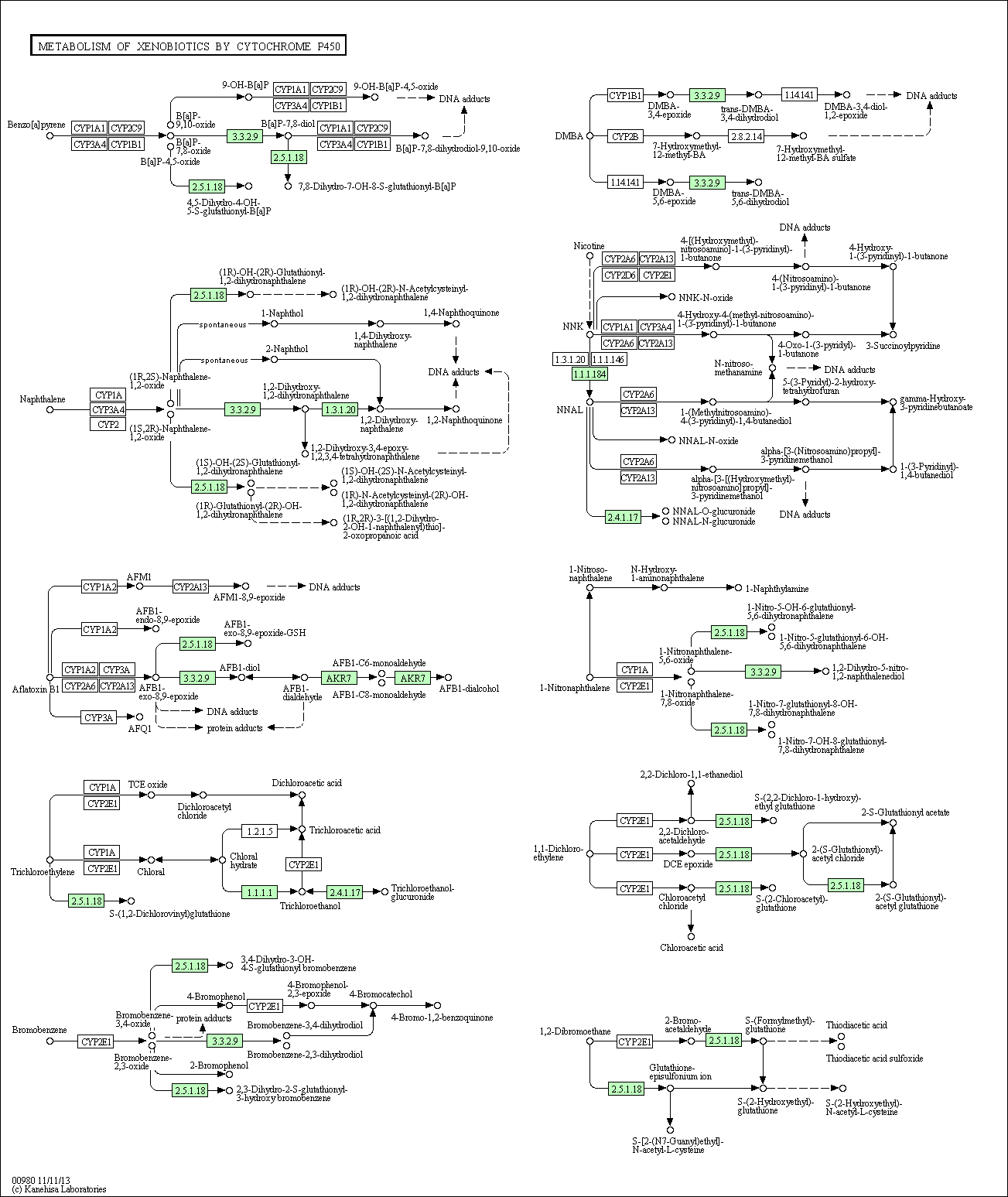

Supplement: Supplemental Information 9 [file peerj-04-1616-s009.gz › map/map00980.png]

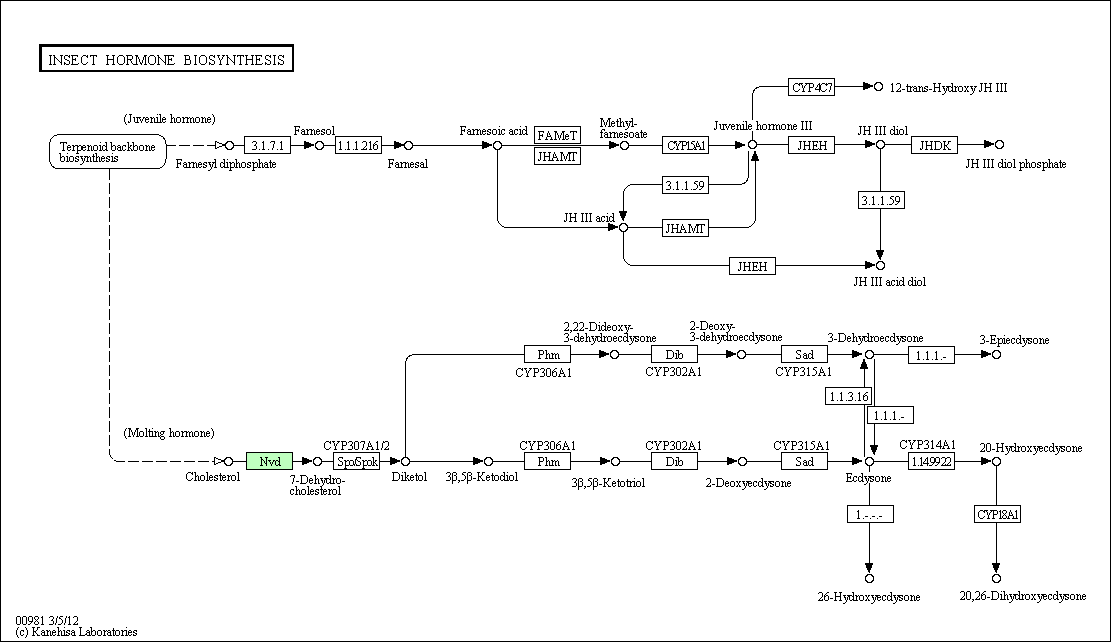

Supplement: Supplemental Information 9 [file peerj-04-1616-s009.gz › map/map00981.png]

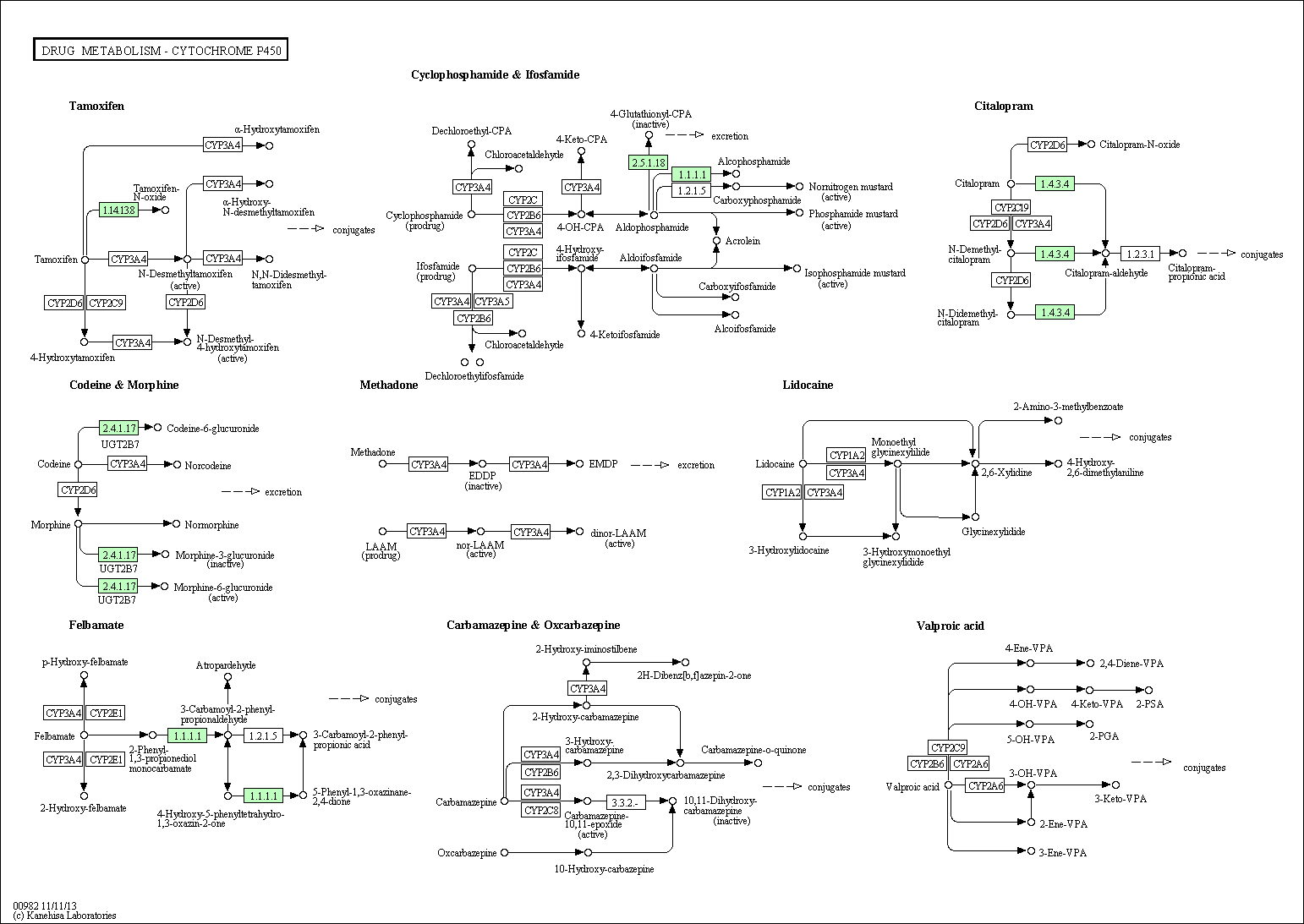

Supplement: Supplemental Information 9 [file peerj-04-1616-s009.gz › map/map00982.png]

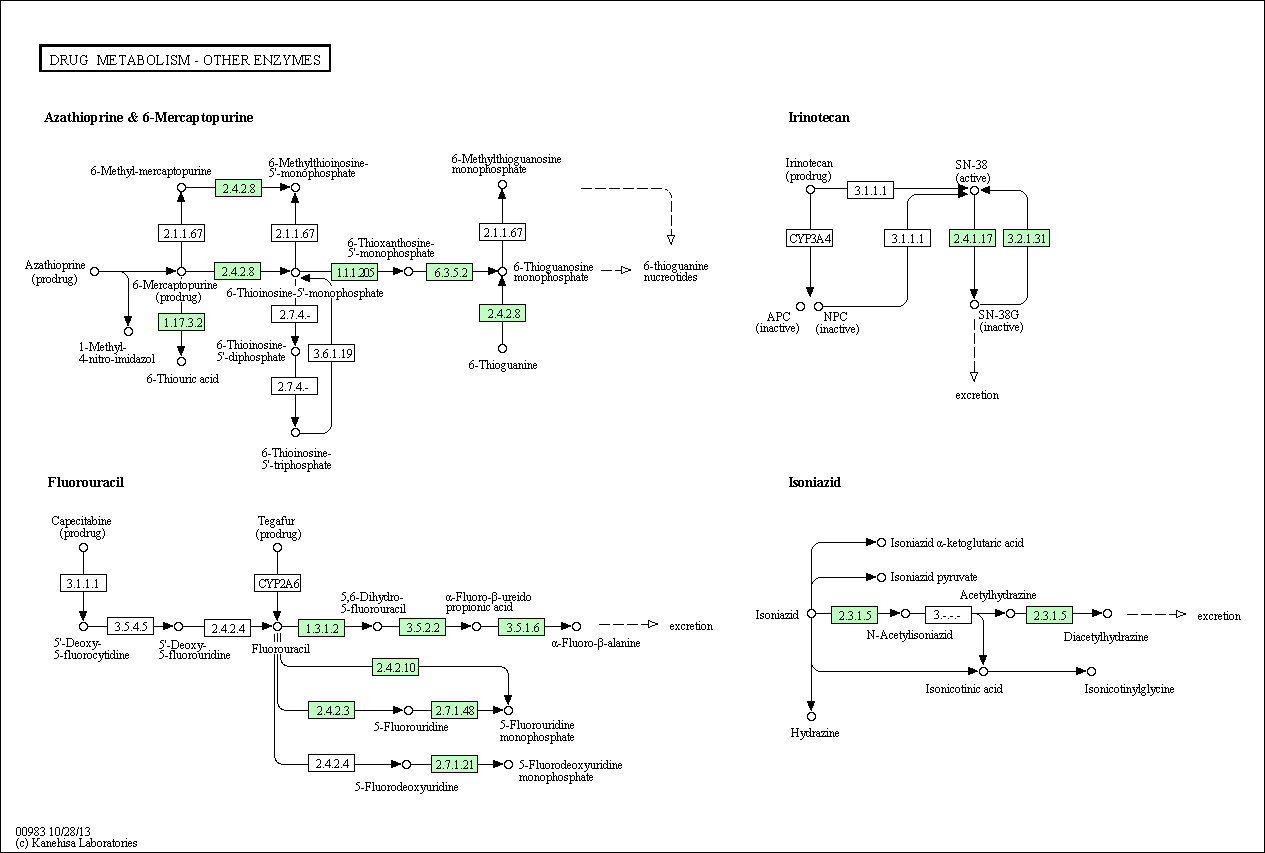

Supplement: Supplemental Information 9 [file peerj-04-1616-s009.gz › map/map00983.png]

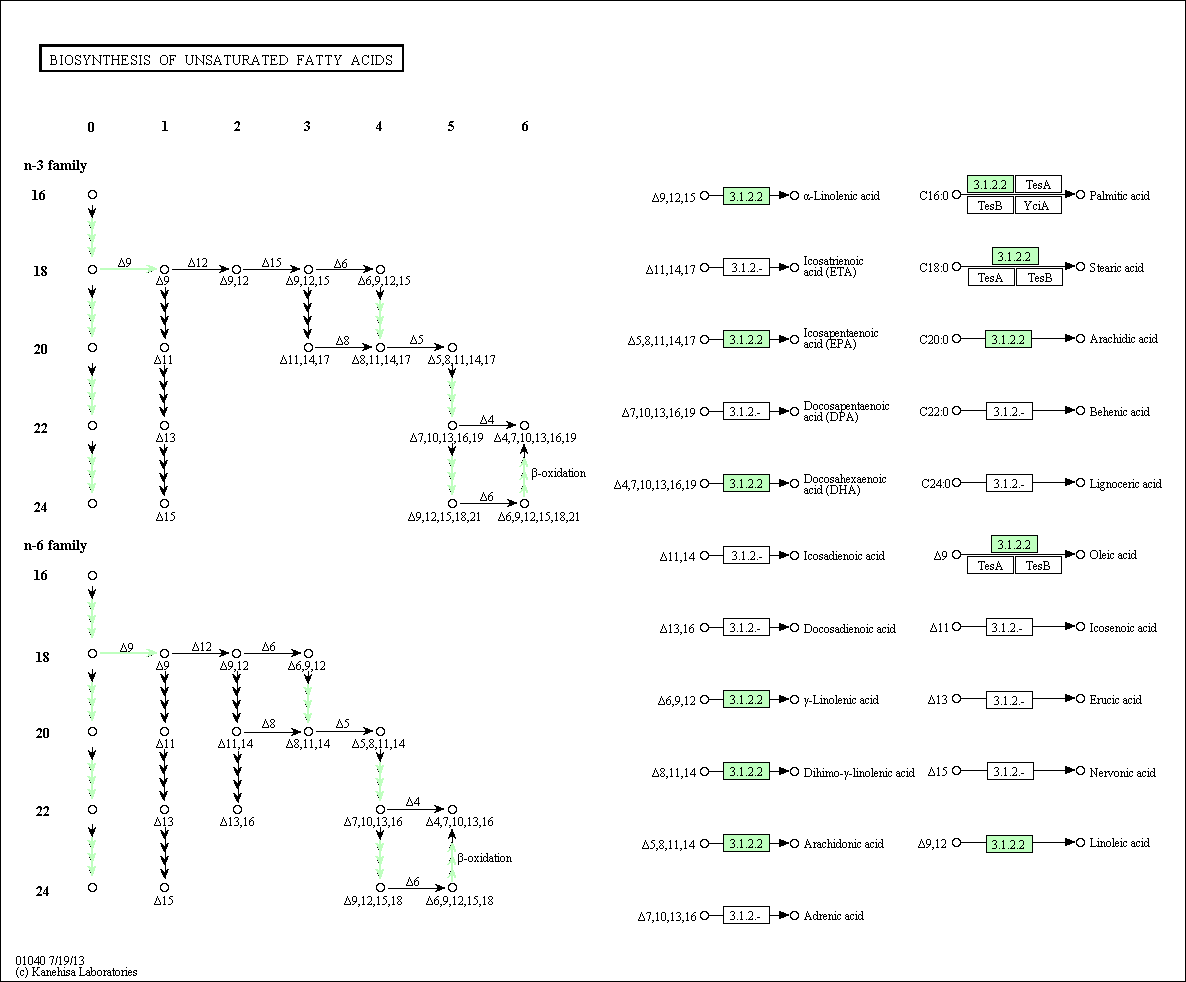

Supplement: Supplemental Information 9 [file peerj-04-1616-s009.gz › map/map01040.png]

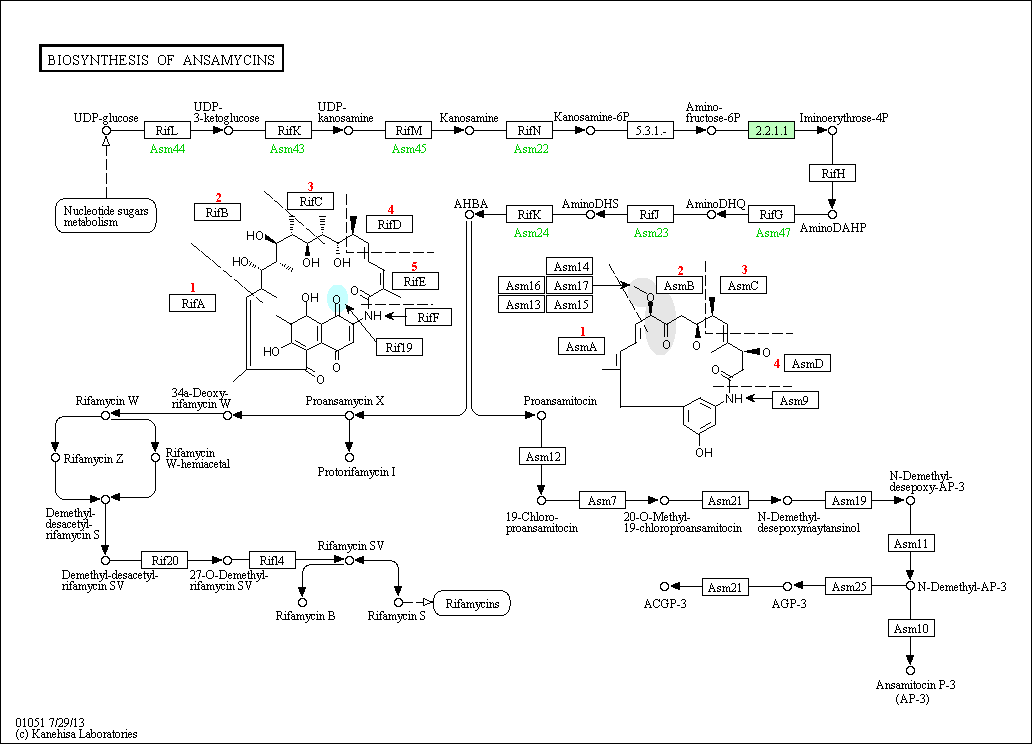

Supplement: Supplemental Information 9 [file peerj-04-1616-s009.gz › map/map01051.png]

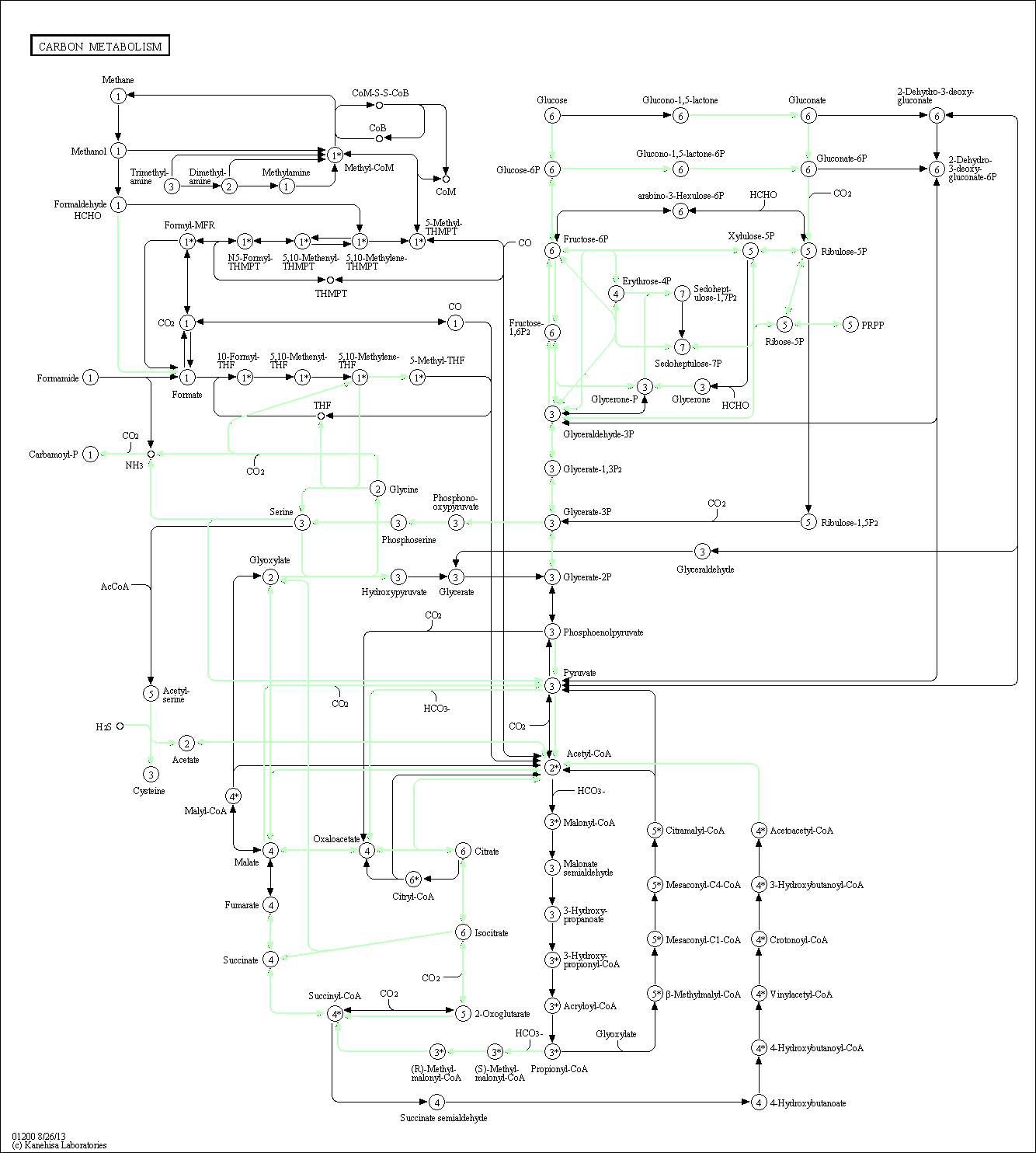

Supplement: Supplemental Information 9 [file peerj-04-1616-s009.gz › map/map01200.png]

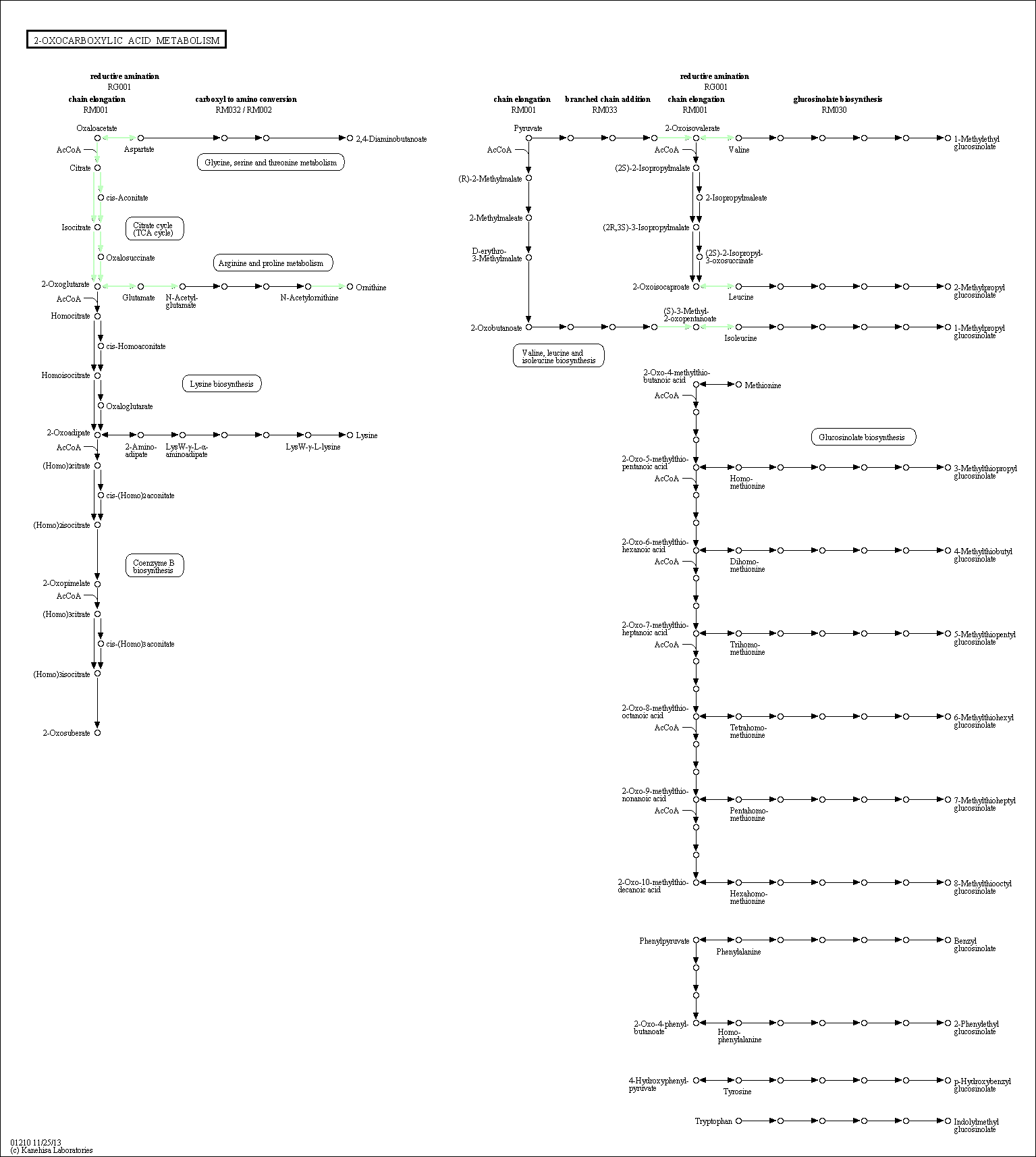

Supplement: Supplemental Information 9 [file peerj-04-1616-s009.gz › map/map01210.png]

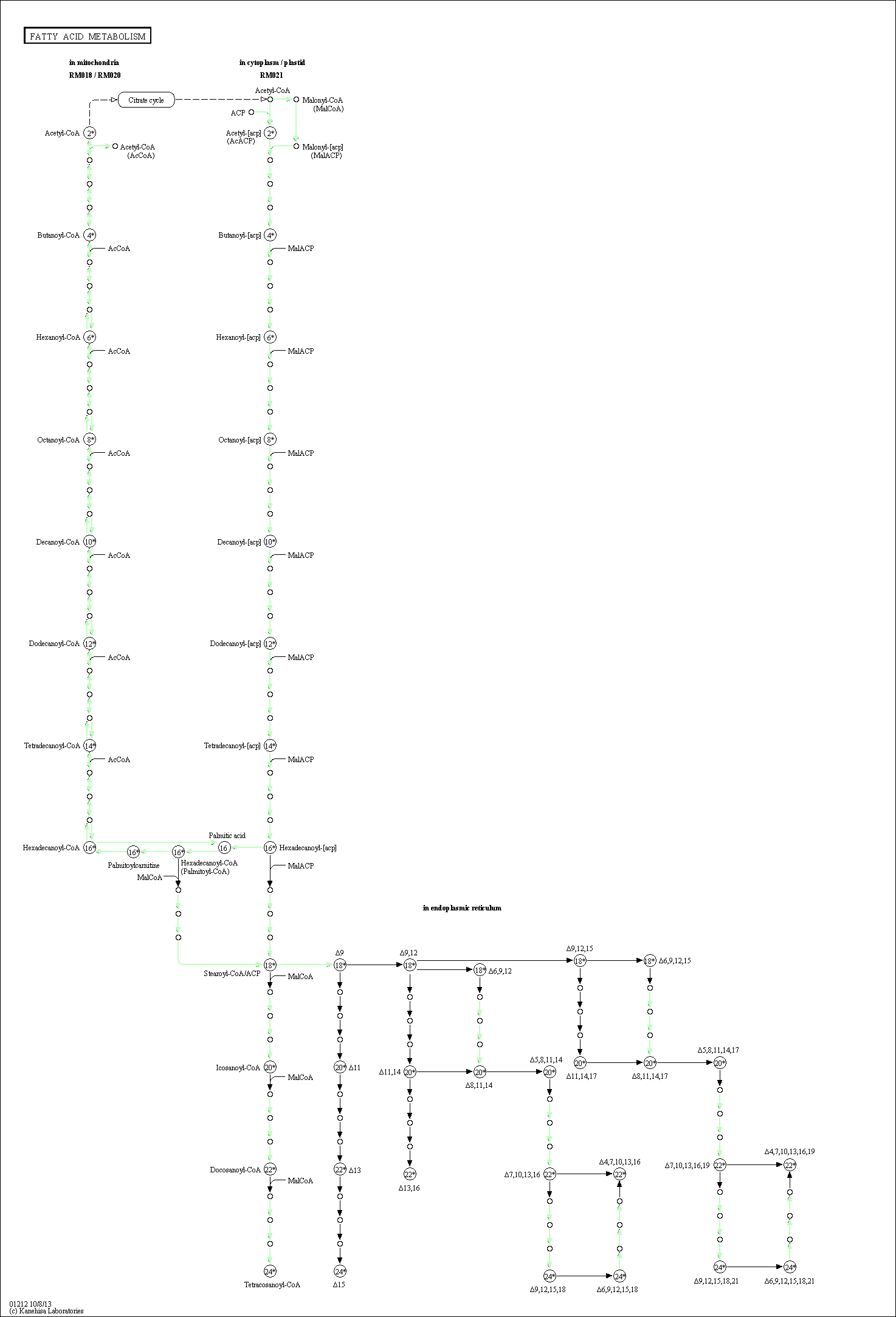

Supplement: Supplemental Information 9 [file peerj-04-1616-s009.gz › map/map01212.png]

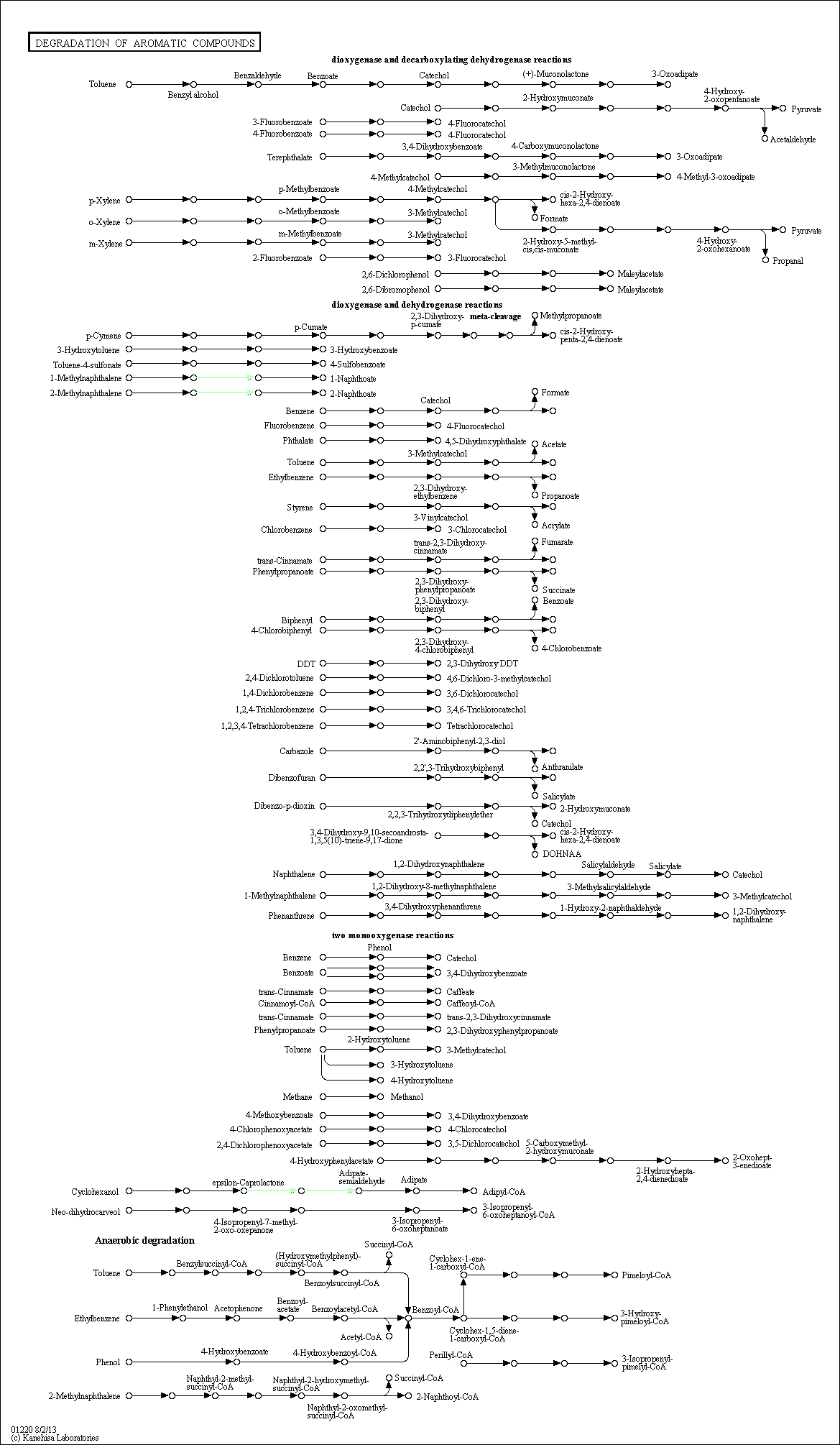

Supplement: Supplemental Information 9 [file peerj-04-1616-s009.gz › map/map01220.png]

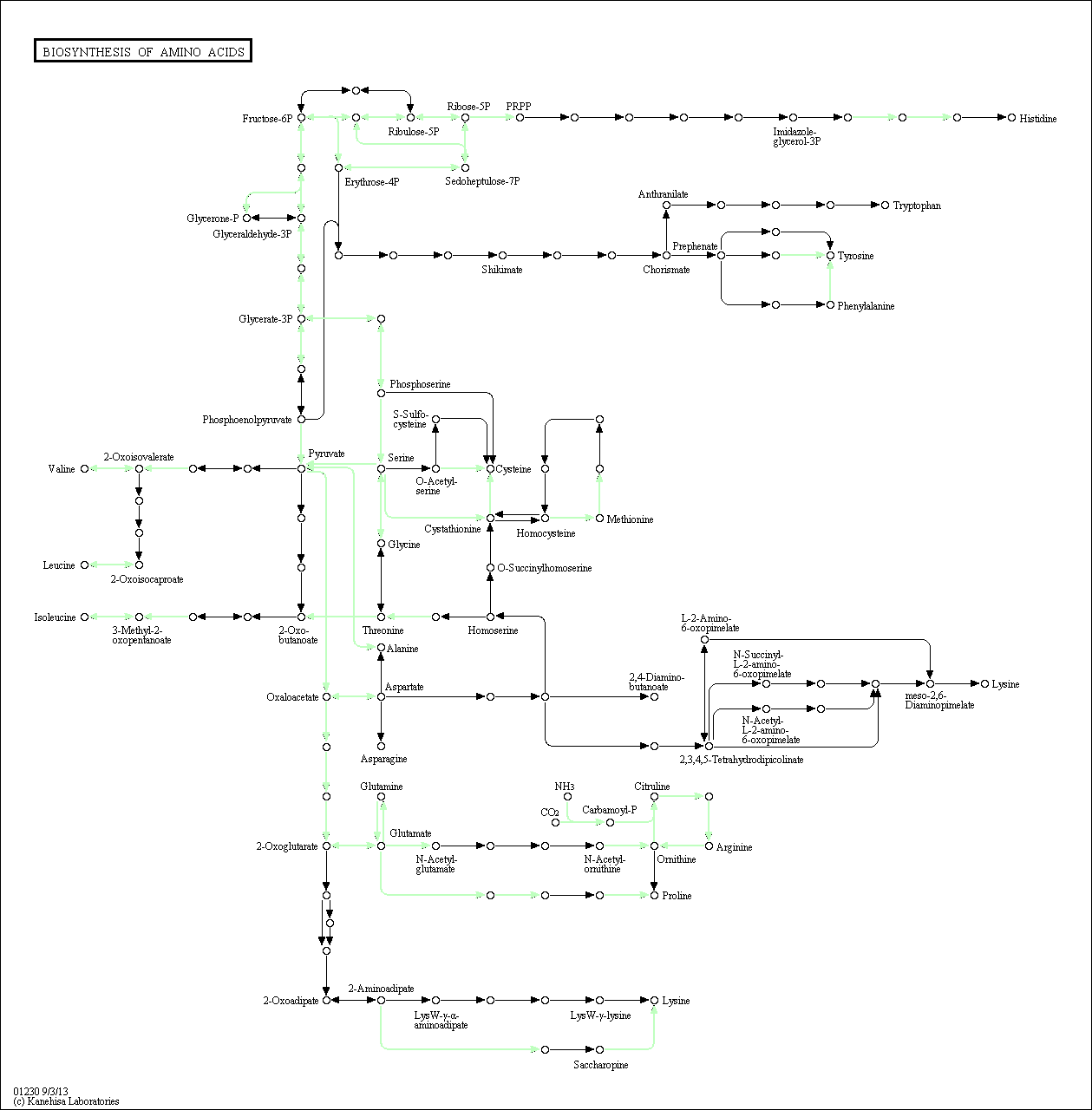

Supplement: Supplemental Information 9 [file peerj-04-1616-s009.gz › map/map01230.png]

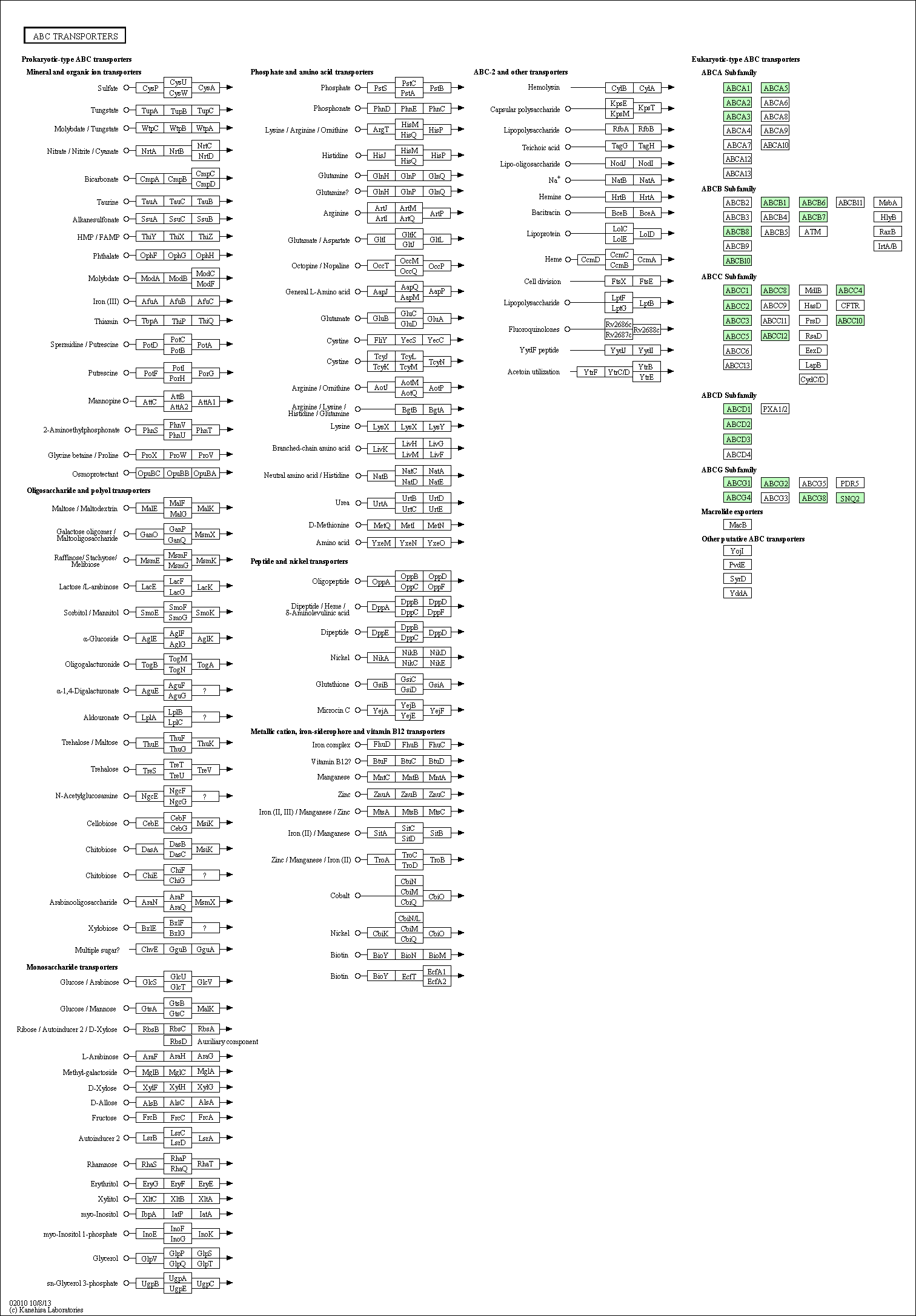

Supplement: Supplemental Information 9 [file peerj-04-1616-s009.gz › map/map02010.png]

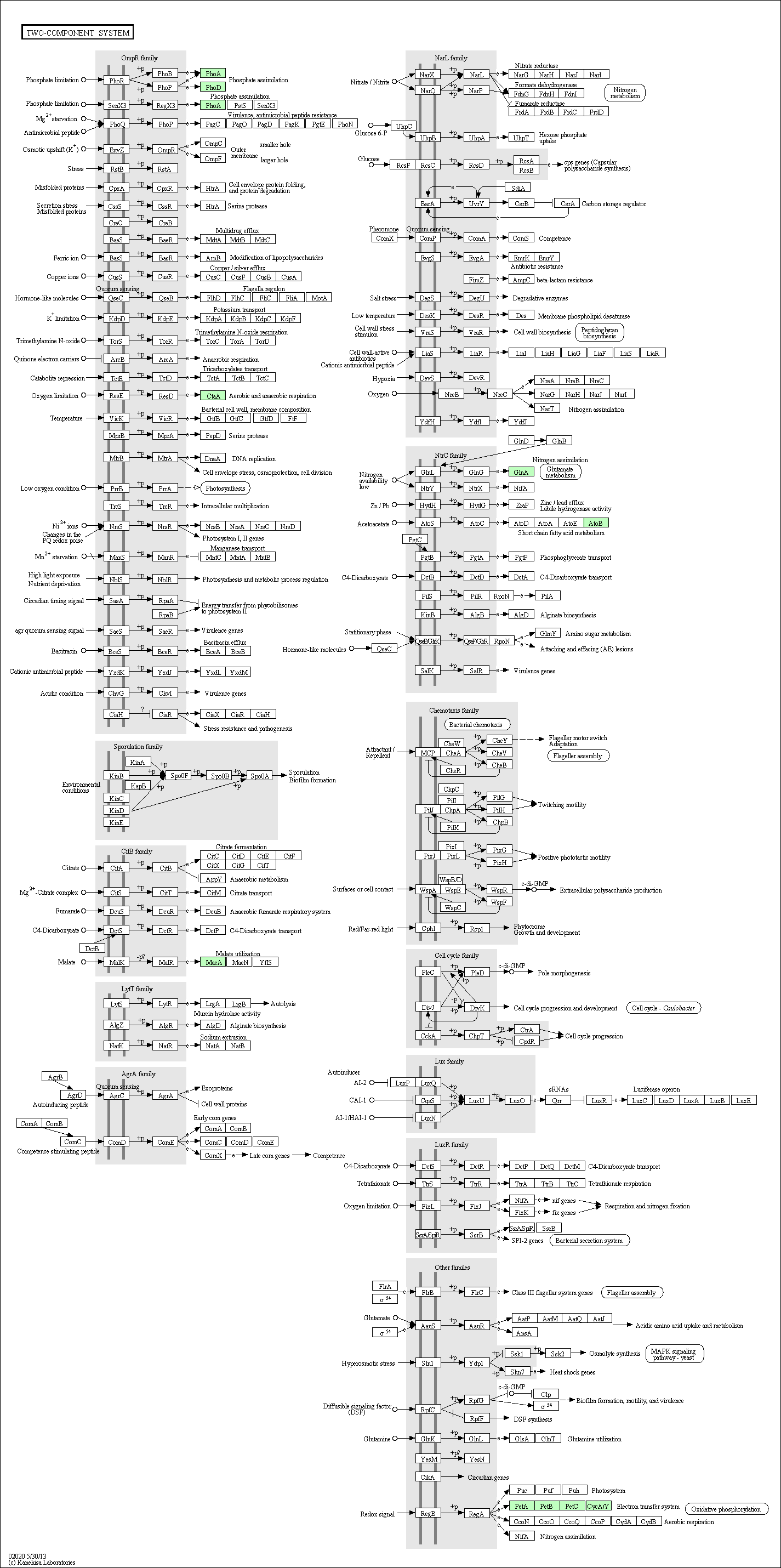

Supplement: Supplemental Information 9 [file peerj-04-1616-s009.gz › map/map02020.png]

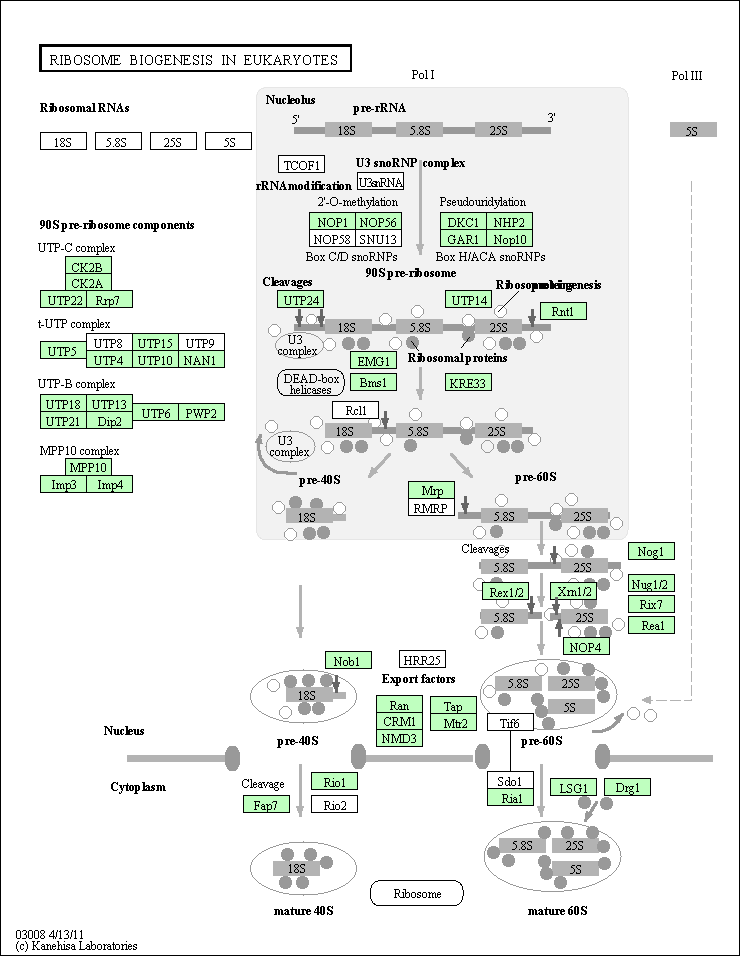

Supplement: Supplemental Information 9 [file peerj-04-1616-s009.gz › map/map03008.png]

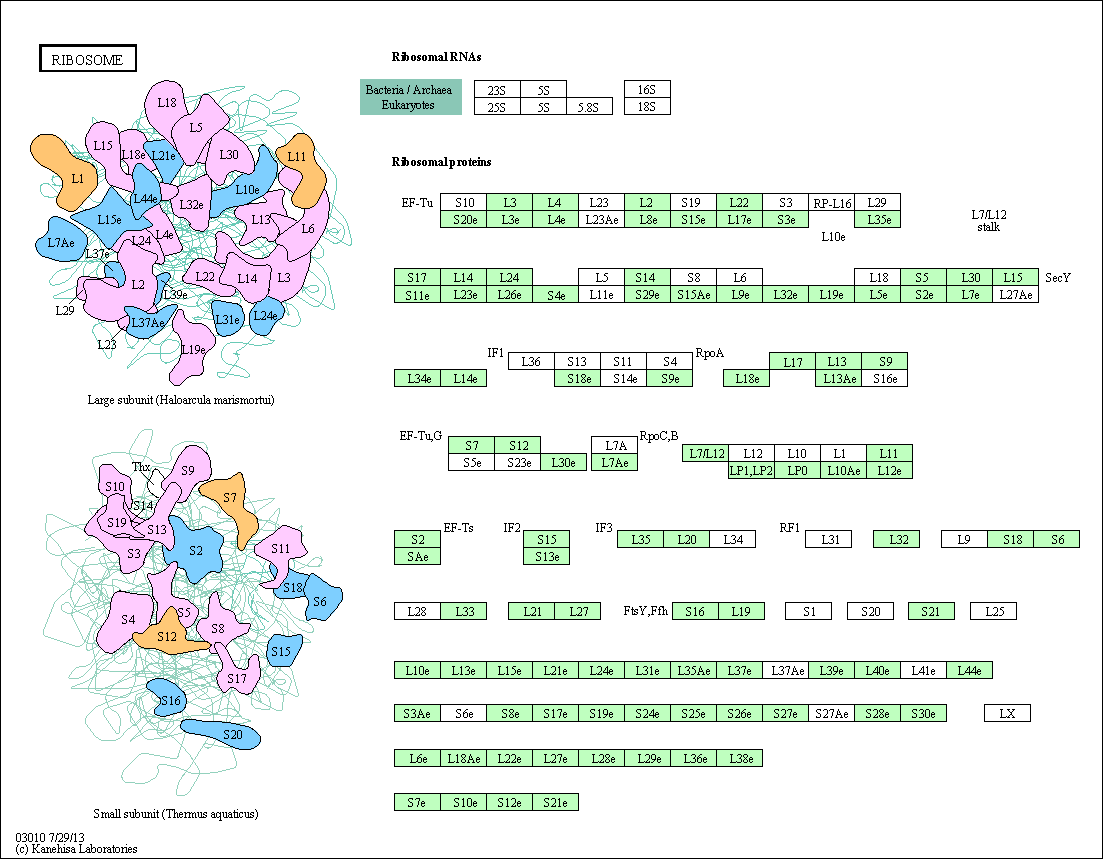

Supplement: Supplemental Information 9 [file peerj-04-1616-s009.gz › map/map03010.png]

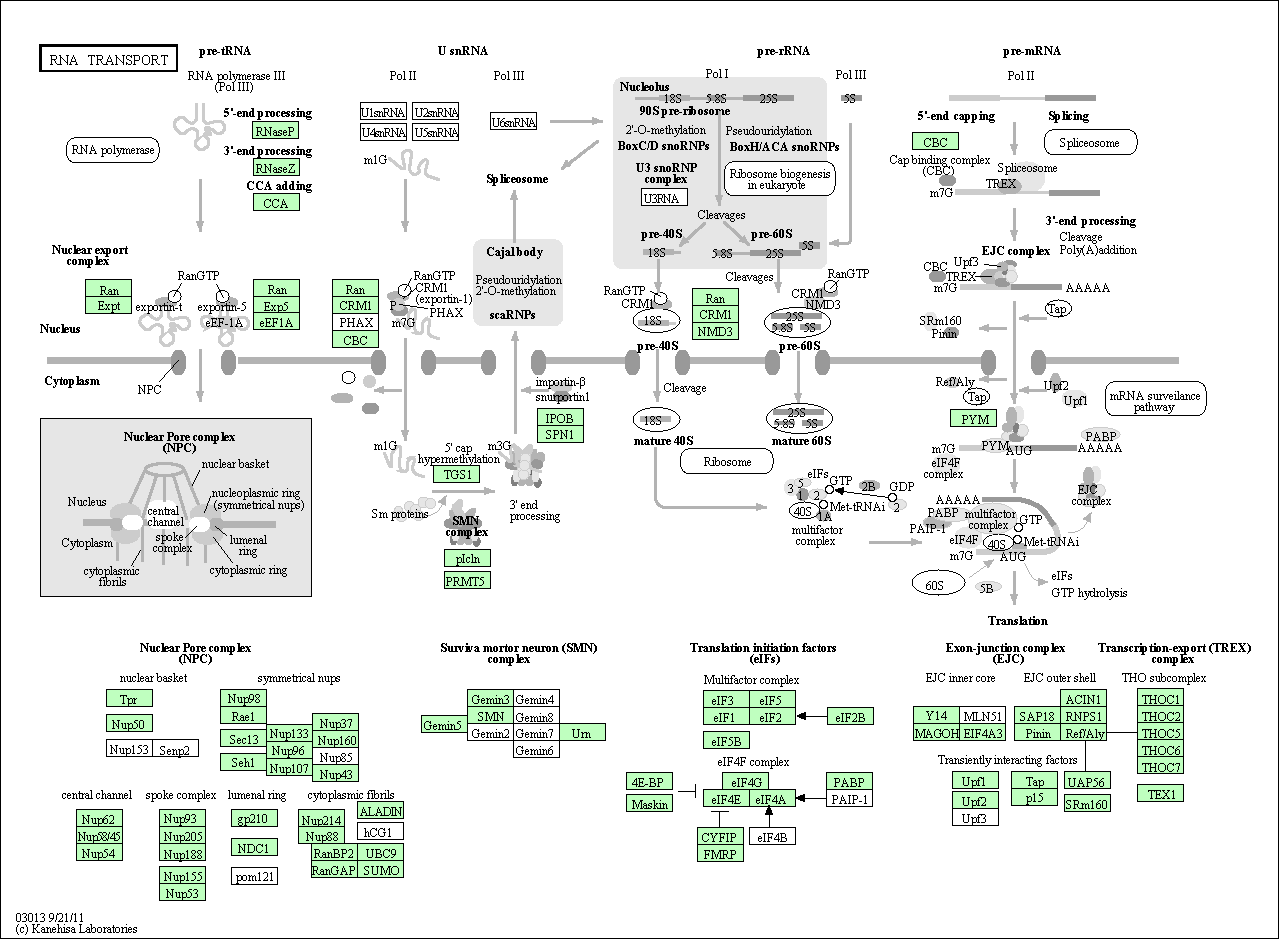

Supplement: Supplemental Information 9 [file peerj-04-1616-s009.gz › map/map03013.png]

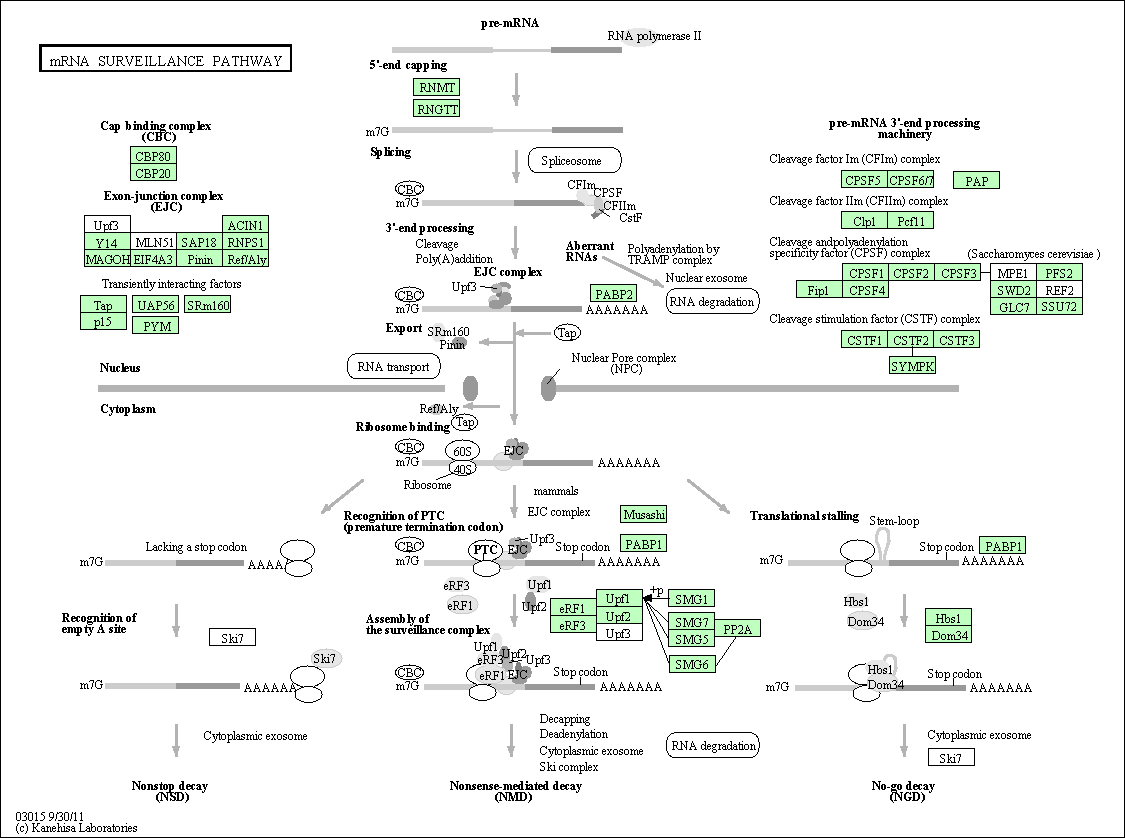

Supplement: Supplemental Information 9 [file peerj-04-1616-s009.gz › map/map03015.png]

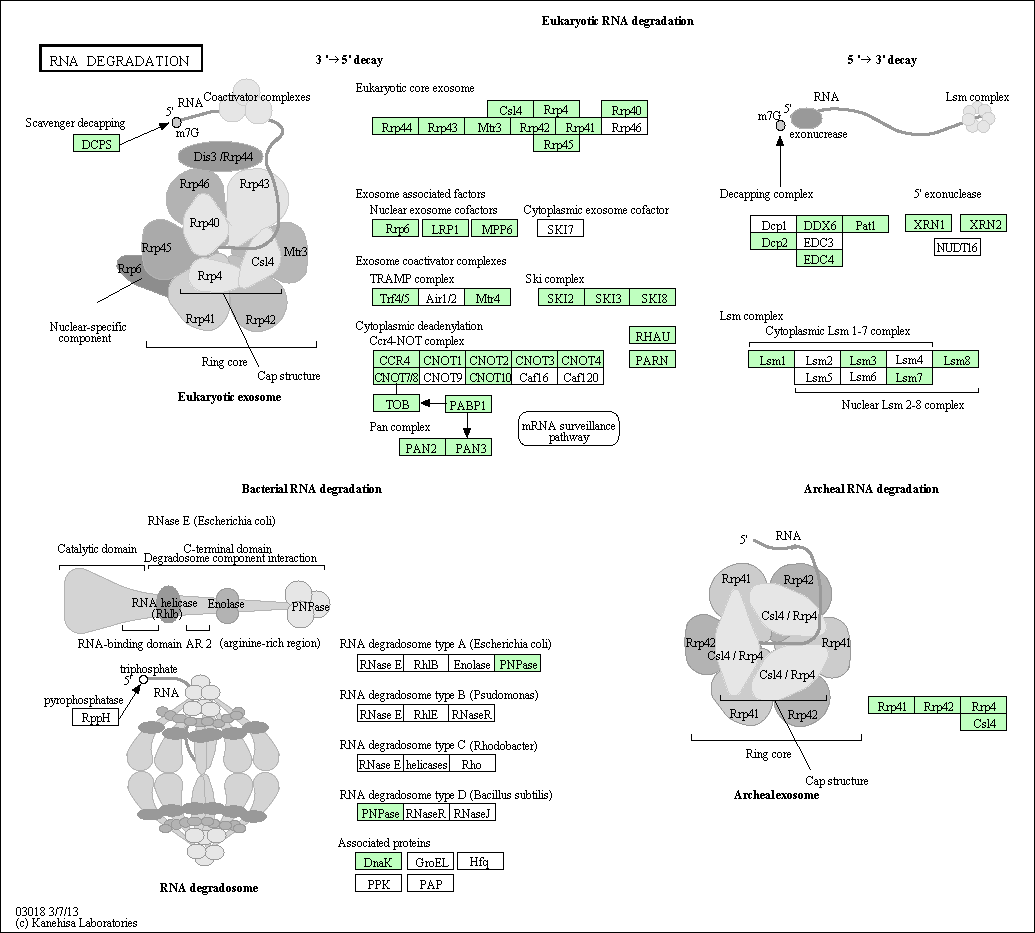

Supplement: Supplemental Information 9 [file peerj-04-1616-s009.gz › map/map03018.png]

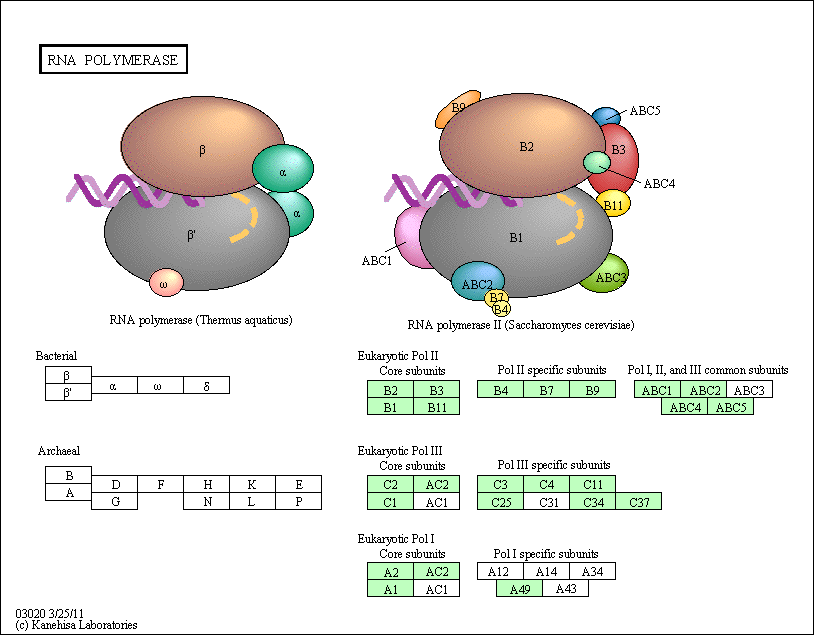

Supplement: Supplemental Information 9 [file peerj-04-1616-s009.gz › map/map03020.png]

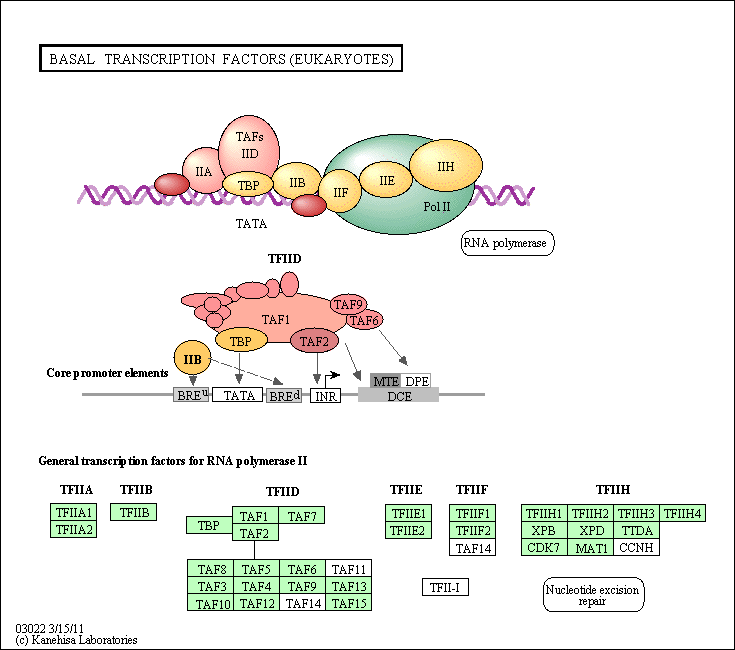

Supplement: Supplemental Information 9 [file peerj-04-1616-s009.gz › map/map03022.png]

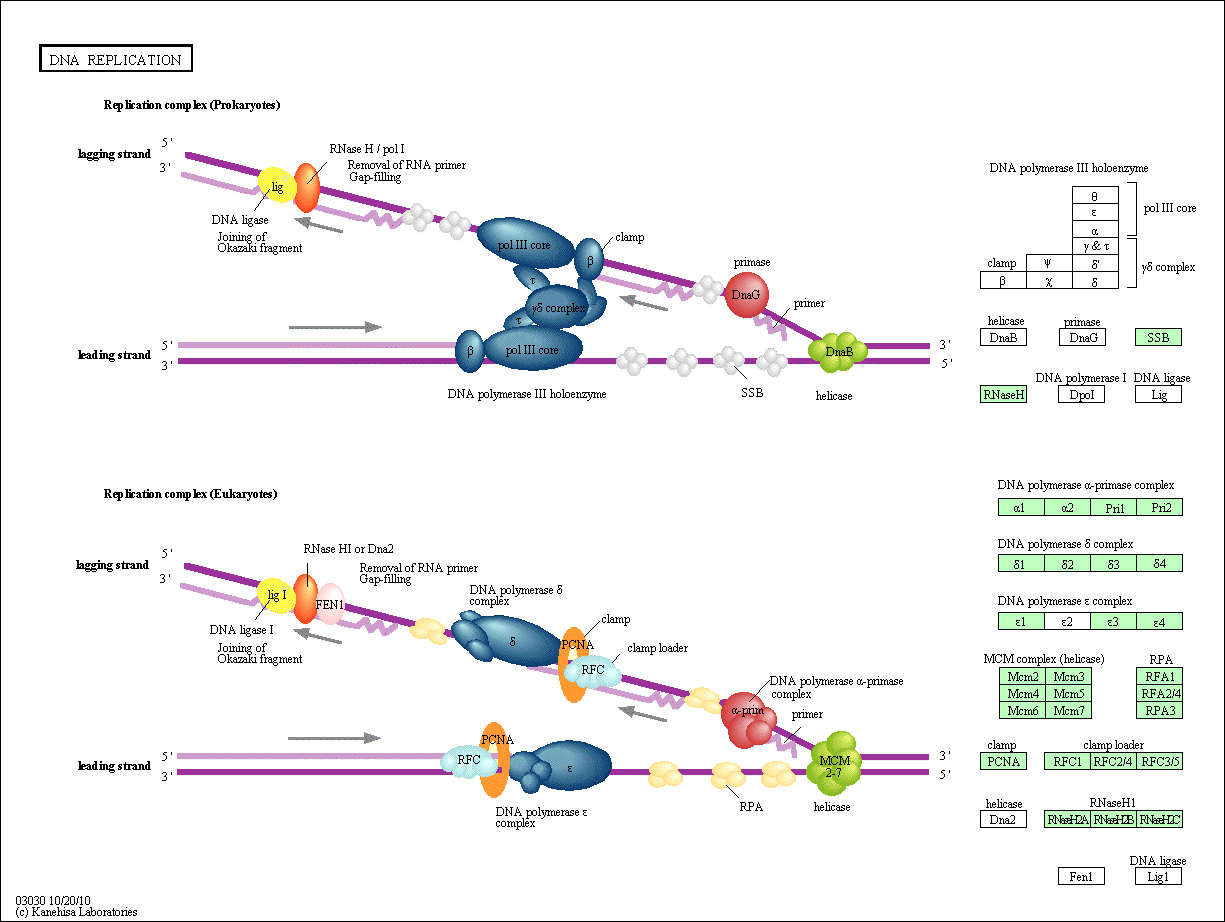

Supplement: Supplemental Information 9 [file peerj-04-1616-s009.gz › map/map03030.png]

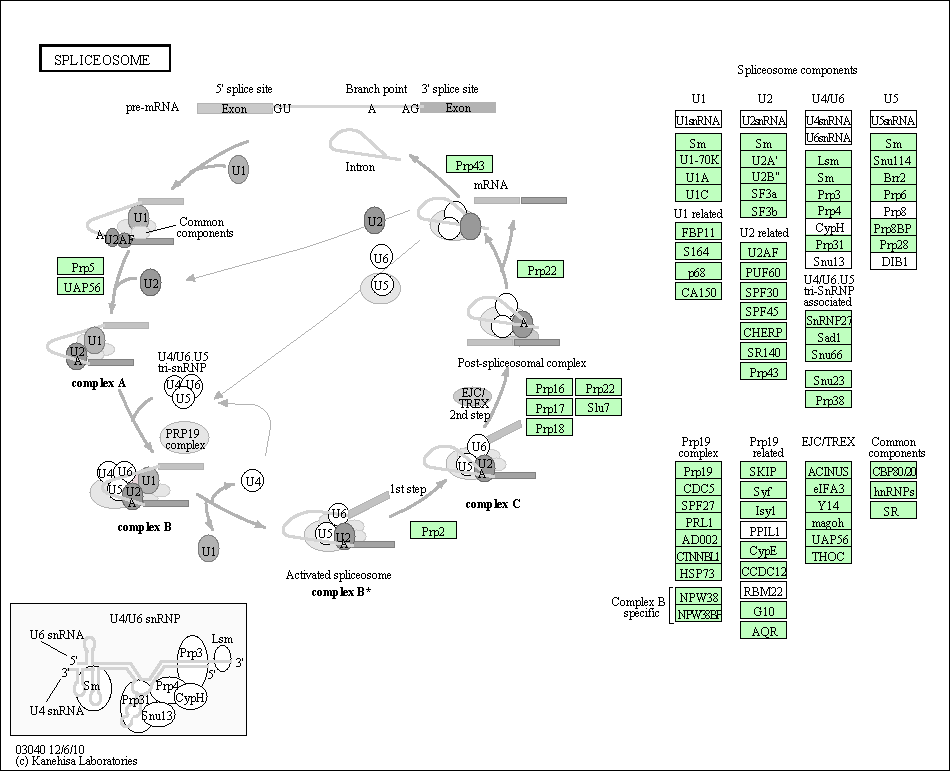

Supplement: Supplemental Information 9 [file peerj-04-1616-s009.gz › map/map03040.png]

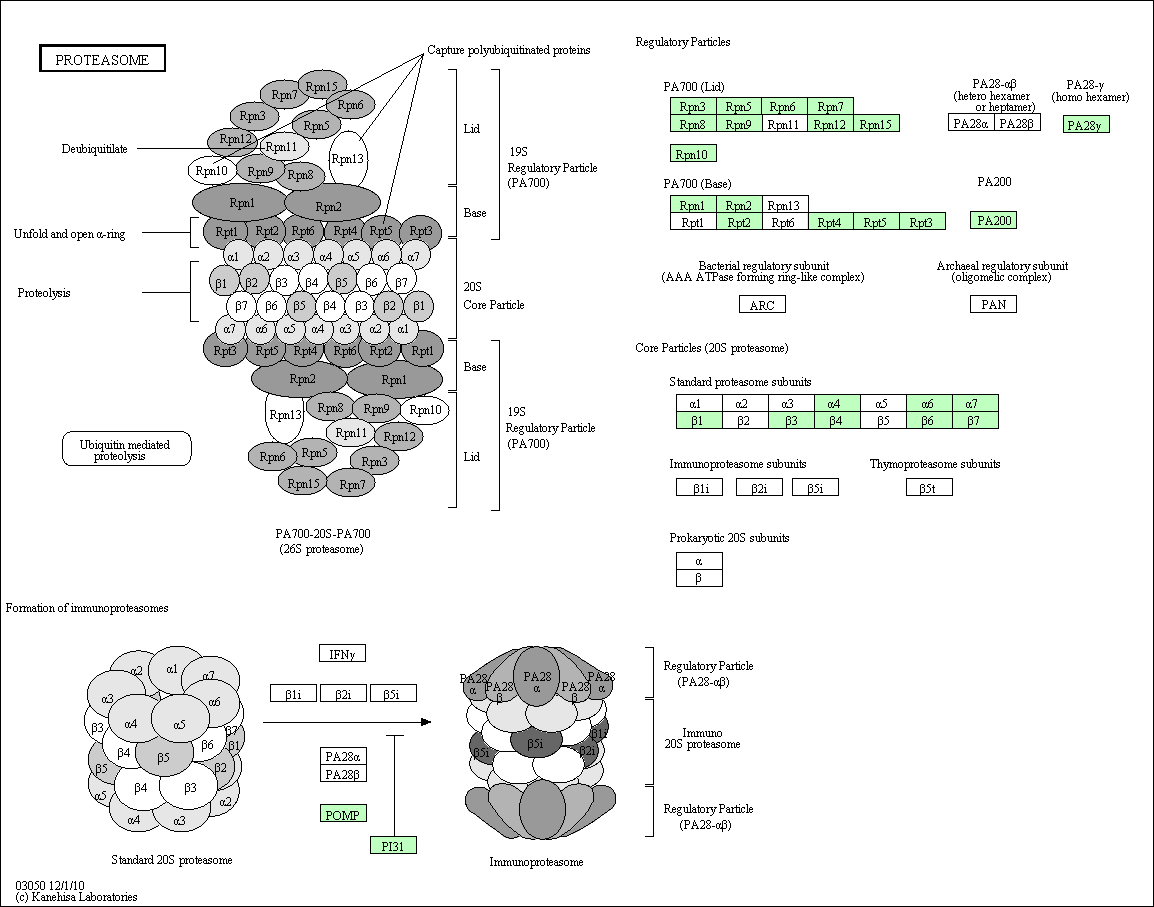

Supplement: Supplemental Information 9 [file peerj-04-1616-s009.gz › map/map03050.png]

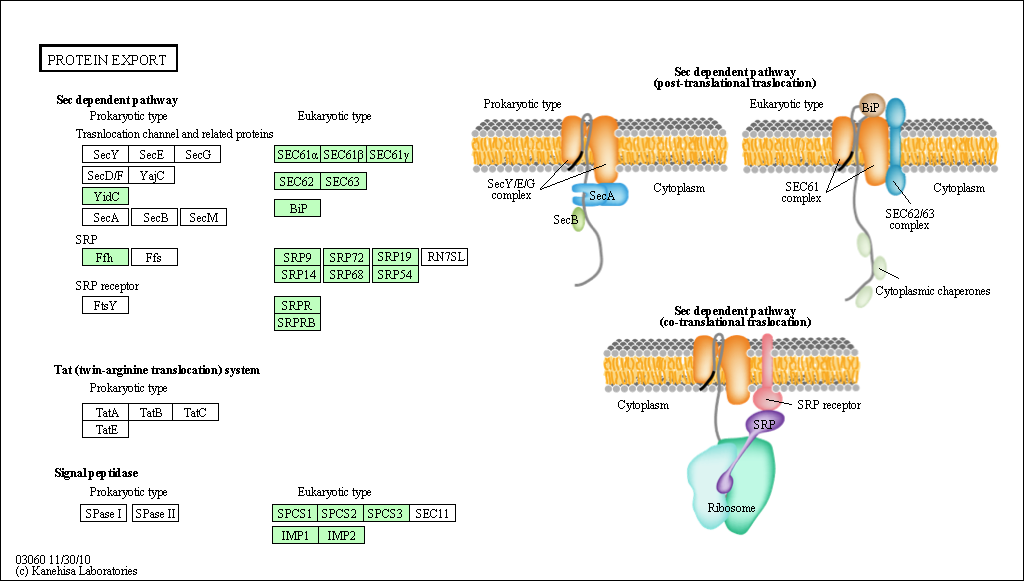

Supplement: Supplemental Information 9 [file peerj-04-1616-s009.gz › map/map03060.png]

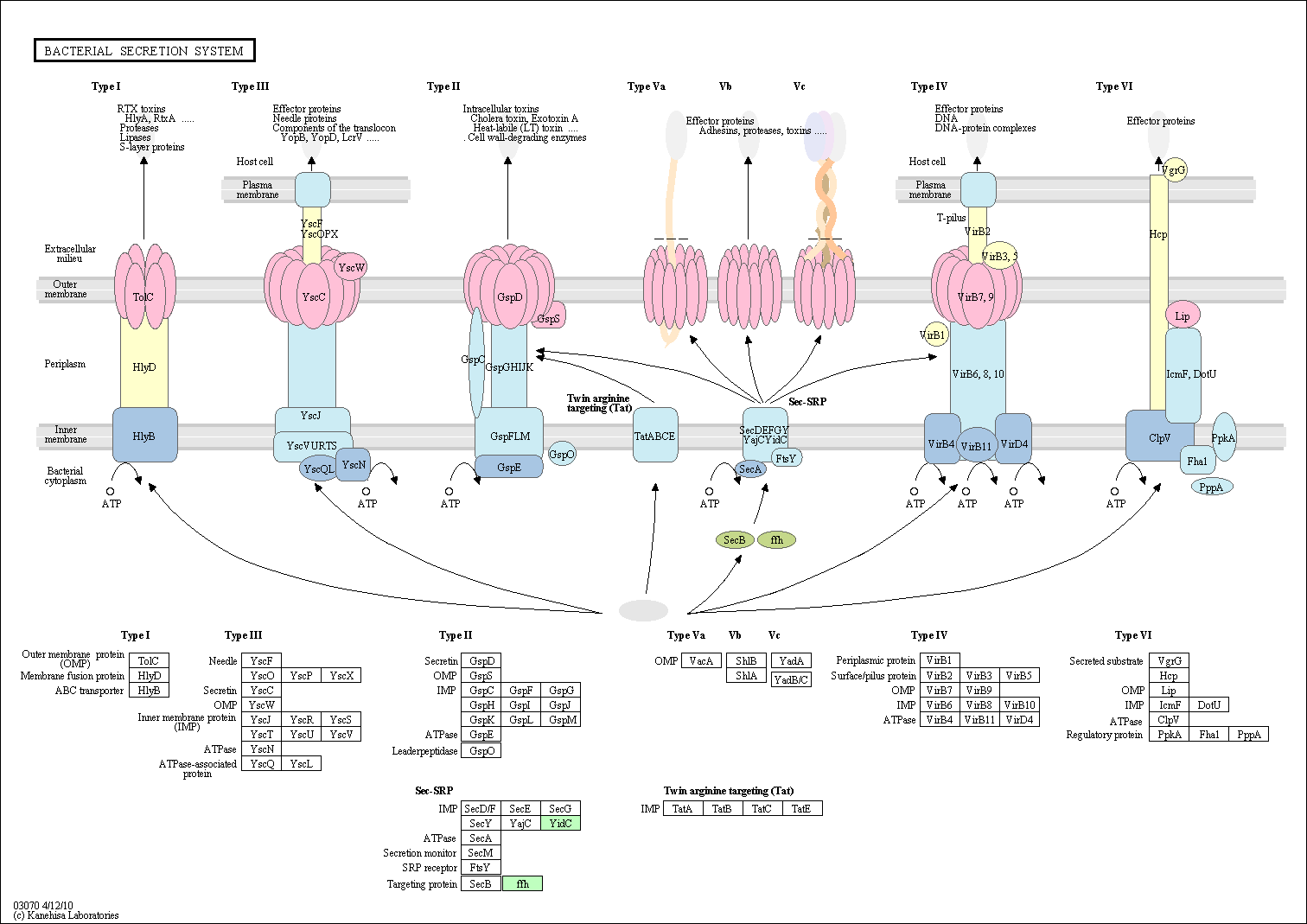

Supplement: Supplemental Information 9 [file peerj-04-1616-s009.gz › map/map03070.png]

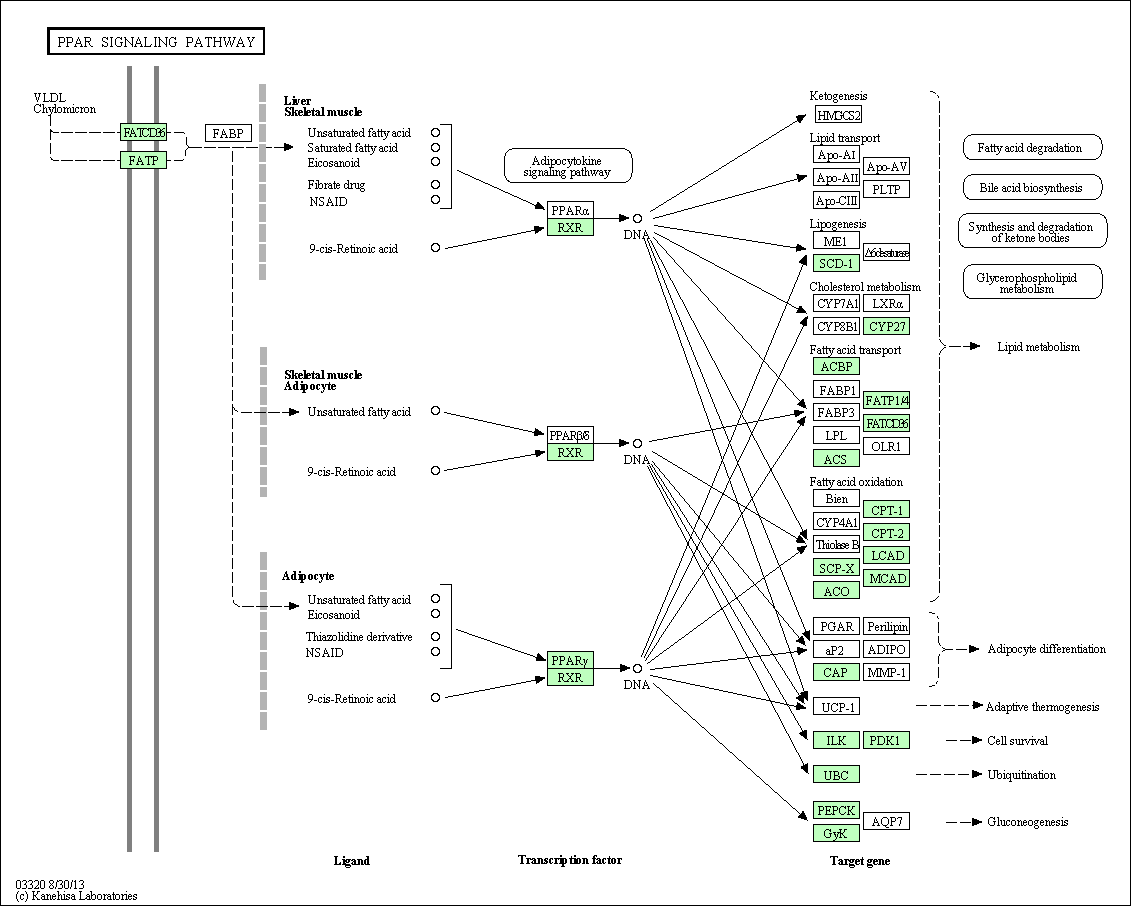

Supplement: Supplemental Information 9 [file peerj-04-1616-s009.gz › map/map03320.png]

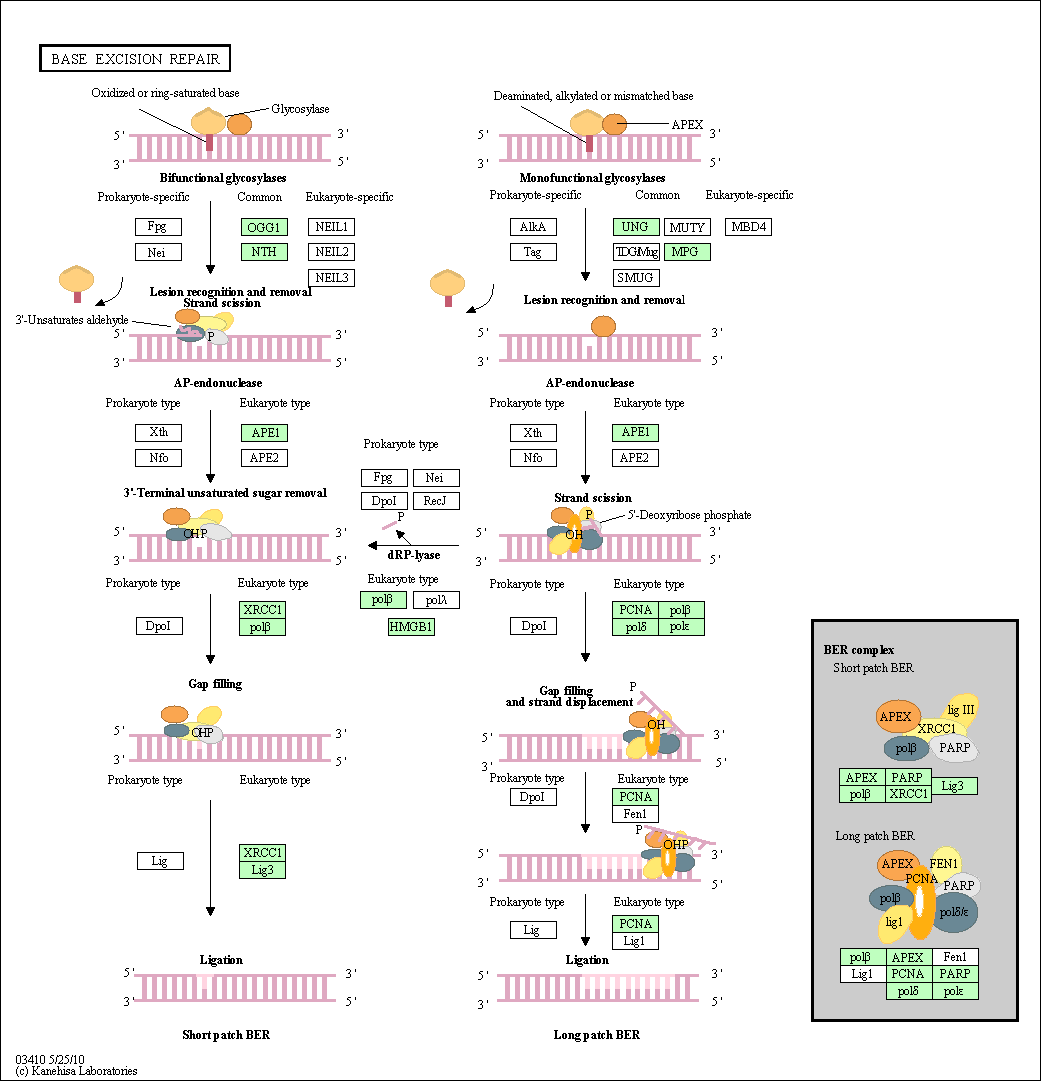

Supplement: Supplemental Information 9 [file peerj-04-1616-s009.gz › map/map03410.png]

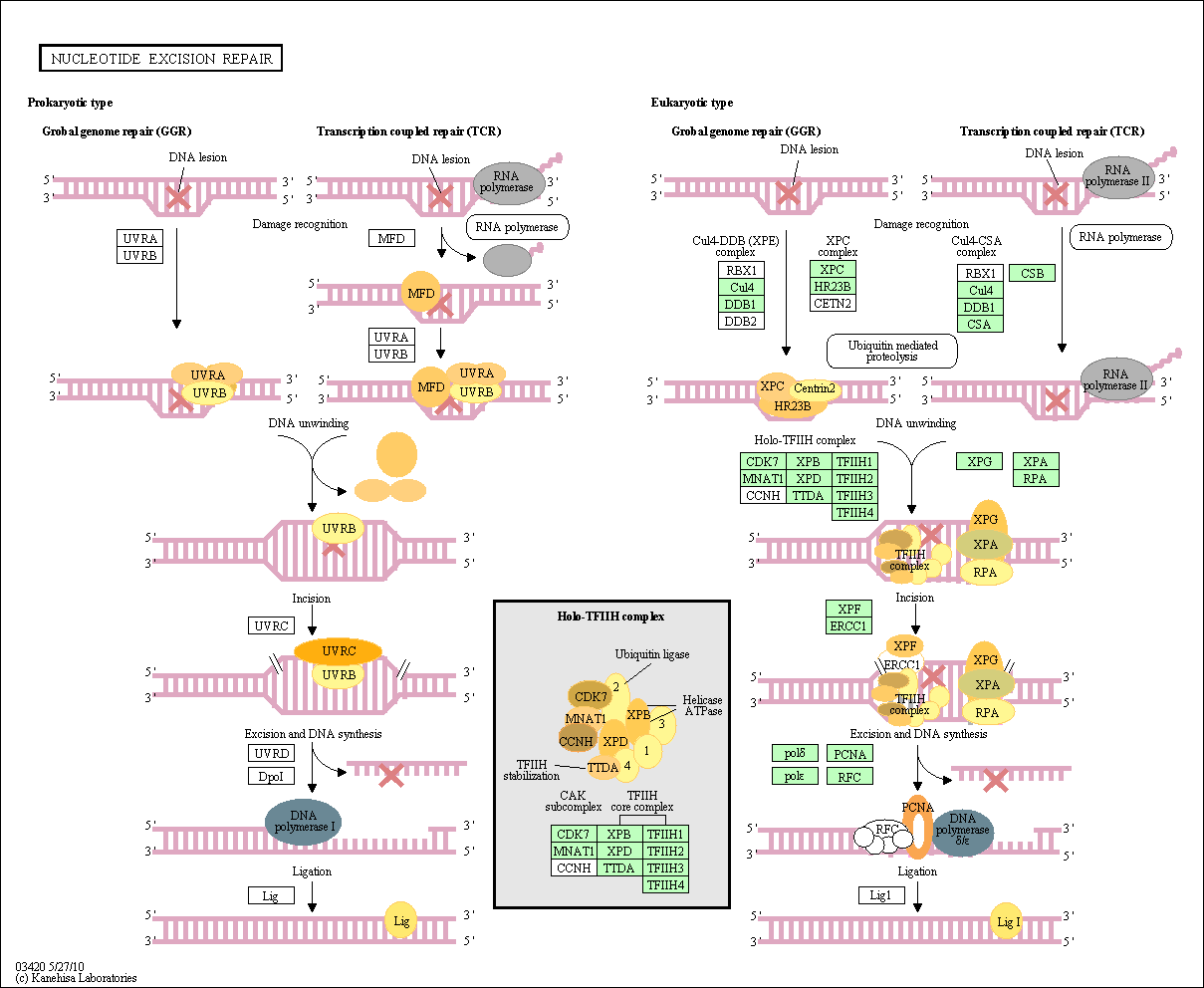

Supplement: Supplemental Information 9 [file peerj-04-1616-s009.gz › map/map03420.png]

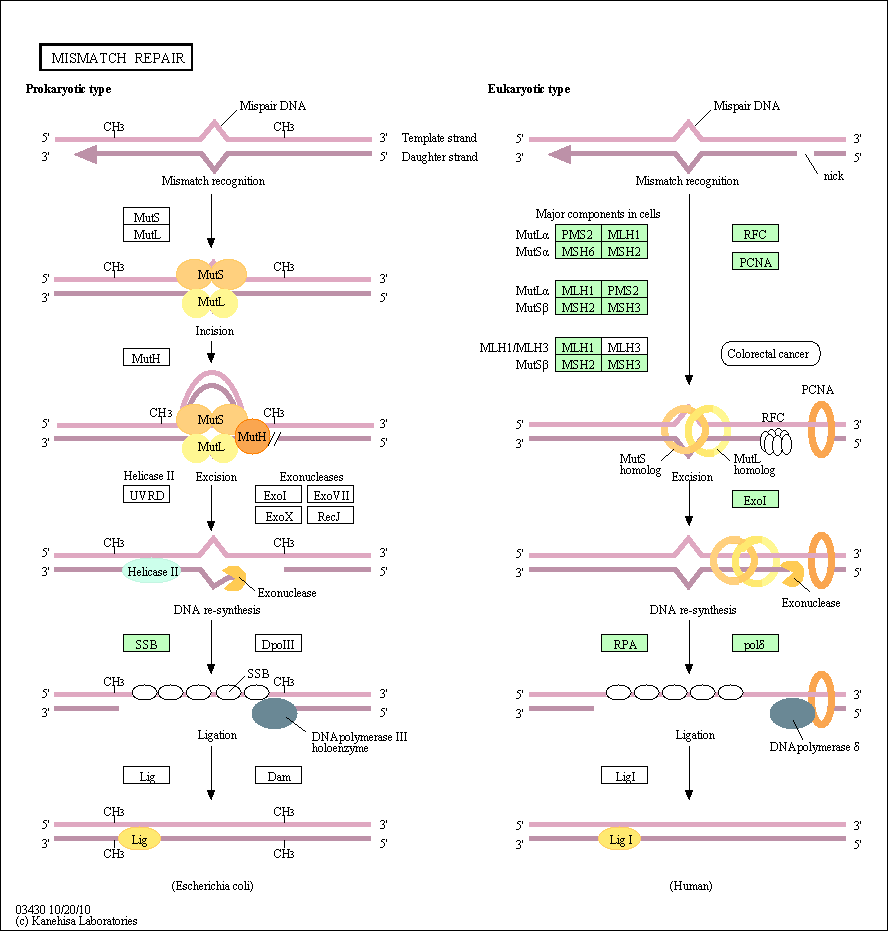

Supplement: Supplemental Information 9 [file peerj-04-1616-s009.gz › map/map03430.png]

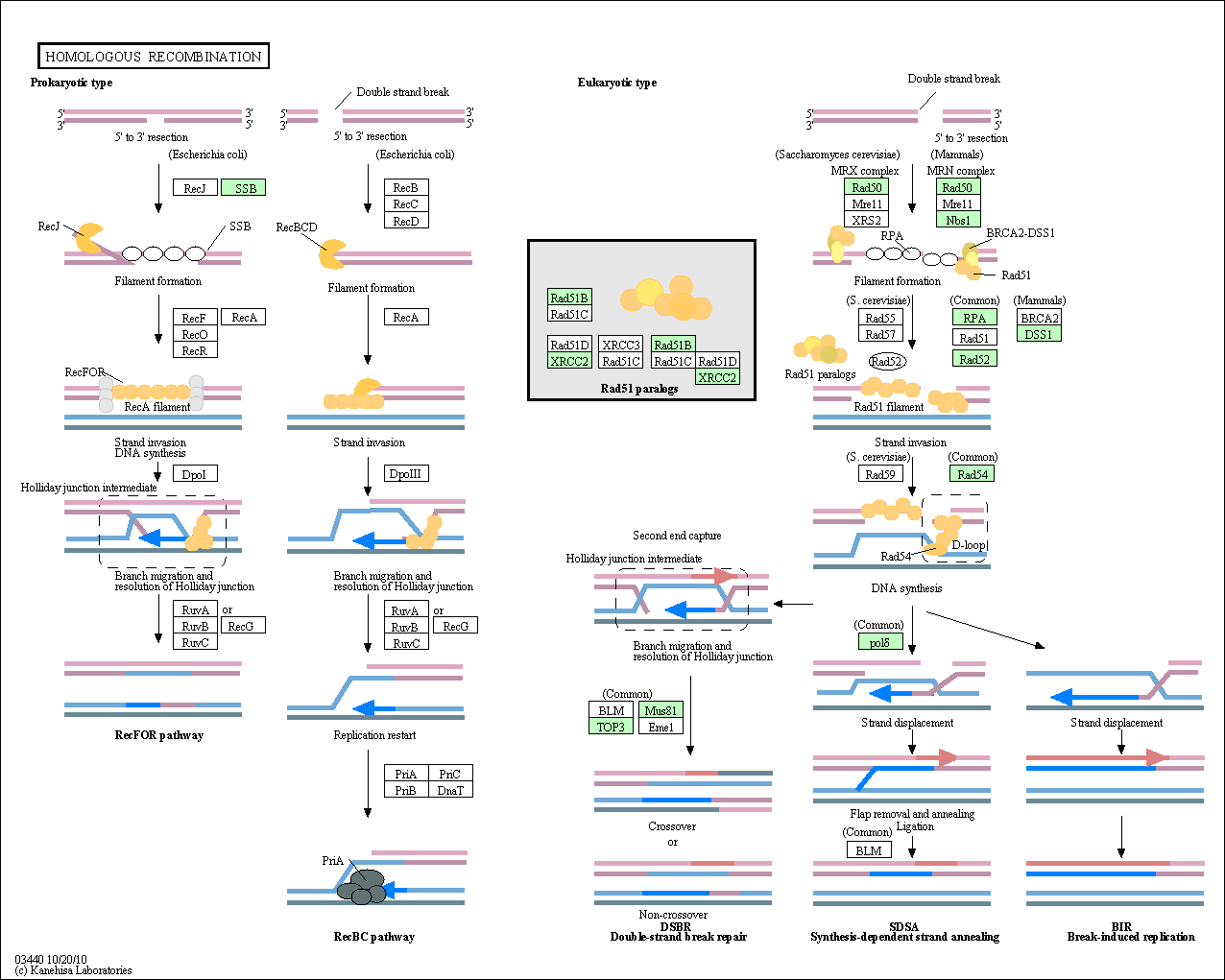

Supplement: Supplemental Information 9 [file peerj-04-1616-s009.gz › map/map03440.png]

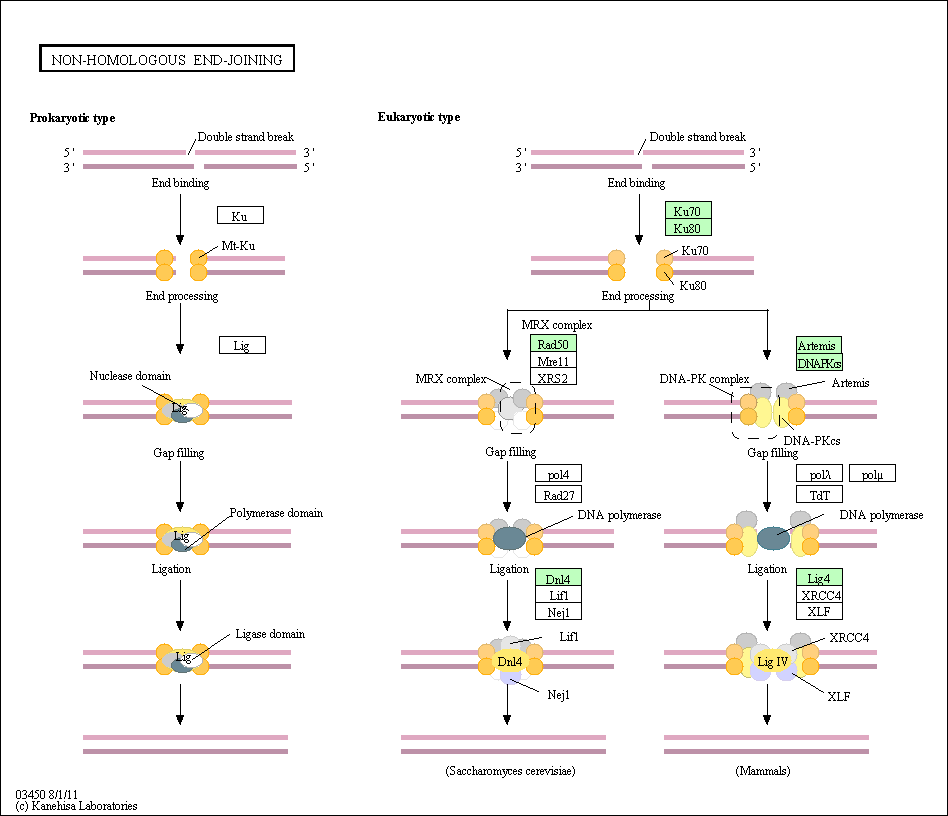

Supplement: Supplemental Information 9 [file peerj-04-1616-s009.gz › map/map03450.png]

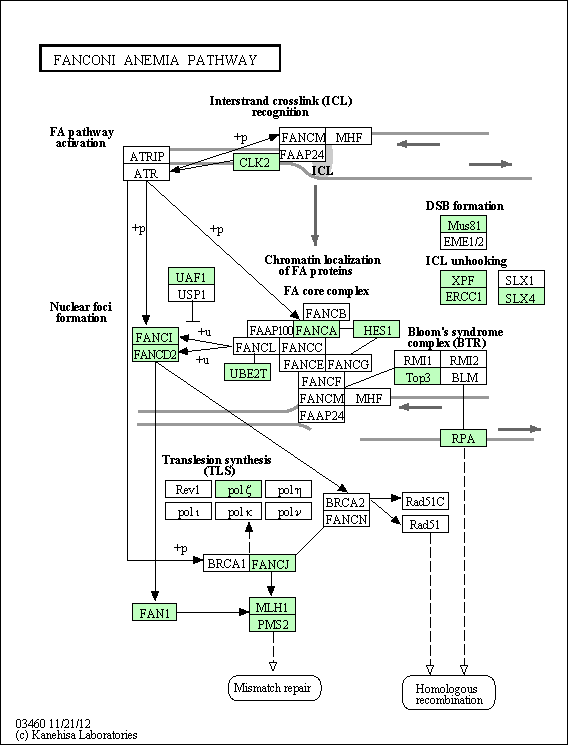

Supplement: Supplemental Information 9 [file peerj-04-1616-s009.gz › map/map03460.png]

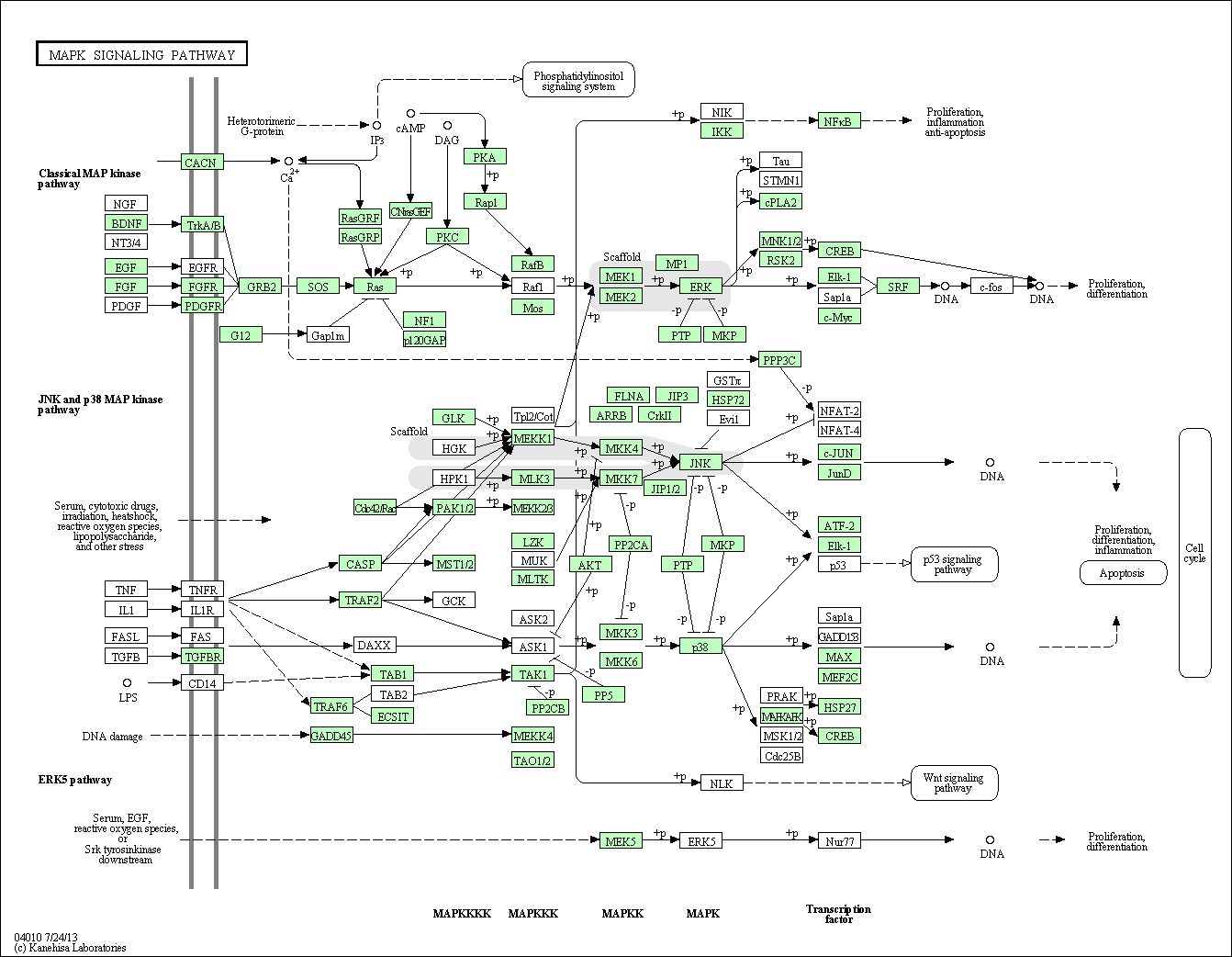

Supplement: Supplemental Information 9 [file peerj-04-1616-s009.gz › map/map04010.png]

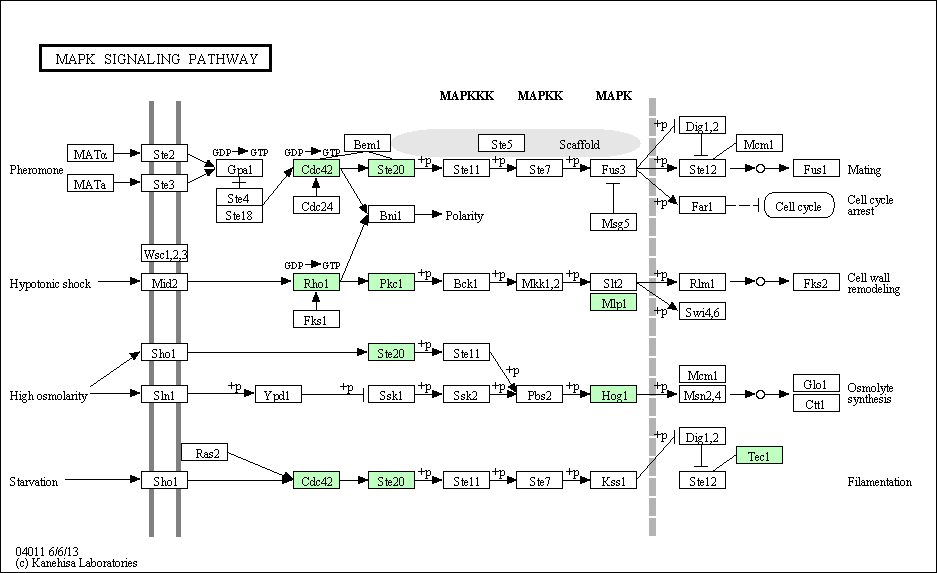

Supplement: Supplemental Information 9 [file peerj-04-1616-s009.gz › map/map04011.png]

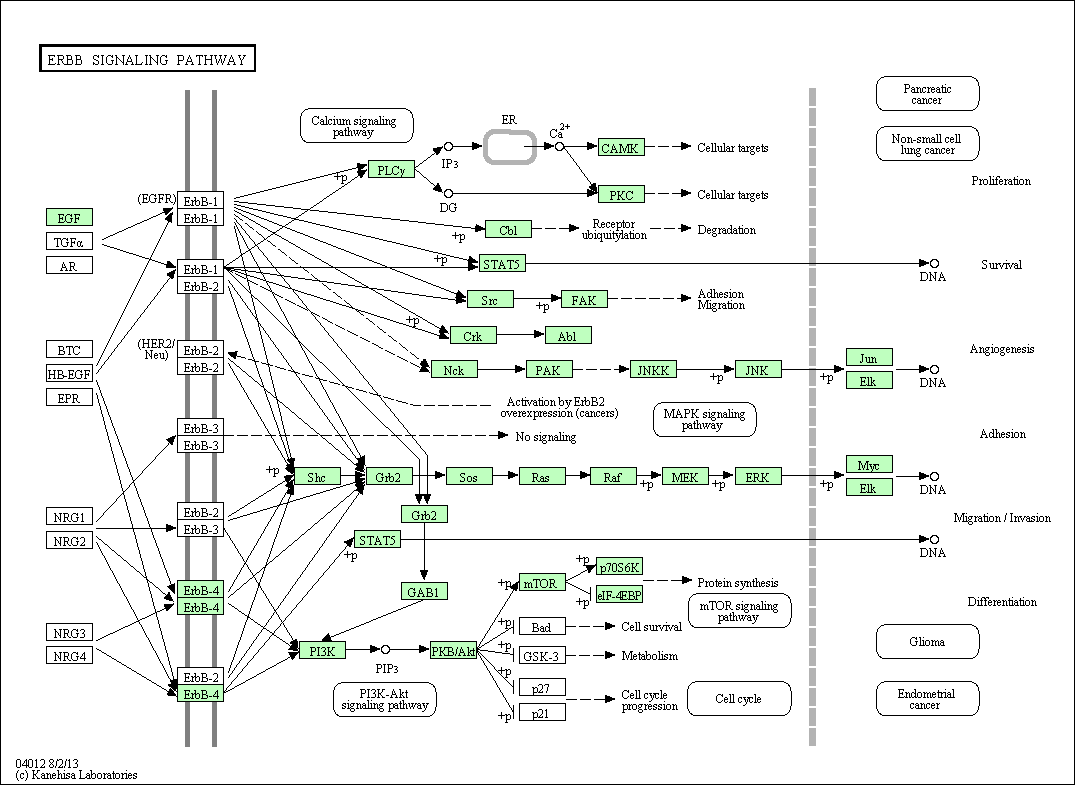

Supplement: Supplemental Information 9 [file peerj-04-1616-s009.gz › map/map04012.png]

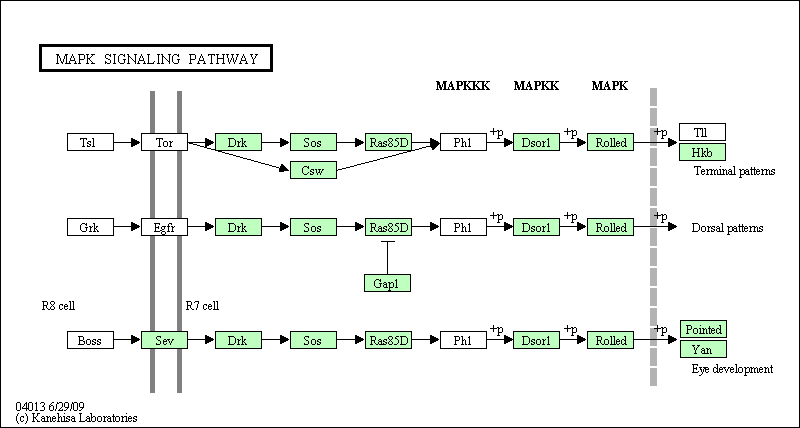

Supplement: Supplemental Information 9 [file peerj-04-1616-s009.gz › map/map04013.png]

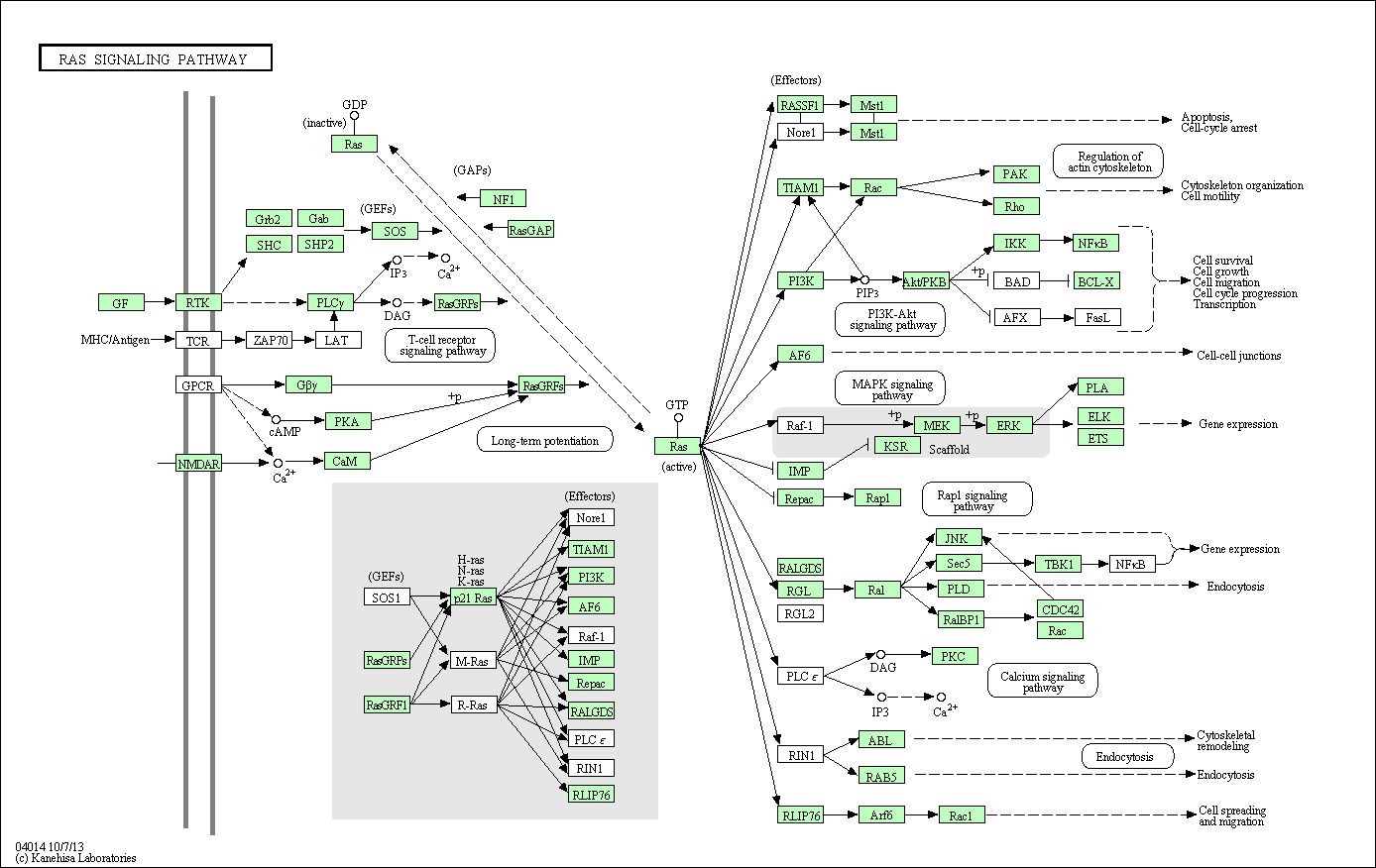

Supplement: Supplemental Information 9 [file peerj-04-1616-s009.gz › map/map04014.png]

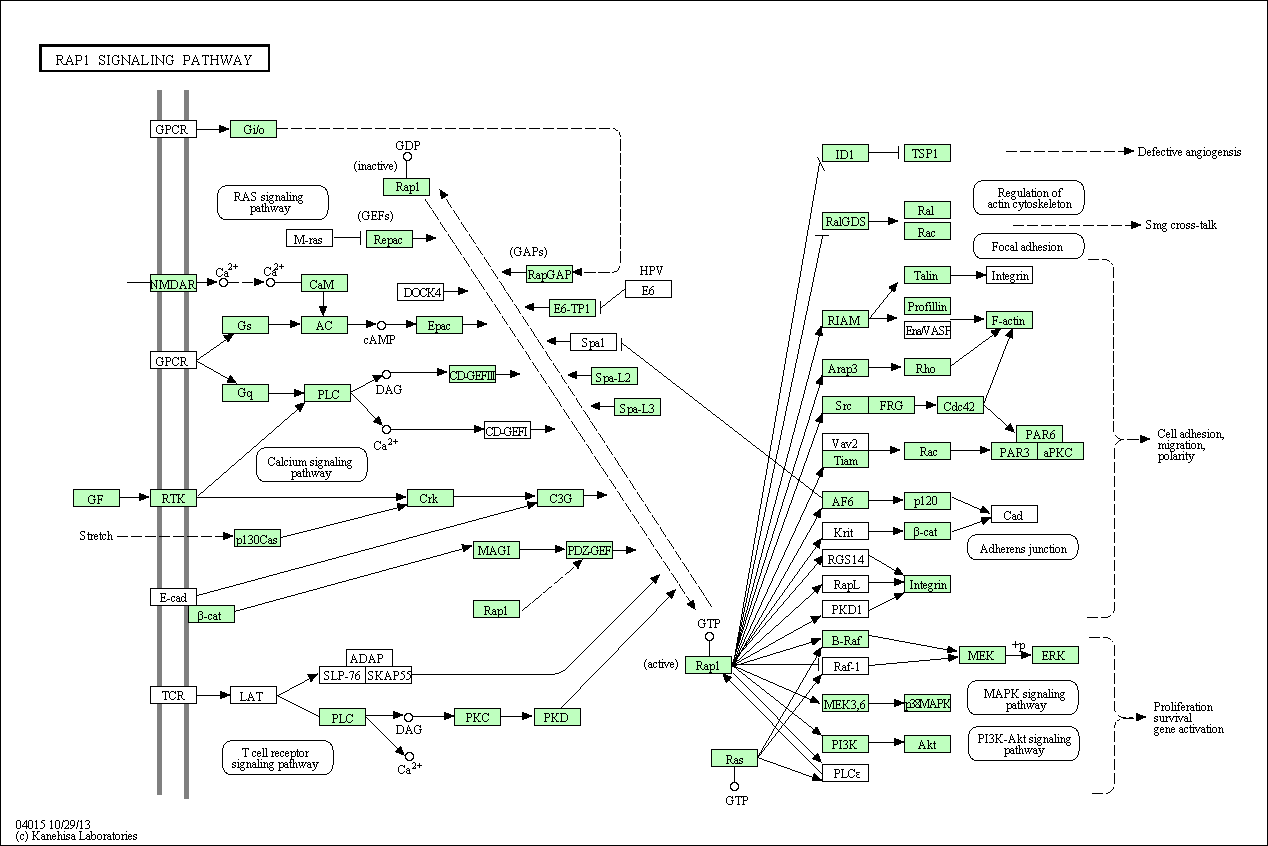

Supplement: Supplemental Information 9 [file peerj-04-1616-s009.gz › map/map04015.png]

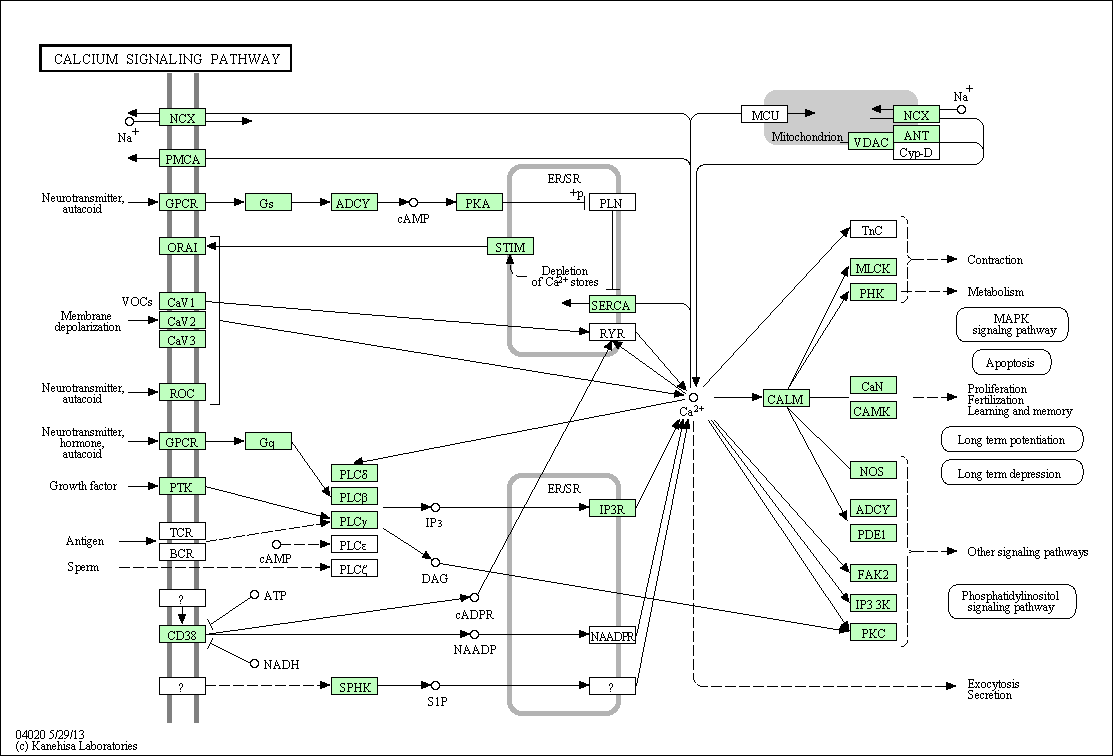

Supplement: Supplemental Information 9 [file peerj-04-1616-s009.gz › map/map04020.png]

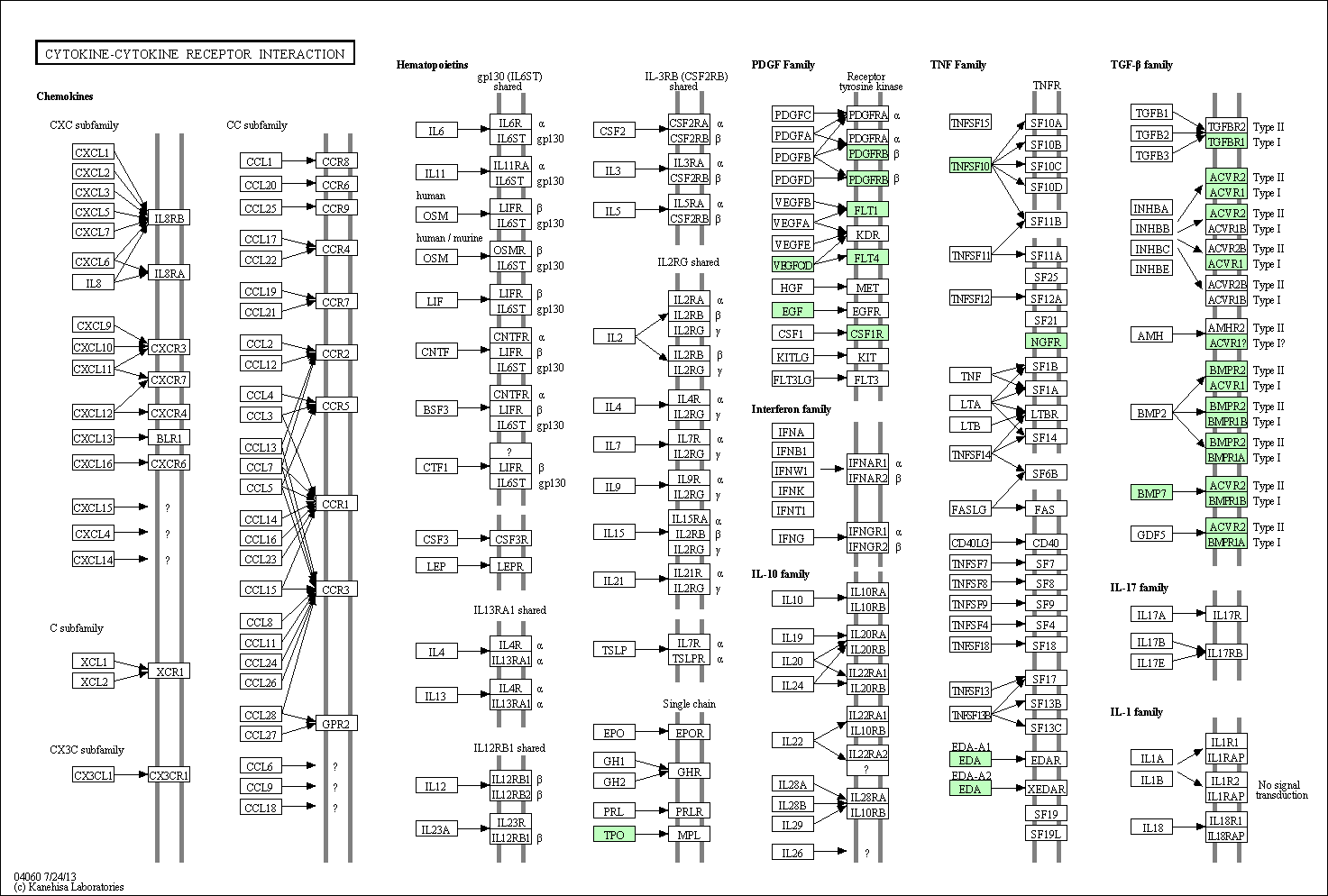

Supplement: Supplemental Information 9 [file peerj-04-1616-s009.gz › map/map04060.png]

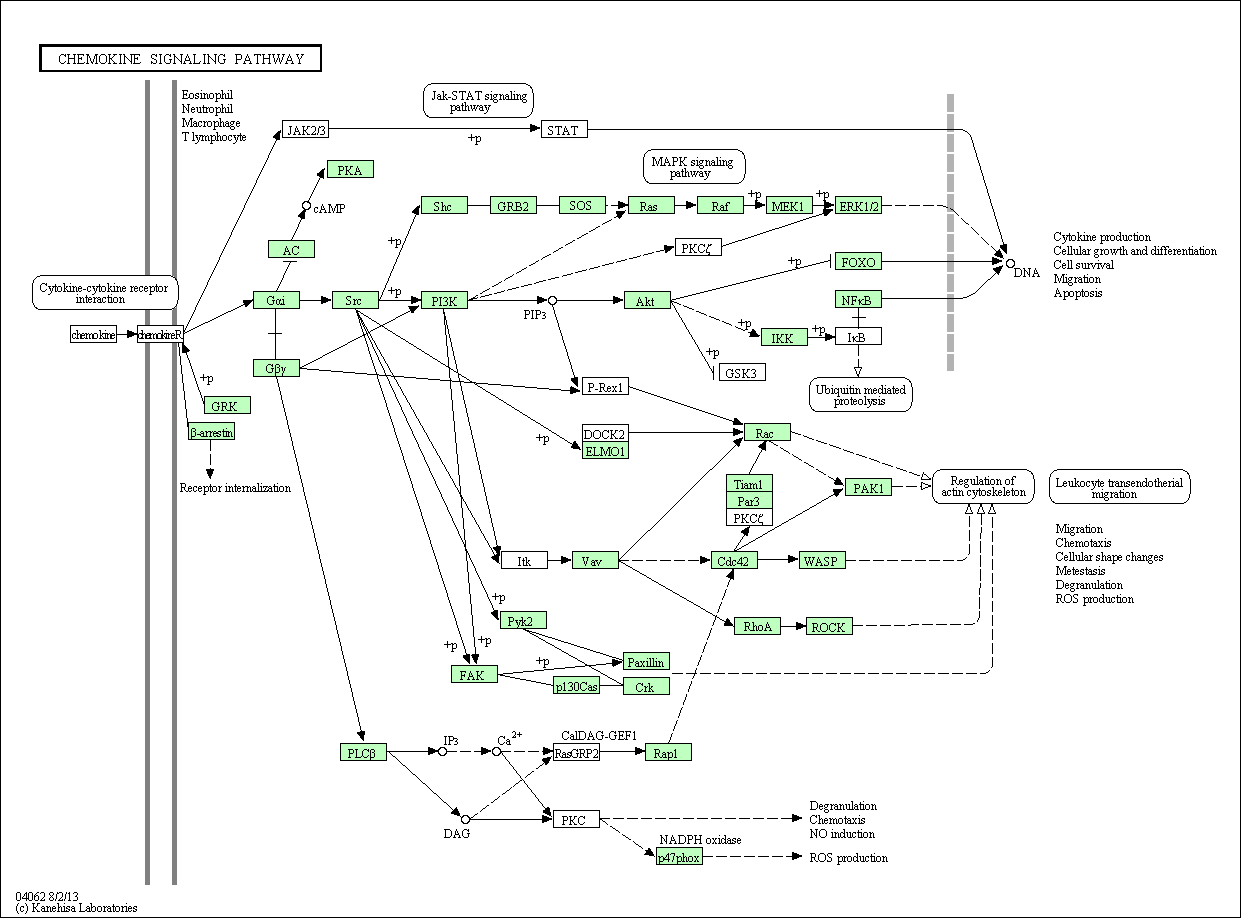

Supplement: Supplemental Information 9 [file peerj-04-1616-s009.gz › map/map04062.png]

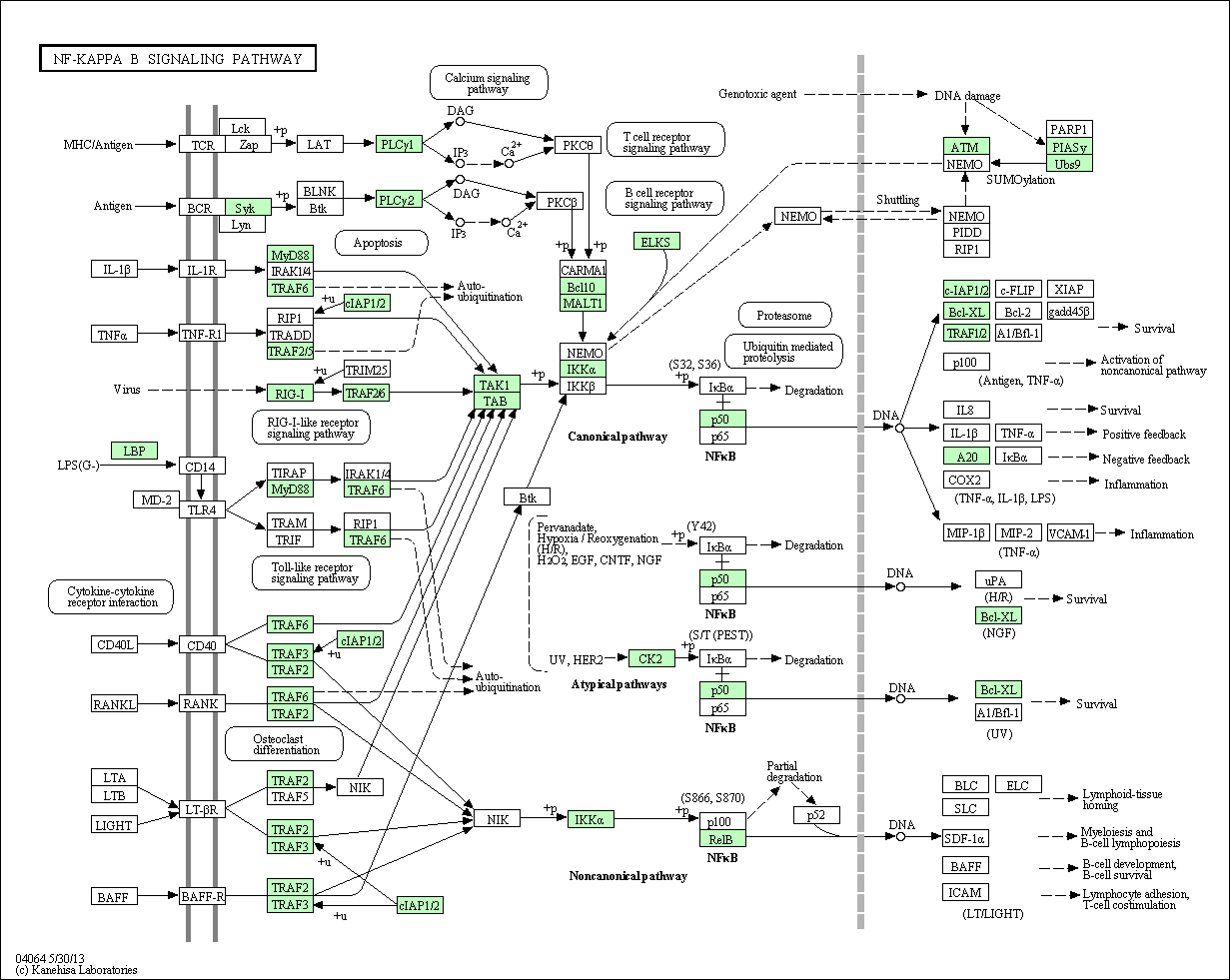

Supplement: Supplemental Information 9 [file peerj-04-1616-s009.gz › map/map04064.png]

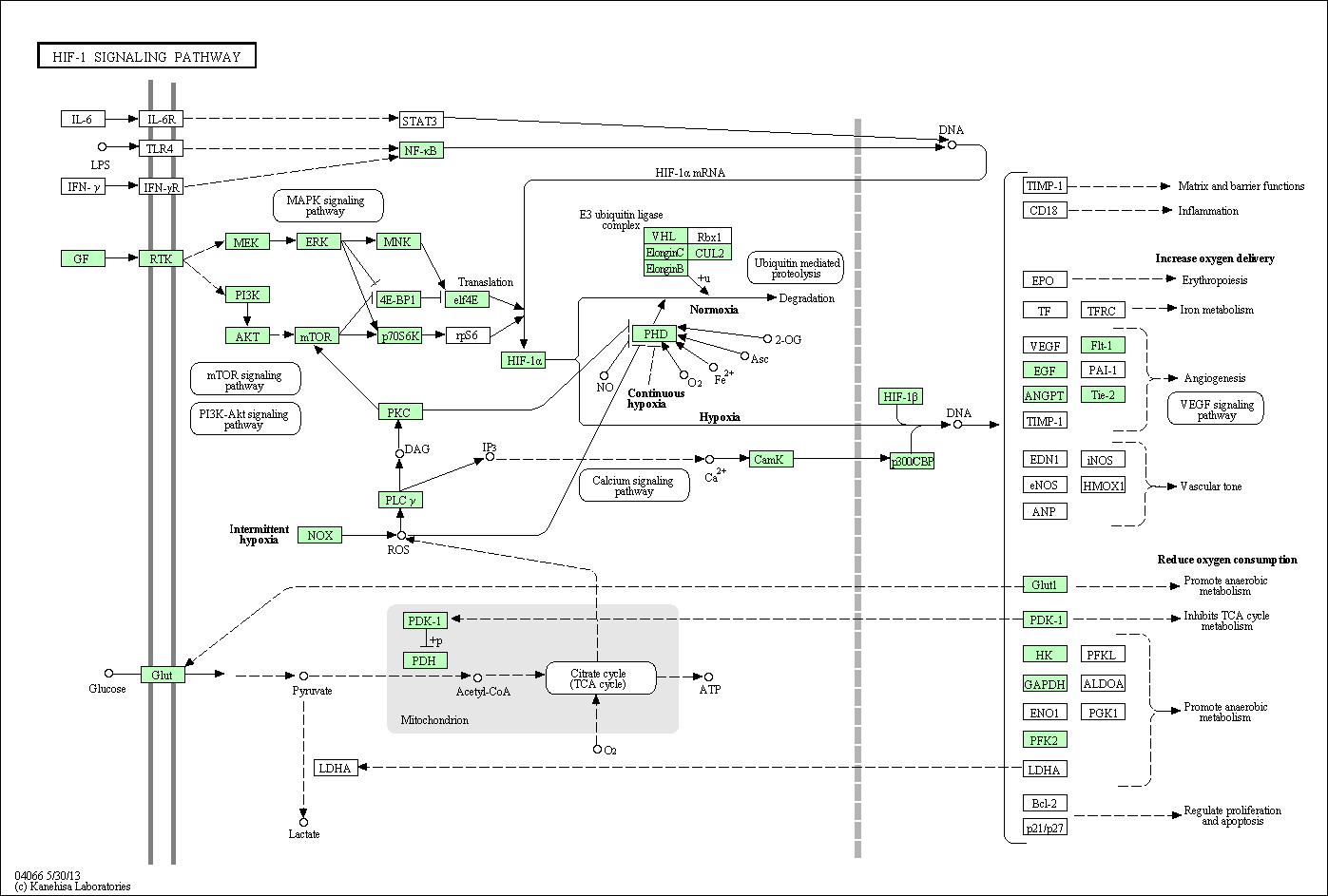

Supplement: Supplemental Information 9 [file peerj-04-1616-s009.gz › map/map04066.png]

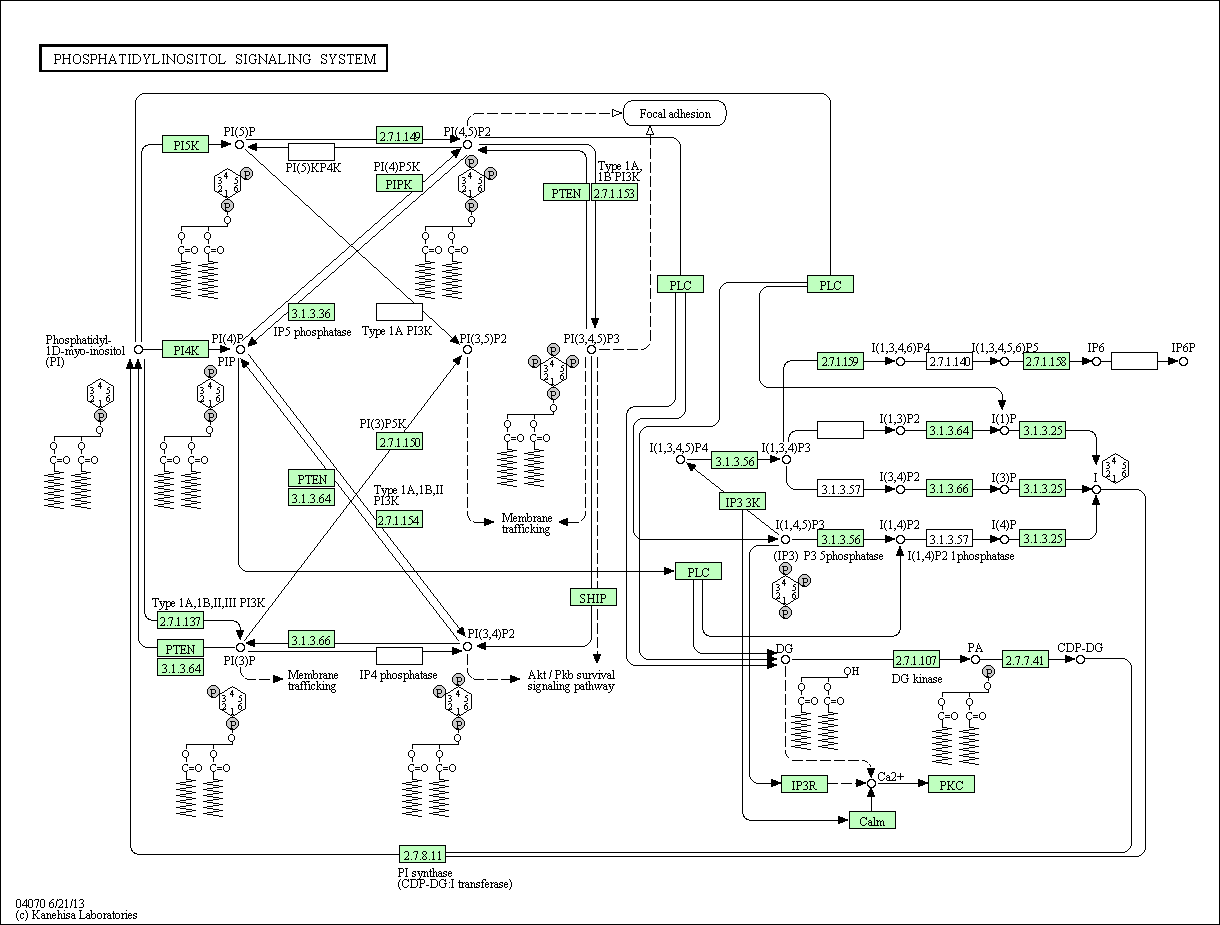

Supplement: Supplemental Information 9 [file peerj-04-1616-s009.gz › map/map04070.png]

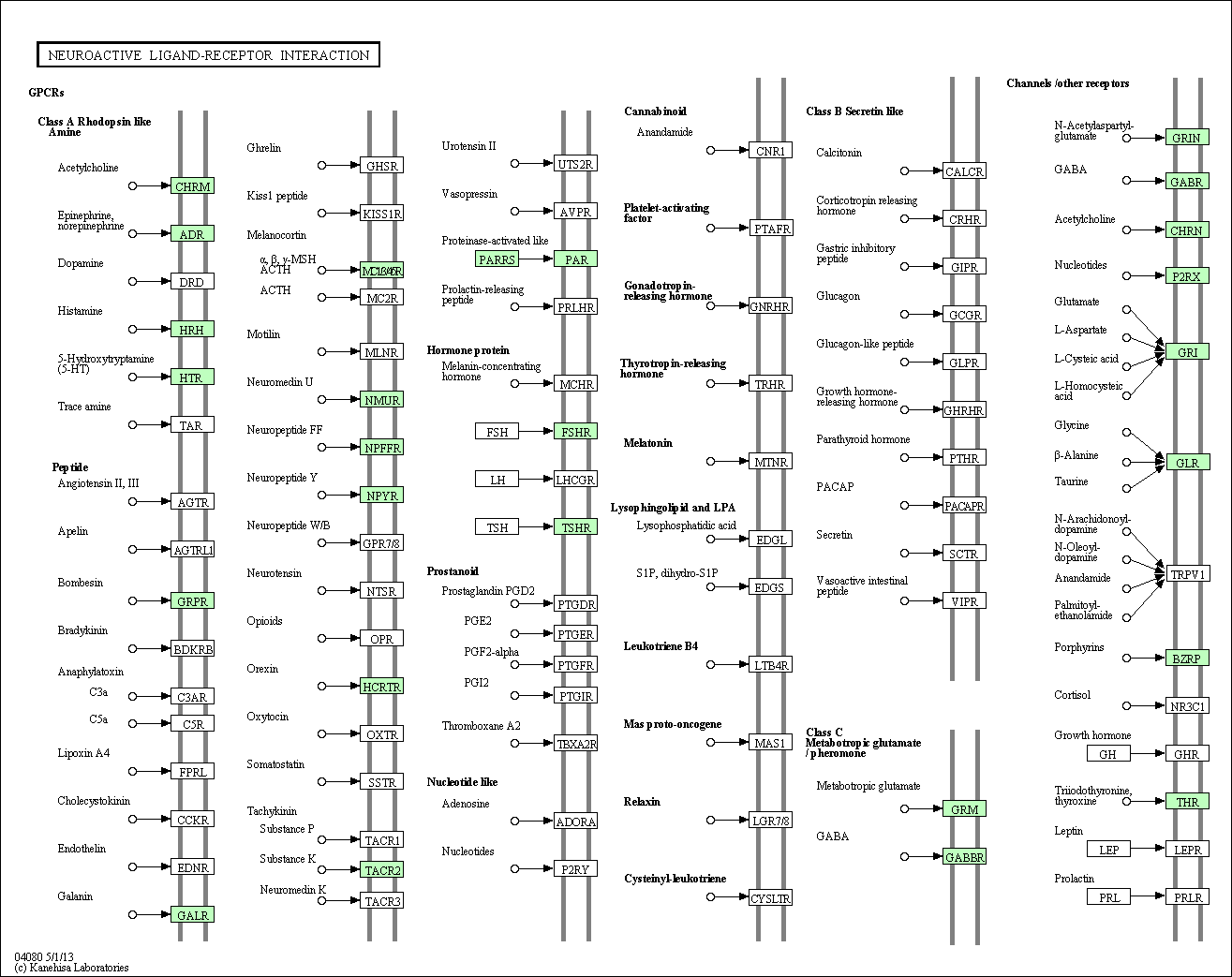

Supplement: Supplemental Information 9 [file peerj-04-1616-s009.gz › map/map04080.png]

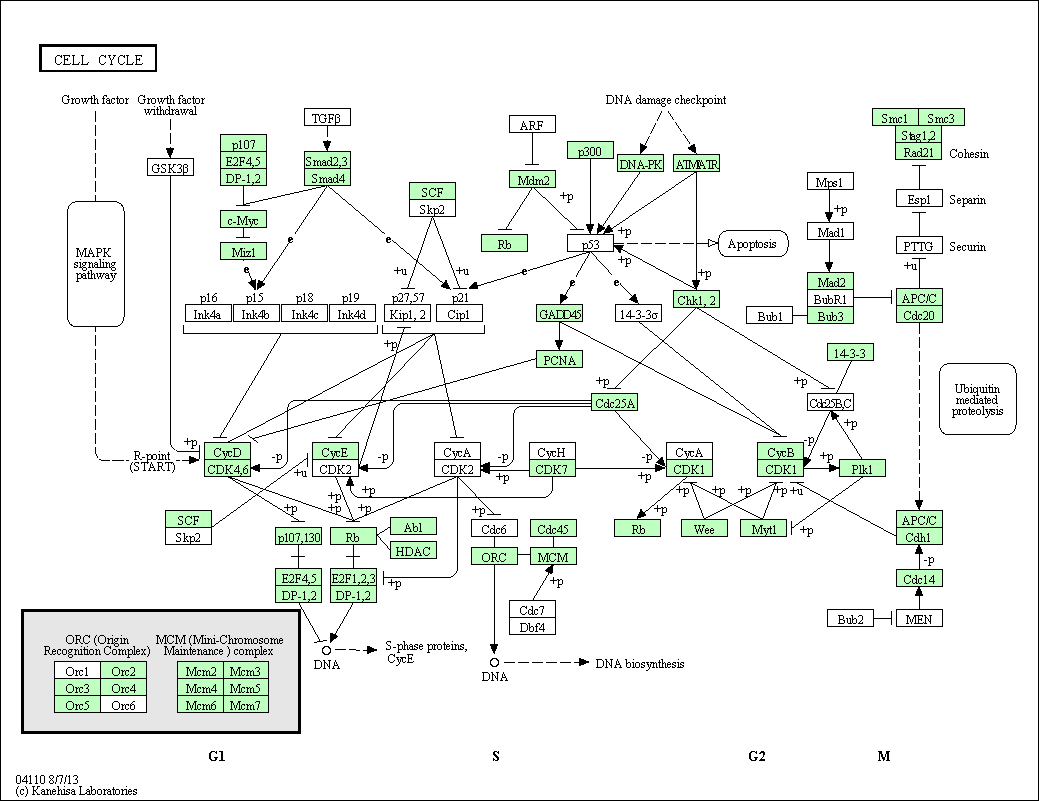

Supplement: Supplemental Information 9 [file peerj-04-1616-s009.gz › map/map04110.png]

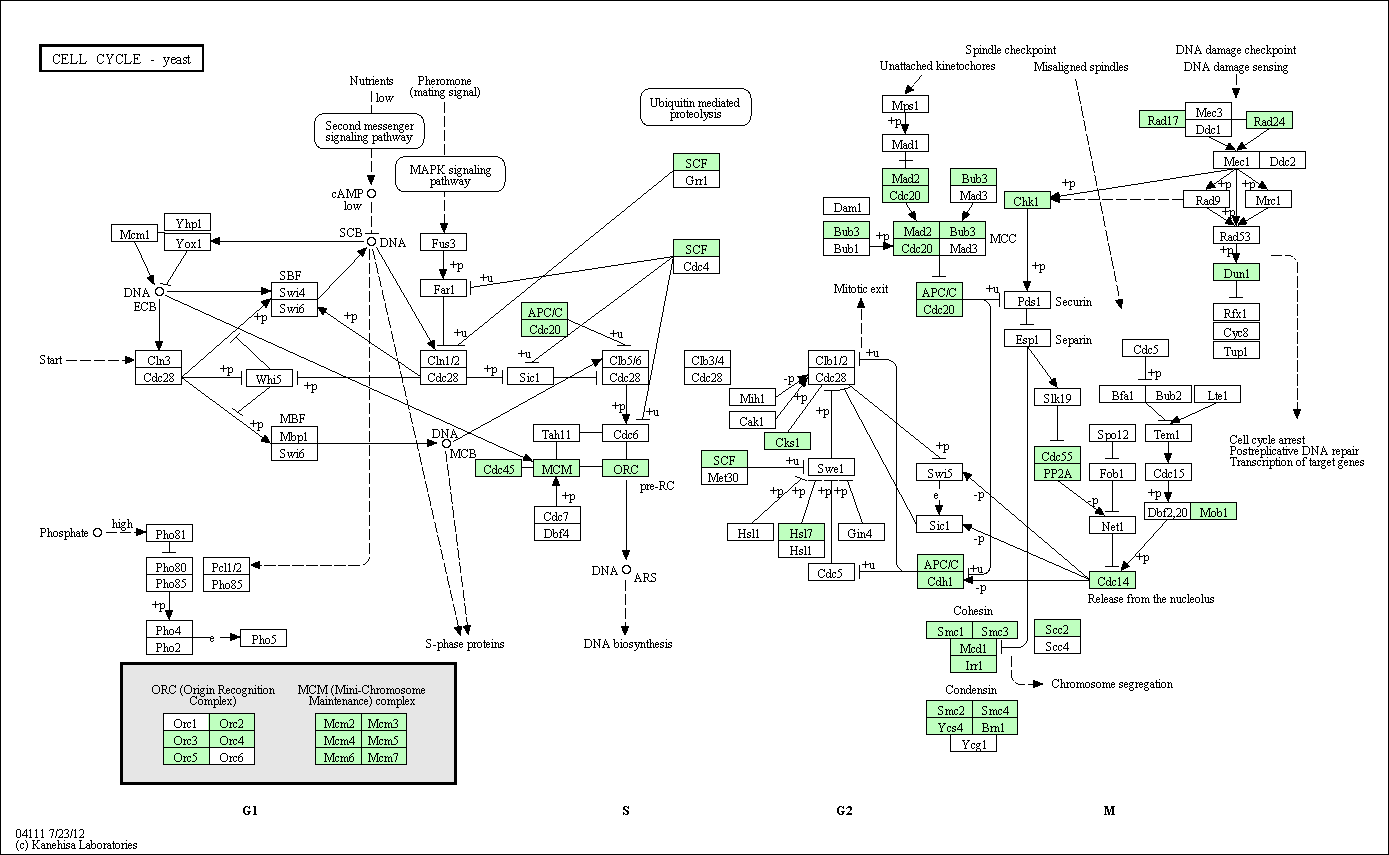

Supplement: Supplemental Information 9 [file peerj-04-1616-s009.gz › map/map04111.png]

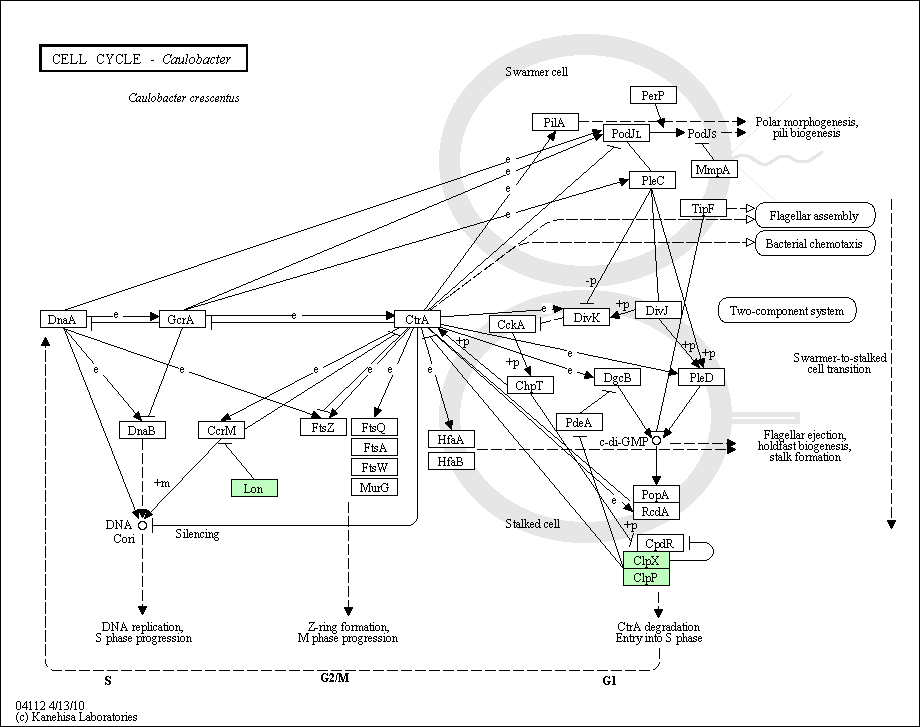

Supplement: Supplemental Information 9 [file peerj-04-1616-s009.gz › map/map04112.png]

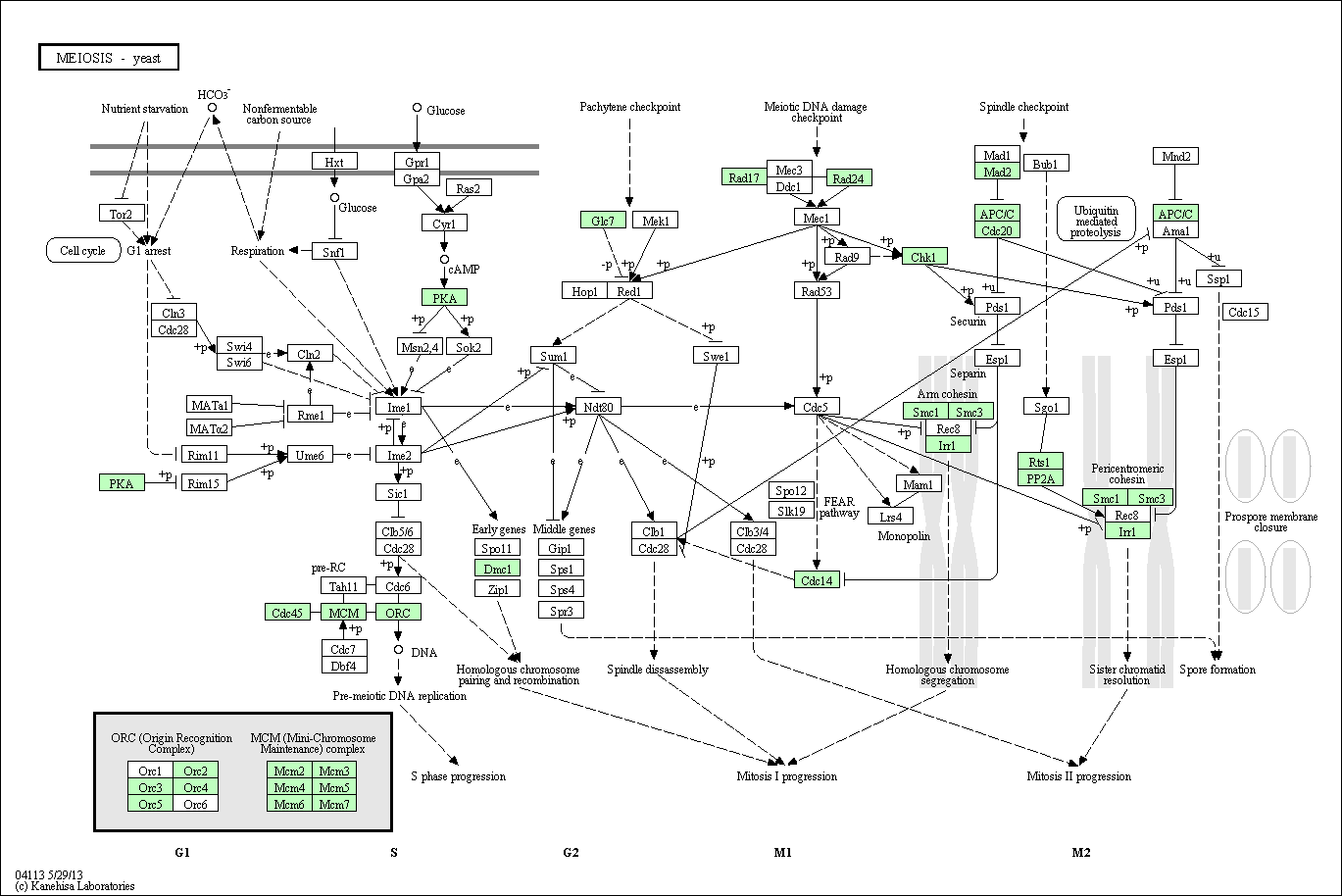

Supplement: Supplemental Information 9 [file peerj-04-1616-s009.gz › map/map04113.png]

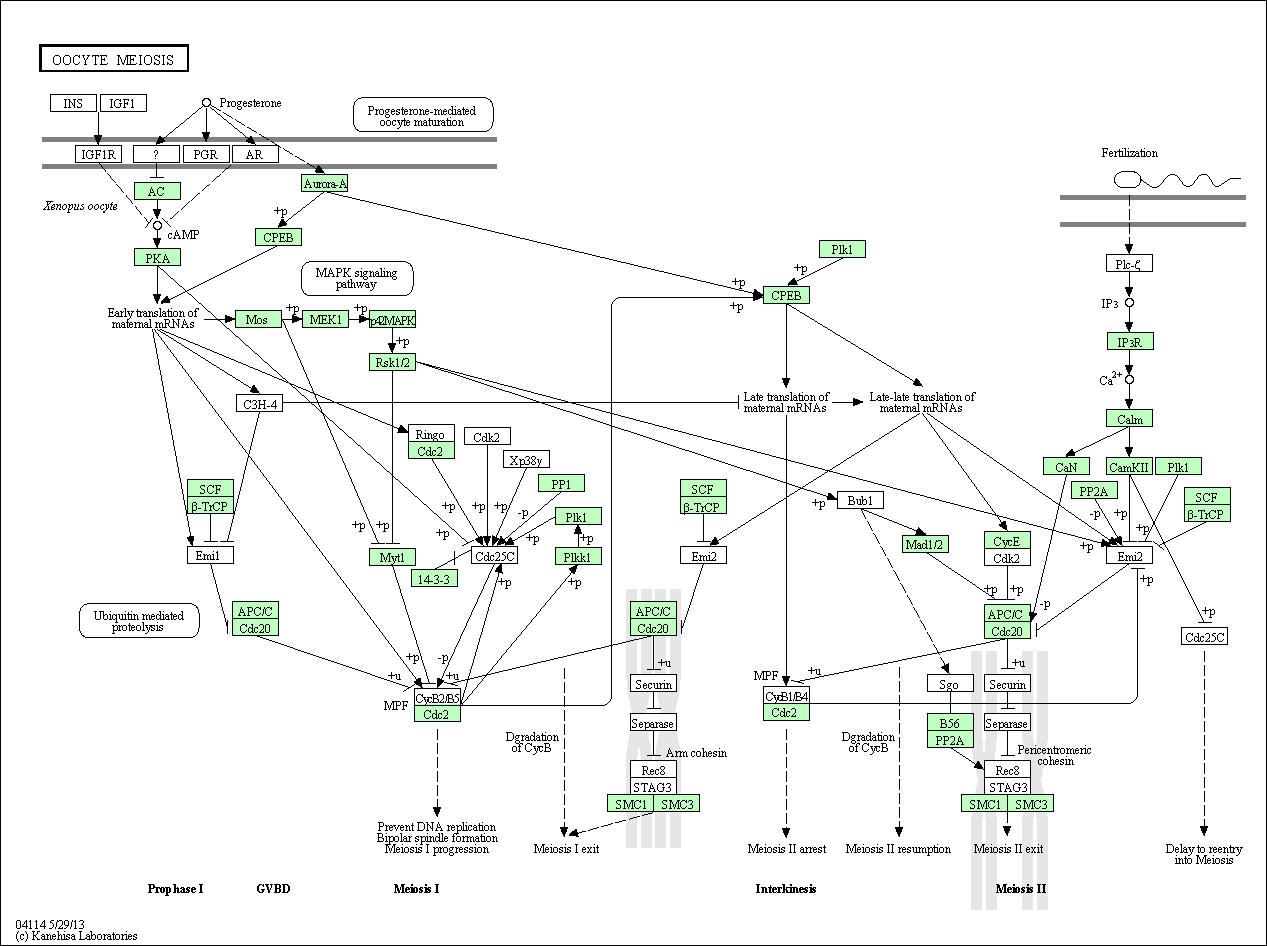

Supplement: Supplemental Information 9 [file peerj-04-1616-s009.gz › map/map04114.png]

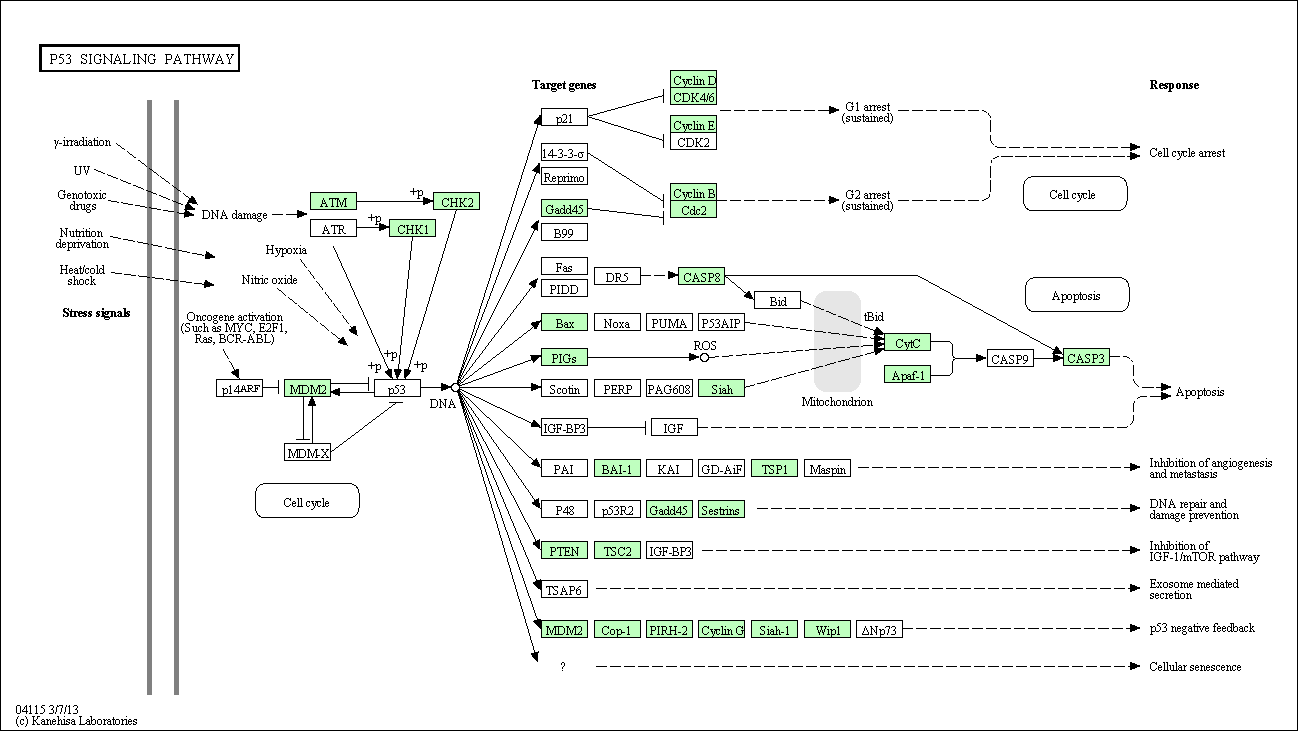

Supplement: Supplemental Information 9 [file peerj-04-1616-s009.gz › map/map04115.png]

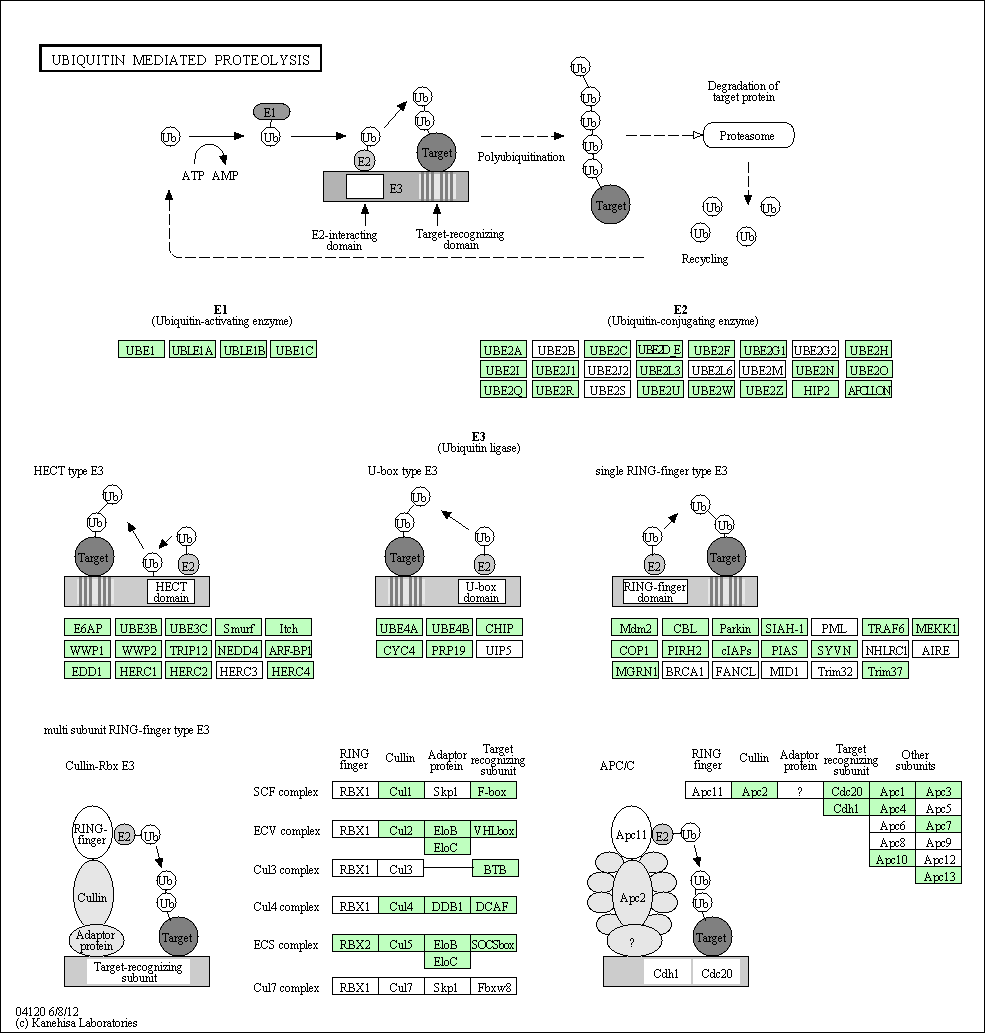

Supplement: Supplemental Information 9 [file peerj-04-1616-s009.gz › map/map04120.png]

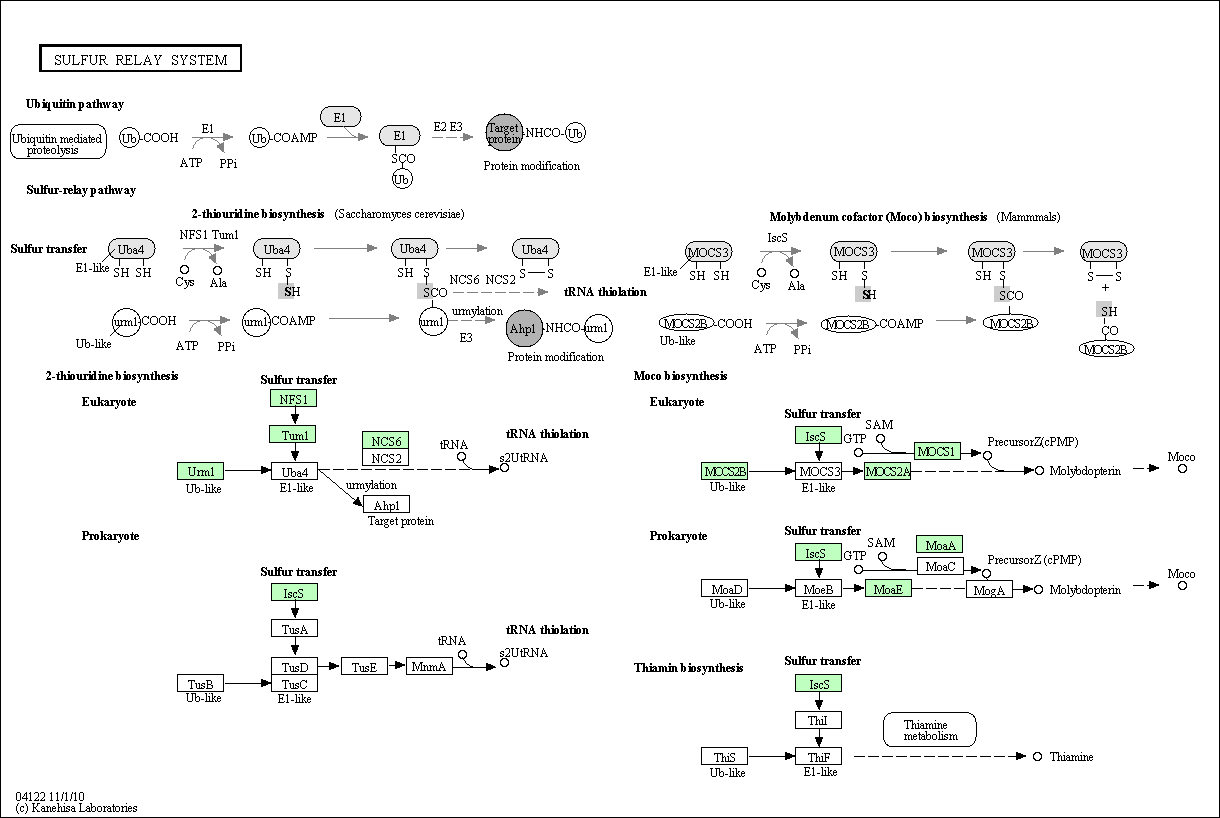

Supplement: Supplemental Information 9 [file peerj-04-1616-s009.gz › map/map04122.png]

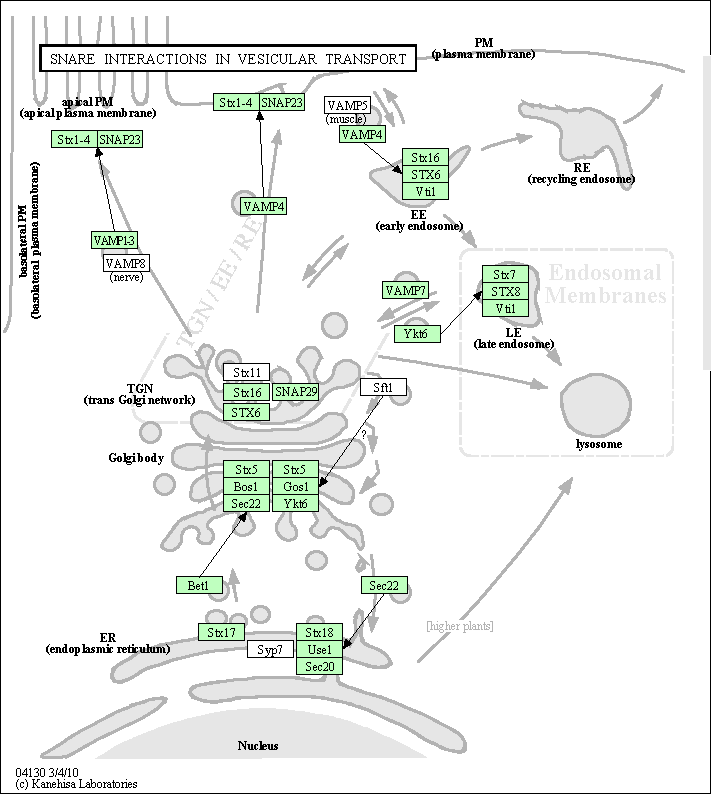

Supplement: Supplemental Information 9 [file peerj-04-1616-s009.gz › map/map04130.png]

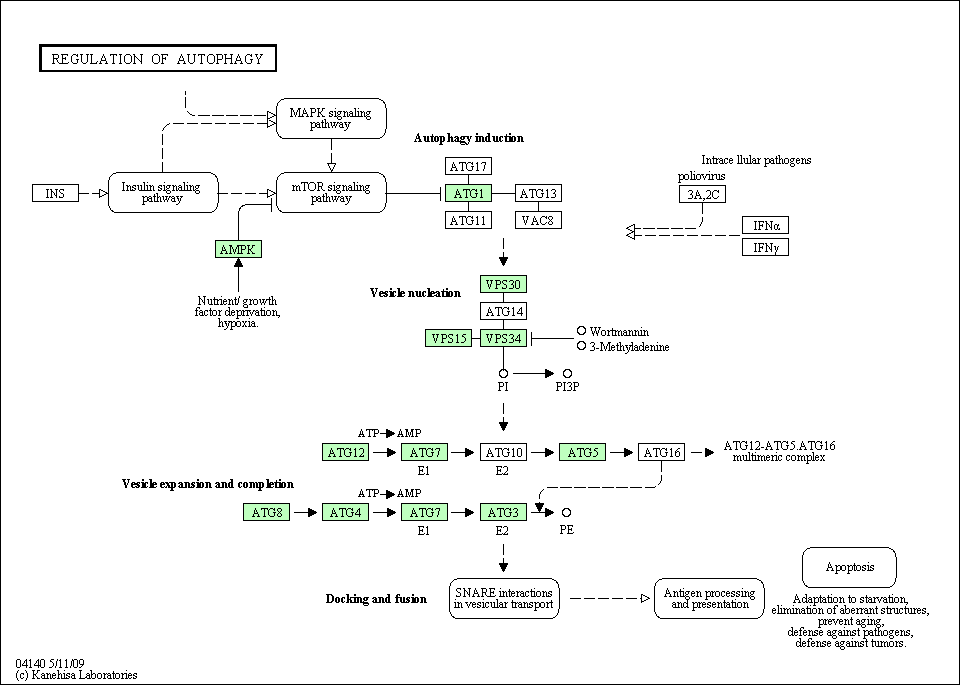

Supplement: Supplemental Information 9 [file peerj-04-1616-s009.gz › map/map04140.png]

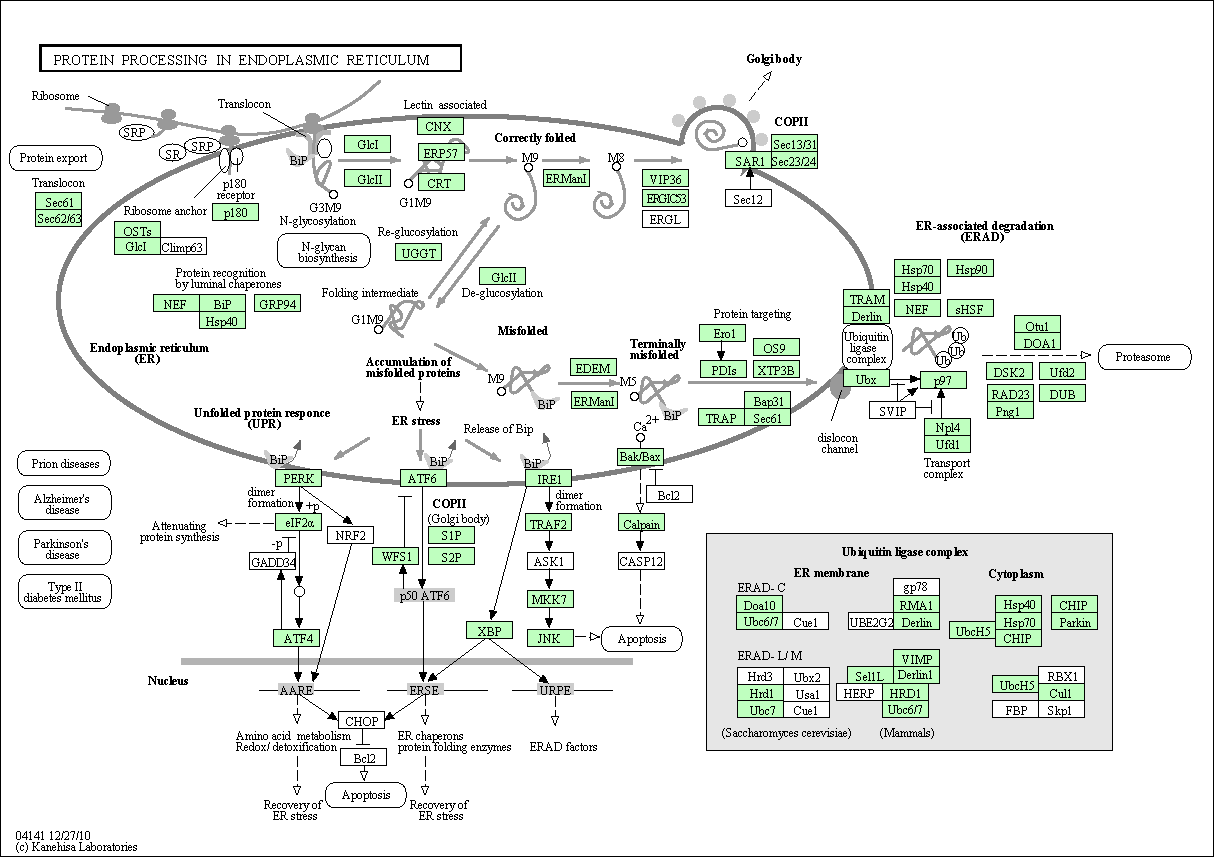

Supplement: Supplemental Information 9 [file peerj-04-1616-s009.gz › map/map04141.png]

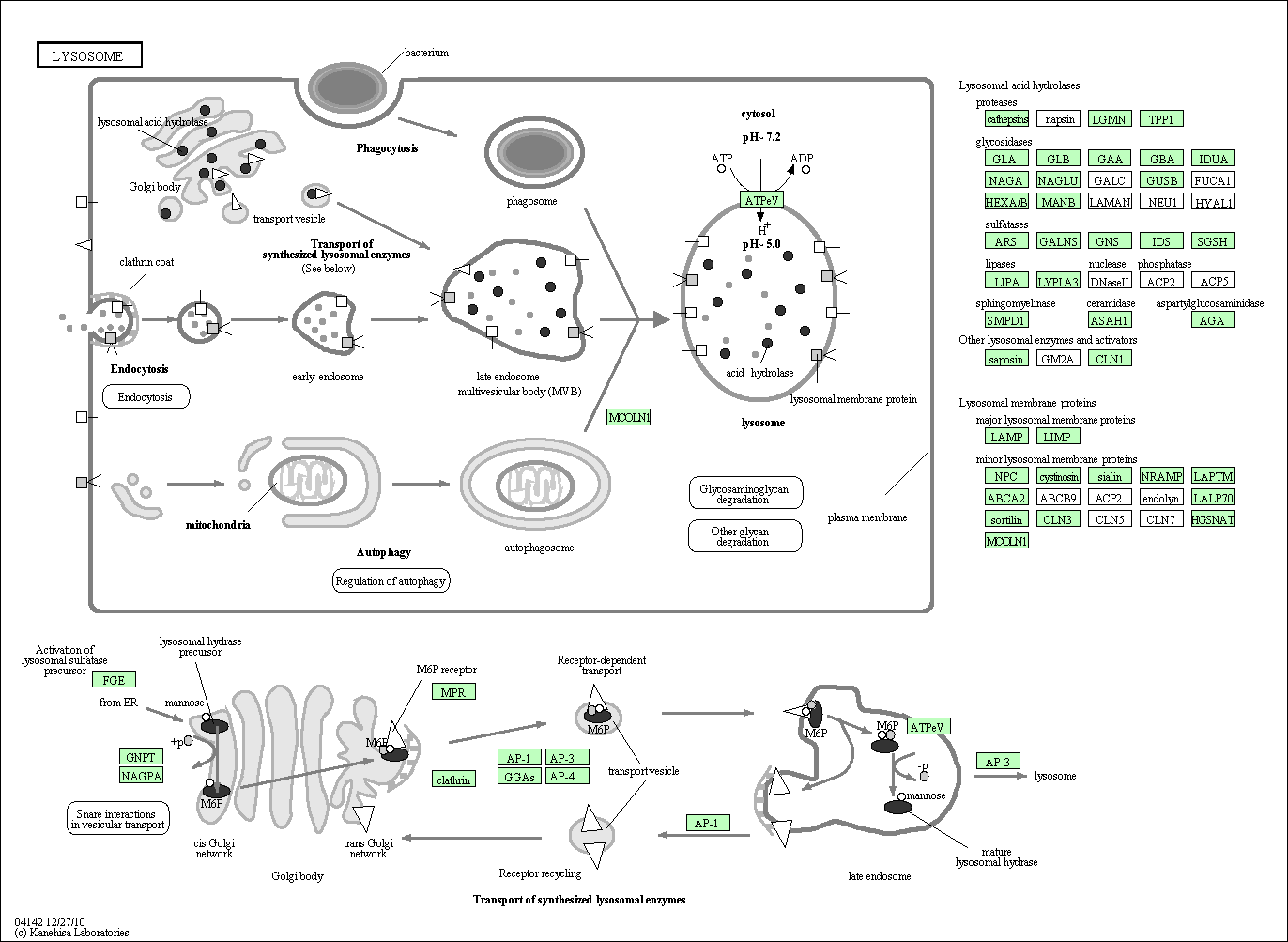

Supplement: Supplemental Information 9 [file peerj-04-1616-s009.gz › map/map04142.png]

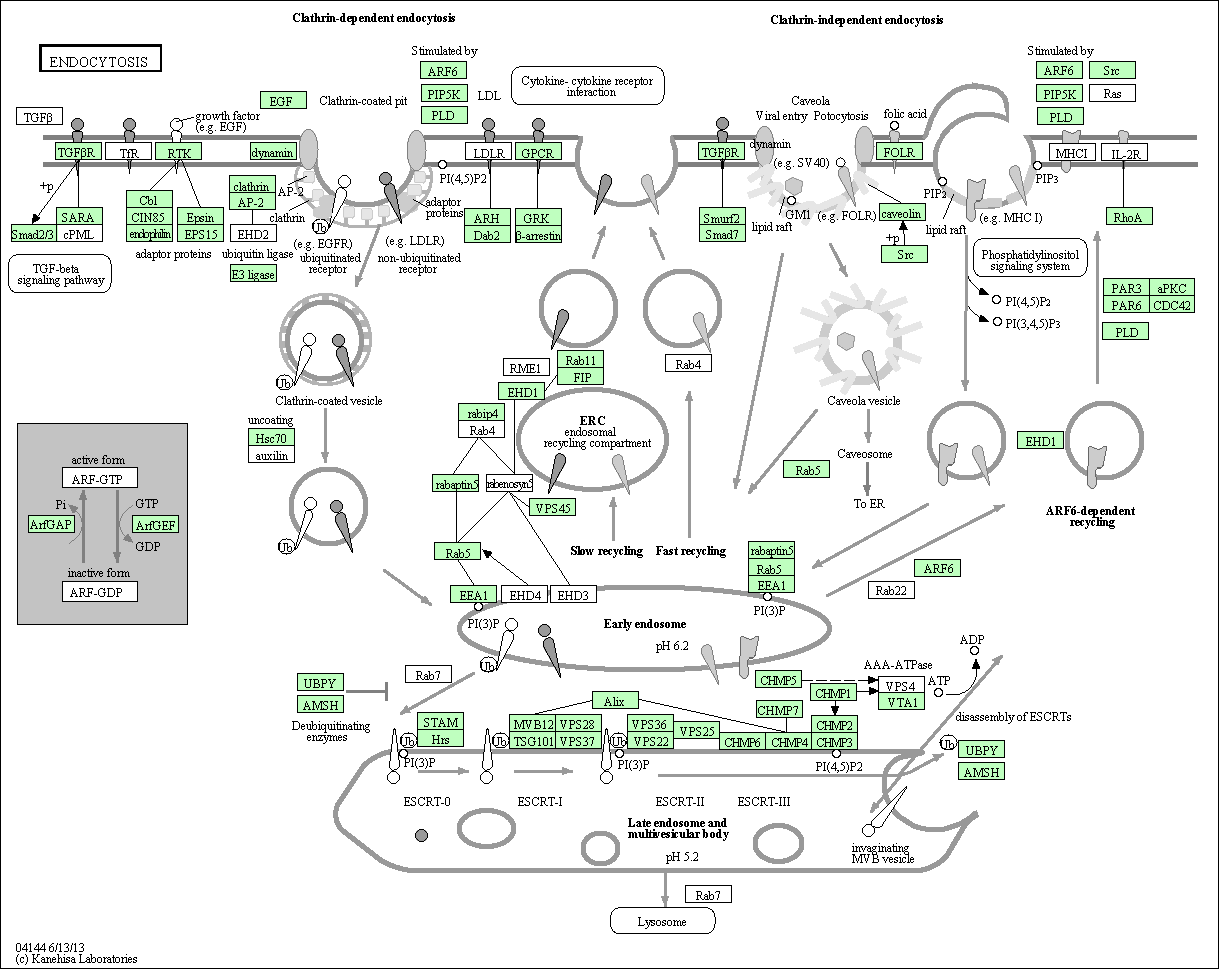

Supplement: Supplemental Information 9 [file peerj-04-1616-s009.gz › map/map04144.png]

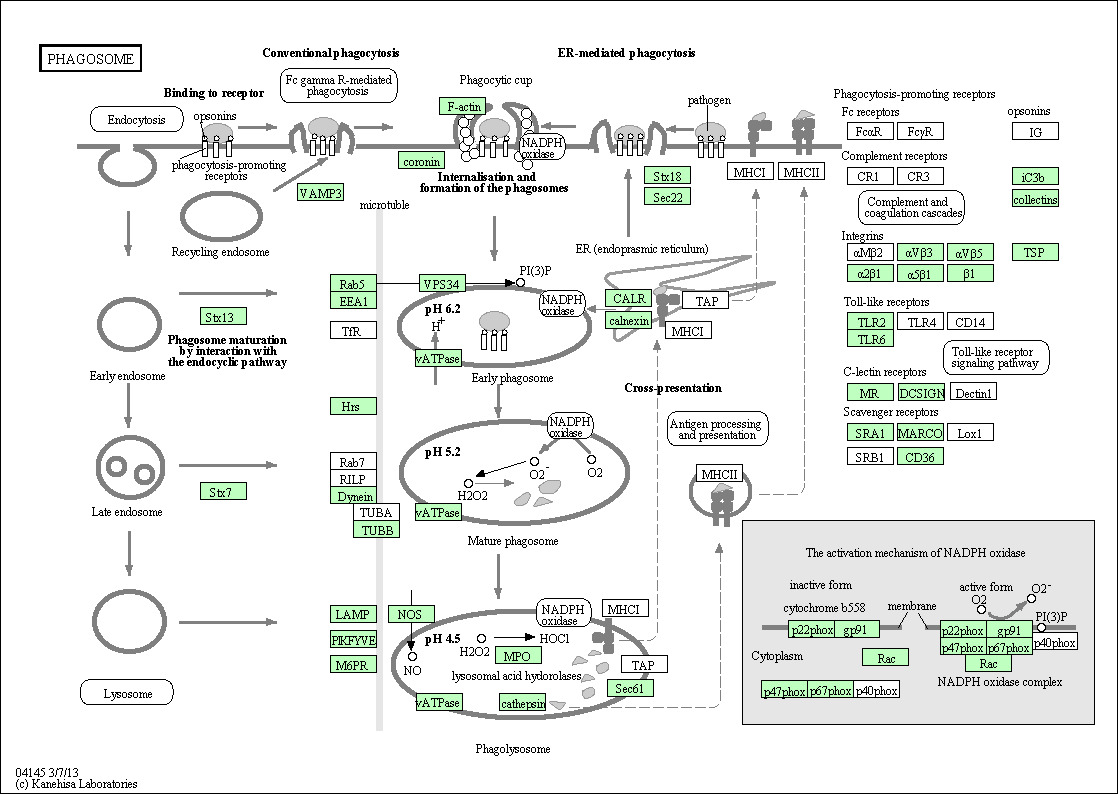

Supplement: Supplemental Information 9 [file peerj-04-1616-s009.gz › map/map04145.png]

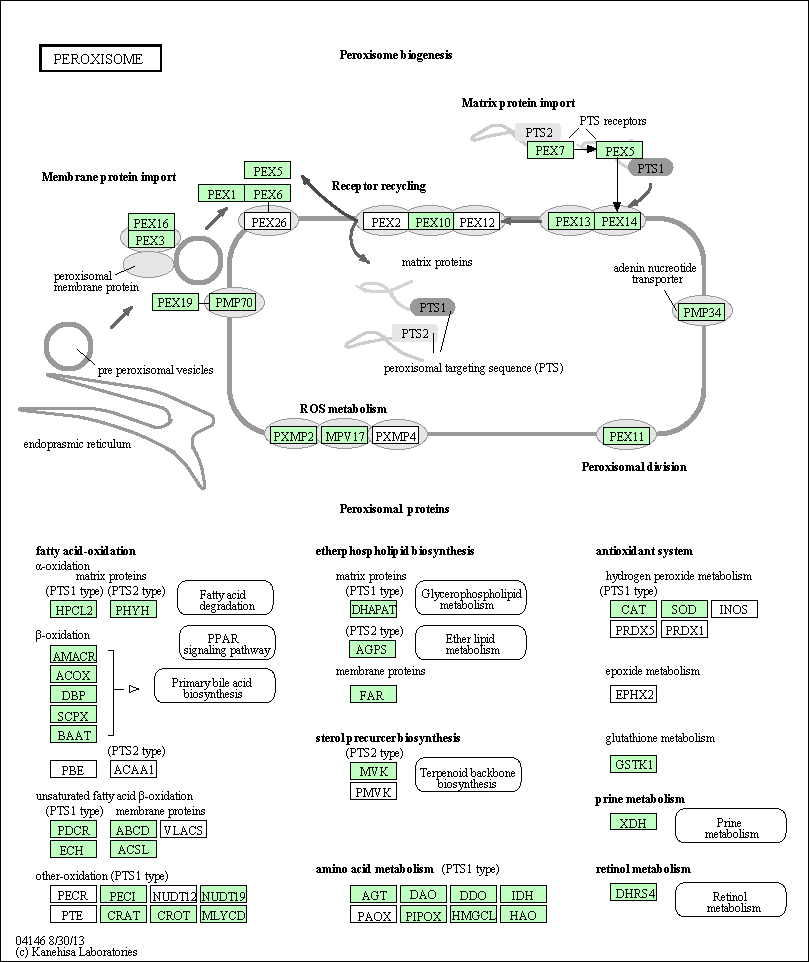

Supplement: Supplemental Information 9 [file peerj-04-1616-s009.gz › map/map04146.png]

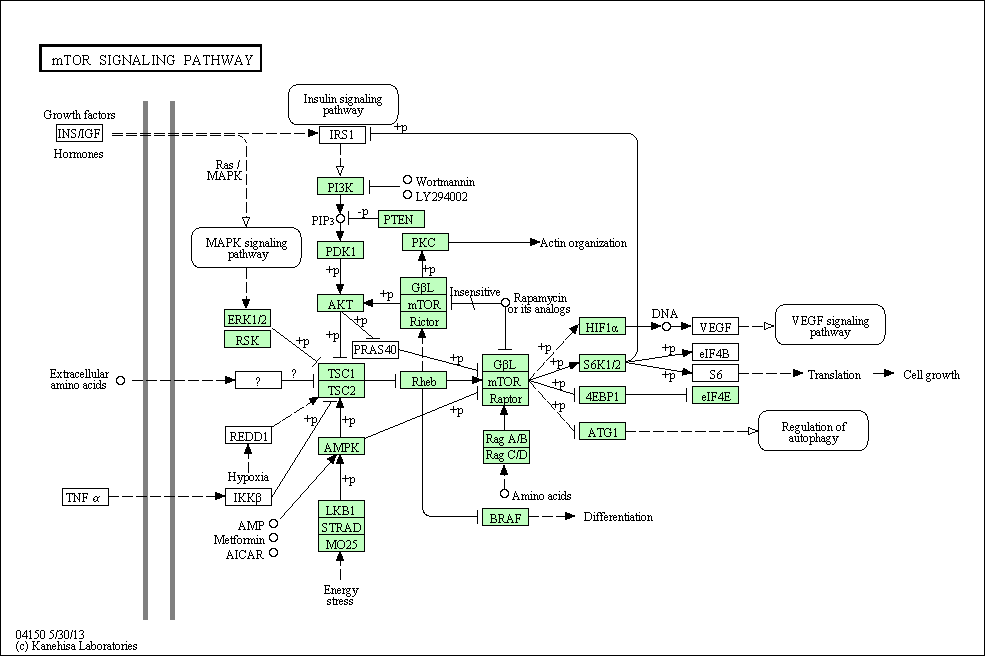

Supplement: Supplemental Information 9 [file peerj-04-1616-s009.gz › map/map04150.png]

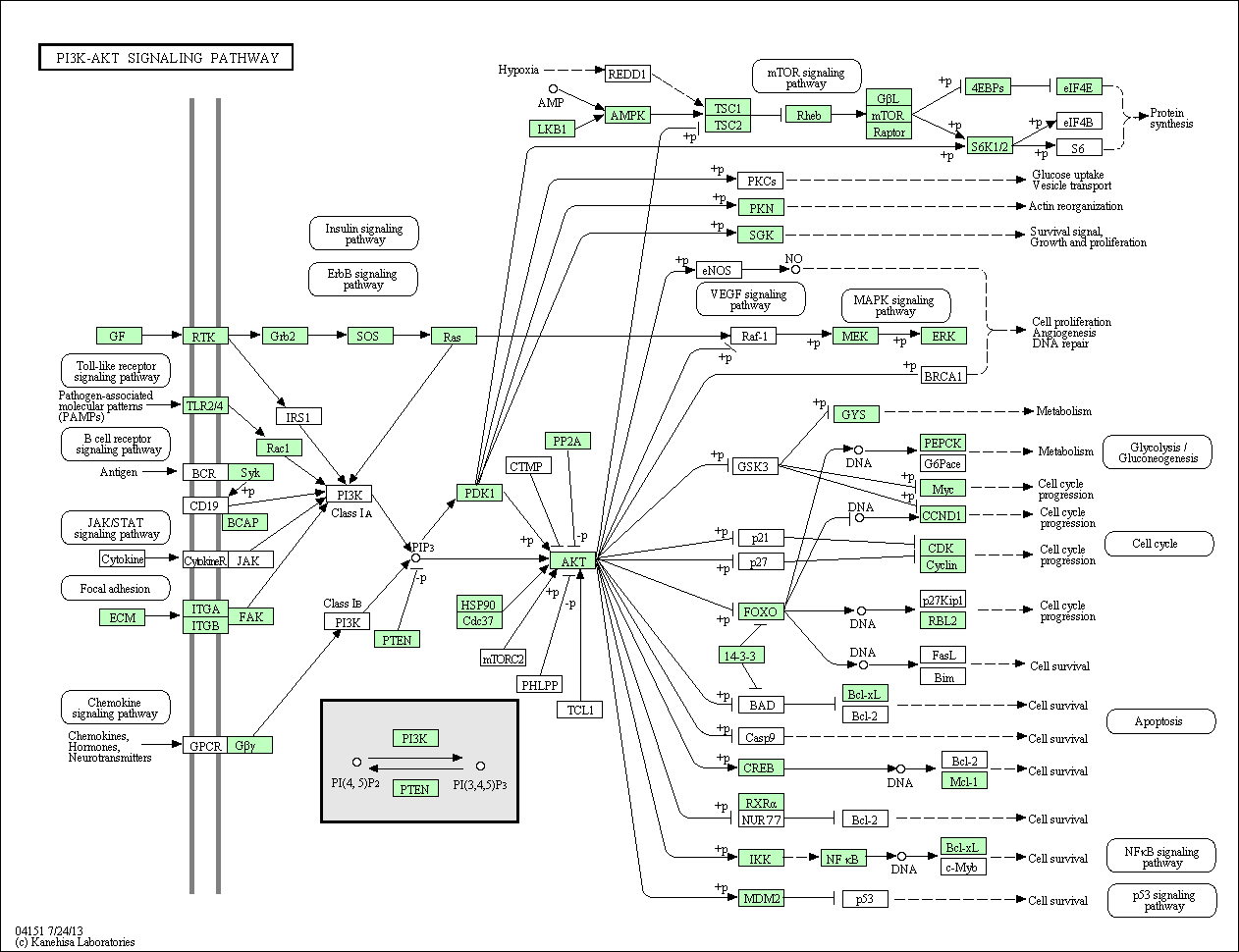

Supplement: Supplemental Information 9 [file peerj-04-1616-s009.gz › map/map04151.png]

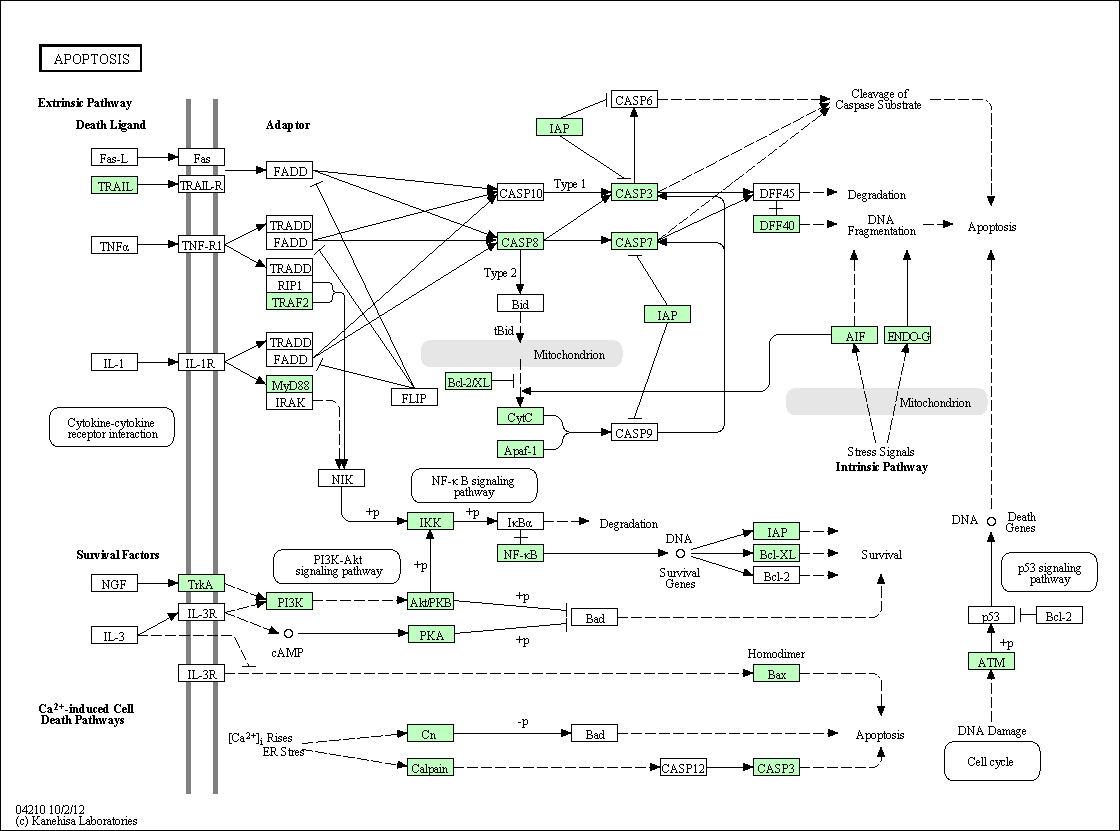

Supplement: Supplemental Information 9 [file peerj-04-1616-s009.gz › map/map04210.png]

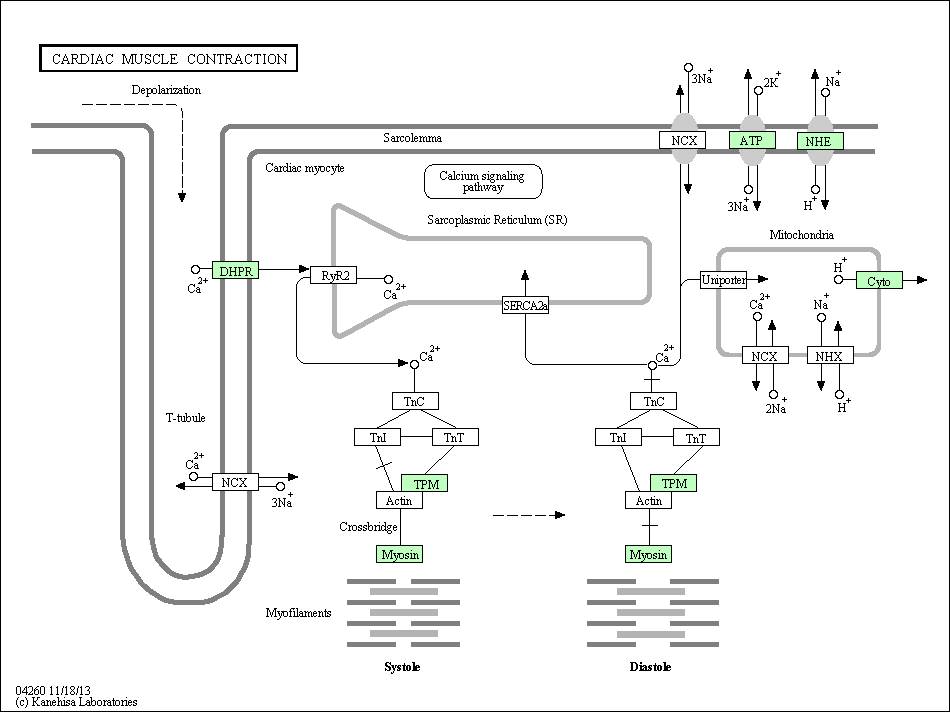

Supplement: Supplemental Information 9 [file peerj-04-1616-s009.gz › map/map04260.png]

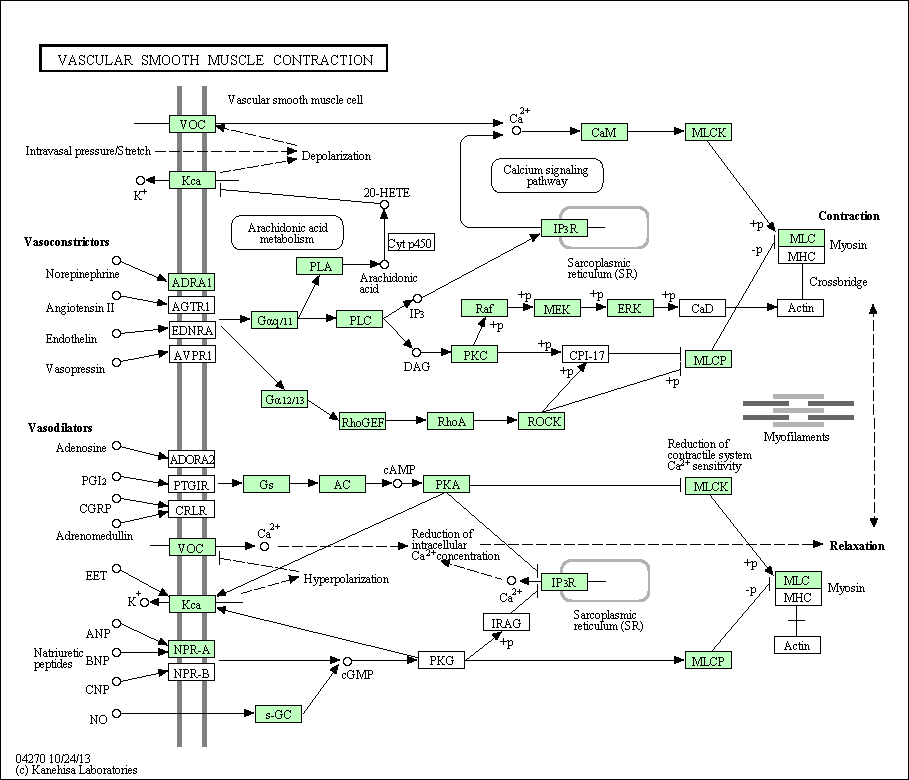

Supplement: Supplemental Information 9 [file peerj-04-1616-s009.gz › map/map04270.png]

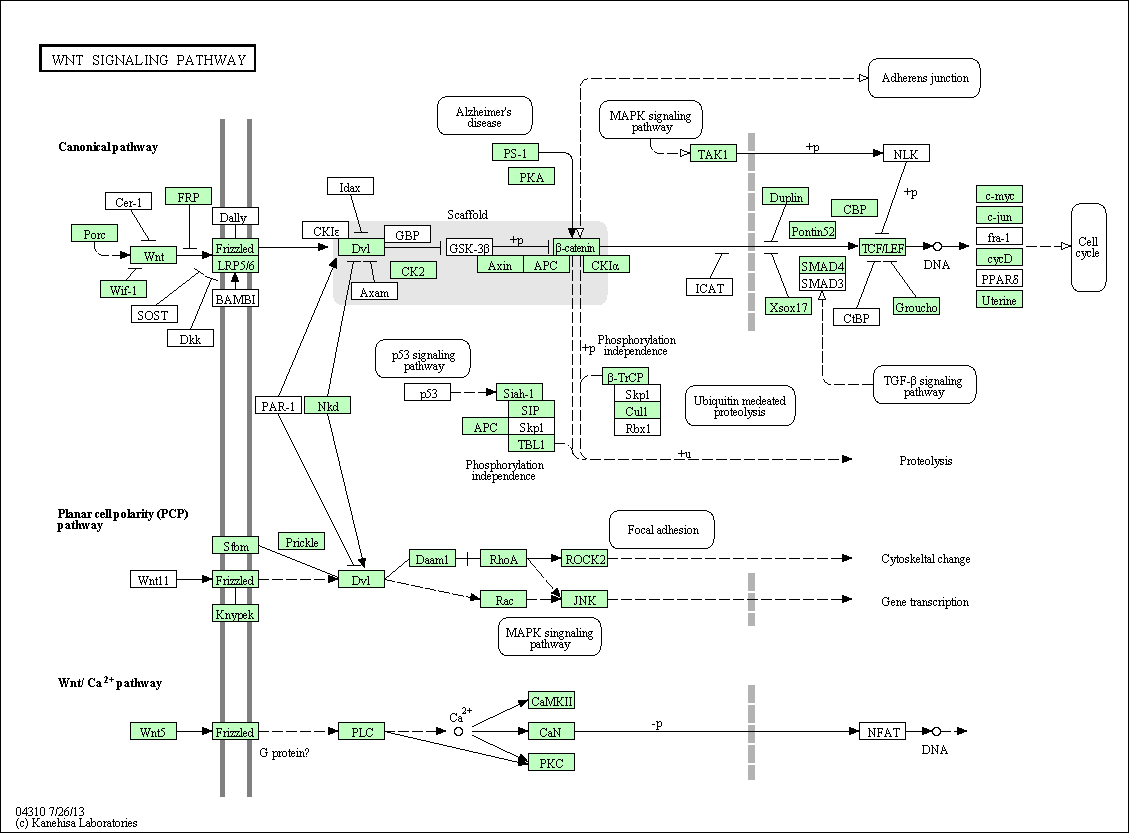

Supplement: Supplemental Information 9 [file peerj-04-1616-s009.gz › map/map04310.png]

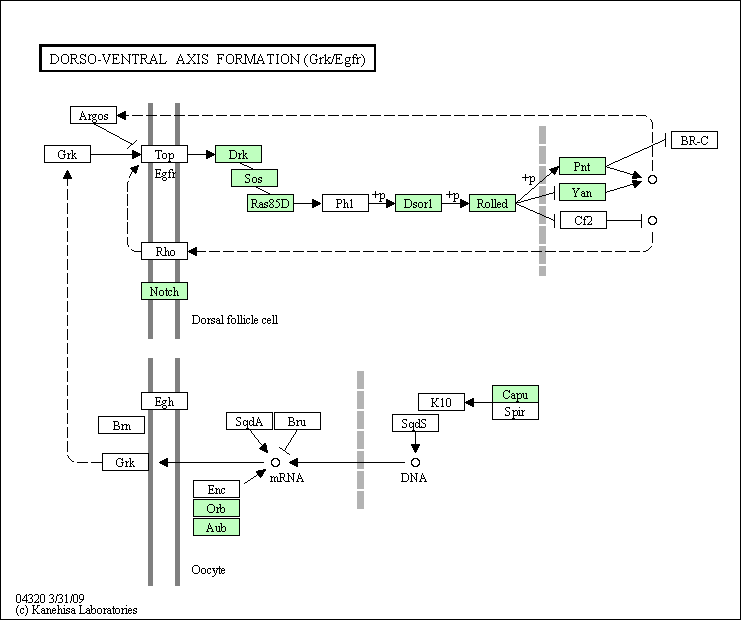

Supplement: Supplemental Information 9 [file peerj-04-1616-s009.gz › map/map04320.png]

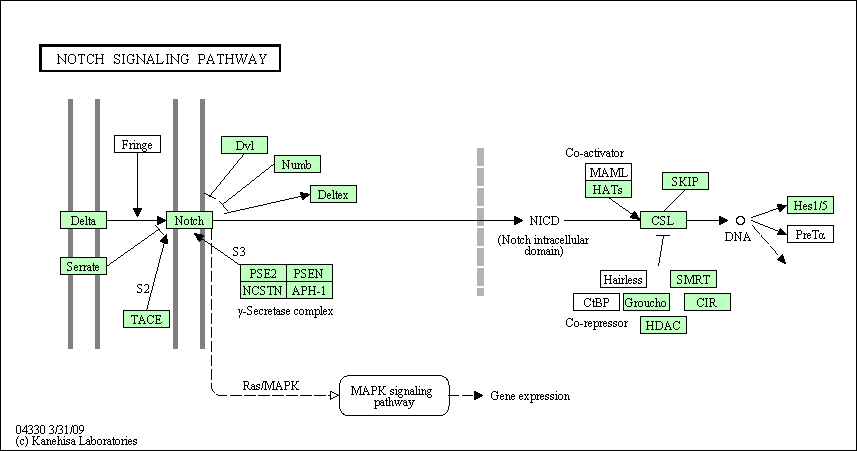

Supplement: Supplemental Information 9 [file peerj-04-1616-s009.gz › map/map04330.png]

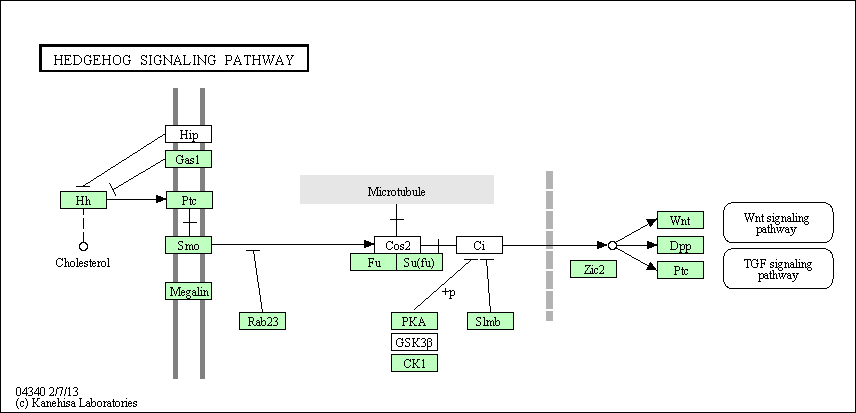

Supplement: Supplemental Information 9 [file peerj-04-1616-s009.gz › map/map04340.png]

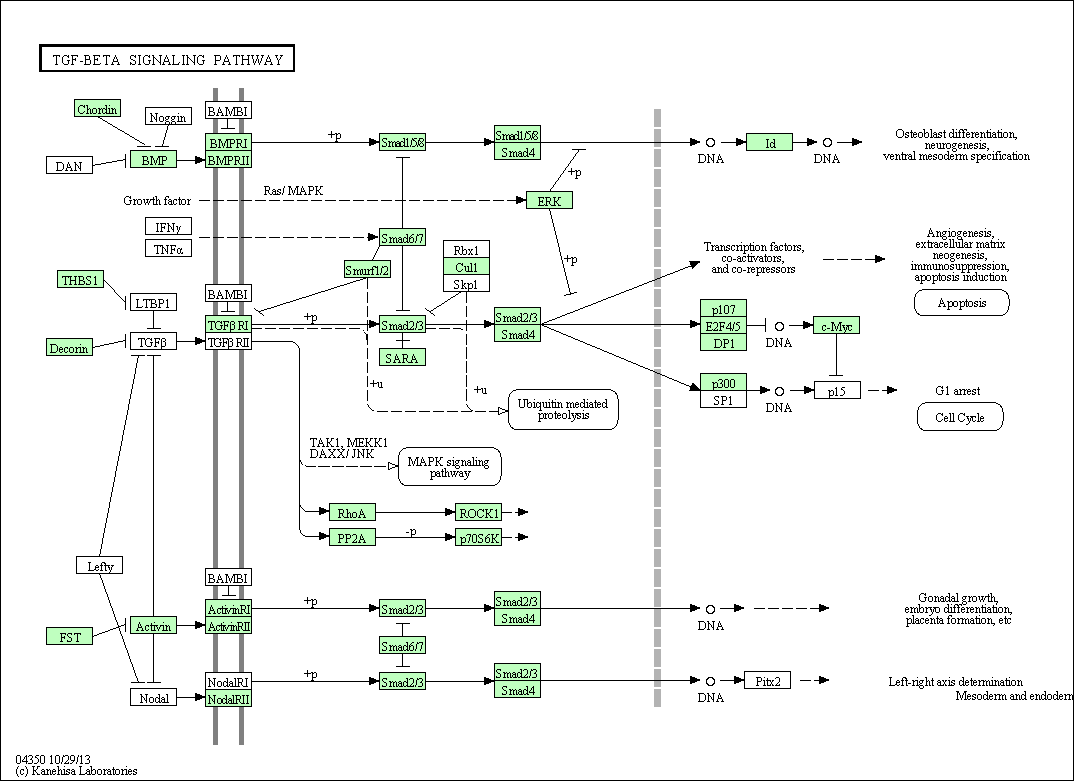

Supplement: Supplemental Information 9 [file peerj-04-1616-s009.gz › map/map04350.png]

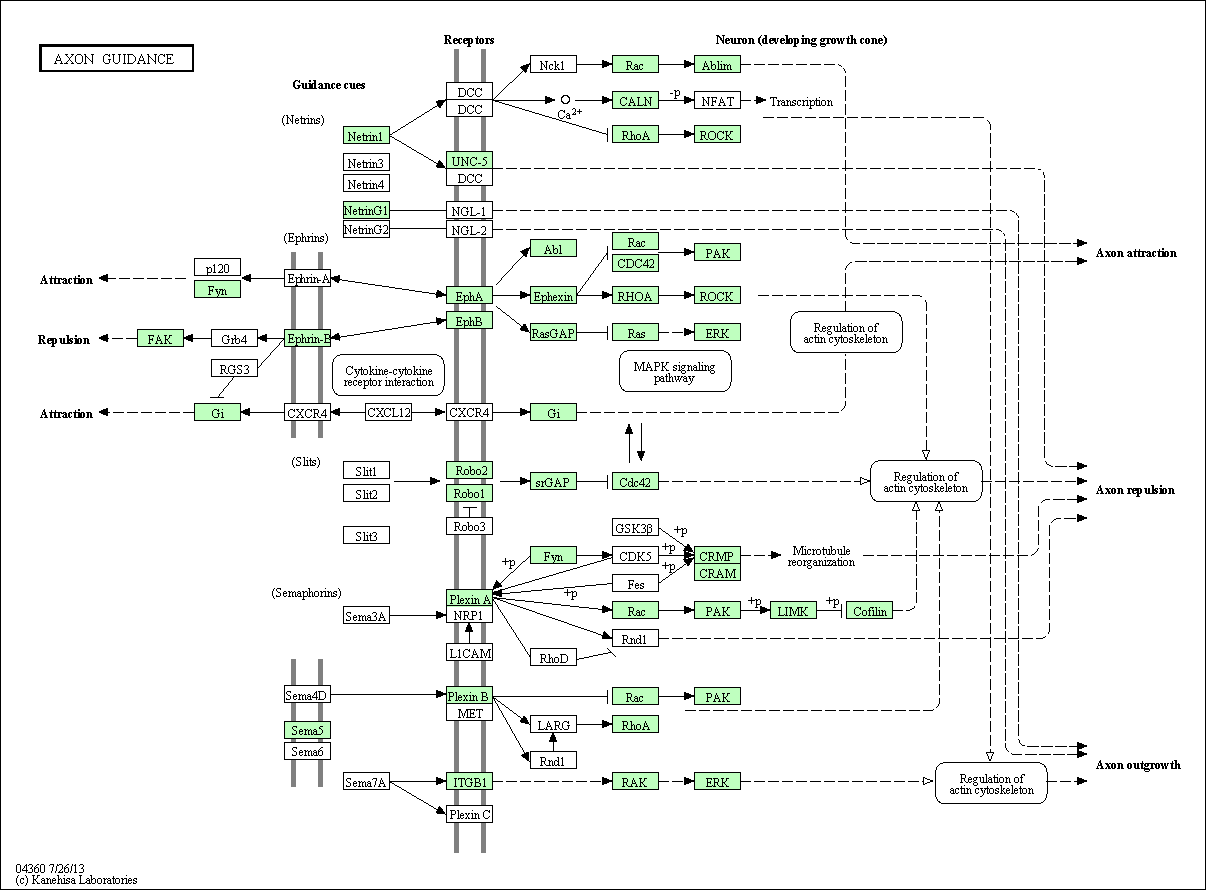

Supplement: Supplemental Information 9 [file peerj-04-1616-s009.gz › map/map04360.png]

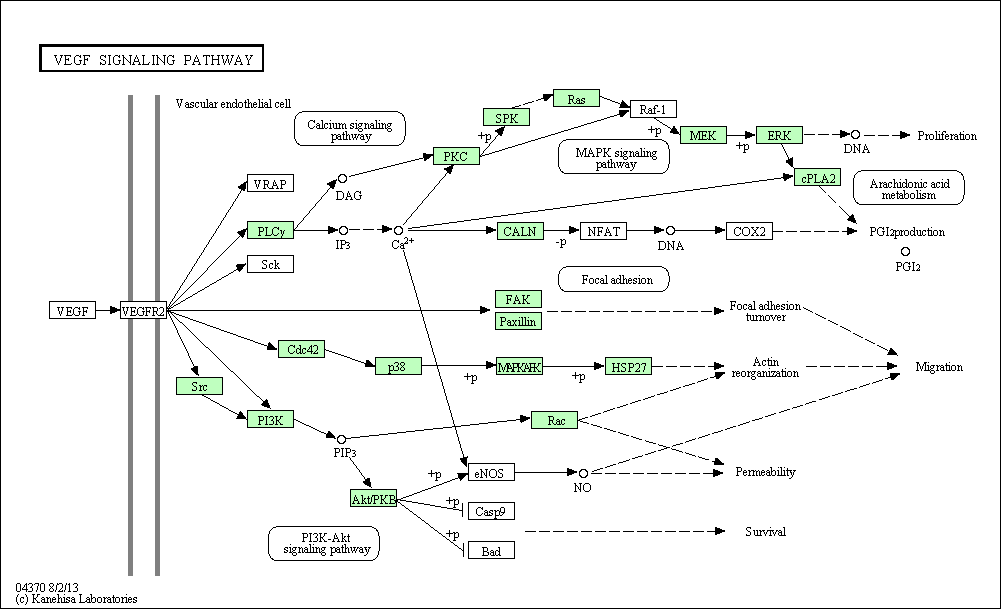

Supplement: Supplemental Information 9 [file peerj-04-1616-s009.gz › map/map04370.png]

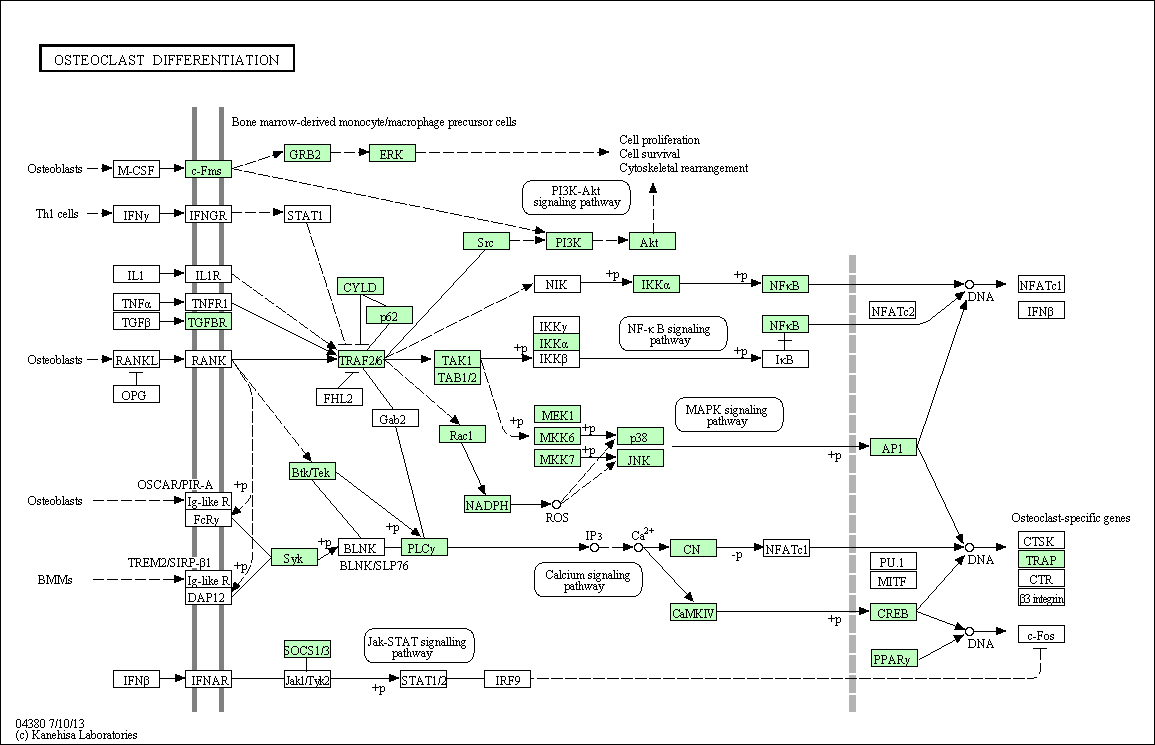

Supplement: Supplemental Information 9 [file peerj-04-1616-s009.gz › map/map04380.png]

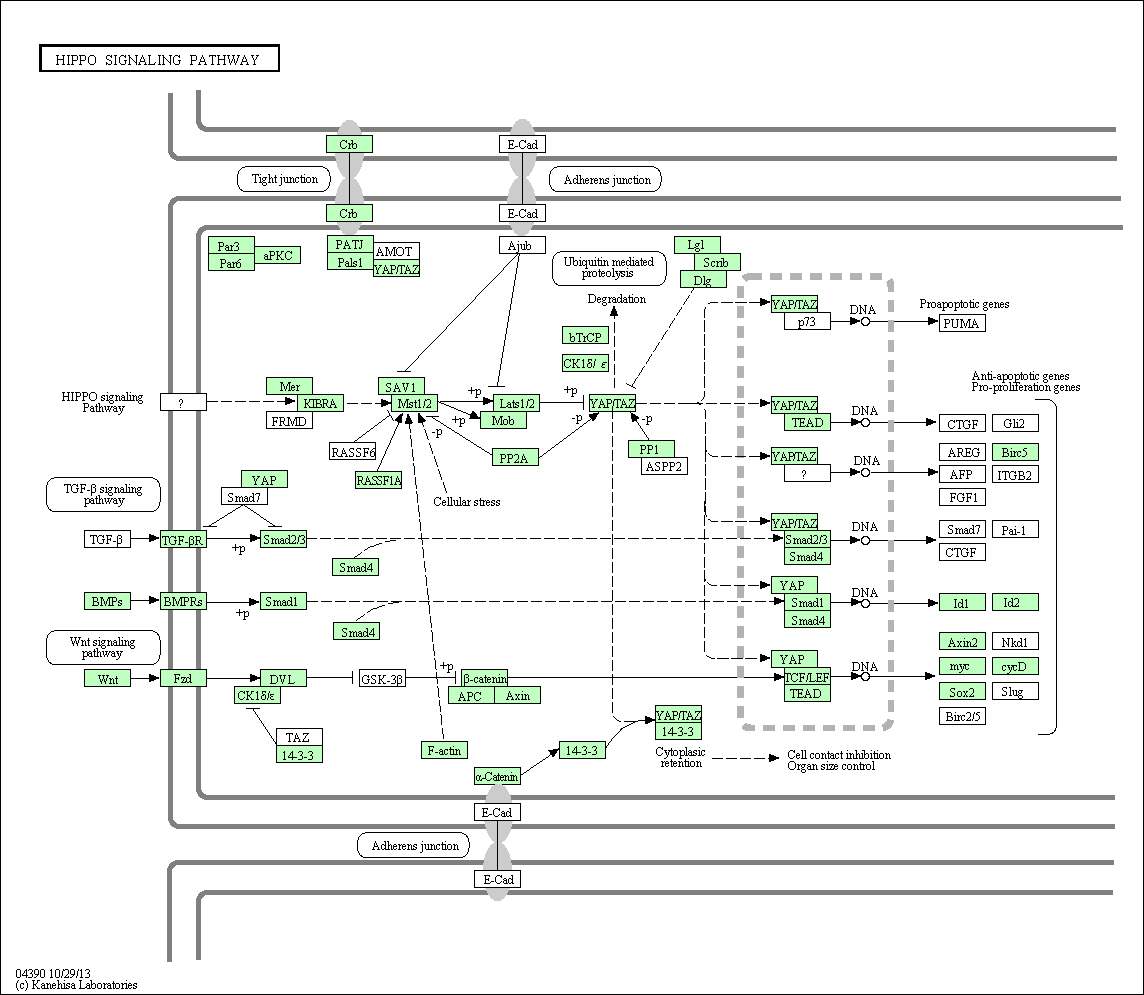

Supplement: Supplemental Information 9 [file peerj-04-1616-s009.gz › map/map04390.png]

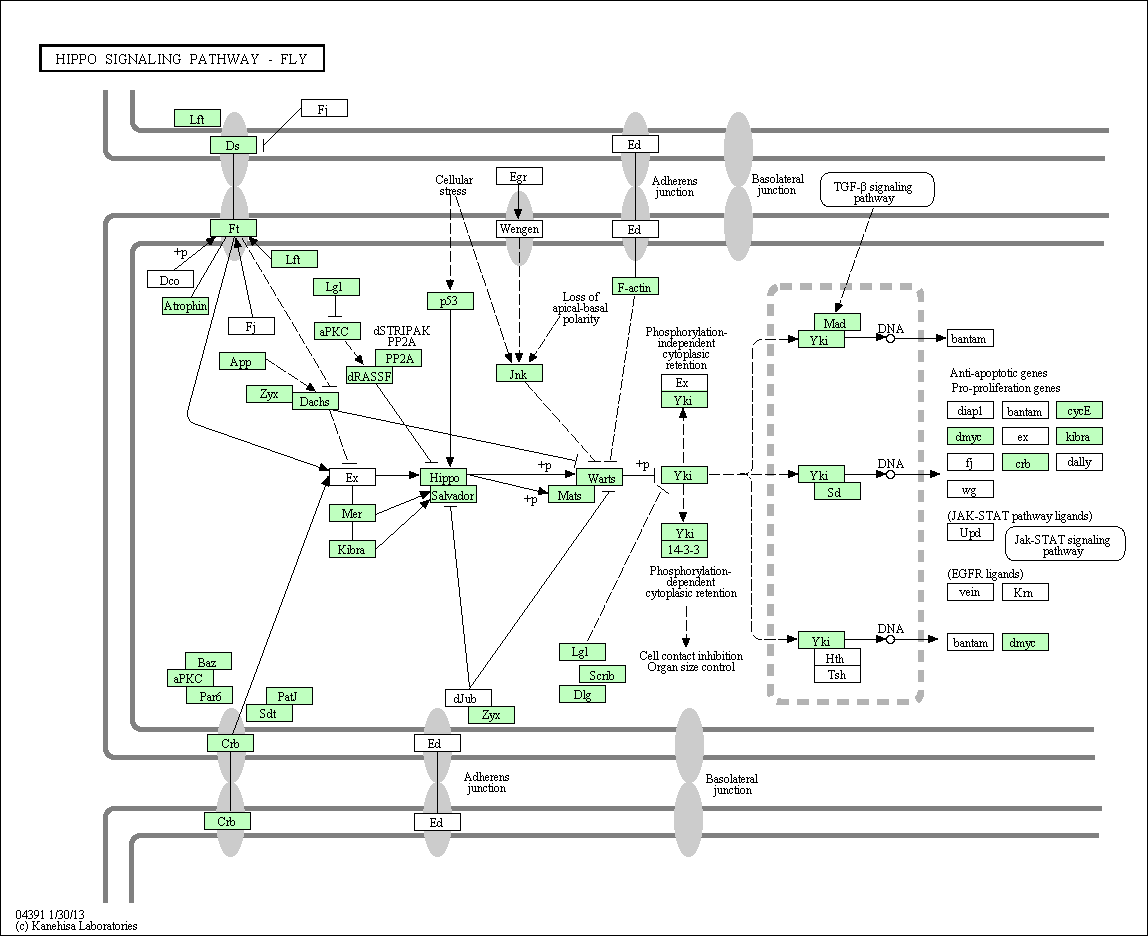

Supplement: Supplemental Information 9 [file peerj-04-1616-s009.gz › map/map04391.png]

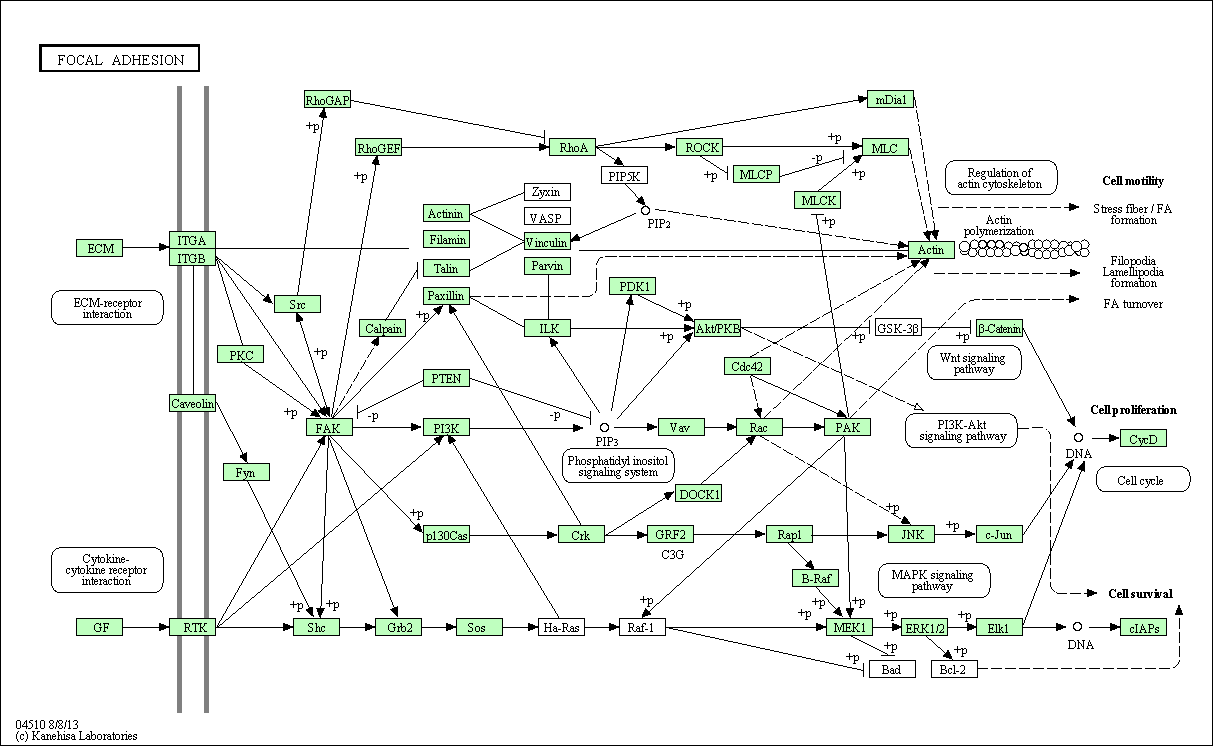

Supplement: Supplemental Information 9 [file peerj-04-1616-s009.gz › map/map04510.png]

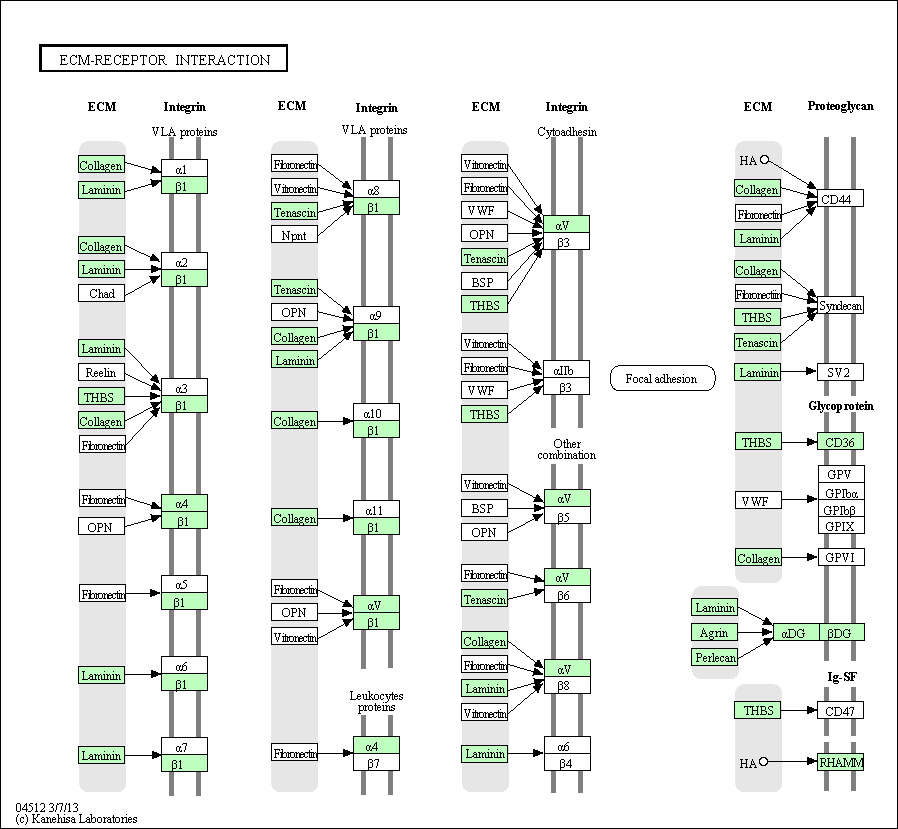

Supplement: Supplemental Information 9 [file peerj-04-1616-s009.gz › map/map04512.png]

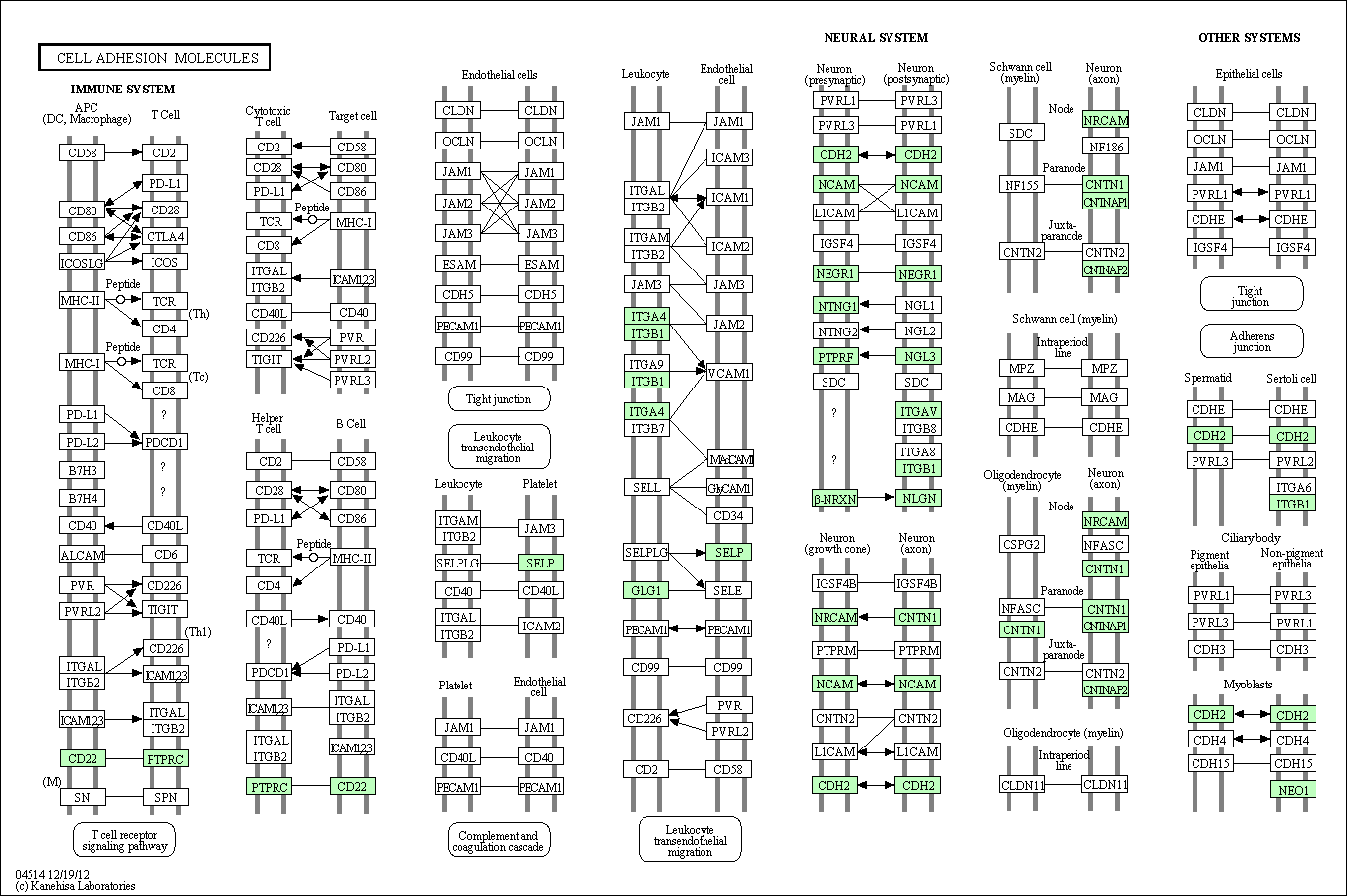

Supplement: Supplemental Information 9 [file peerj-04-1616-s009.gz › map/map04514.png]

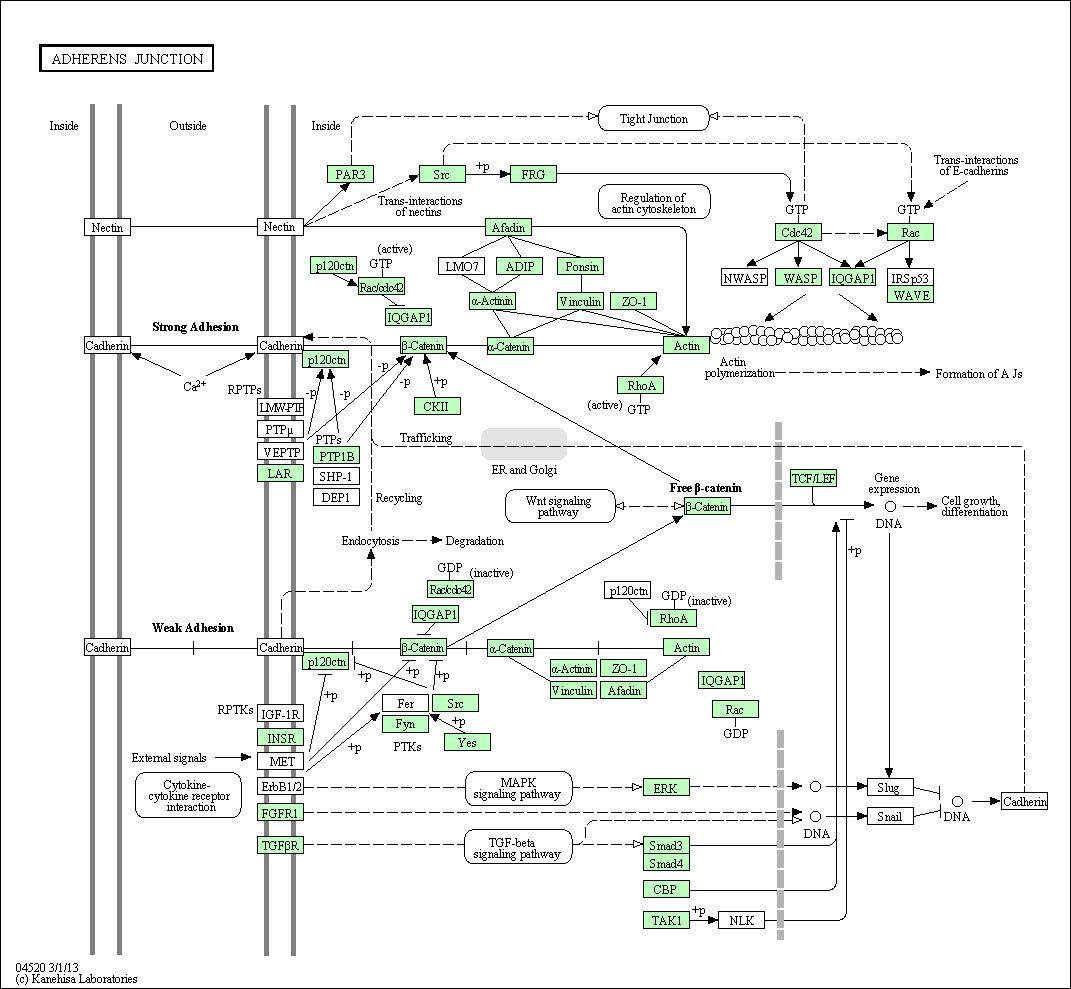

Supplement: Supplemental Information 9 [file peerj-04-1616-s009.gz › map/map04520.png]

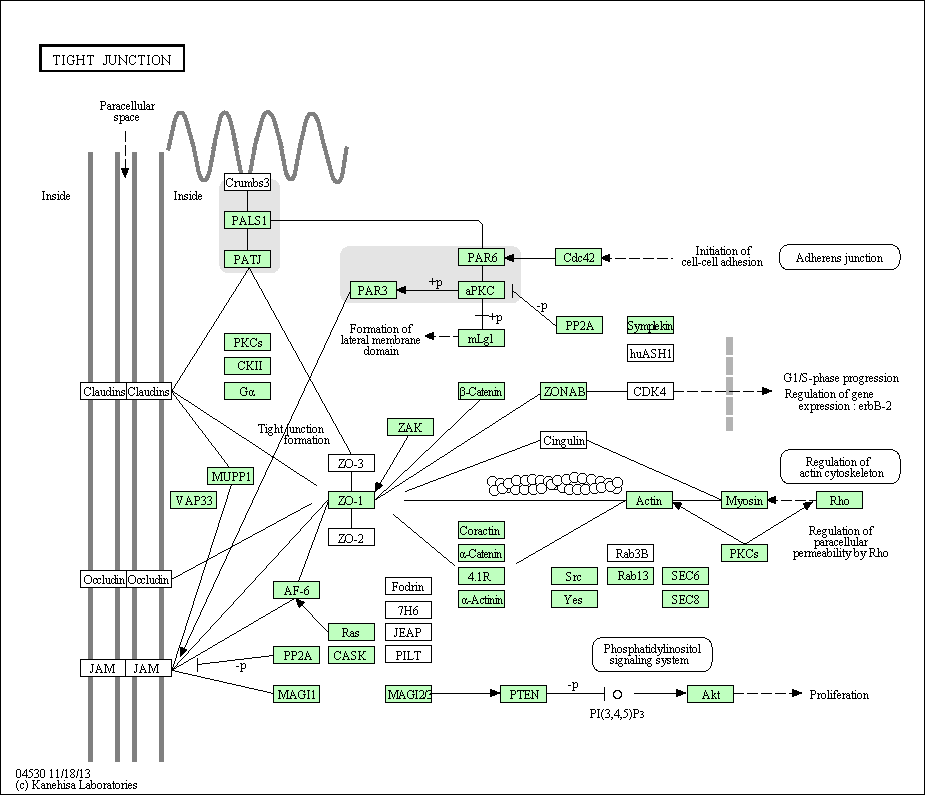

Supplement: Supplemental Information 9 [file peerj-04-1616-s009.gz › map/map04530.png]

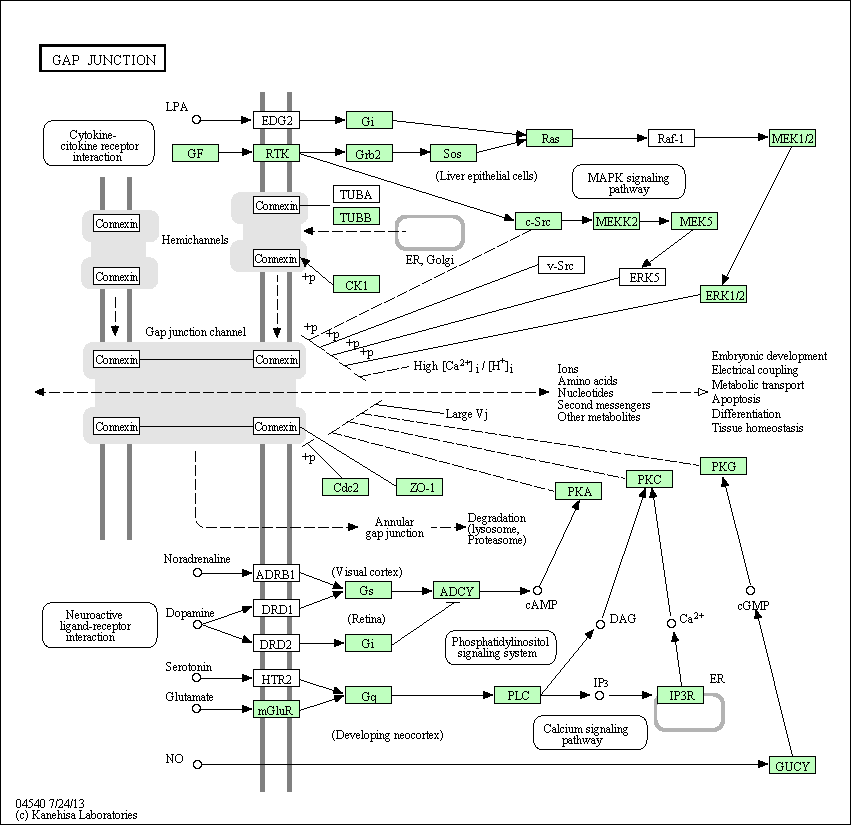

Supplement: Supplemental Information 9 [file peerj-04-1616-s009.gz › map/map04540.png]

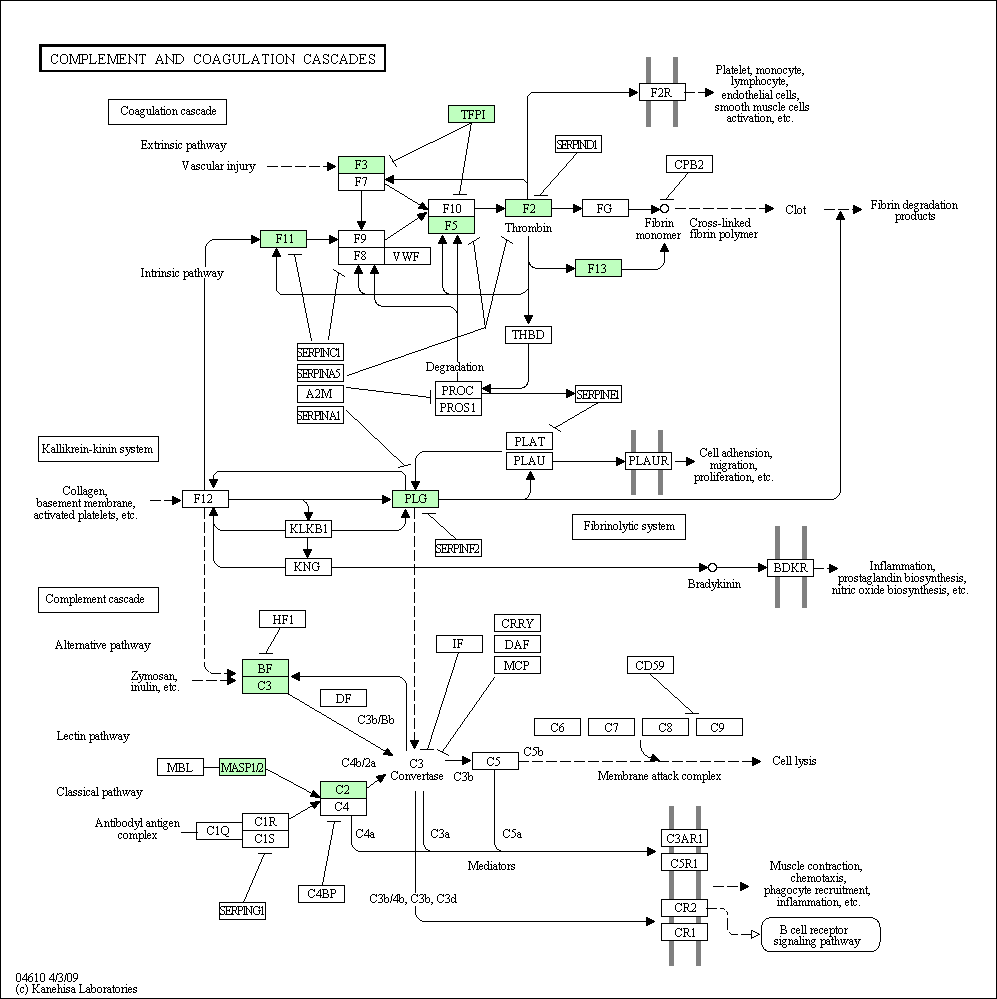

Supplement: Supplemental Information 9 [file peerj-04-1616-s009.gz › map/map04610.png]

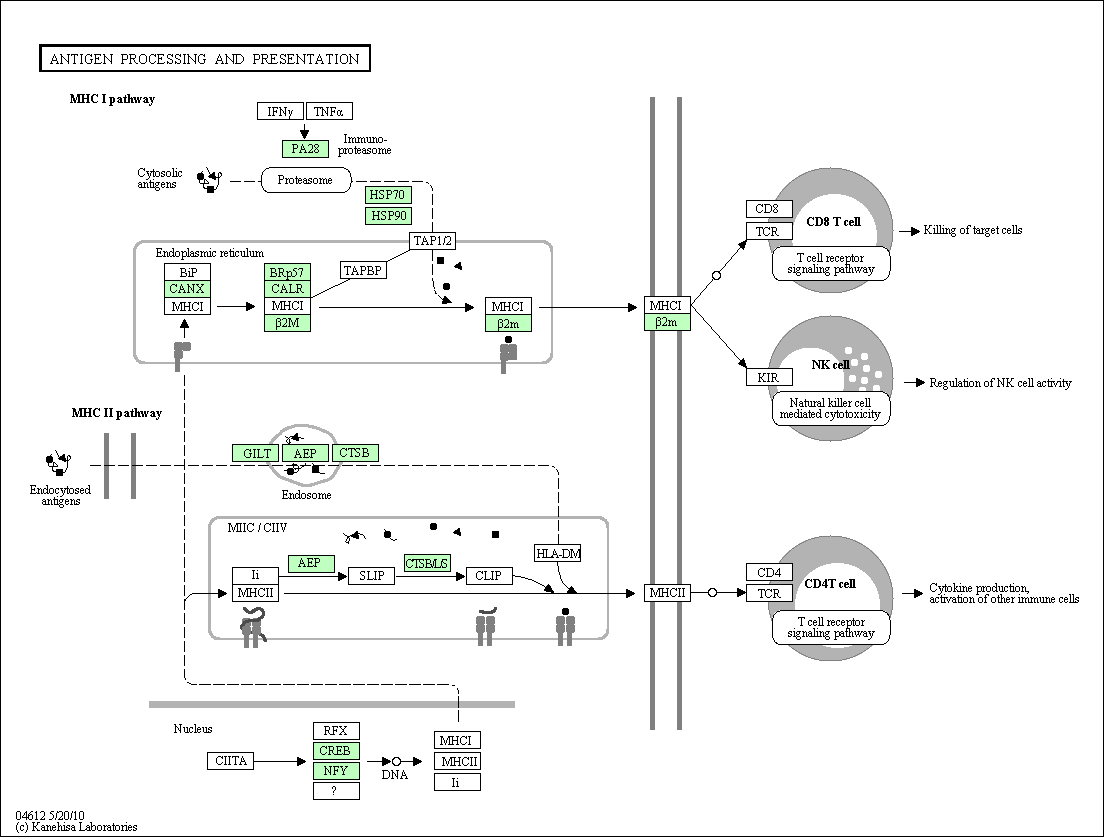

Supplement: Supplemental Information 9 [file peerj-04-1616-s009.gz › map/map04612.png]
