# Supplementary figures and images for: RNA-Seq of the Caribbean reef-building coral Orbicella faveolata (Scleractinia-Merulinidae) under bleaching and disease stress expands models of coral innate immunity (part 1 of 2)
Source: PeerJ. 2016 Feb 15;4:e1616. doi: 10.7717/peerj.1616 (PMC4768675; doi:10.7717/peerj.1616)

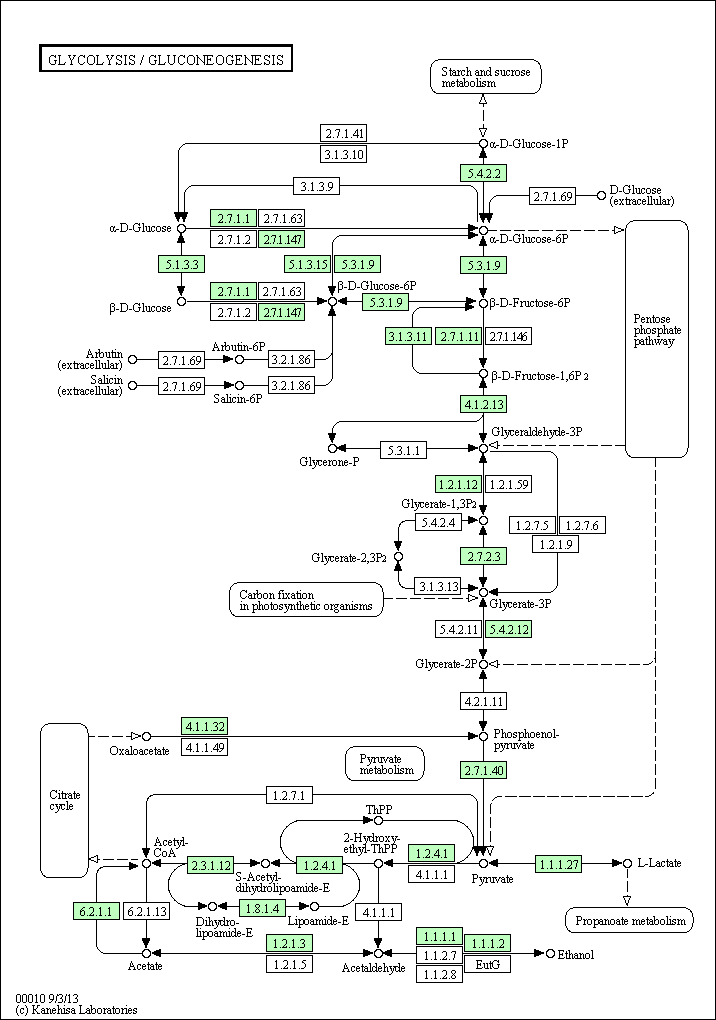

Supplement: Supplemental Information 9 [file peerj-04-1616-s009.gz › map/map00010.png]

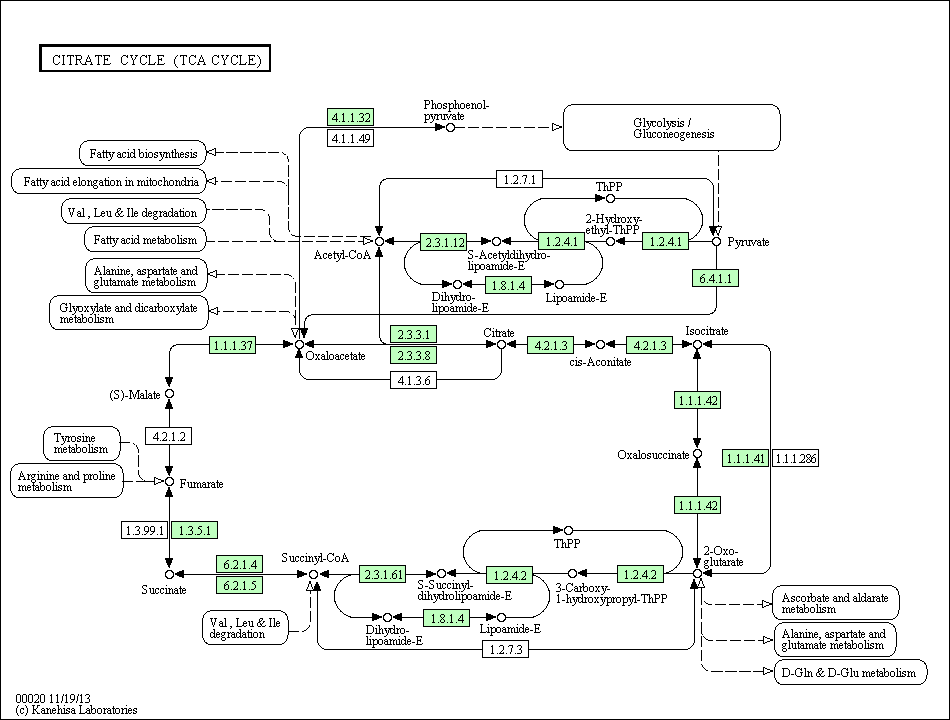

Supplement: Supplemental Information 9 [file peerj-04-1616-s009.gz › map/map00020.png]

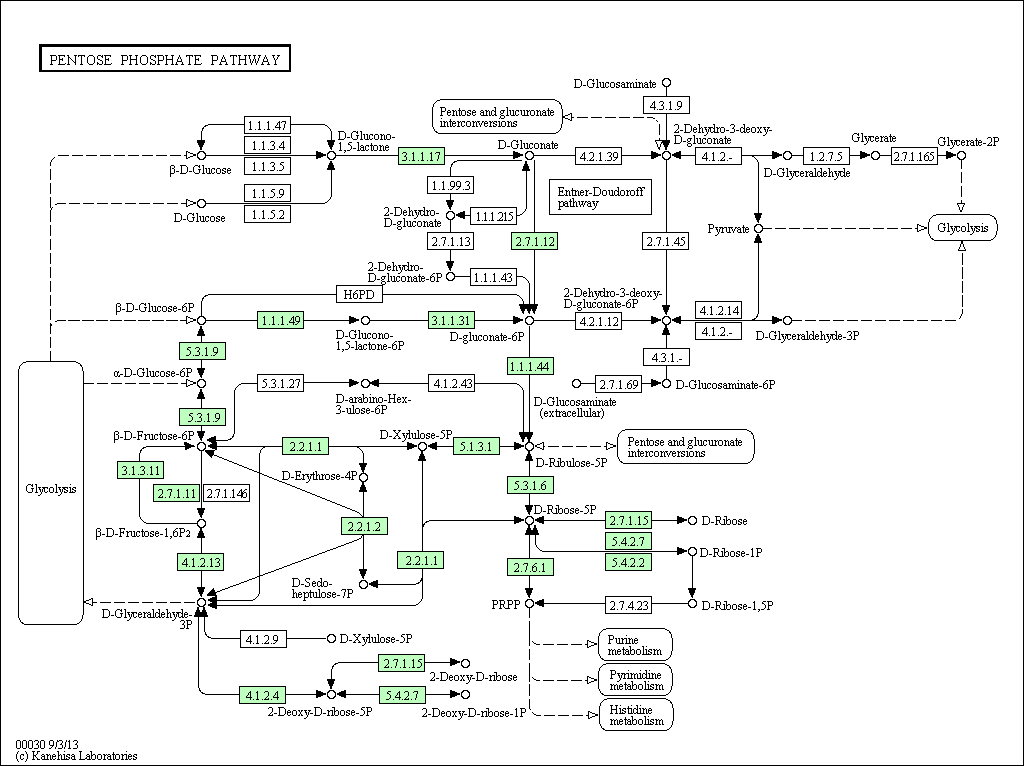

Supplement: Supplemental Information 9 [file peerj-04-1616-s009.gz › map/map00030.png]

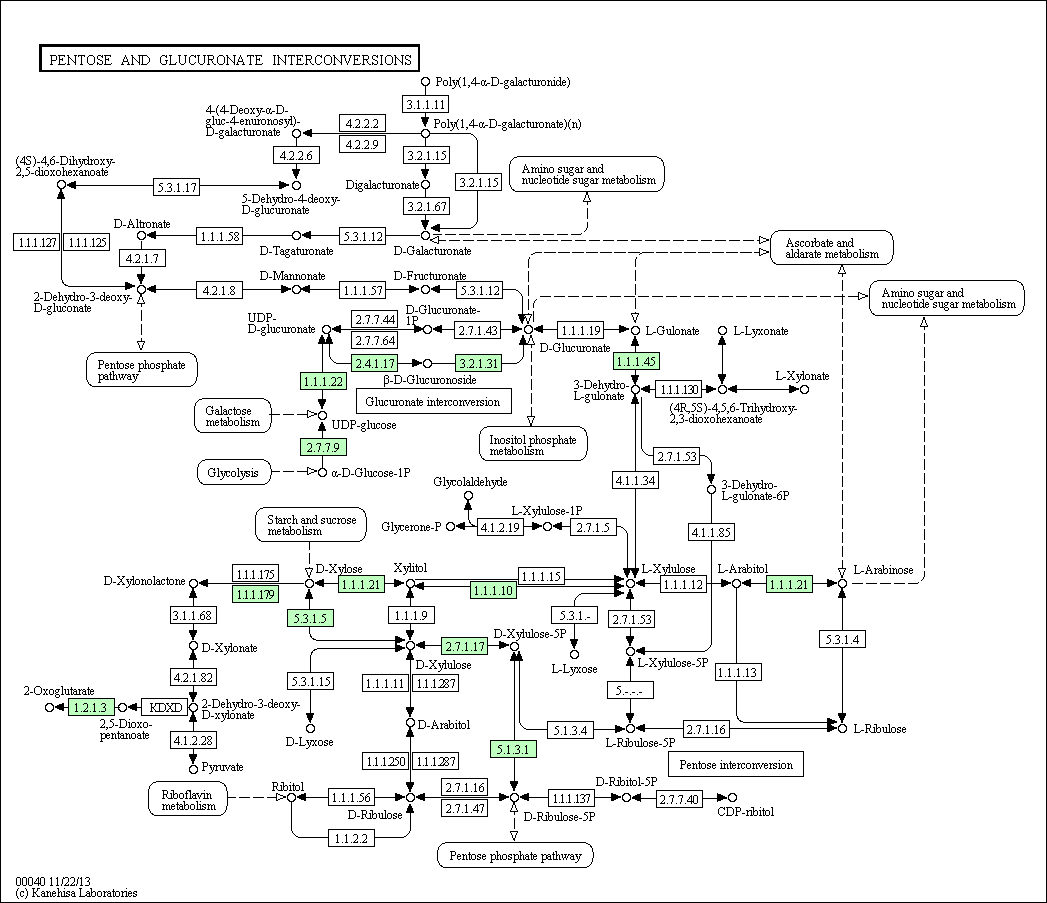

Supplement: Supplemental Information 9 [file peerj-04-1616-s009.gz › map/map00040.png]

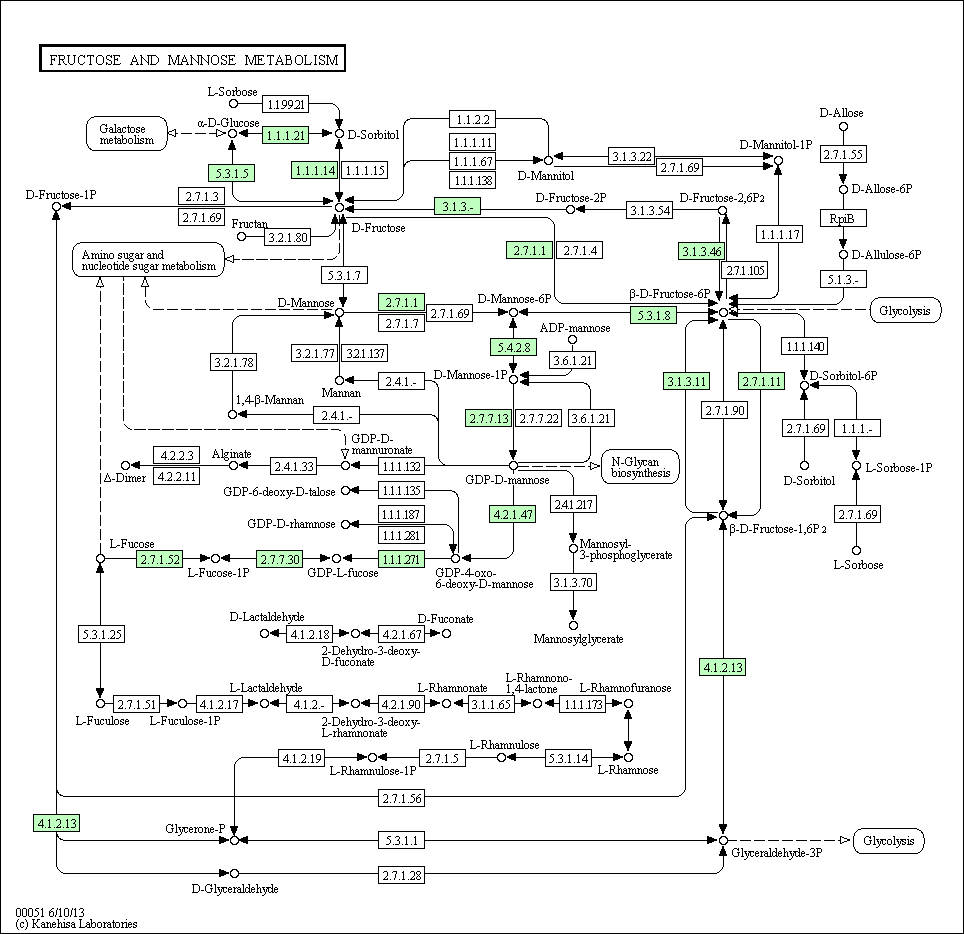

Supplement: Supplemental Information 9 [file peerj-04-1616-s009.gz › map/map00051.png]

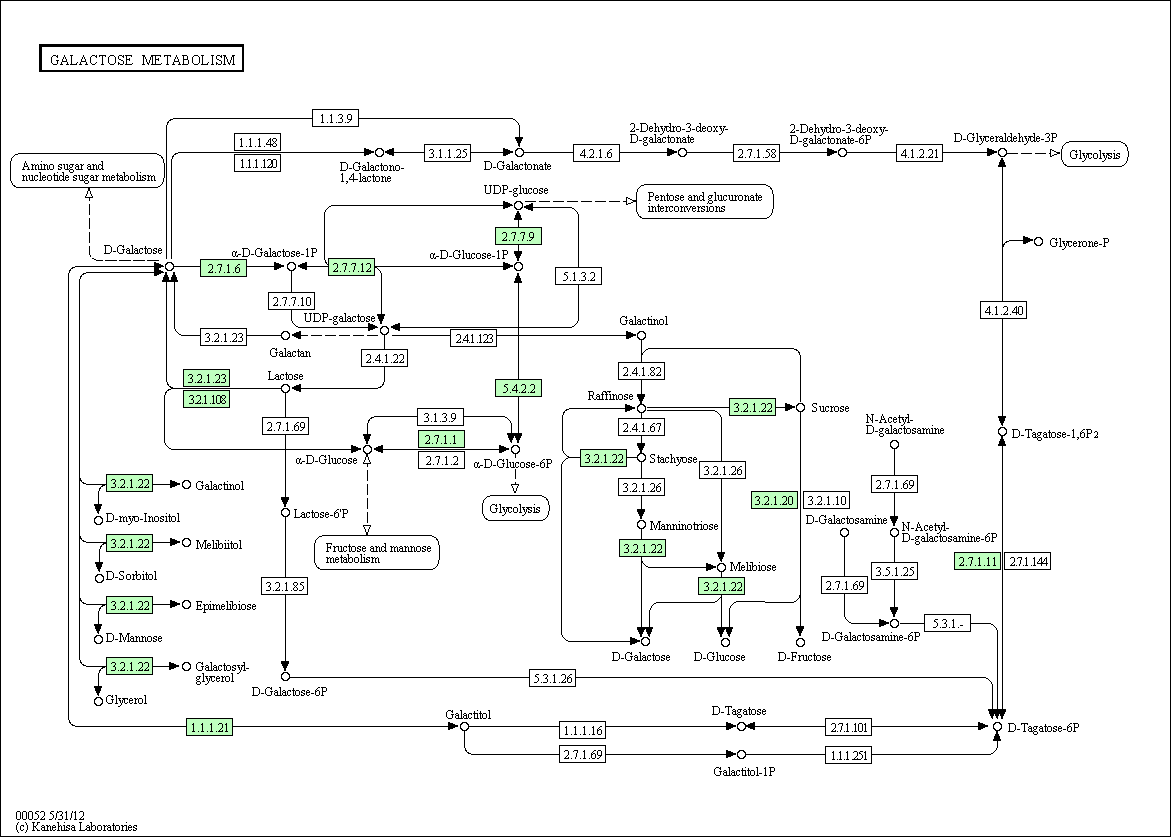

Supplement: Supplemental Information 9 [file peerj-04-1616-s009.gz › map/map00052.png]

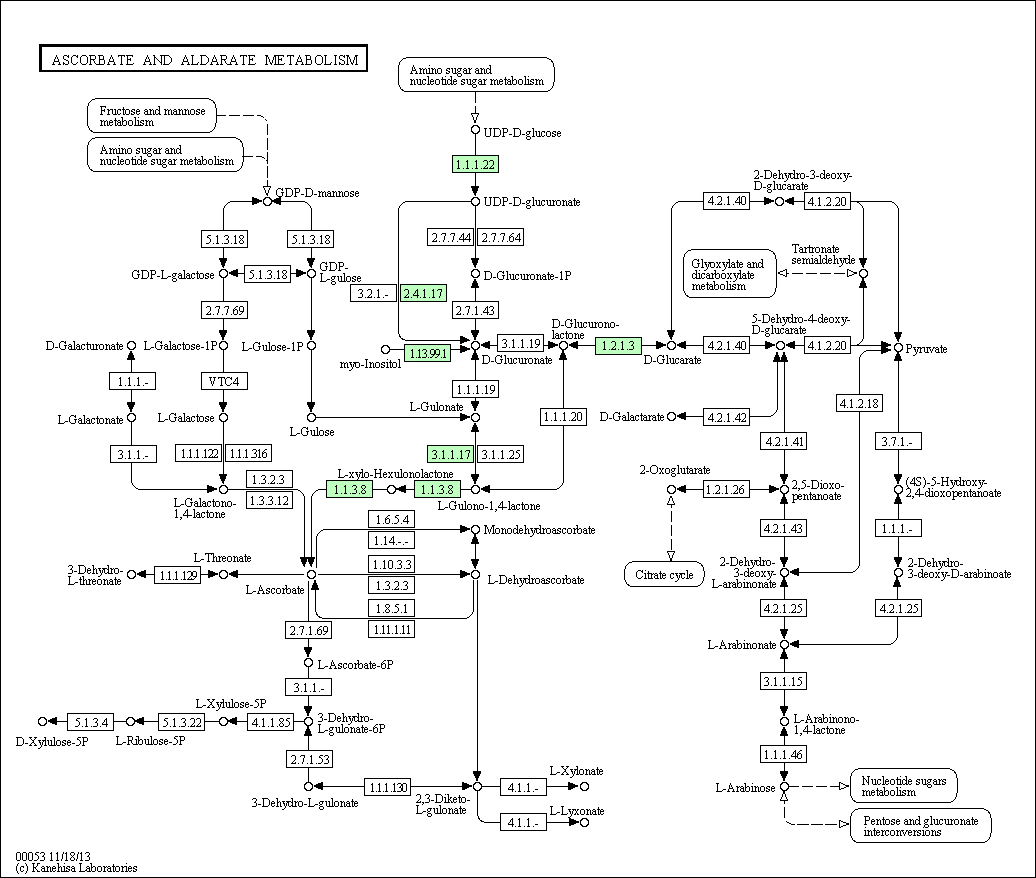

Supplement: Supplemental Information 9 [file peerj-04-1616-s009.gz › map/map00053.png]

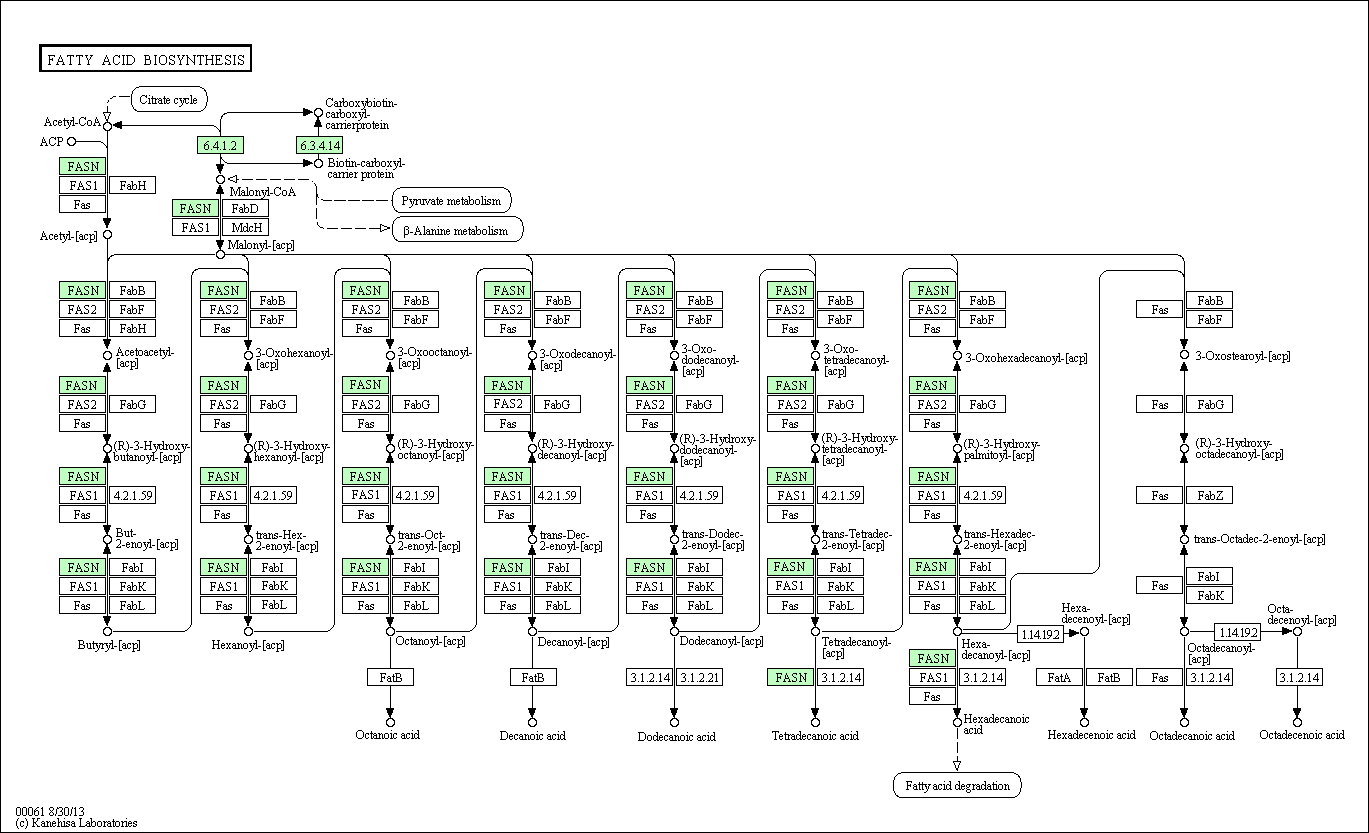

Supplement: Supplemental Information 9 [file peerj-04-1616-s009.gz › map/map00061.png]

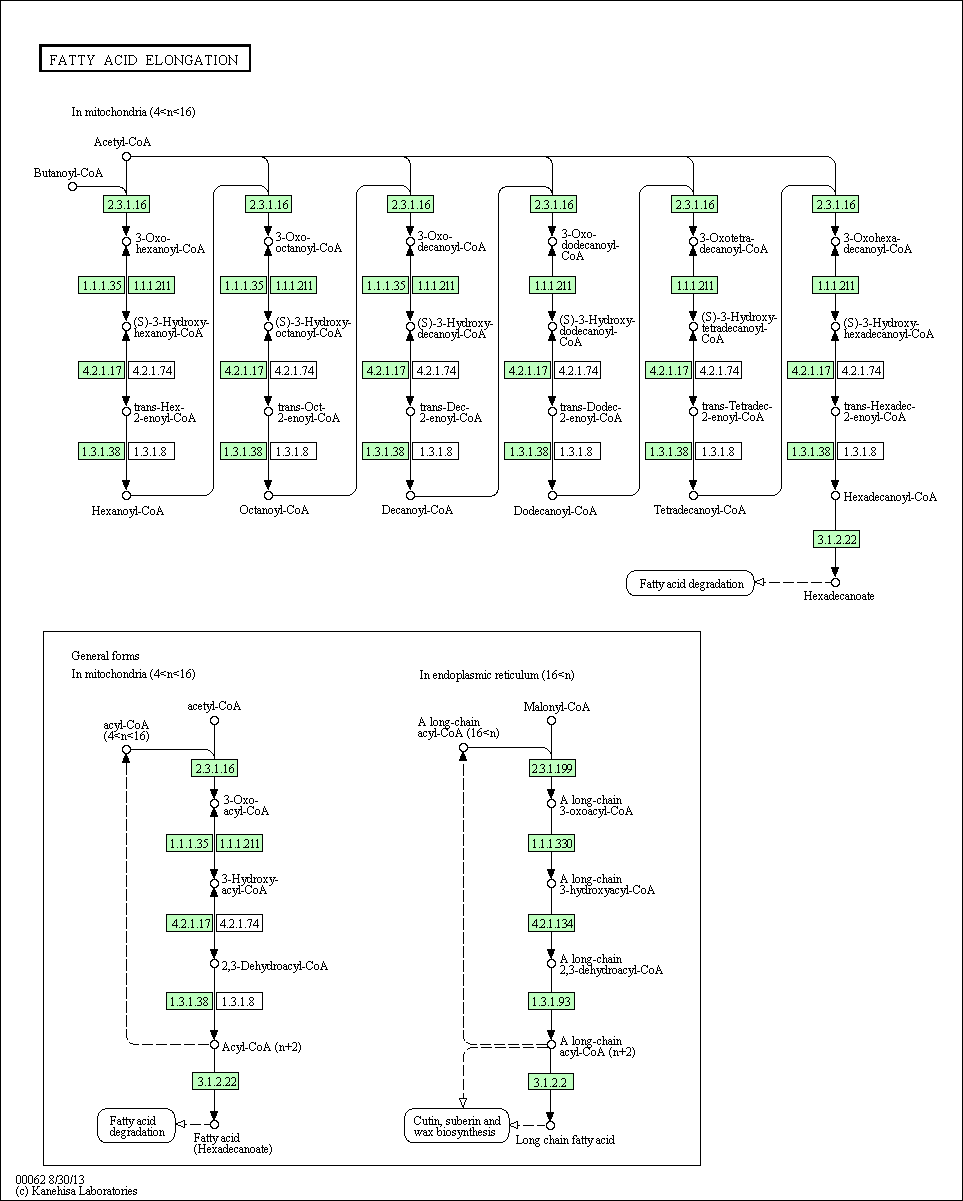

Supplement: Supplemental Information 9 [file peerj-04-1616-s009.gz › map/map00062.png]

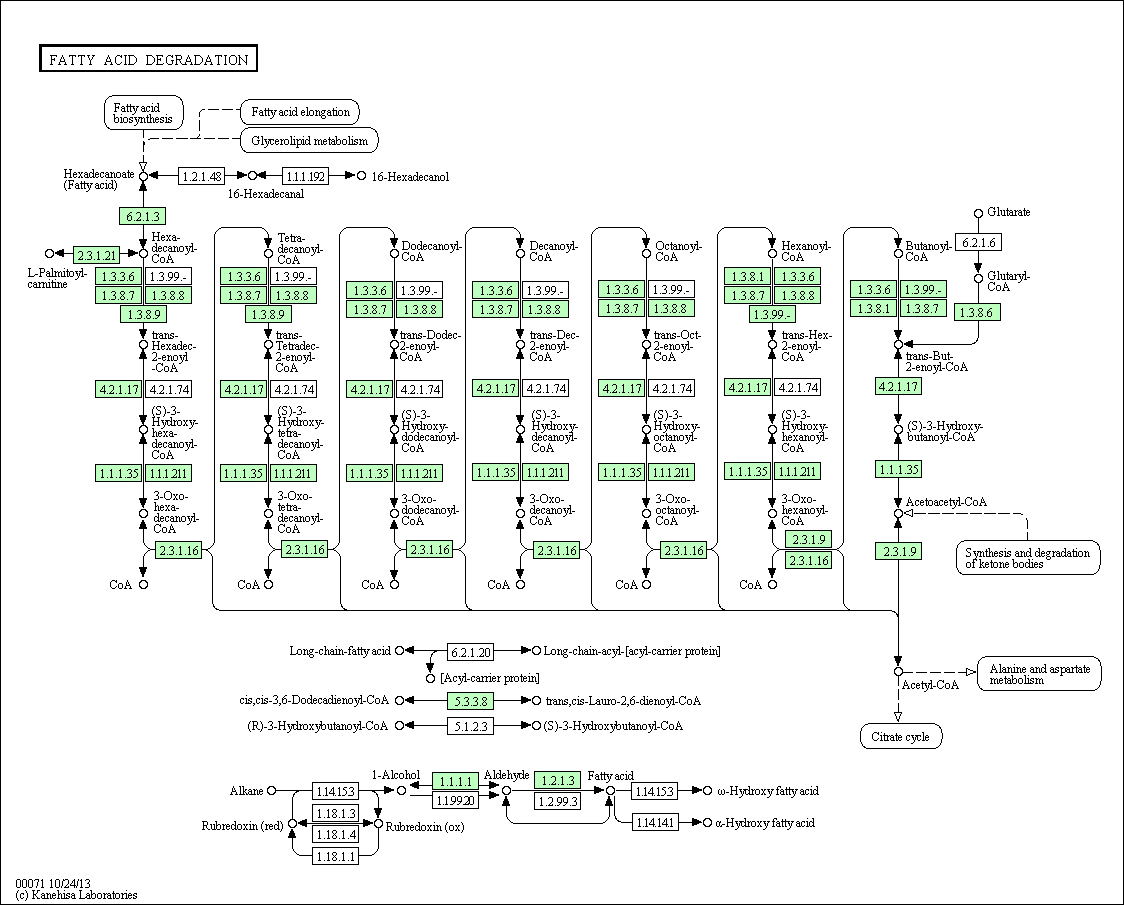

Supplement: Supplemental Information 9 [file peerj-04-1616-s009.gz › map/map00071.png]

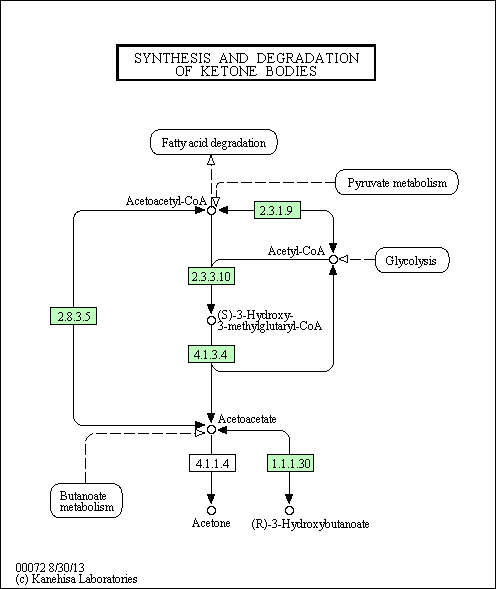

Supplement: Supplemental Information 9 [file peerj-04-1616-s009.gz › map/map00072.png]

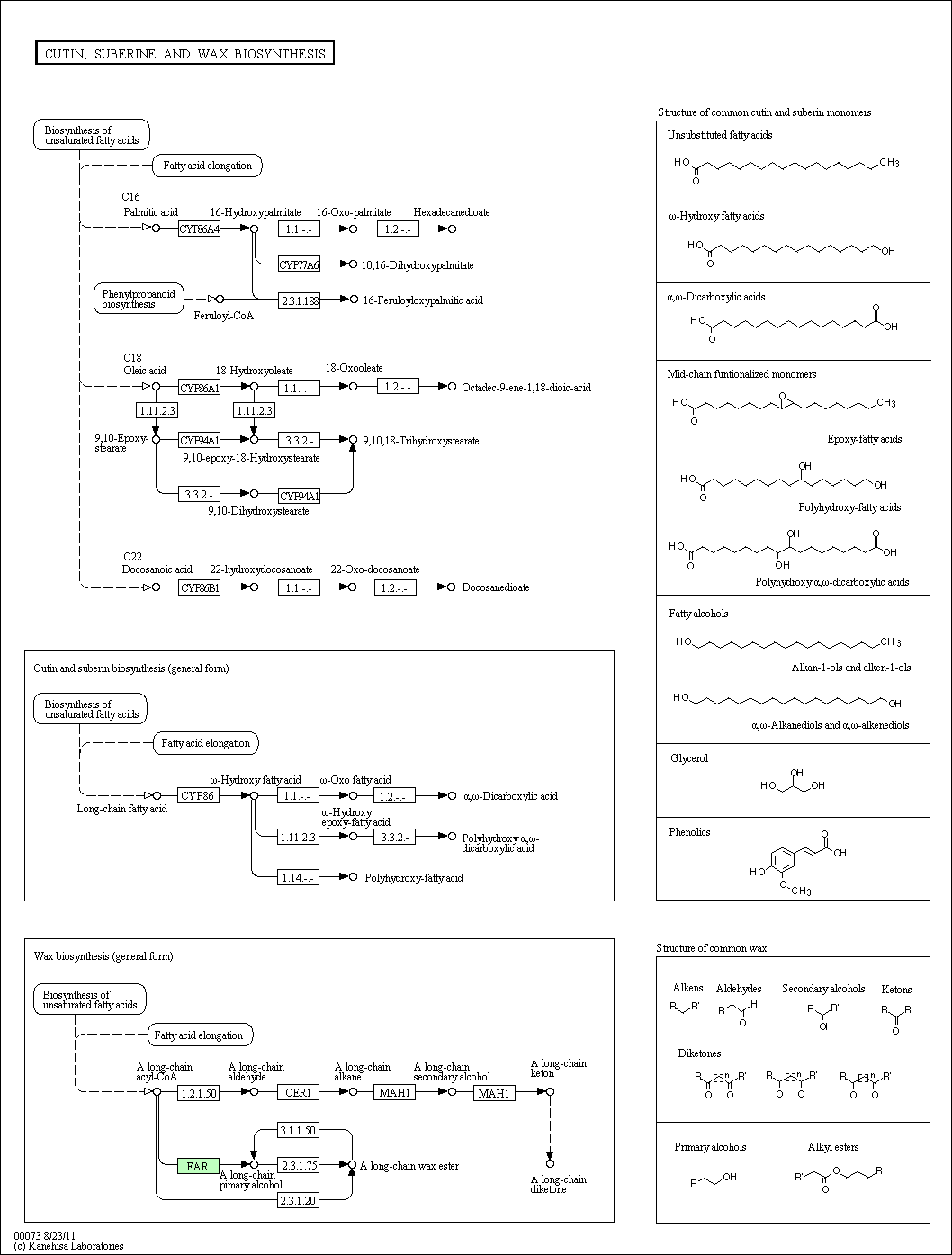

Supplement: Supplemental Information 9 [file peerj-04-1616-s009.gz › map/map00073.png]

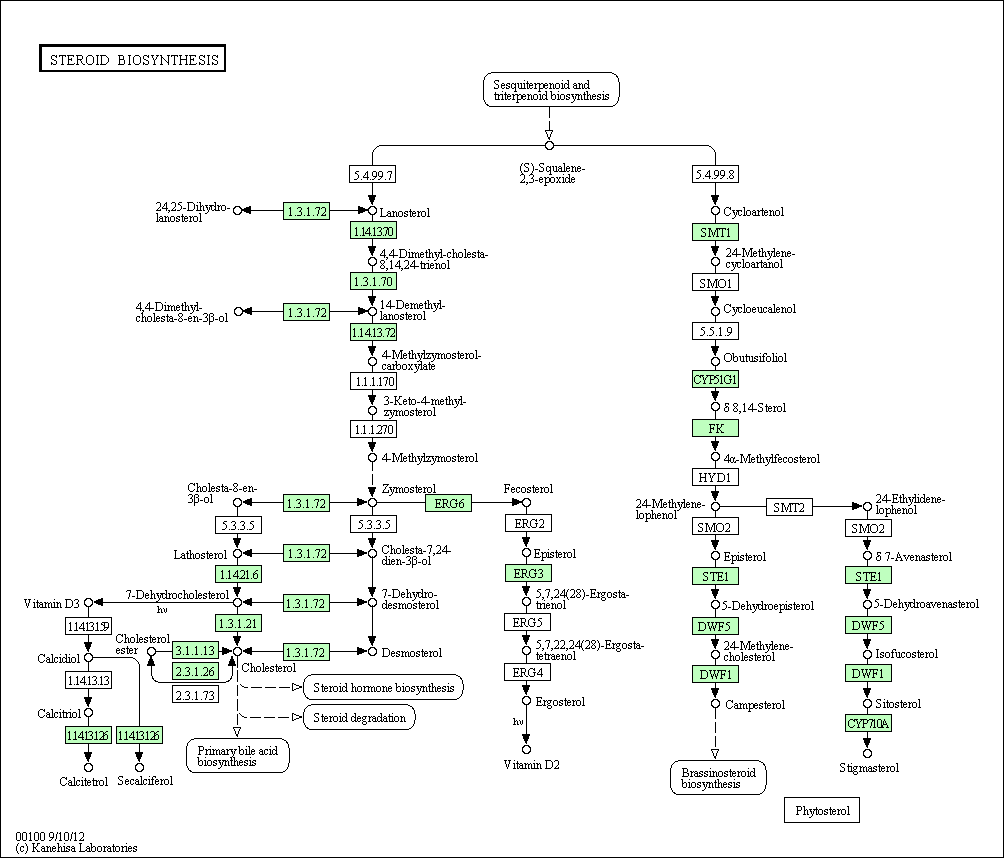

Supplement: Supplemental Information 9 [file peerj-04-1616-s009.gz › map/map00100.png]

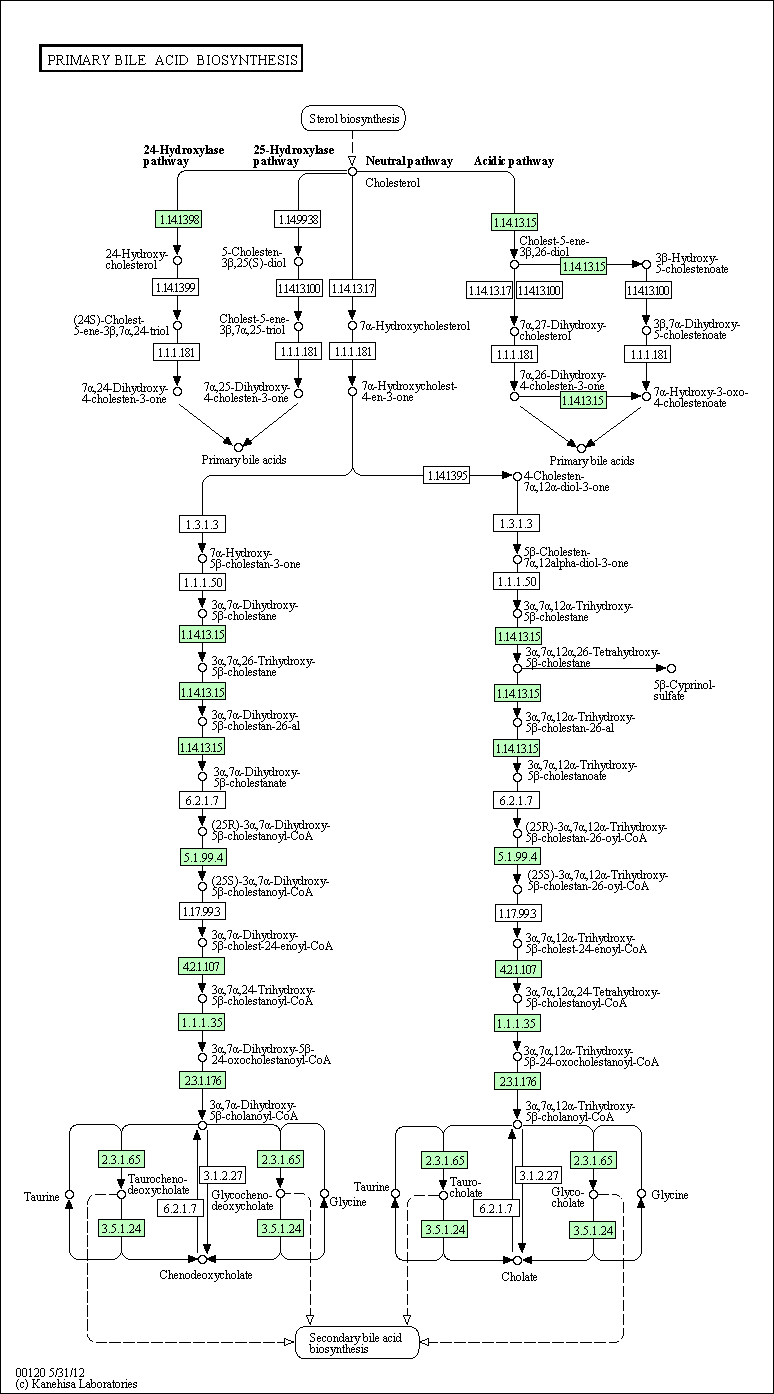

Supplement: Supplemental Information 9 [file peerj-04-1616-s009.gz › map/map00120.png]

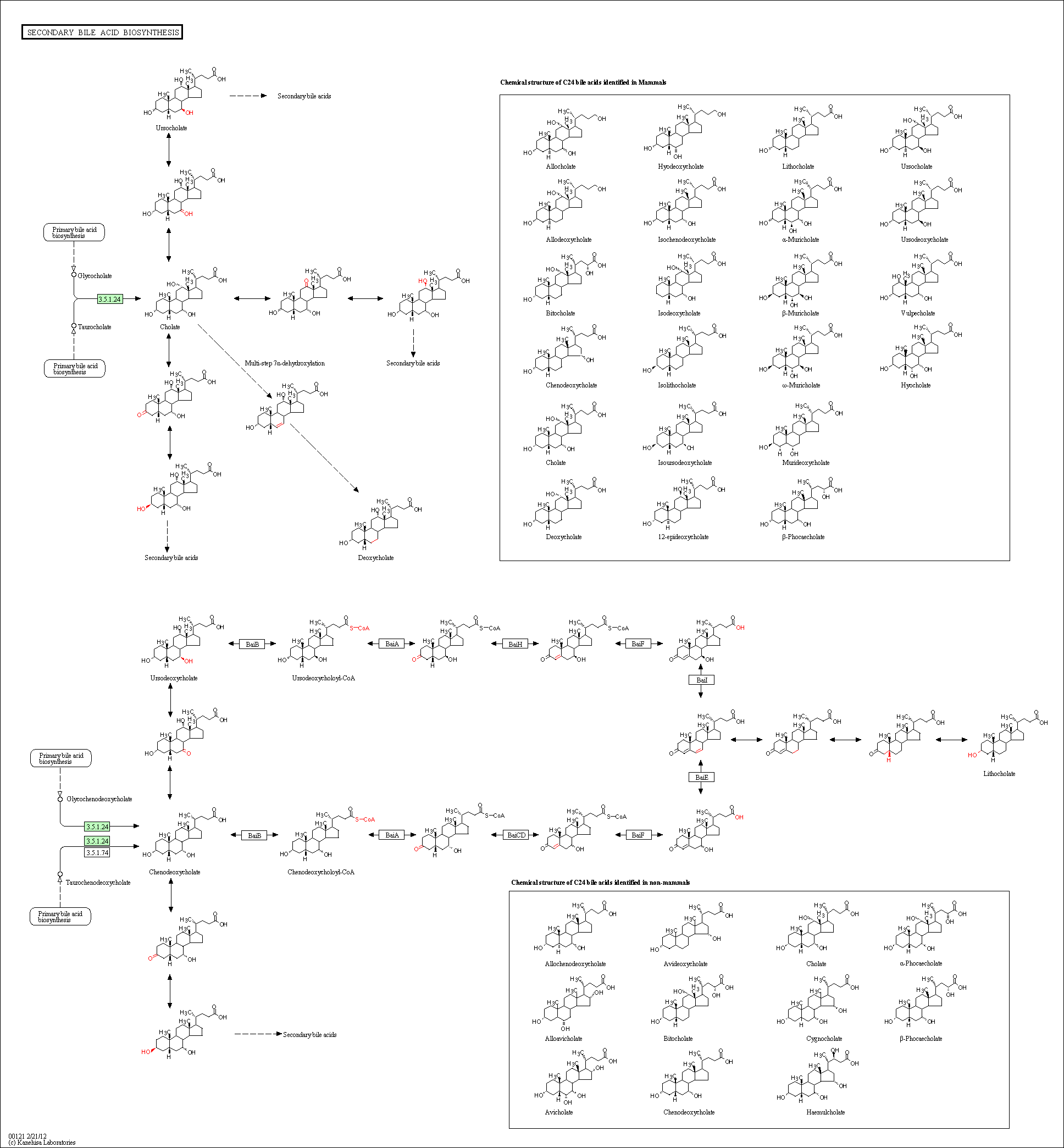

Supplement: Supplemental Information 9 [file peerj-04-1616-s009.gz › map/map00121.png]

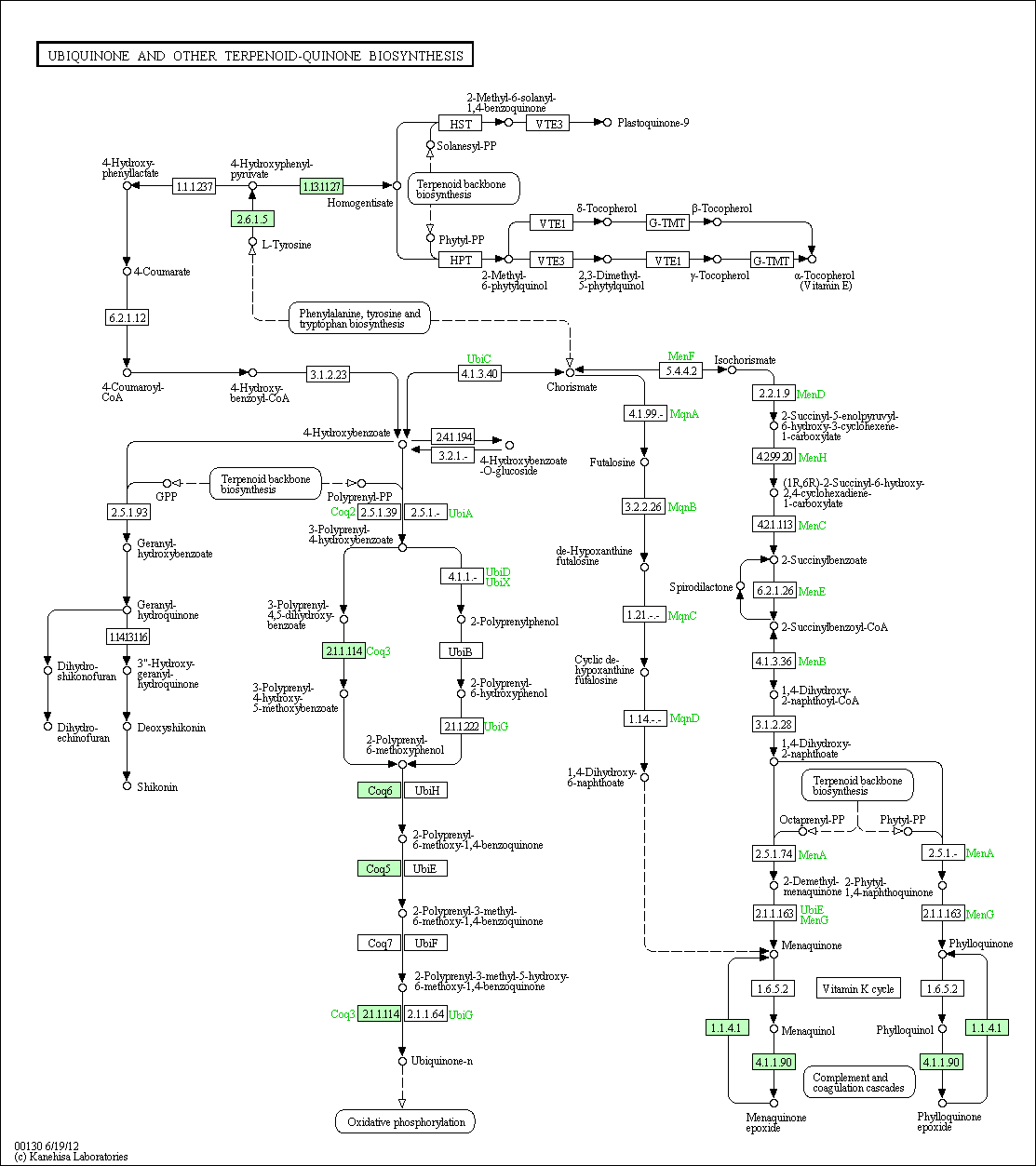

Supplement: Supplemental Information 9 [file peerj-04-1616-s009.gz › map/map00130.png]

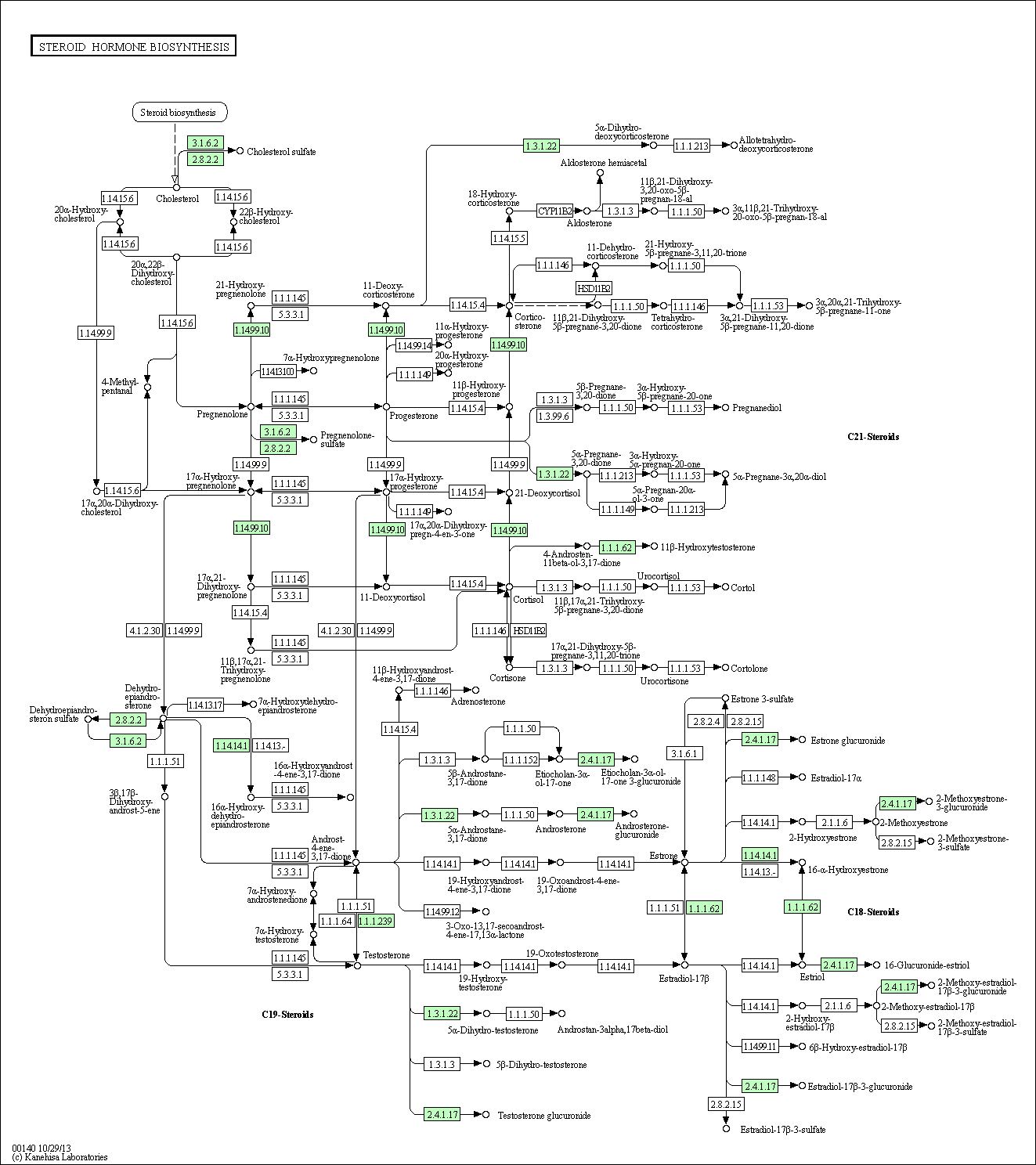

Supplement: Supplemental Information 9 [file peerj-04-1616-s009.gz › map/map00140.png]

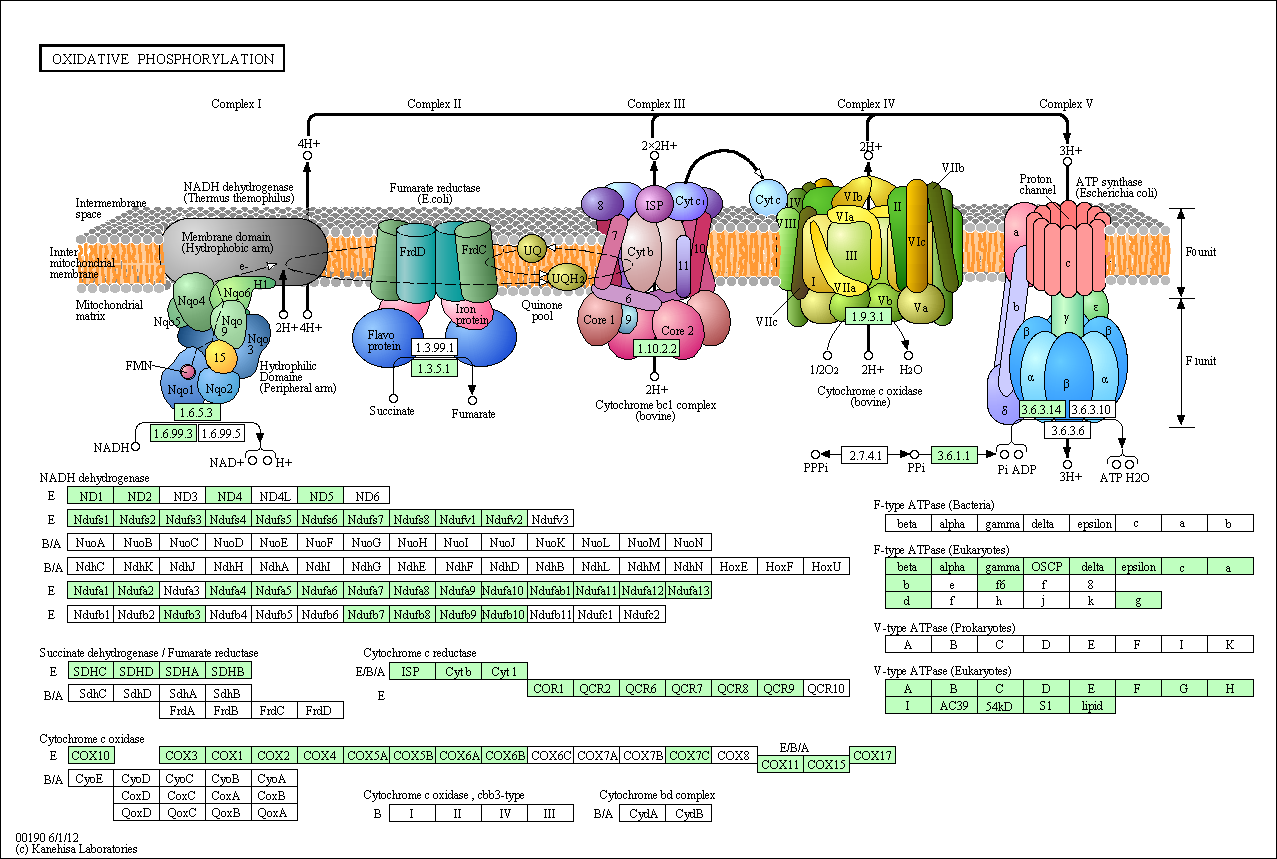

Supplement: Supplemental Information 9 [file peerj-04-1616-s009.gz › map/map00190.png]

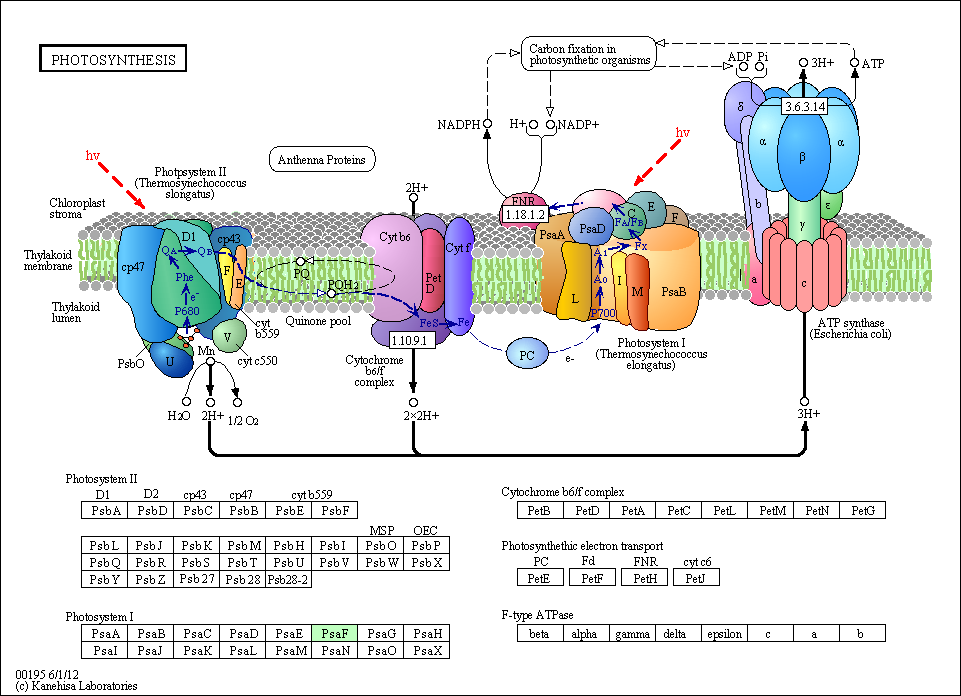

Supplement: Supplemental Information 9 [file peerj-04-1616-s009.gz › map/map00195.png]

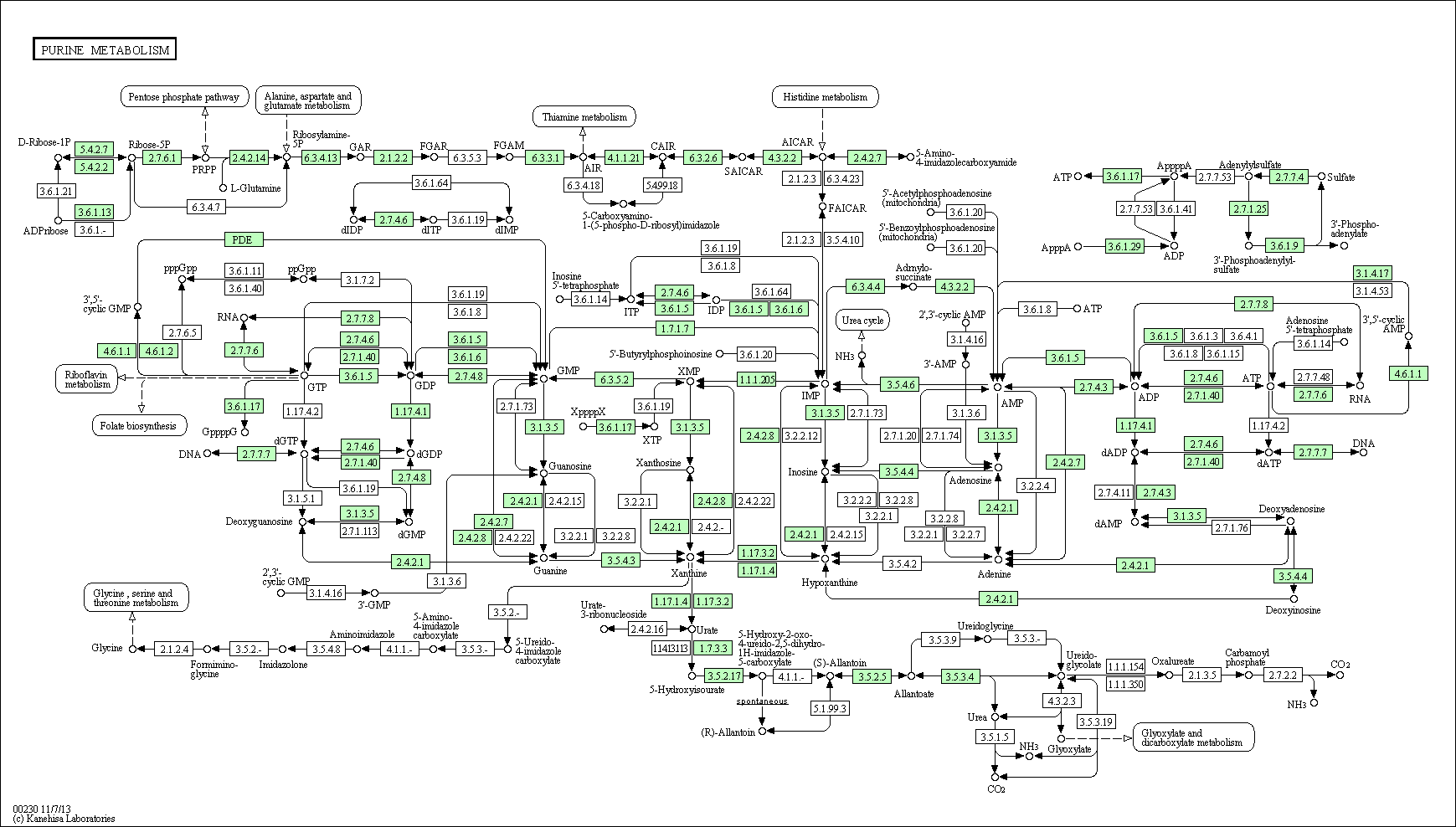

Supplement: Supplemental Information 9 [file peerj-04-1616-s009.gz › map/map00230.png]

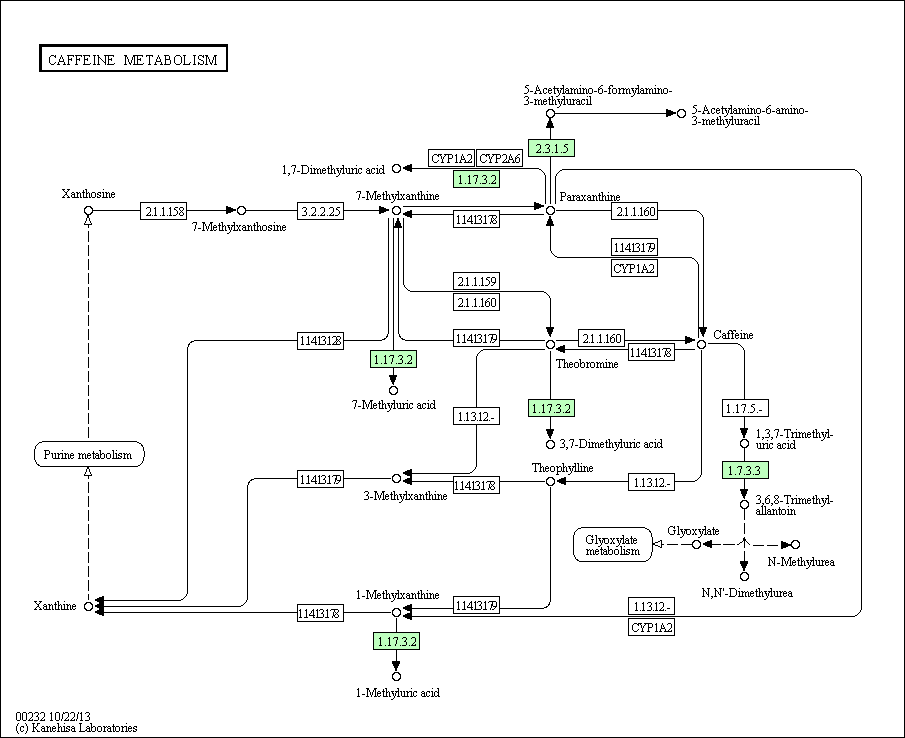

Supplement: Supplemental Information 9 [file peerj-04-1616-s009.gz › map/map00232.png]

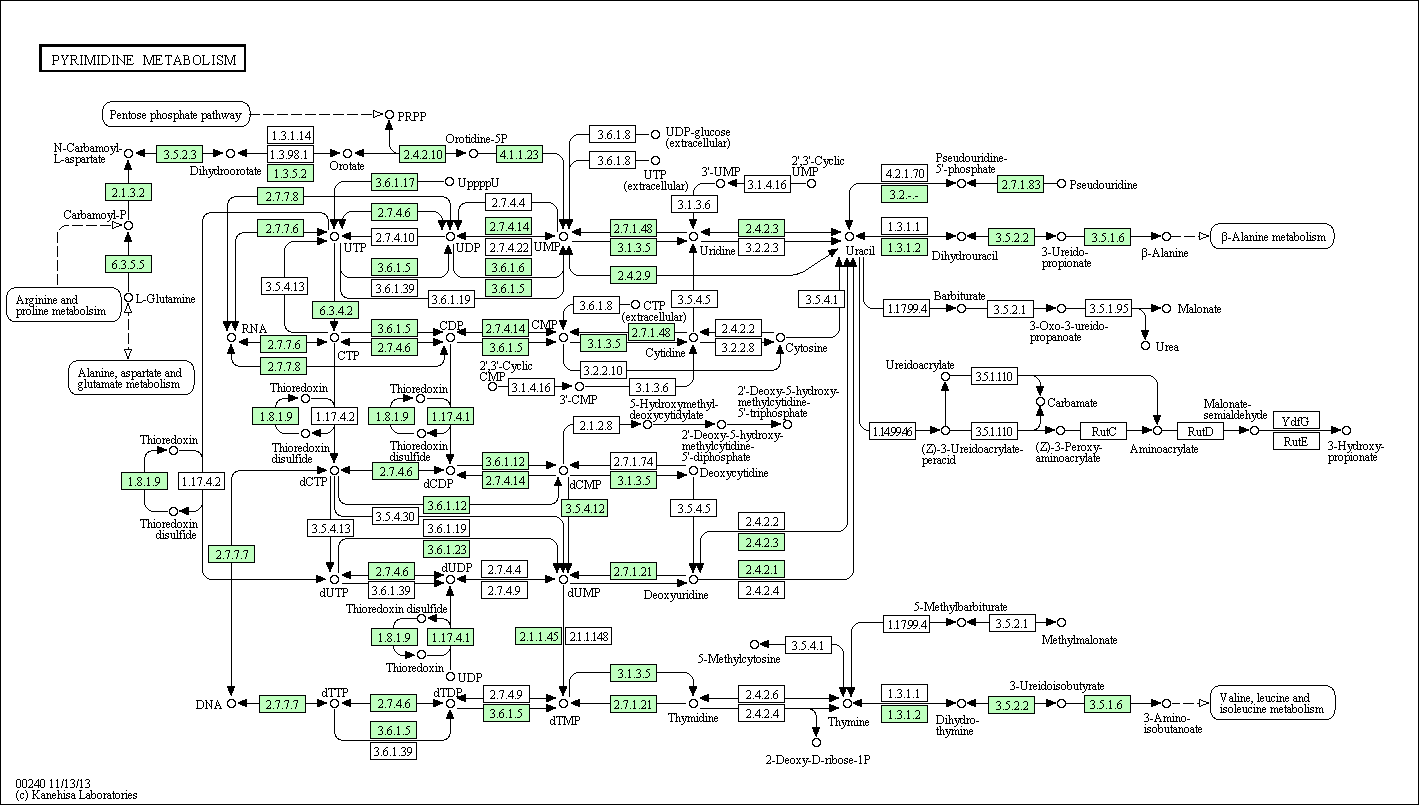

Supplement: Supplemental Information 9 [file peerj-04-1616-s009.gz › map/map00240.png]

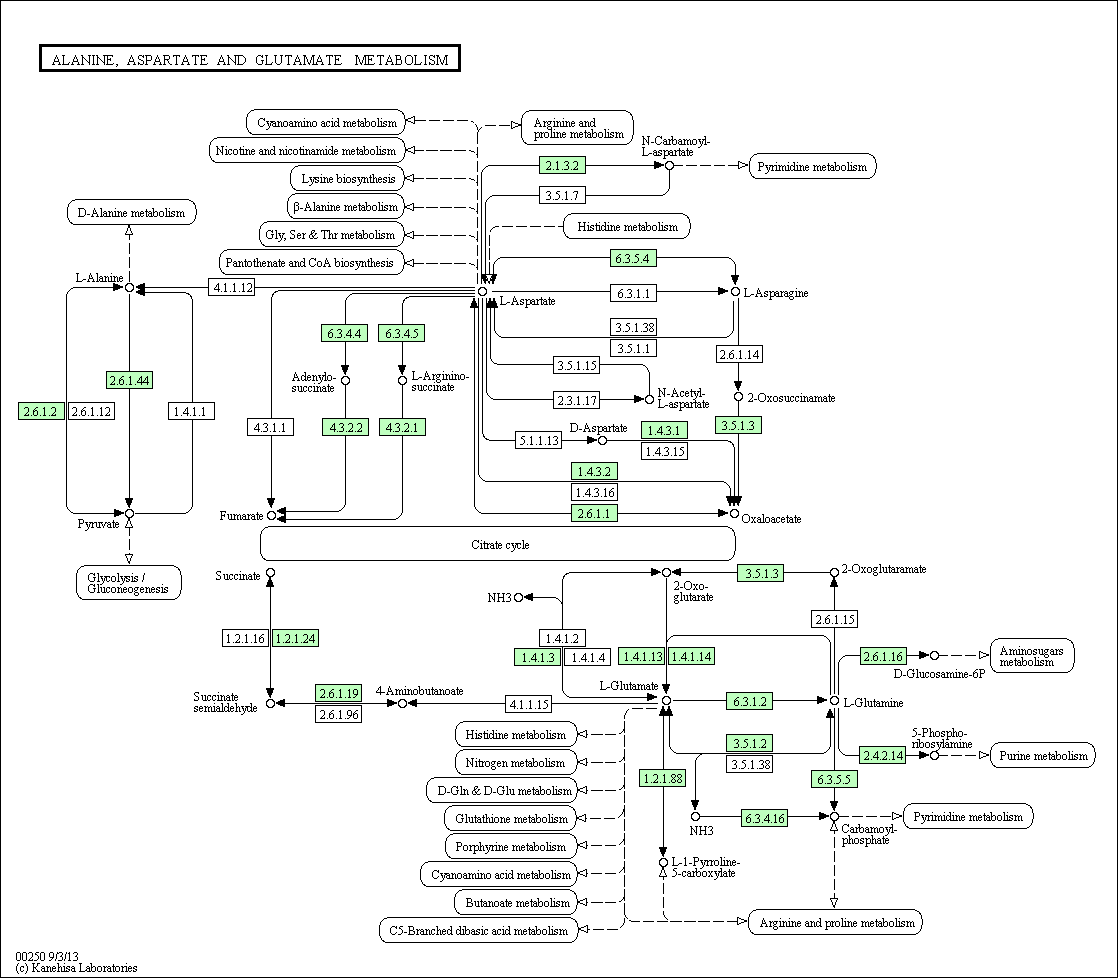

Supplement: Supplemental Information 9 [file peerj-04-1616-s009.gz › map/map00250.png]

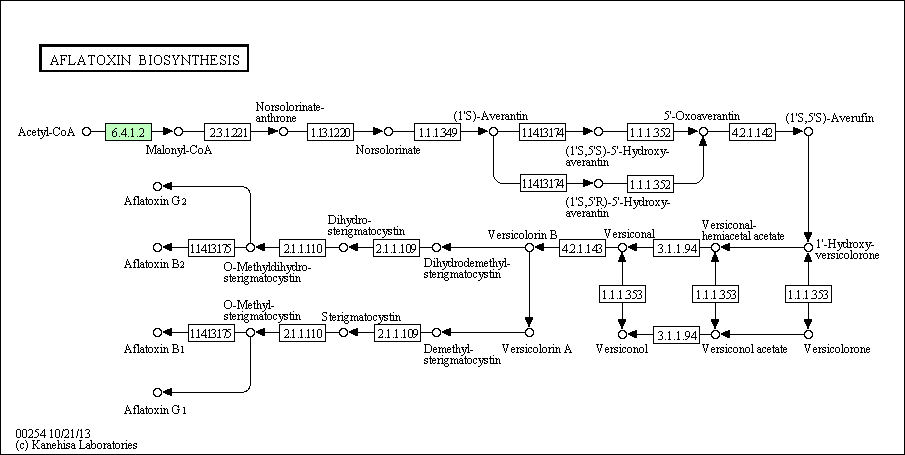

Supplement: Supplemental Information 9 [file peerj-04-1616-s009.gz › map/map00254.png]

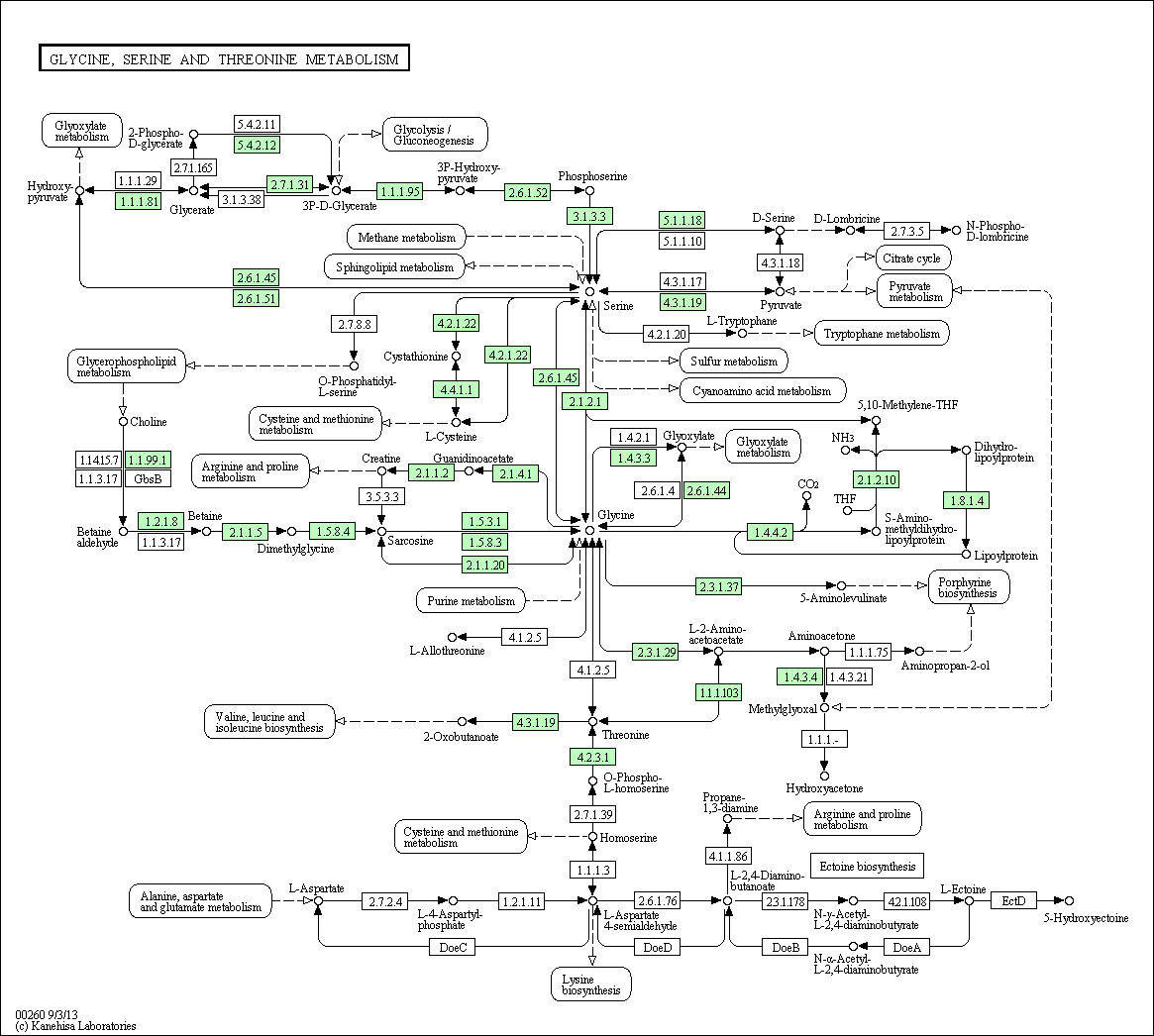

Supplement: Supplemental Information 9 [file peerj-04-1616-s009.gz › map/map00260.png]

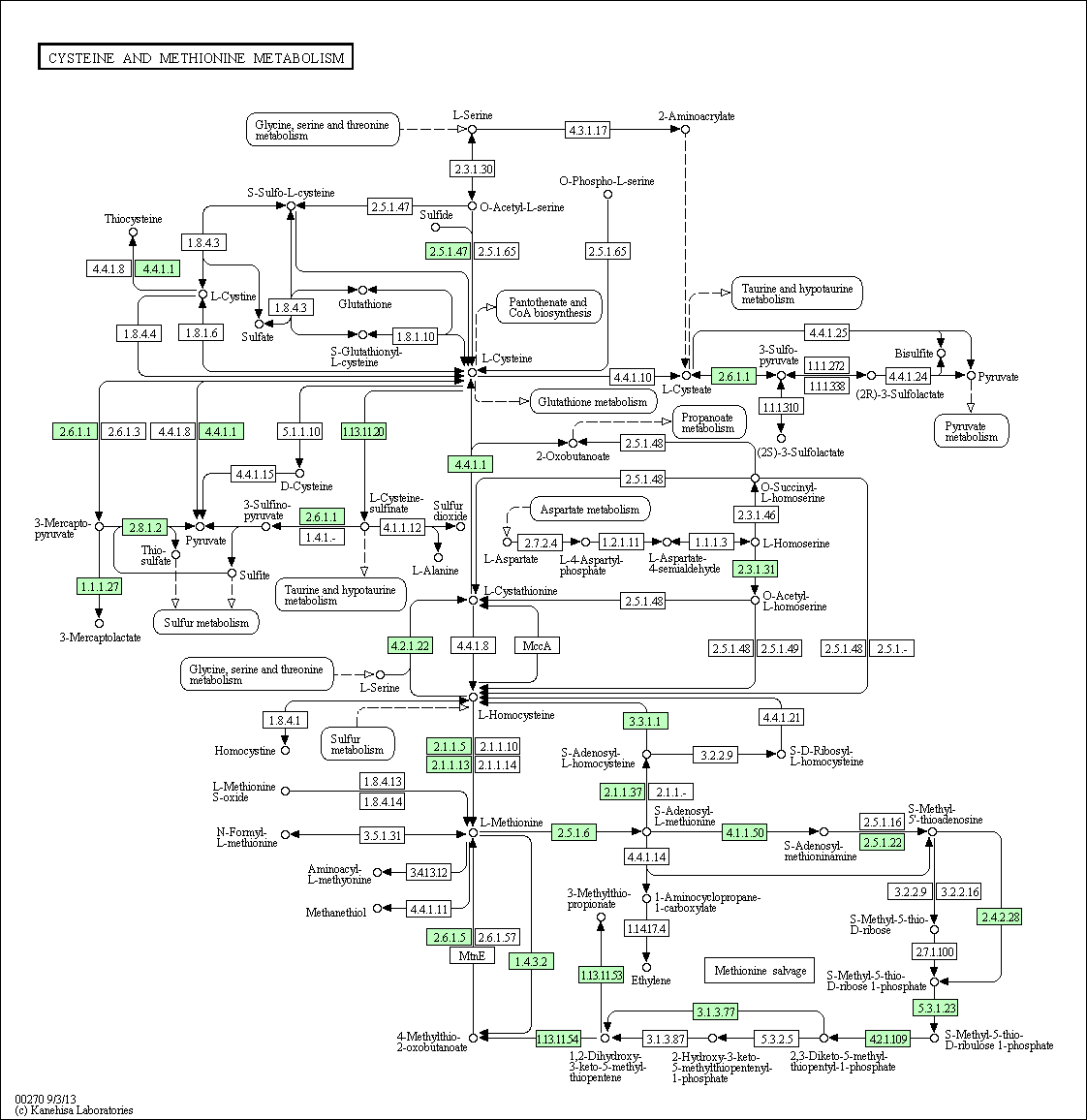

Supplement: Supplemental Information 9 [file peerj-04-1616-s009.gz › map/map00270.png]

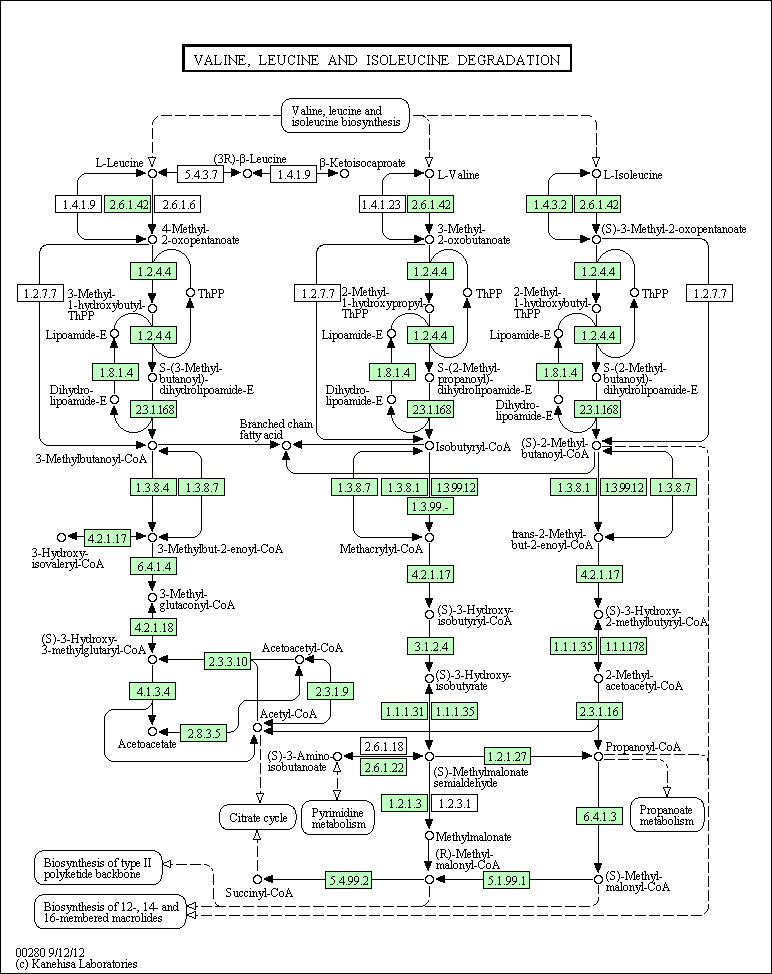

Supplement: Supplemental Information 9 [file peerj-04-1616-s009.gz › map/map00280.png]

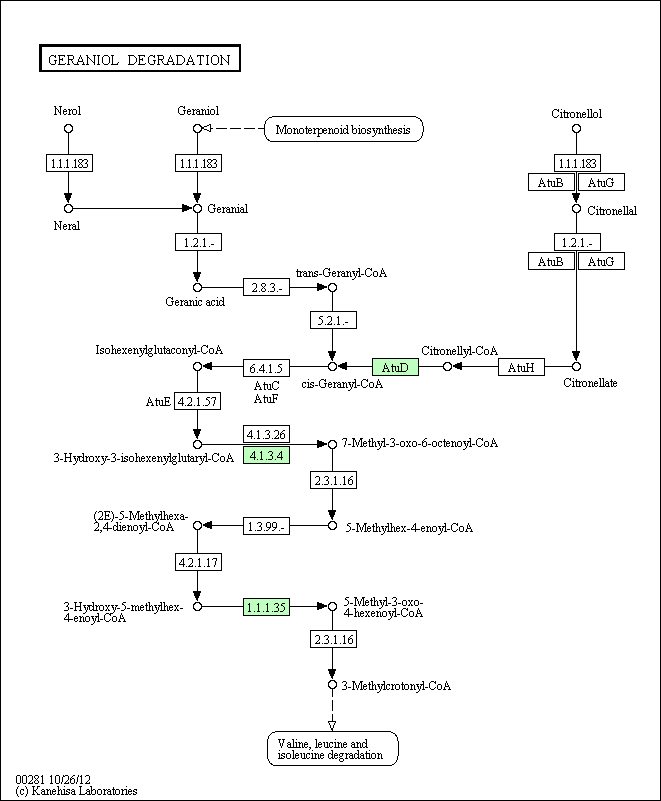

Supplement: Supplemental Information 9 [file peerj-04-1616-s009.gz › map/map00281.png]

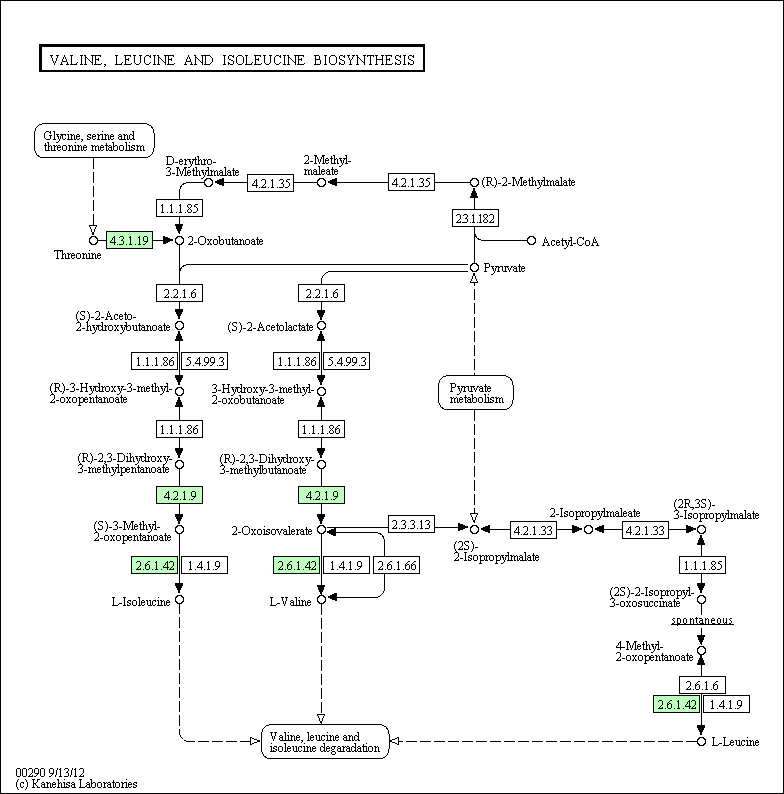

Supplement: Supplemental Information 9 [file peerj-04-1616-s009.gz › map/map00290.png]

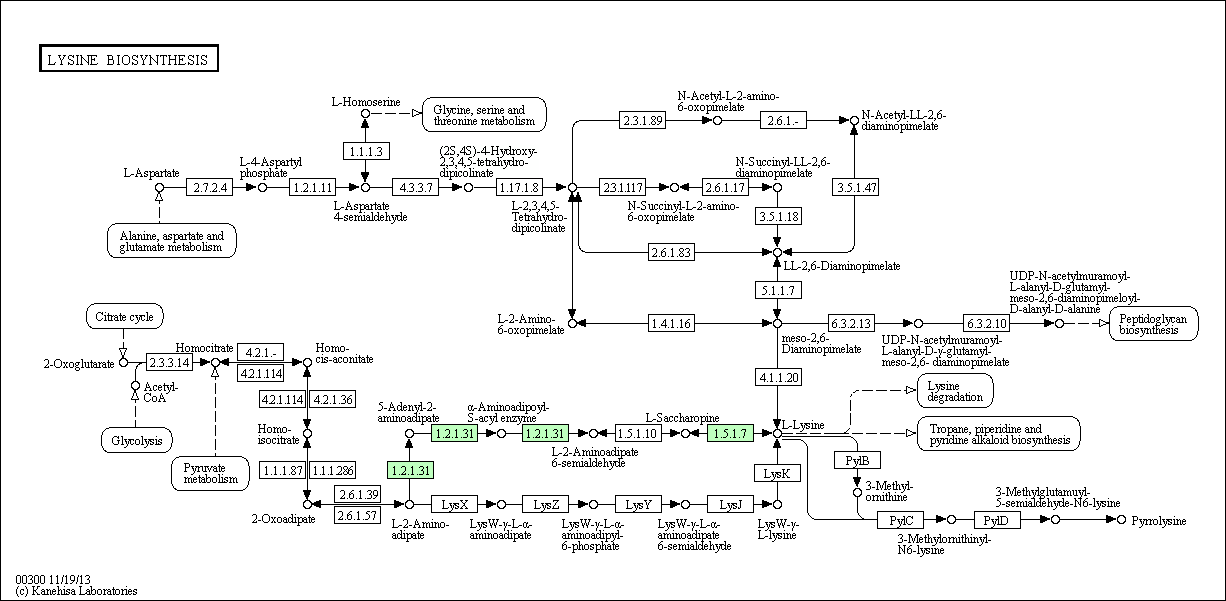

Supplement: Supplemental Information 9 [file peerj-04-1616-s009.gz › map/map00300.png]

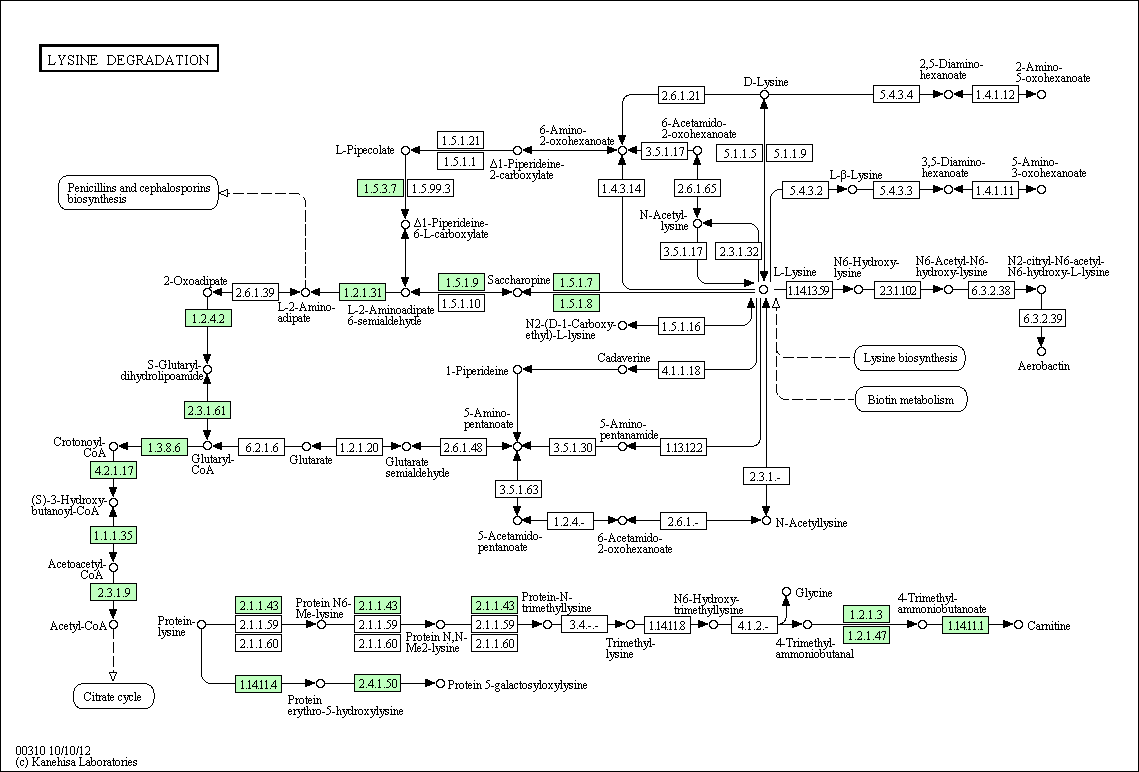

Supplement: Supplemental Information 9 [file peerj-04-1616-s009.gz › map/map00310.png]

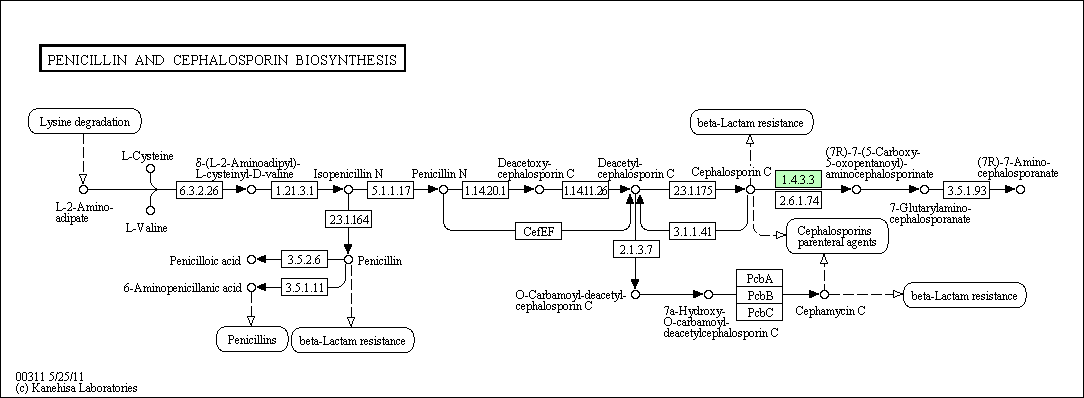

Supplement: Supplemental Information 9 [file peerj-04-1616-s009.gz › map/map00311.png]

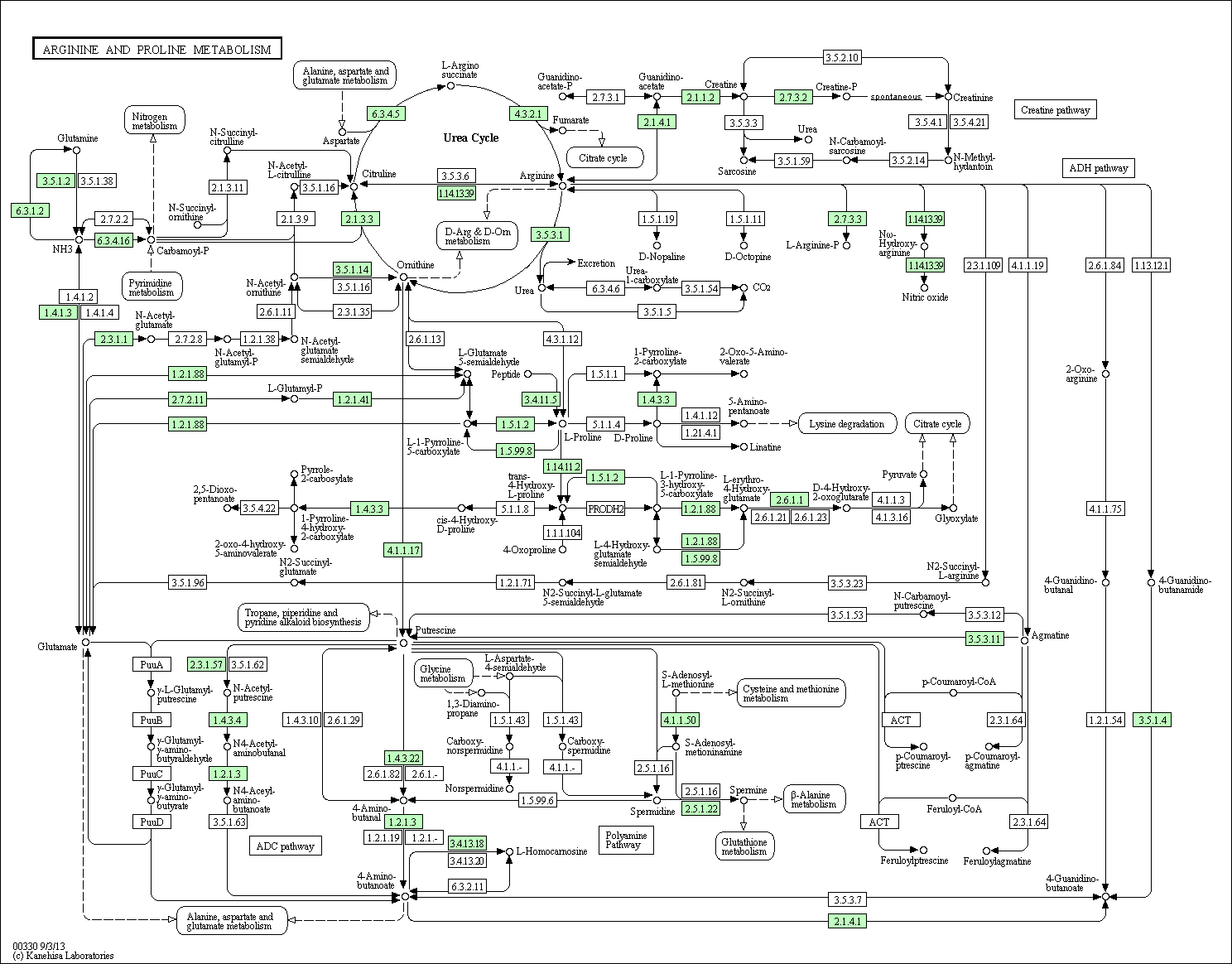

Supplement: Supplemental Information 9 [file peerj-04-1616-s009.gz › map/map00330.png]

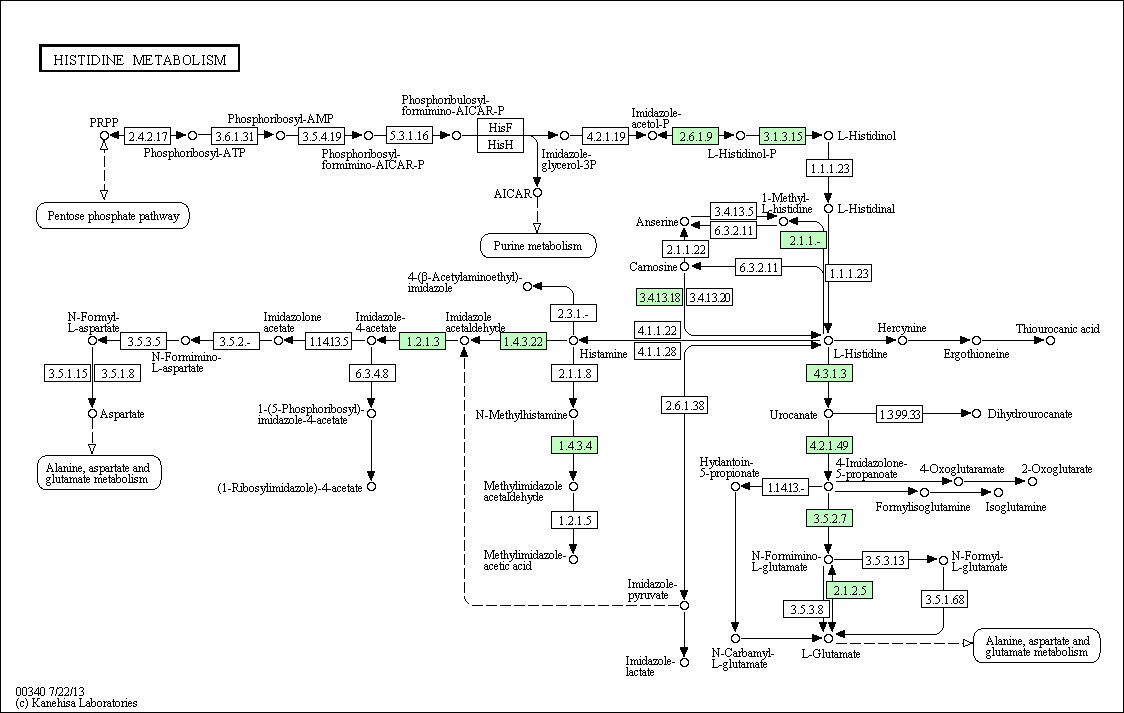

Supplement: Supplemental Information 9 [file peerj-04-1616-s009.gz › map/map00340.png]

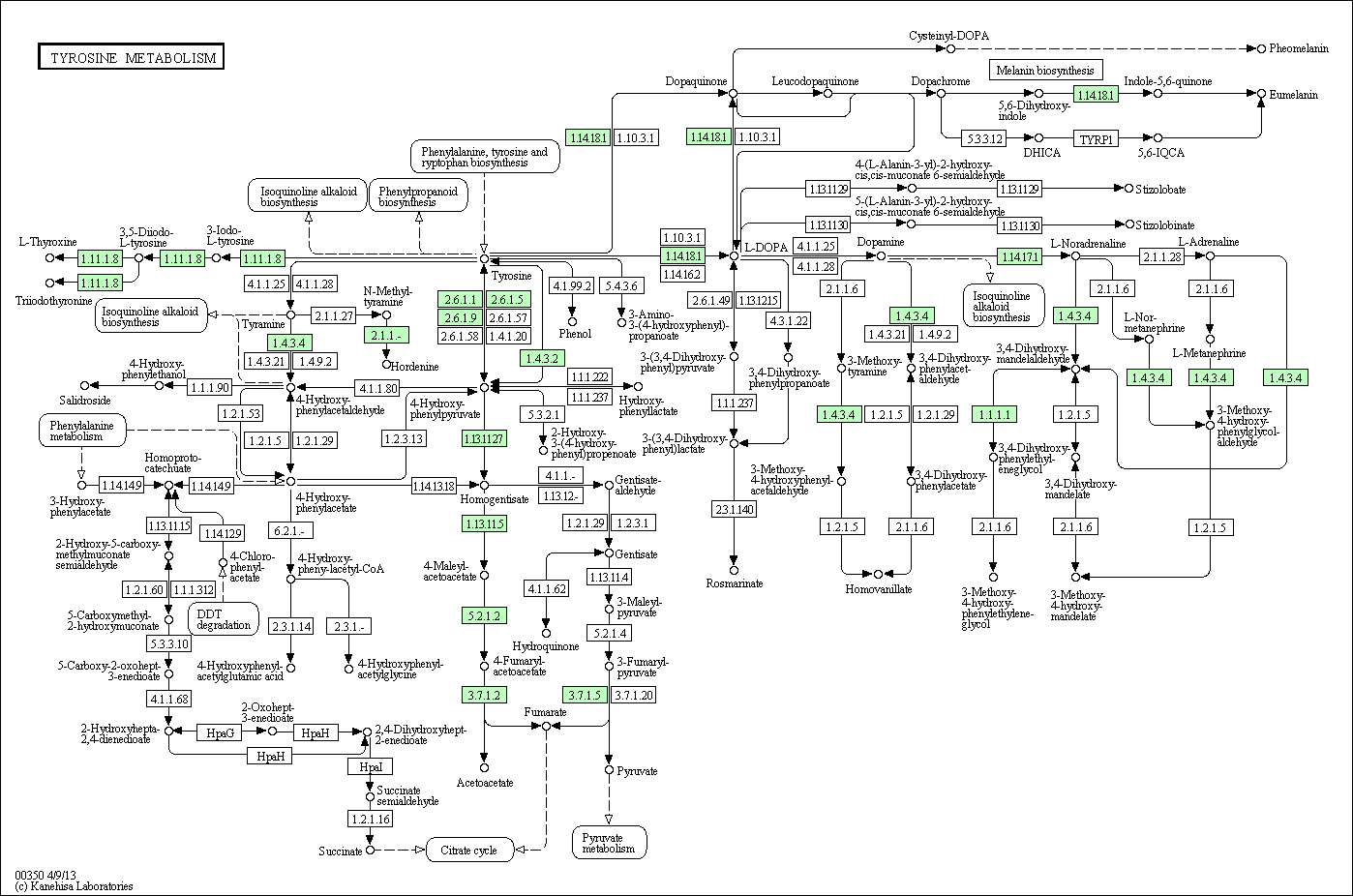

Supplement: Supplemental Information 9 [file peerj-04-1616-s009.gz › map/map00350.png]

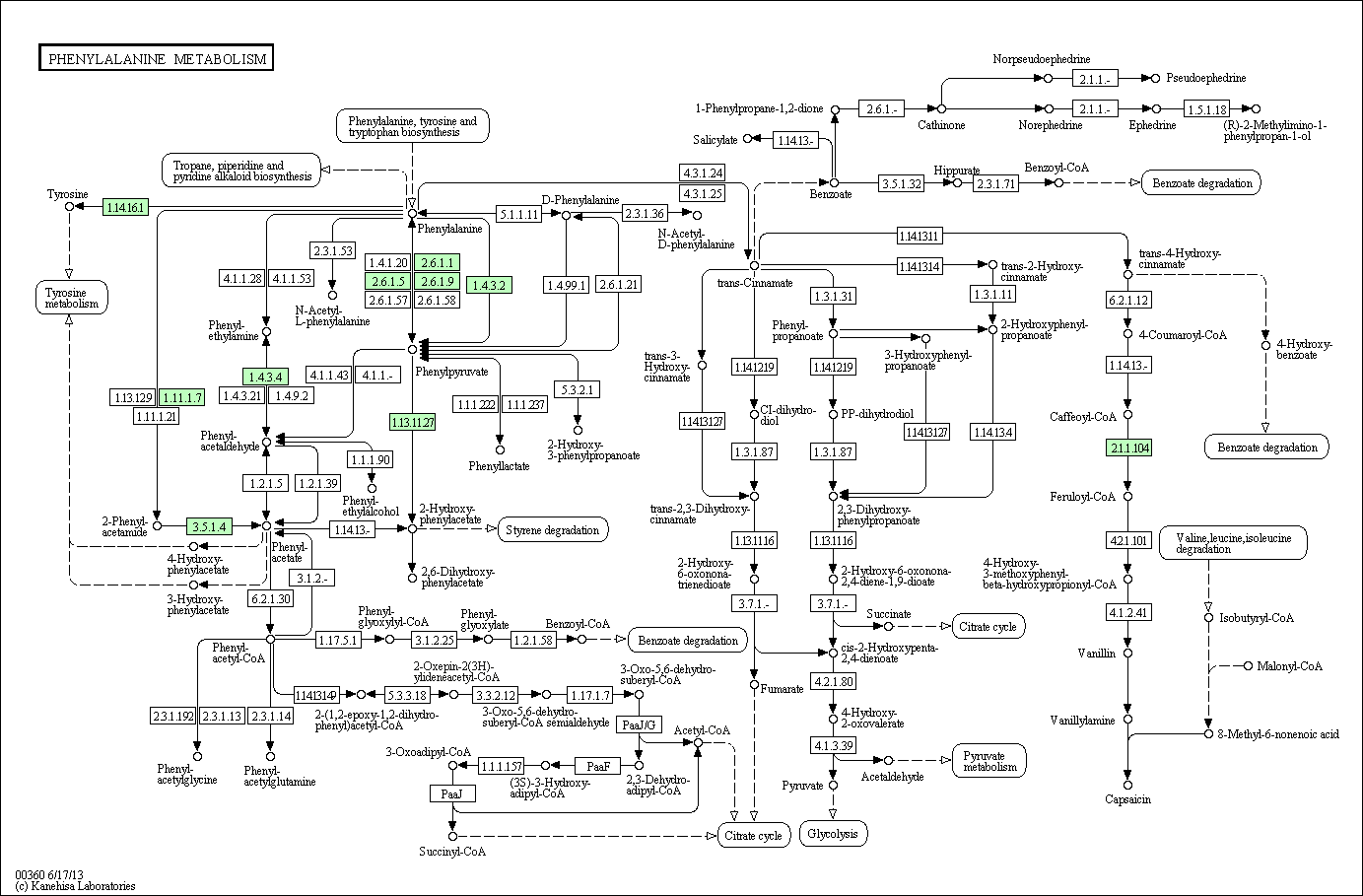

Supplement: Supplemental Information 9 [file peerj-04-1616-s009.gz › map/map00360.png]

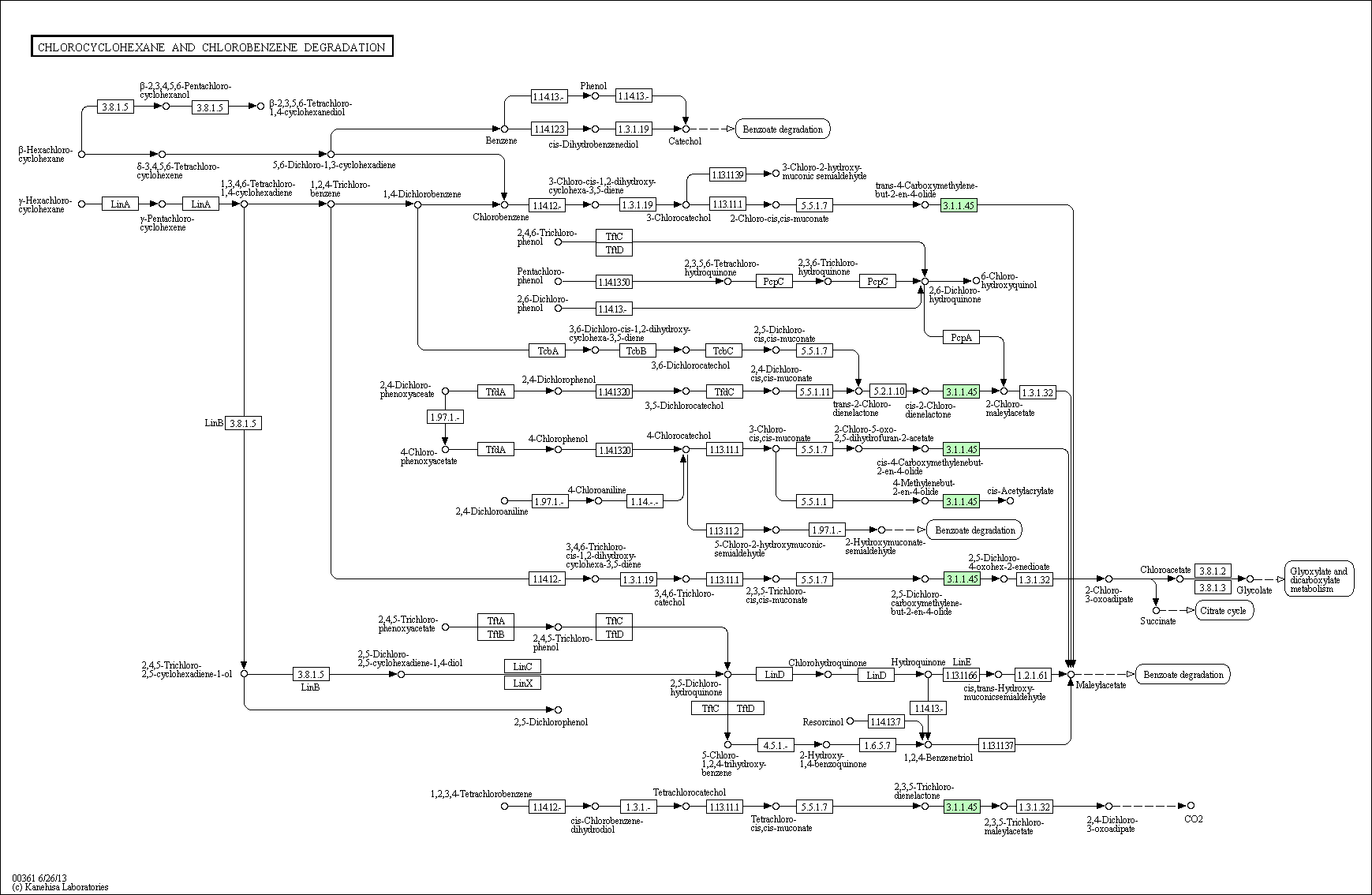

Supplement: Supplemental Information 9 [file peerj-04-1616-s009.gz › map/map00361.png]

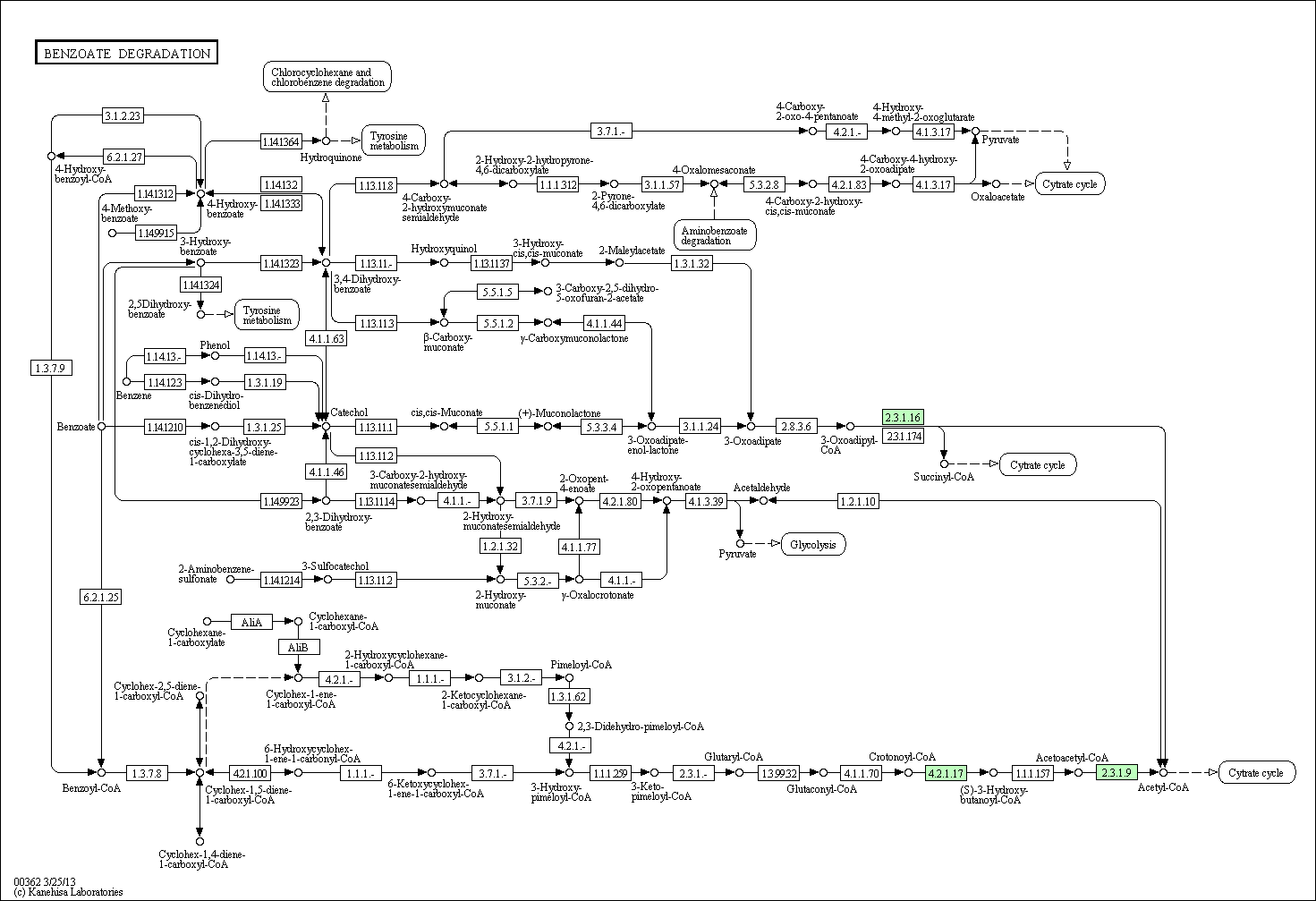

Supplement: Supplemental Information 9 [file peerj-04-1616-s009.gz › map/map00362.png]

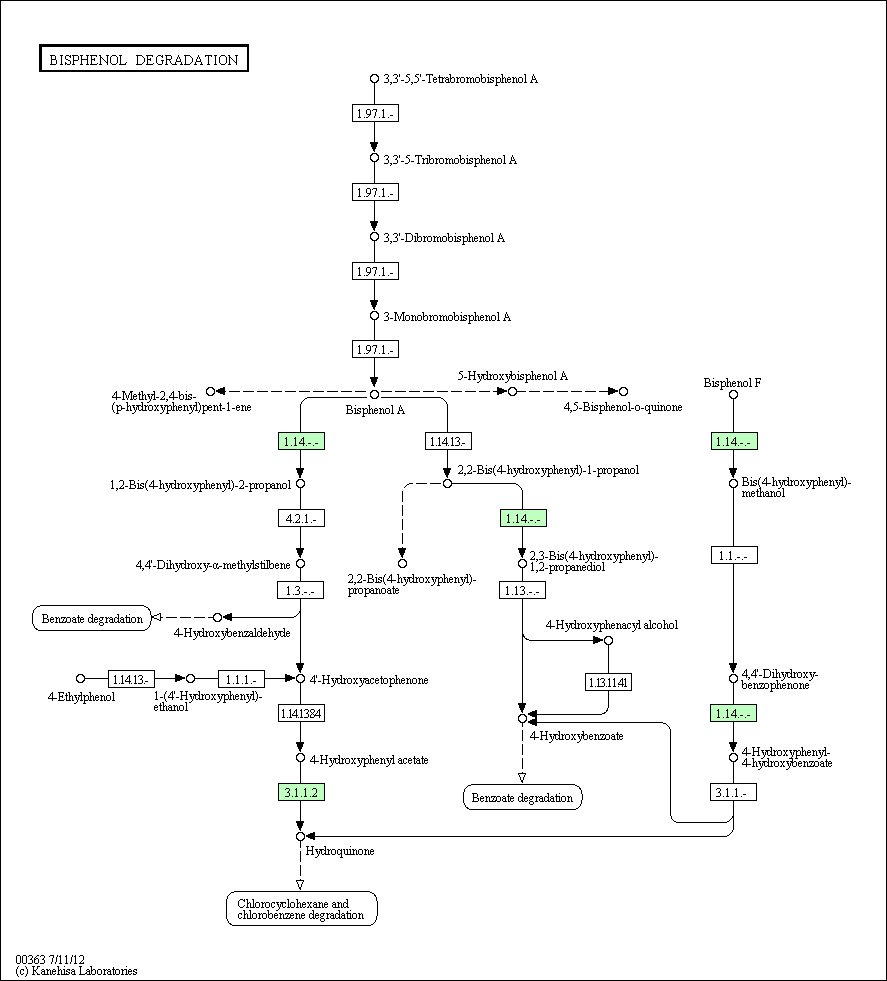

Supplement: Supplemental Information 9 [file peerj-04-1616-s009.gz › map/map00363.png]

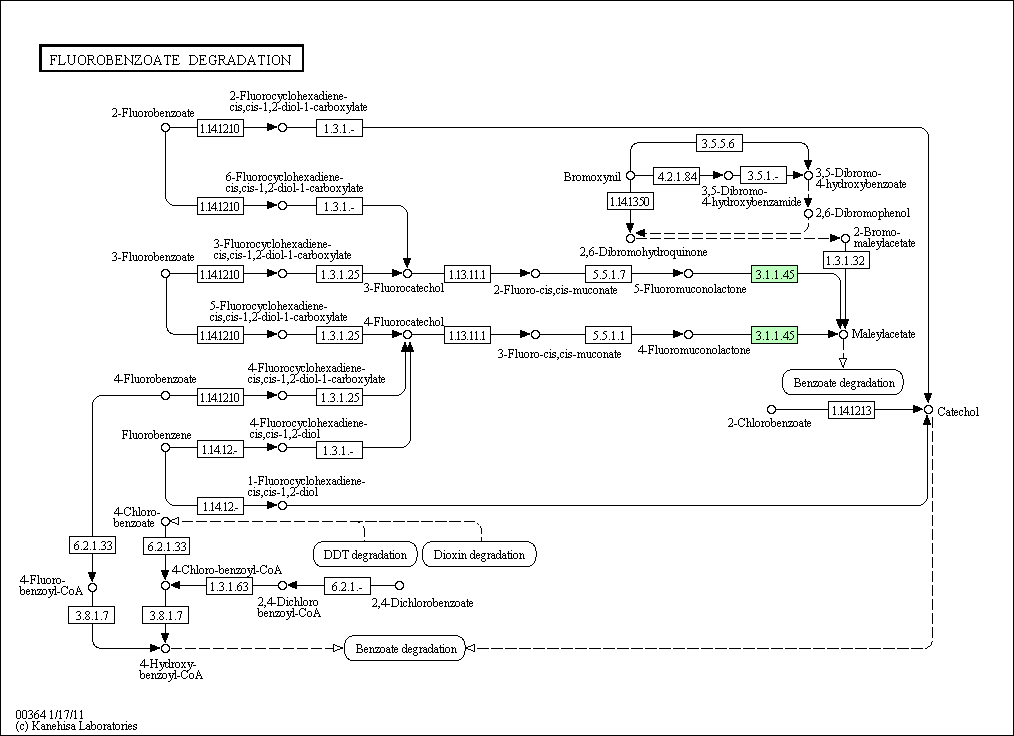

Supplement: Supplemental Information 9 [file peerj-04-1616-s009.gz › map/map00364.png]

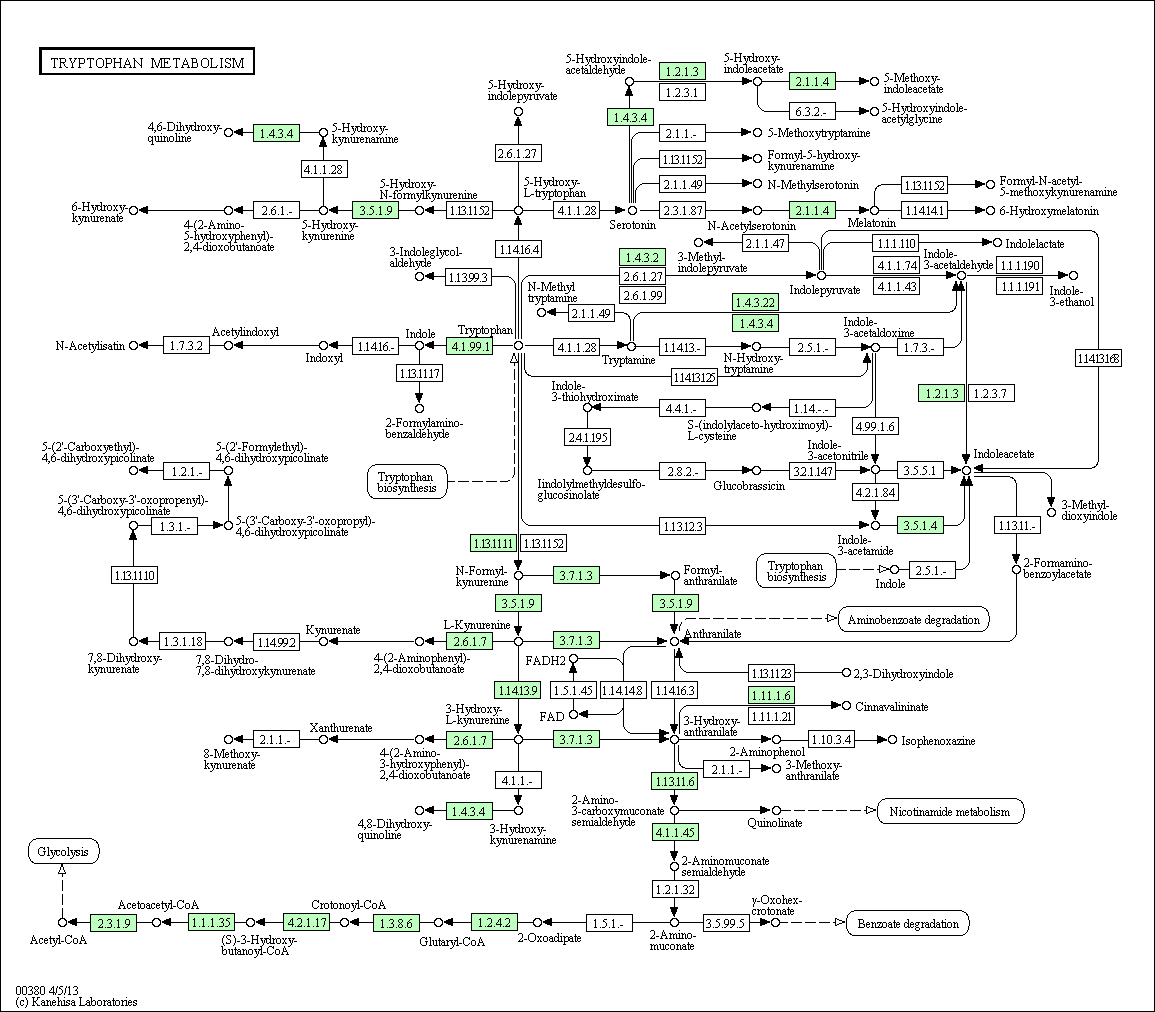

Supplement: Supplemental Information 9 [file peerj-04-1616-s009.gz › map/map00380.png]

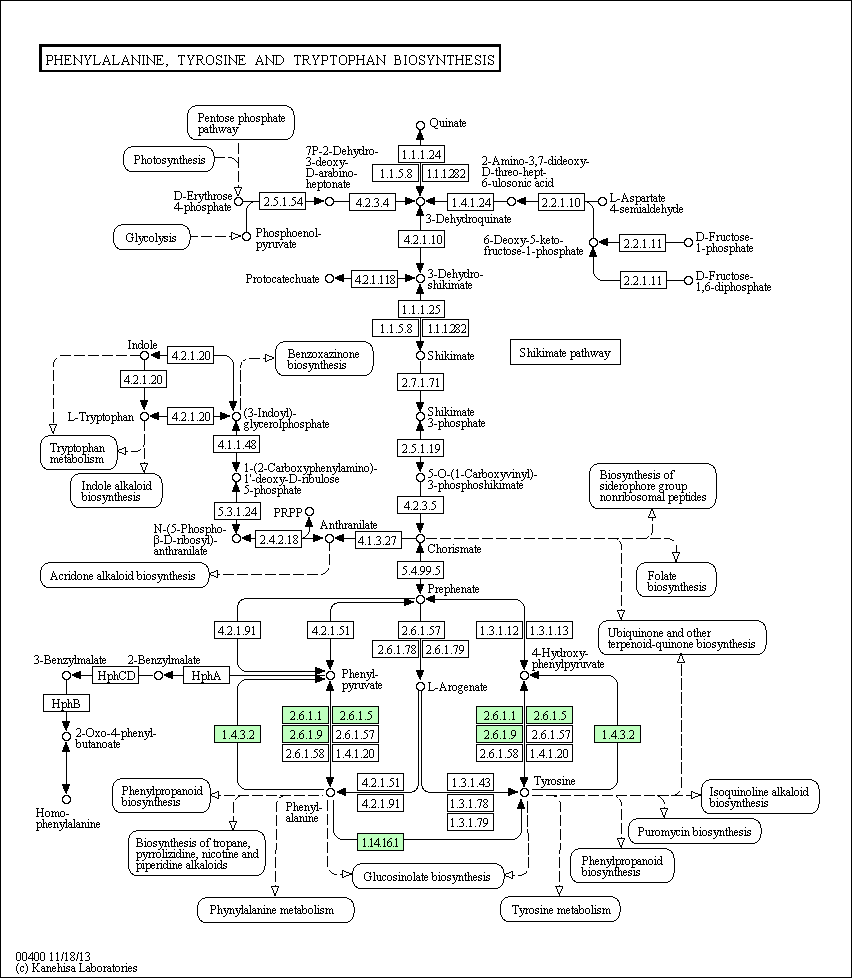

Supplement: Supplemental Information 9 [file peerj-04-1616-s009.gz › map/map00400.png]

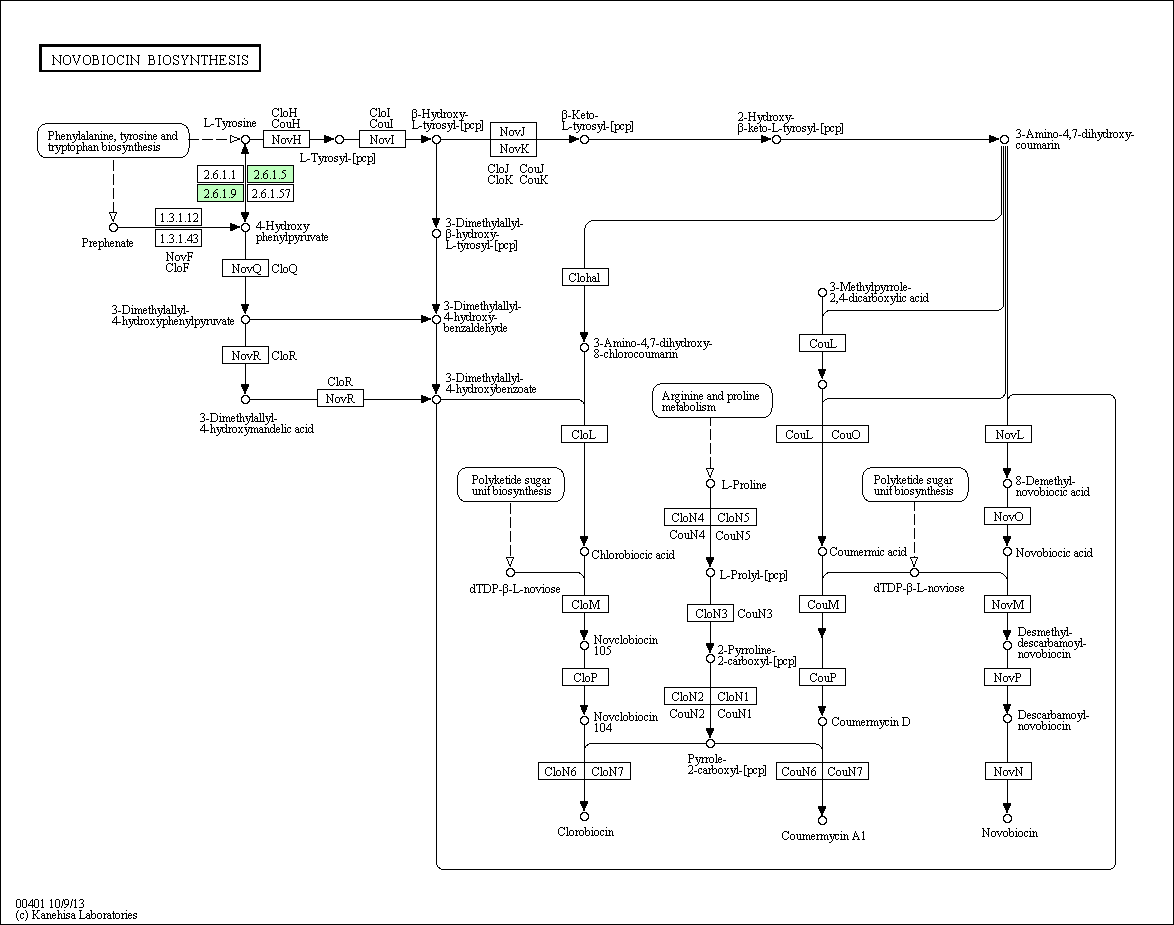

Supplement: Supplemental Information 9 [file peerj-04-1616-s009.gz › map/map00401.png]

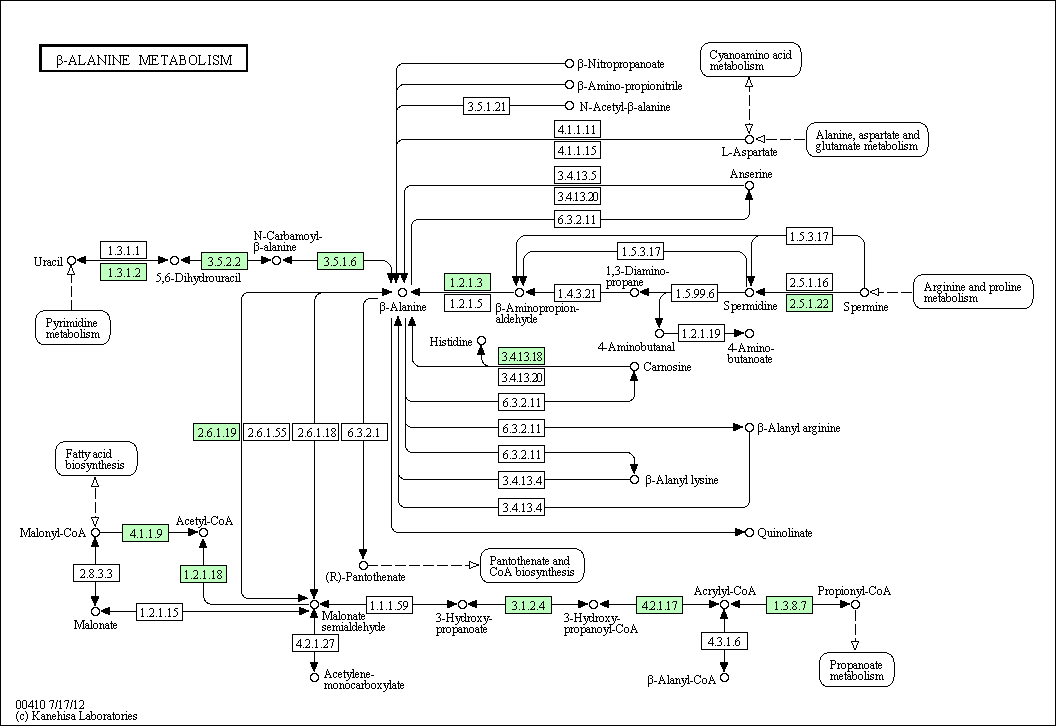

Supplement: Supplemental Information 9 [file peerj-04-1616-s009.gz › map/map00410.png]

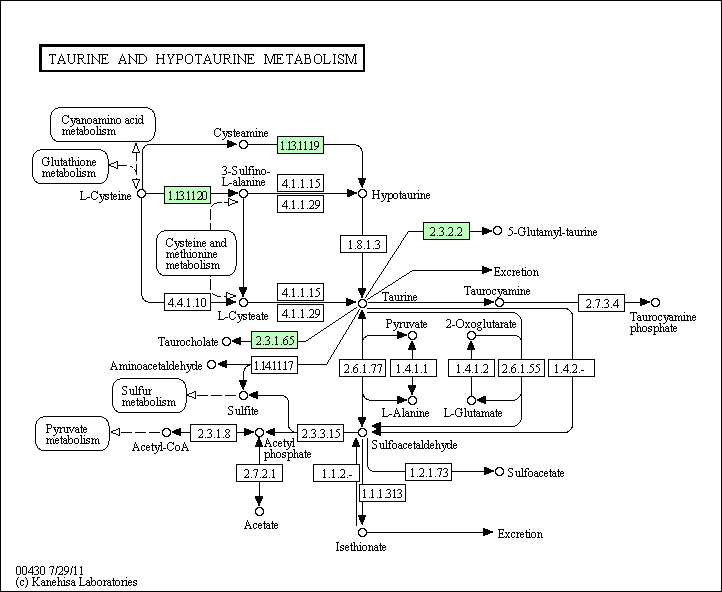

Supplement: Supplemental Information 9 [file peerj-04-1616-s009.gz › map/map00430.png]

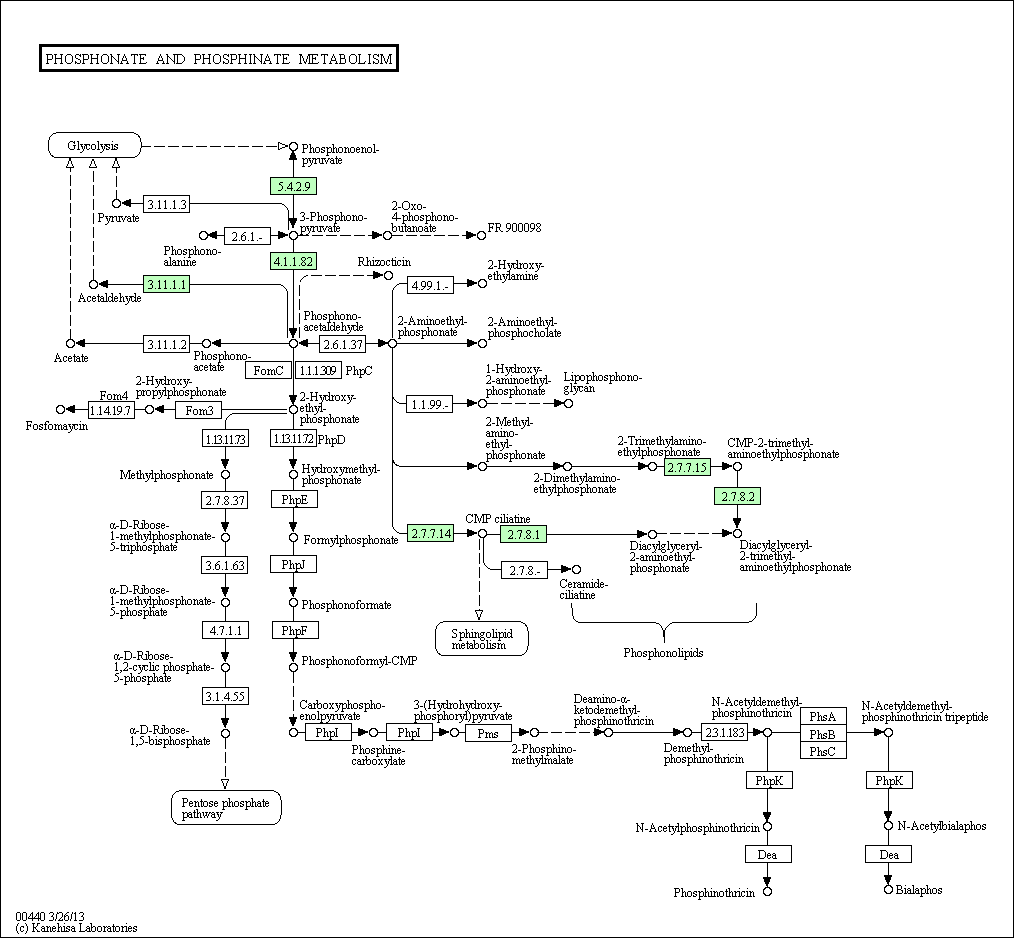

Supplement: Supplemental Information 9 [file peerj-04-1616-s009.gz › map/map00440.png]

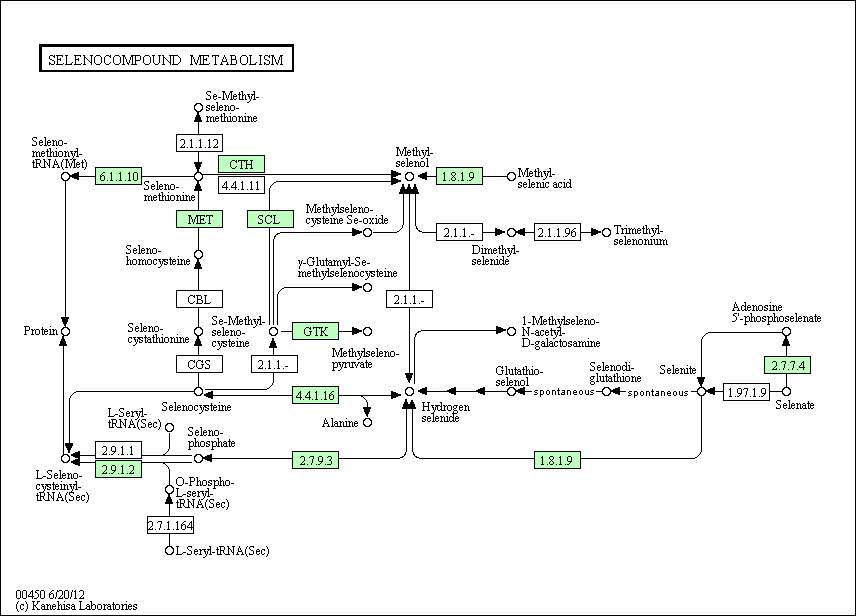

Supplement: Supplemental Information 9 [file peerj-04-1616-s009.gz › map/map00450.png]

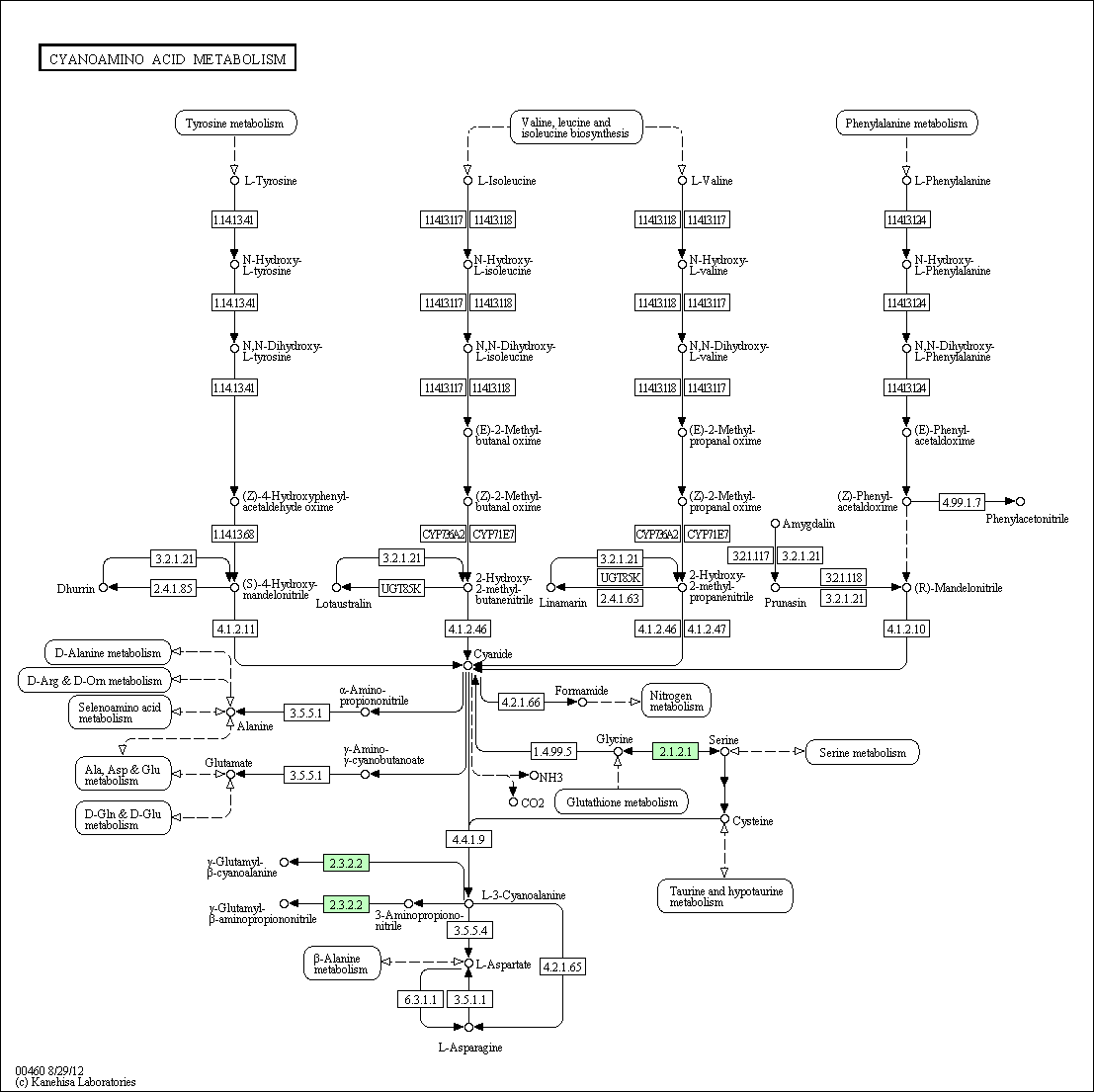

Supplement: Supplemental Information 9 [file peerj-04-1616-s009.gz › map/map00460.png]

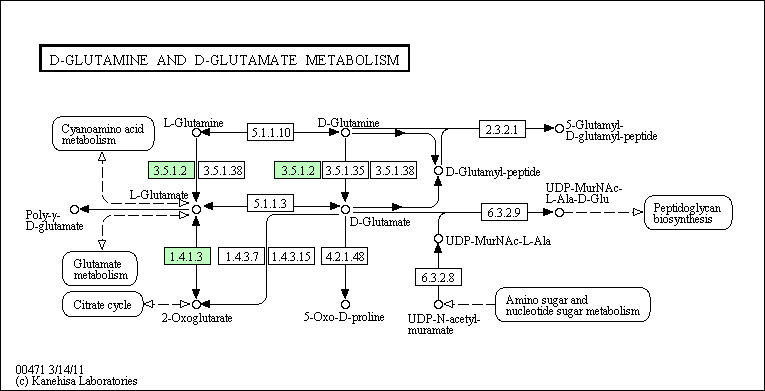

Supplement: Supplemental Information 9 [file peerj-04-1616-s009.gz › map/map00471.png]

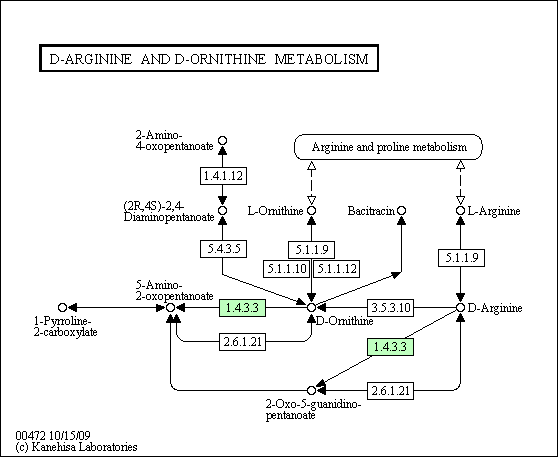

Supplement: Supplemental Information 9 [file peerj-04-1616-s009.gz › map/map00472.png]

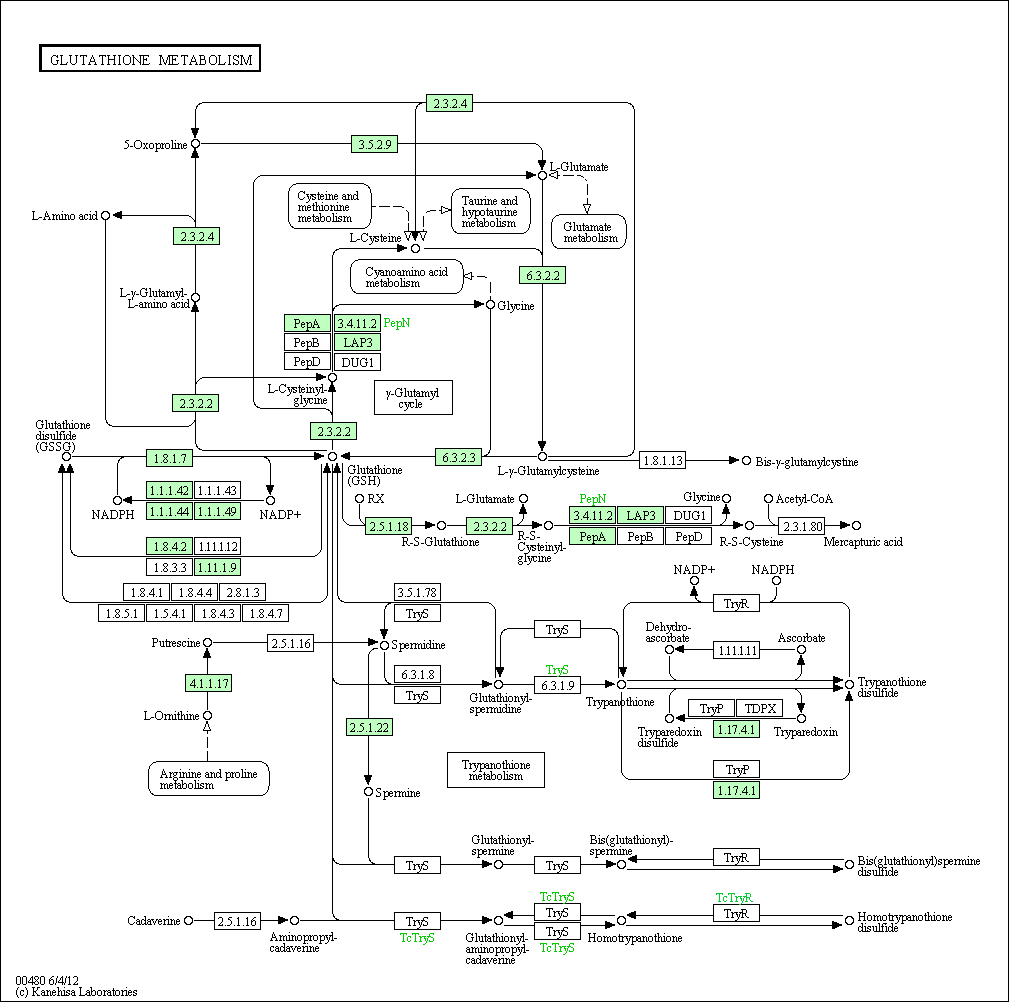

Supplement: Supplemental Information 9 [file peerj-04-1616-s009.gz › map/map00480.png]

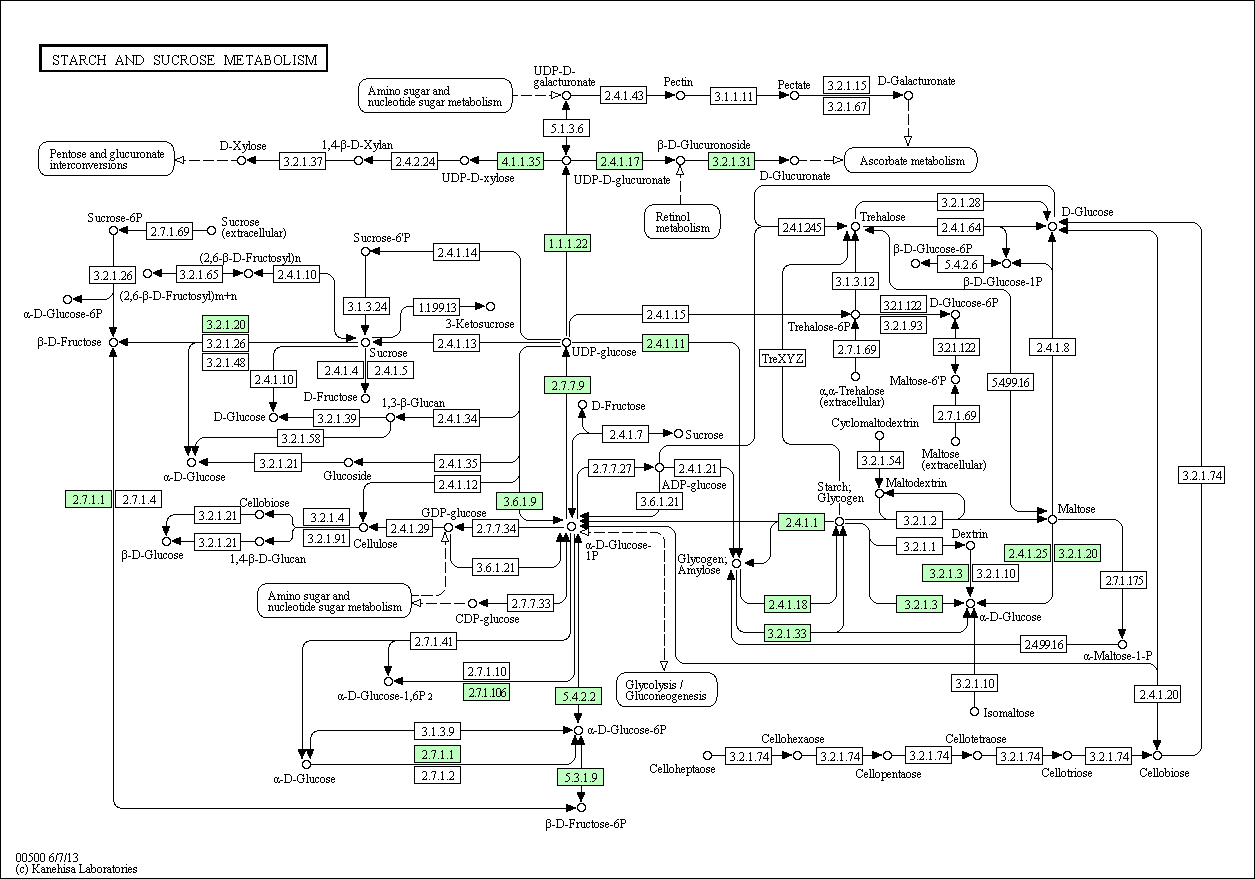

Supplement: Supplemental Information 9 [file peerj-04-1616-s009.gz › map/map00500.png]

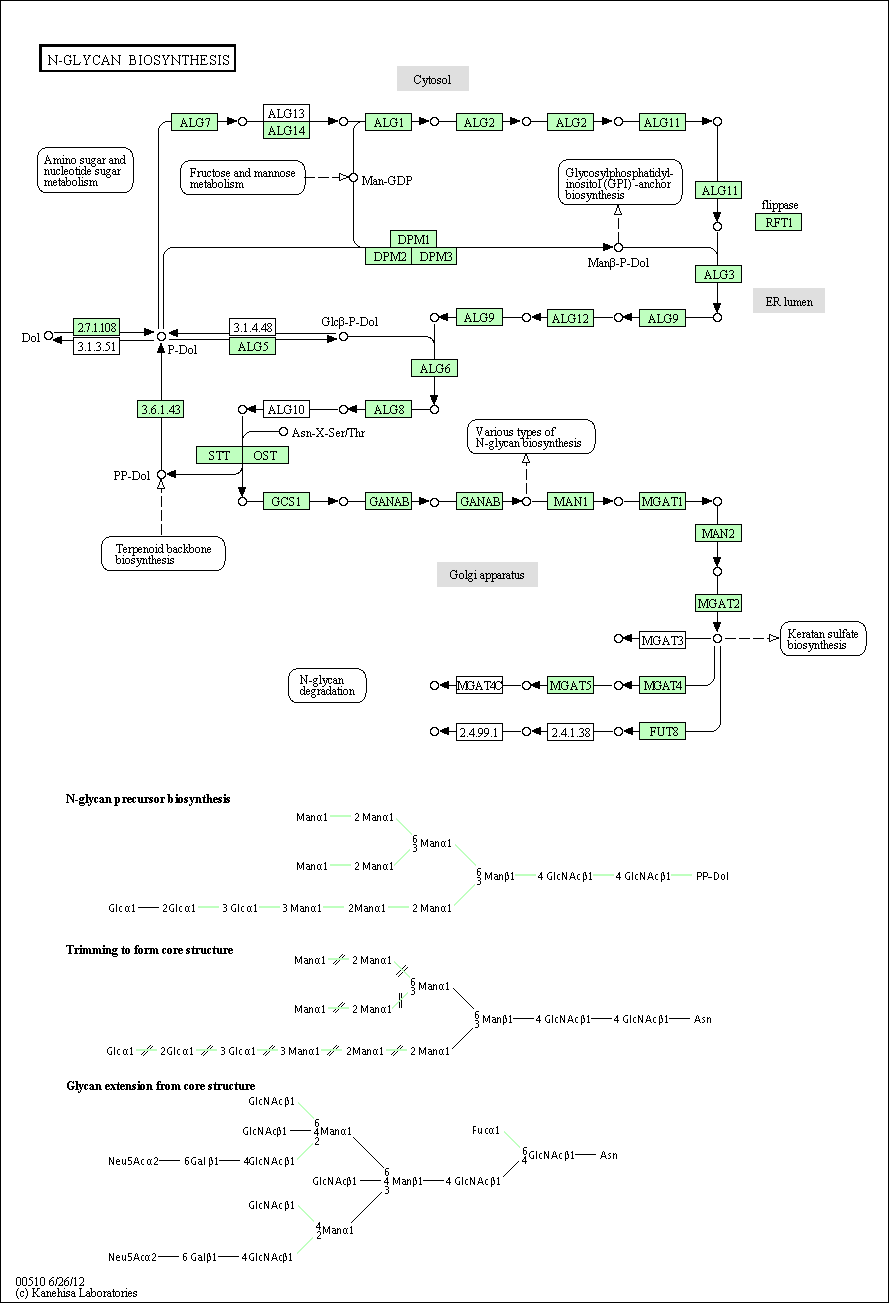

Supplement: Supplemental Information 9 [file peerj-04-1616-s009.gz › map/map00510.png]

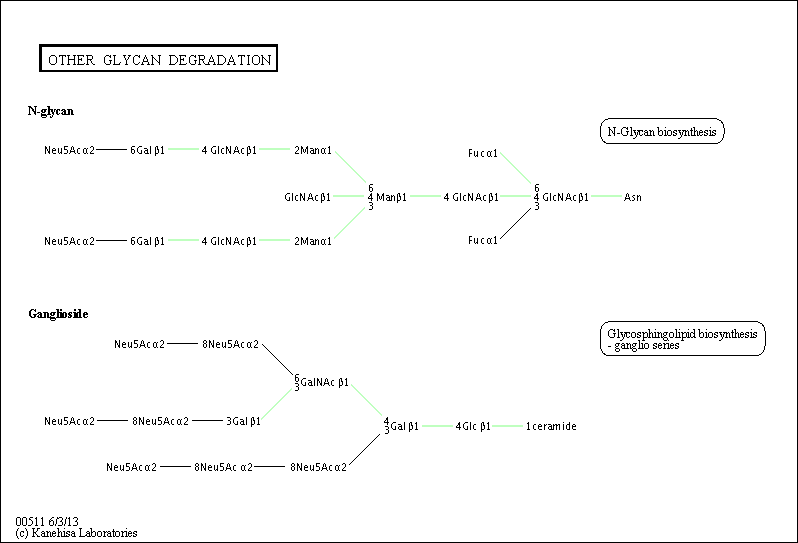

Supplement: Supplemental Information 9 [file peerj-04-1616-s009.gz › map/map00511.png]

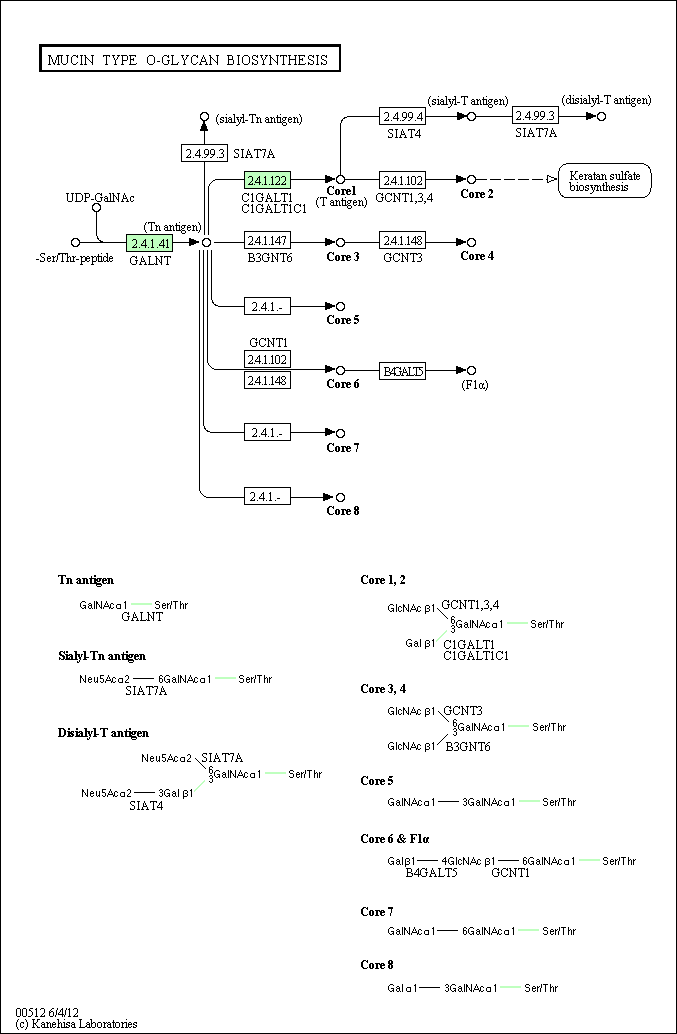

Supplement: Supplemental Information 9 [file peerj-04-1616-s009.gz › map/map00512.png]

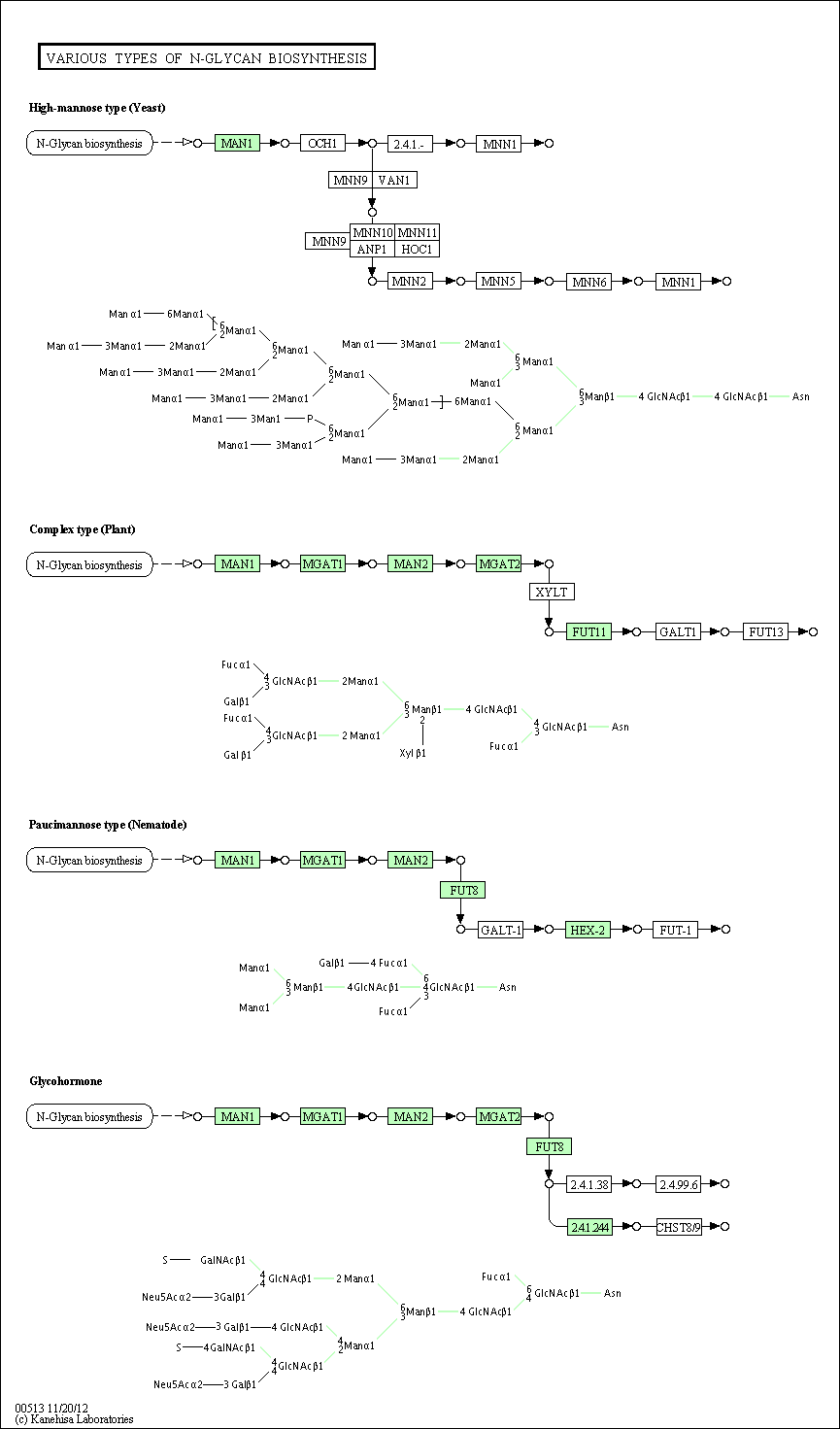

Supplement: Supplemental Information 9 [file peerj-04-1616-s009.gz › map/map00513.png]

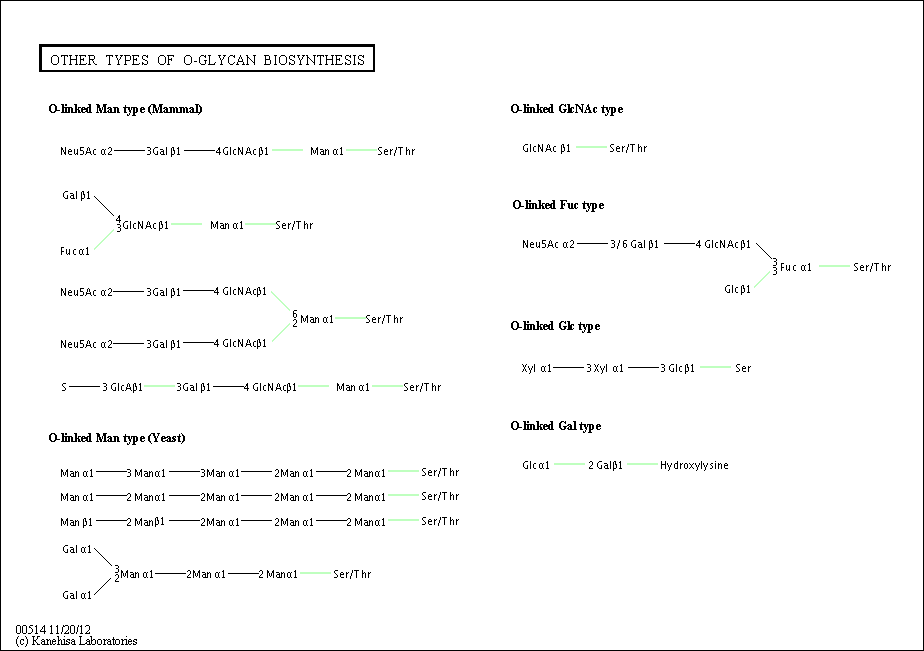

Supplement: Supplemental Information 9 [file peerj-04-1616-s009.gz › map/map00514.png]

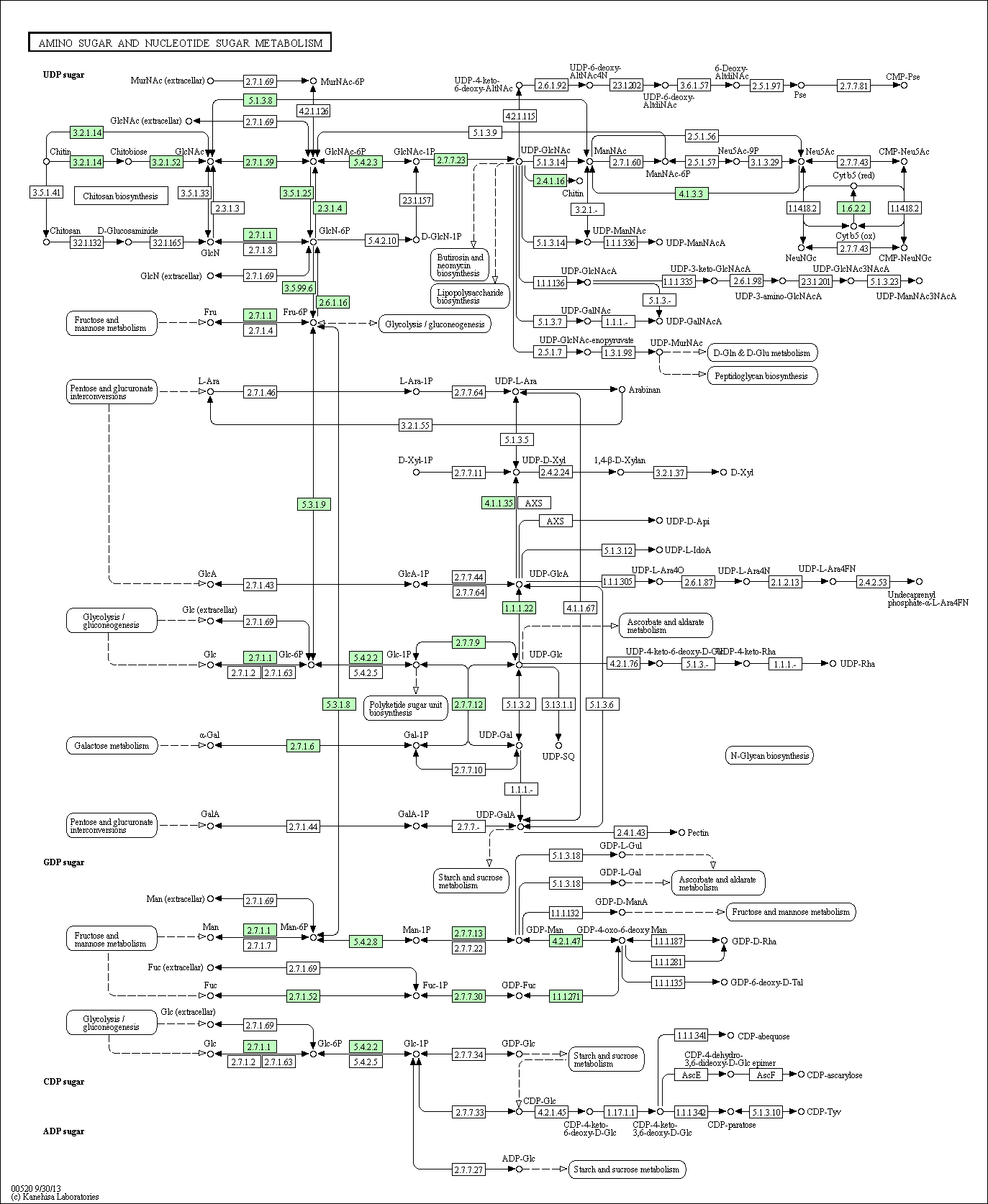

Supplement: Supplemental Information 9 [file peerj-04-1616-s009.gz › map/map00520.png]

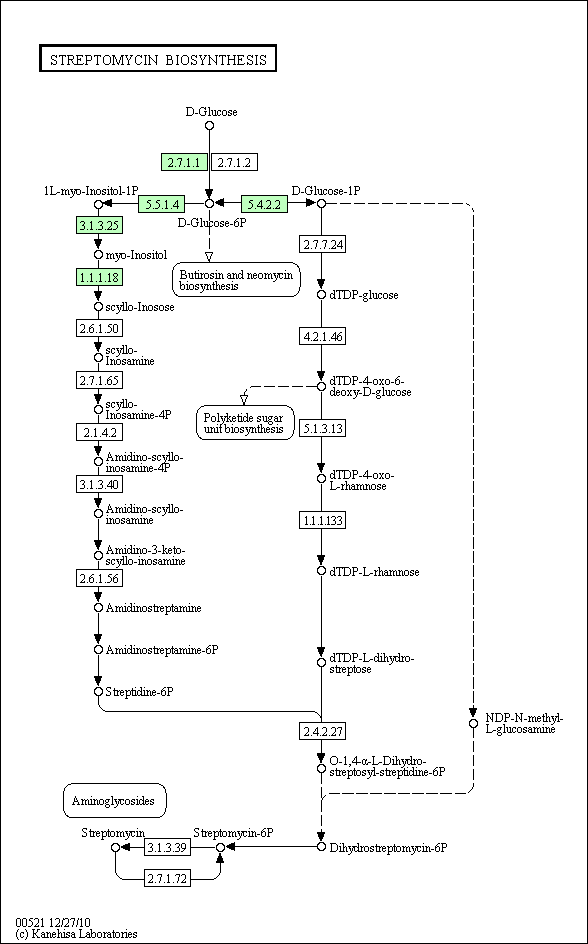

Supplement: Supplemental Information 9 [file peerj-04-1616-s009.gz › map/map00521.png]

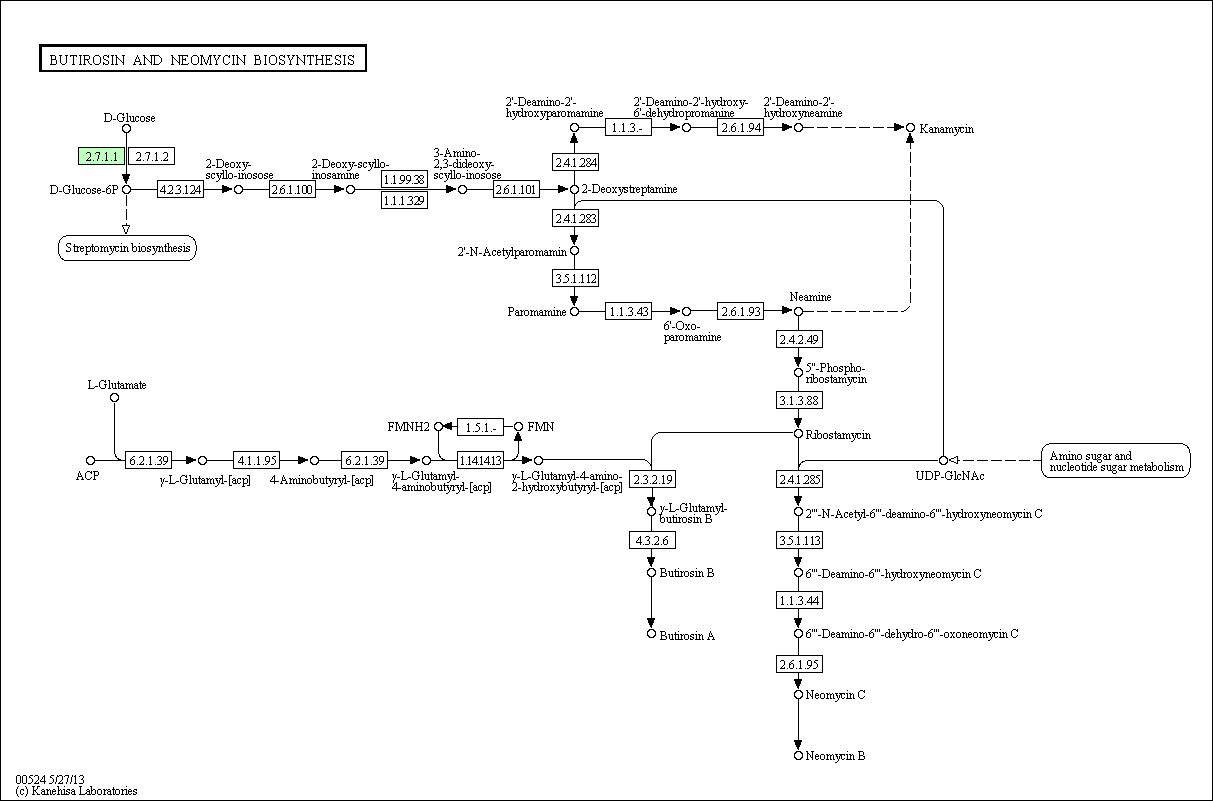

Supplement: Supplemental Information 9 [file peerj-04-1616-s009.gz › map/map00524.png]

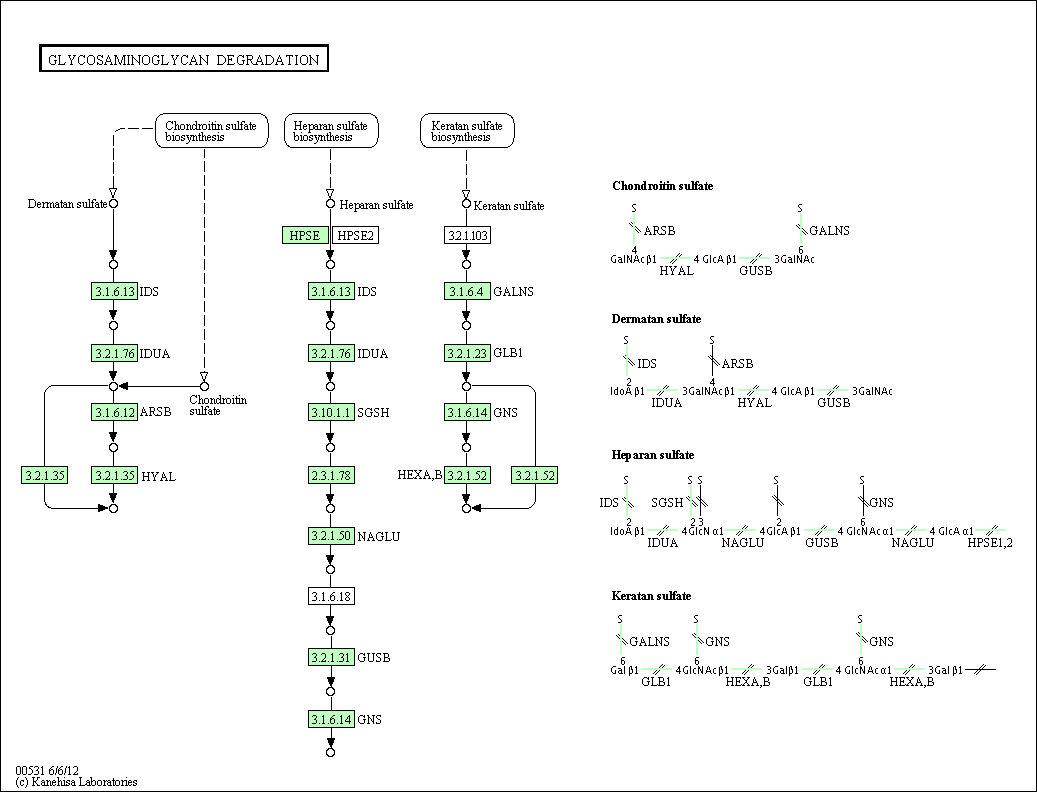

Supplement: Supplemental Information 9 [file peerj-04-1616-s009.gz › map/map00531.png]

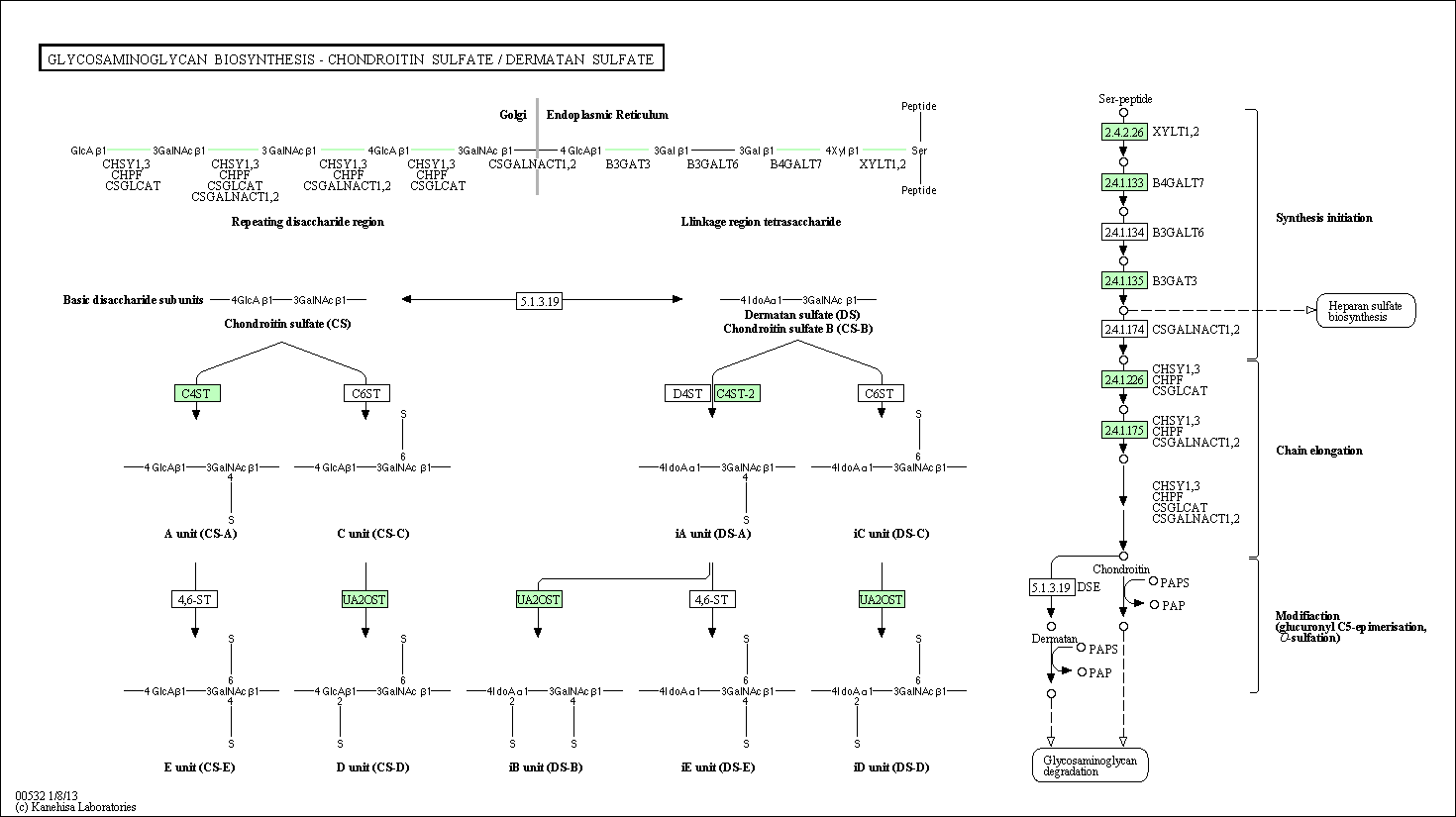

Supplement: Supplemental Information 9 [file peerj-04-1616-s009.gz › map/map00532.png]

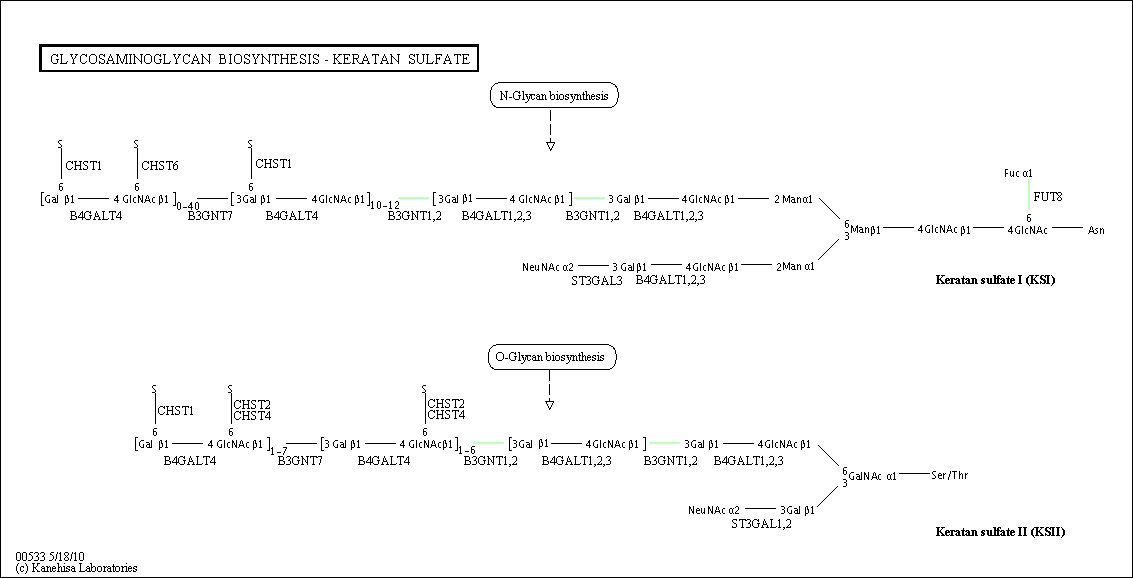

Supplement: Supplemental Information 9 [file peerj-04-1616-s009.gz › map/map00533.png]

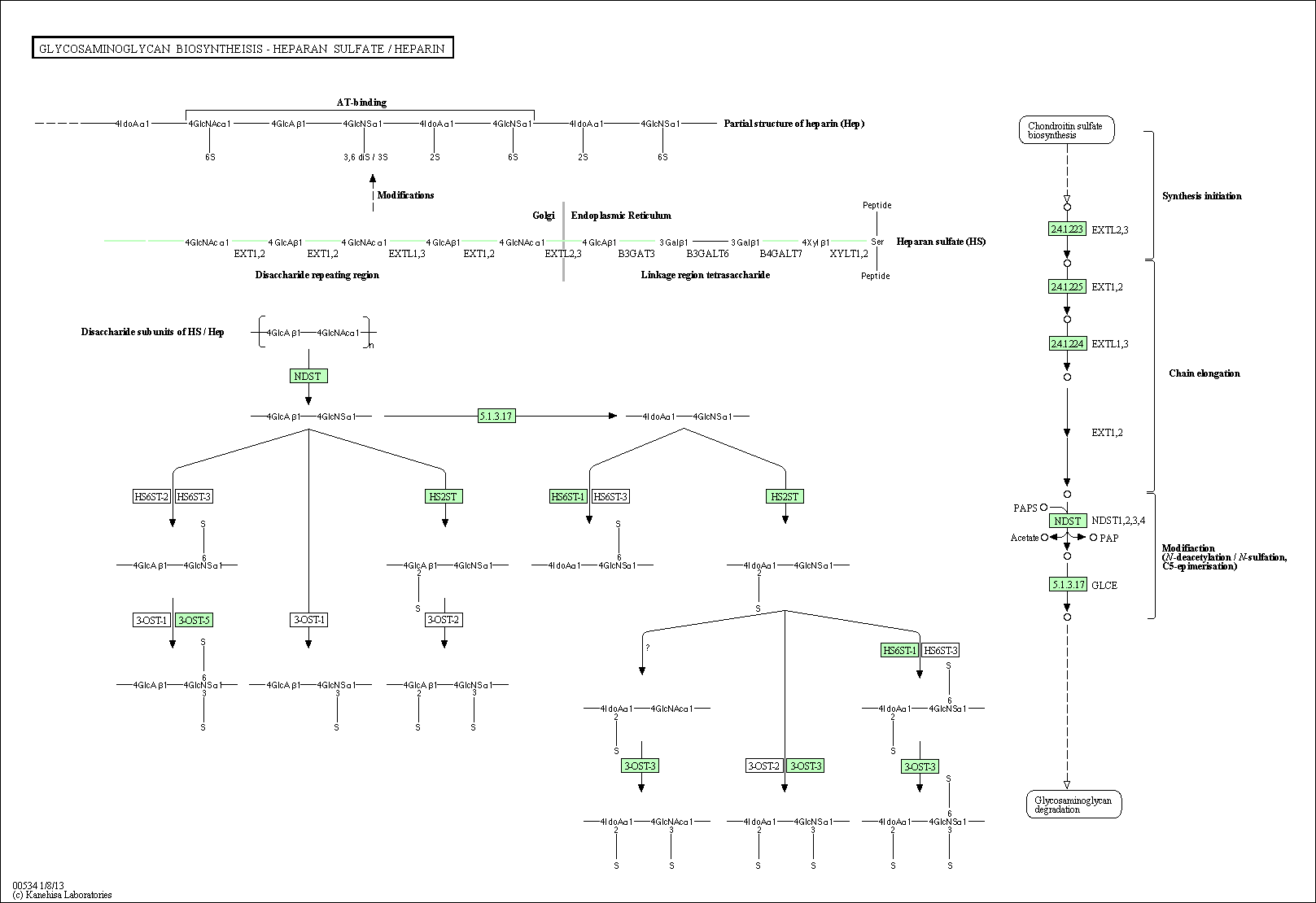

Supplement: Supplemental Information 9 [file peerj-04-1616-s009.gz › map/map00534.png]

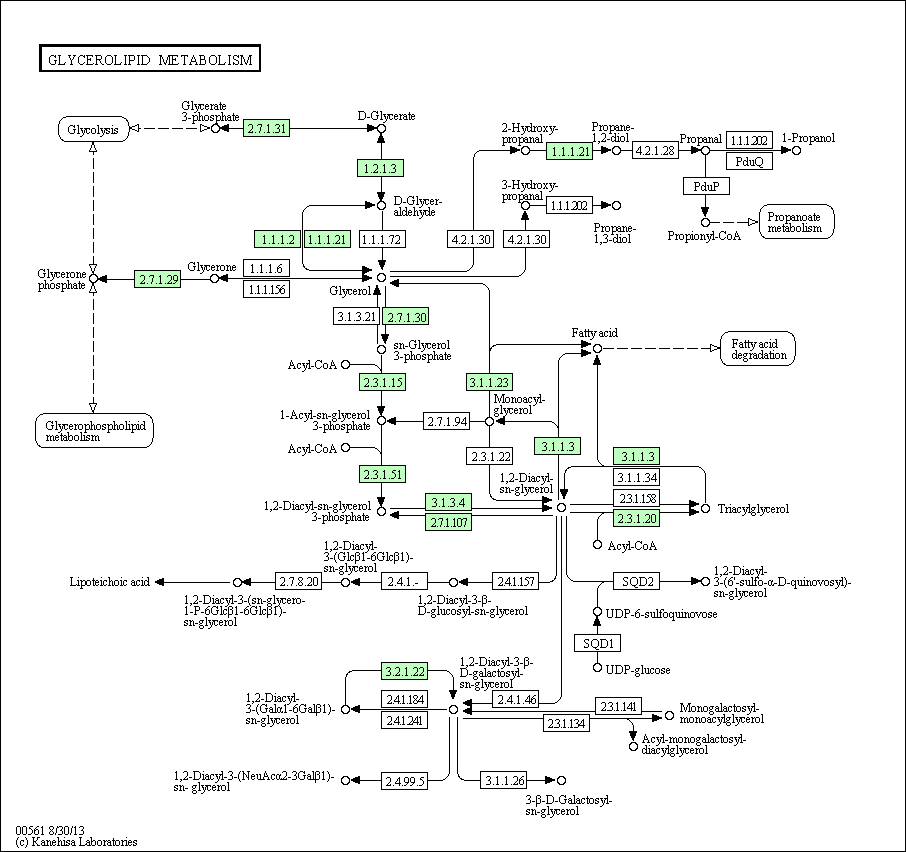

Supplement: Supplemental Information 9 [file peerj-04-1616-s009.gz › map/map00561.png]

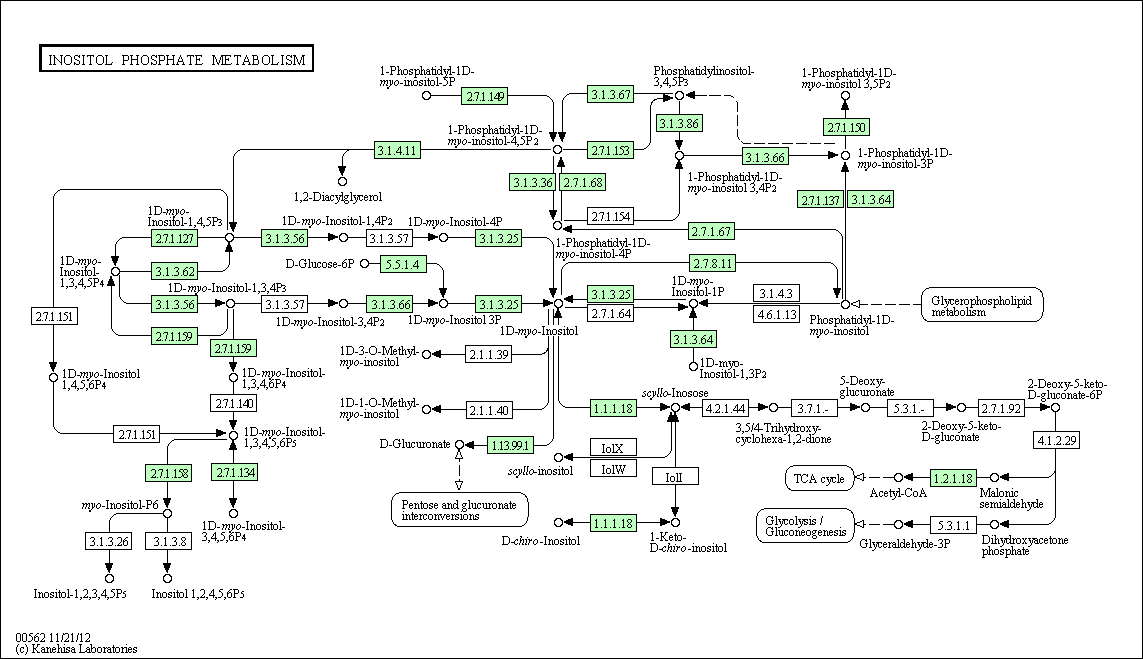

Supplement: Supplemental Information 9 [file peerj-04-1616-s009.gz › map/map00562.png]

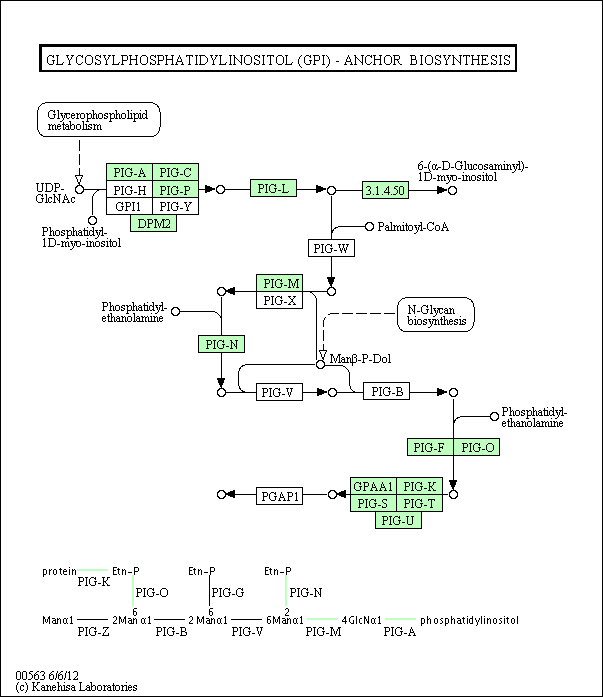

Supplement: Supplemental Information 9 [file peerj-04-1616-s009.gz › map/map00563.png]

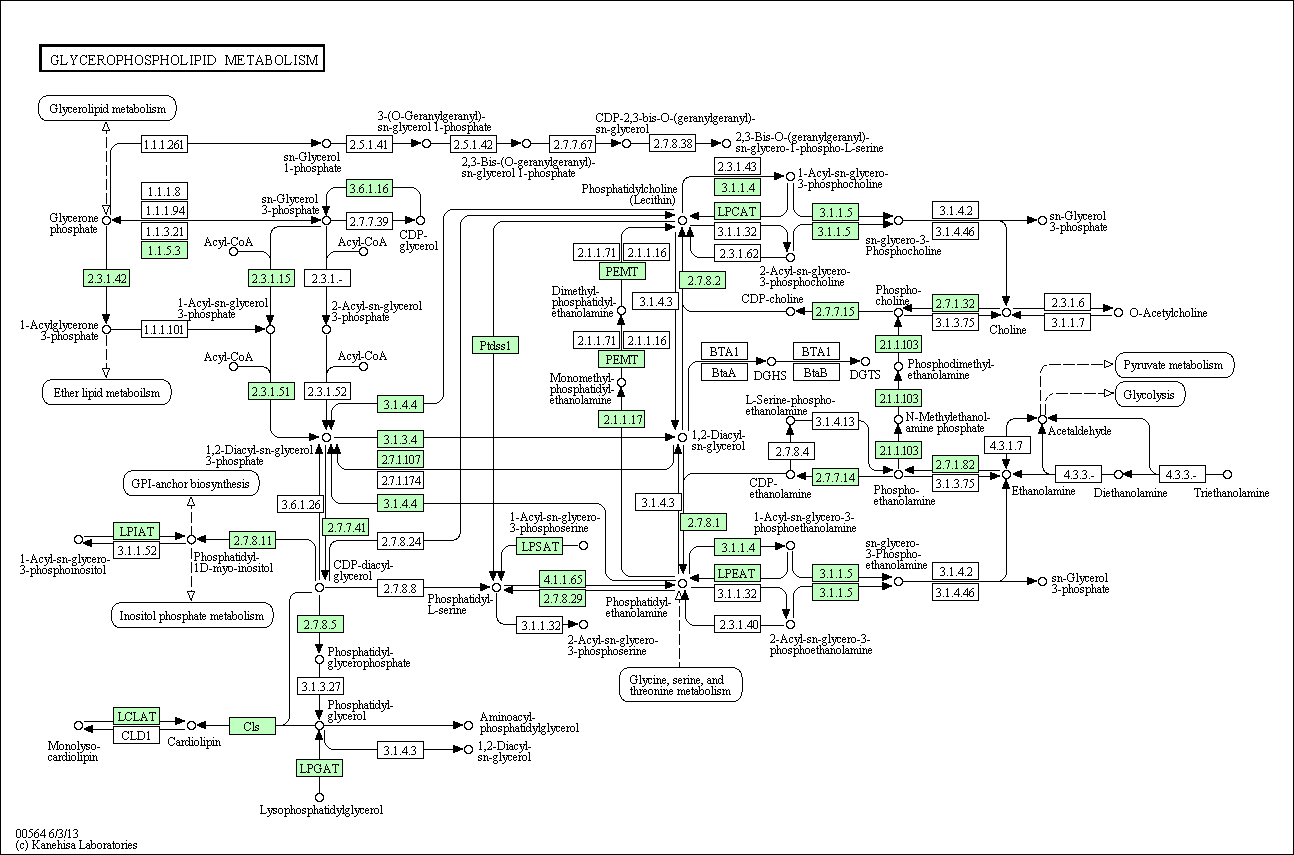

Supplement: Supplemental Information 9 [file peerj-04-1616-s009.gz › map/map00564.png]

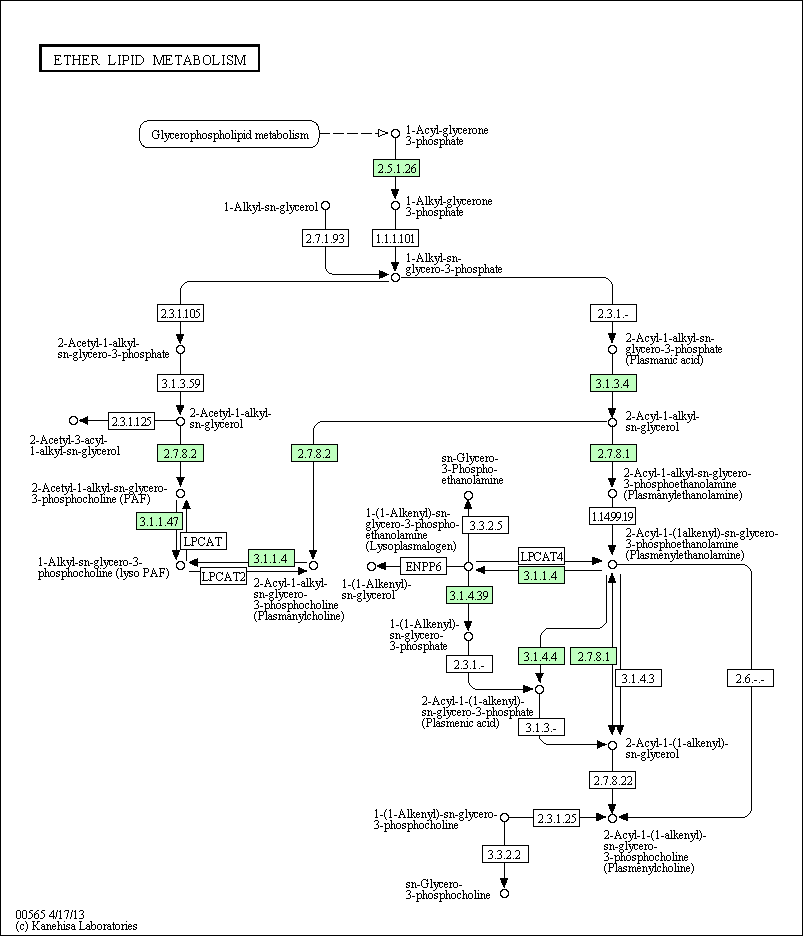

Supplement: Supplemental Information 9 [file peerj-04-1616-s009.gz › map/map00565.png]

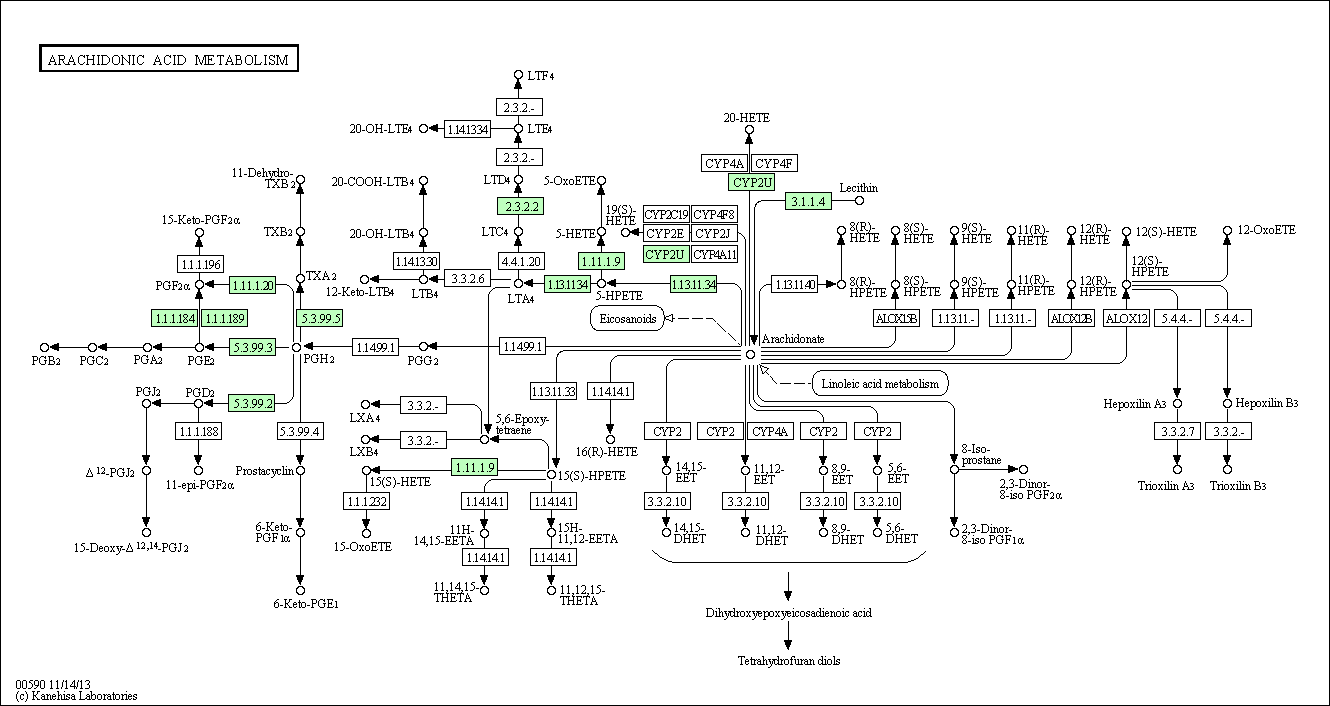

Supplement: Supplemental Information 9 [file peerj-04-1616-s009.gz › map/map00590.png]

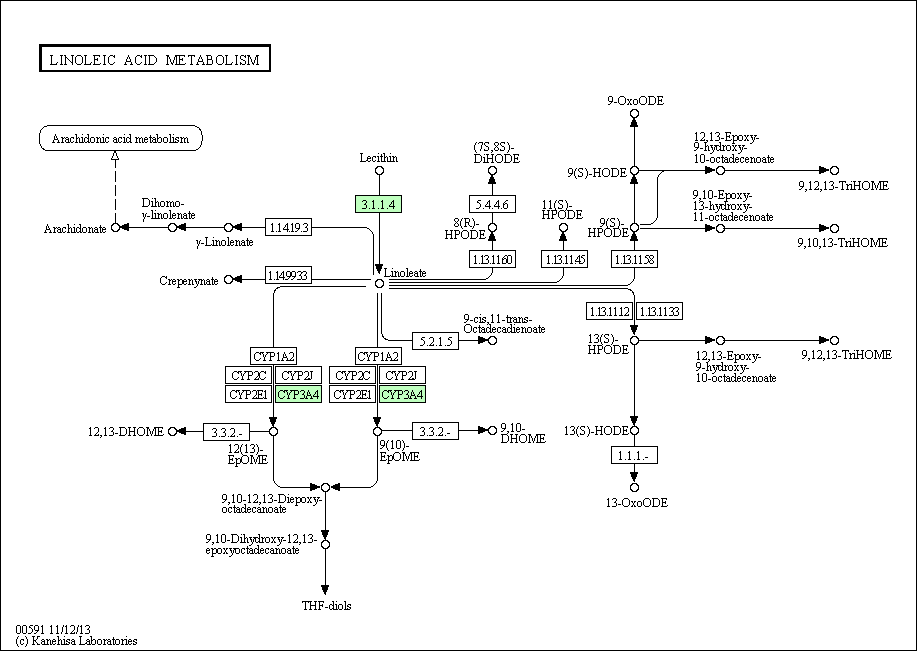

Supplement: Supplemental Information 9 [file peerj-04-1616-s009.gz › map/map00591.png]

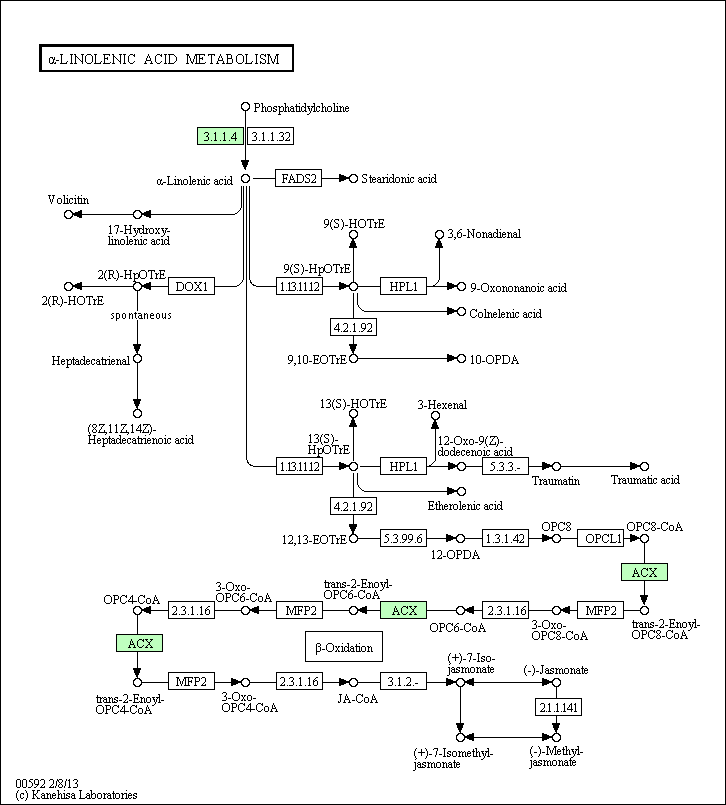

Supplement: Supplemental Information 9 [file peerj-04-1616-s009.gz › map/map00592.png]

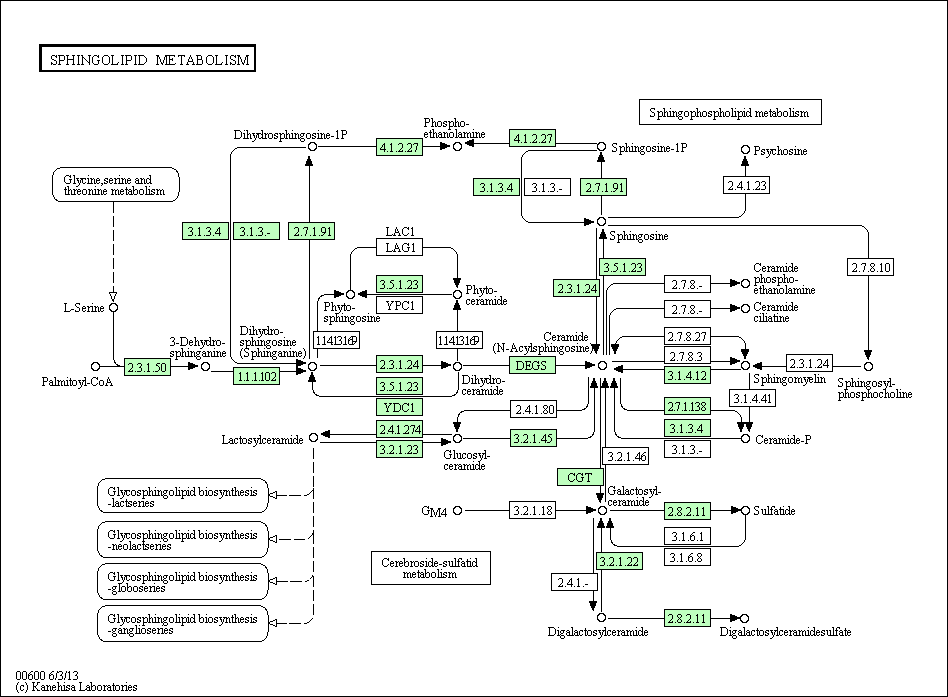

Supplement: Supplemental Information 9 [file peerj-04-1616-s009.gz › map/map00600.png]

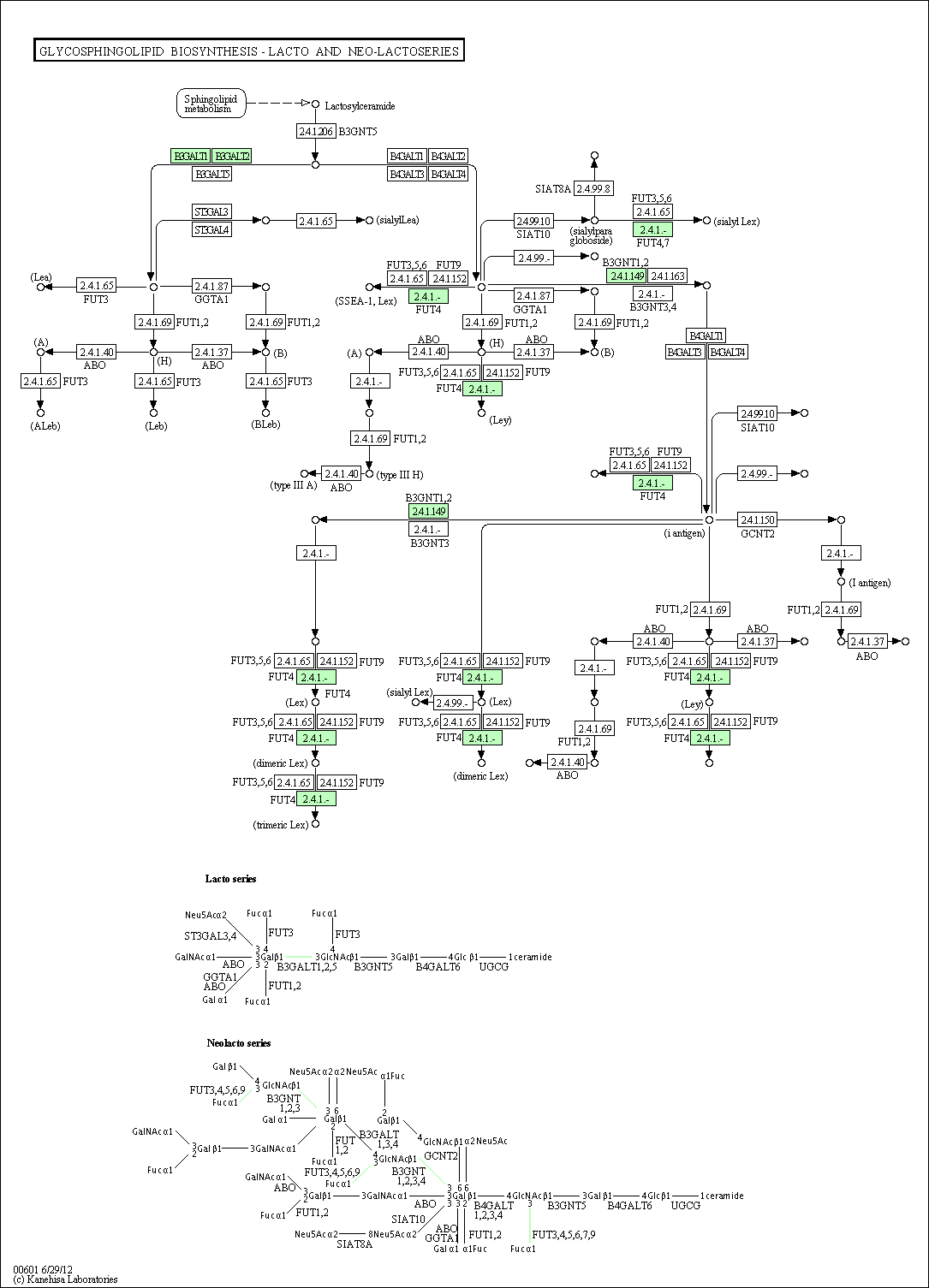

Supplement: Supplemental Information 9 [file peerj-04-1616-s009.gz › map/map00601.png]

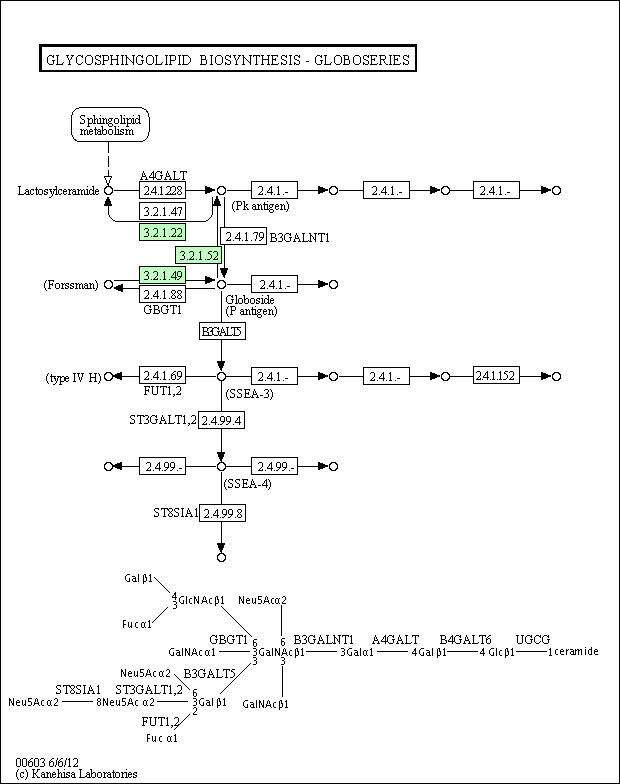

Supplement: Supplemental Information 9 [file peerj-04-1616-s009.gz › map/map00603.png]

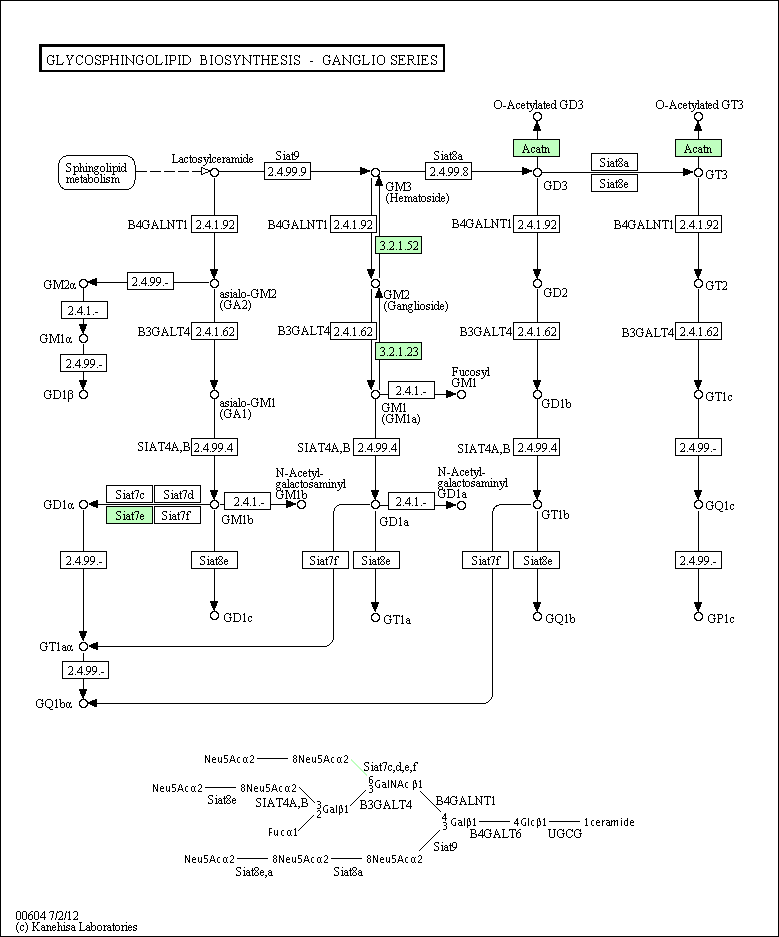

Supplement: Supplemental Information 9 [file peerj-04-1616-s009.gz › map/map00604.png]

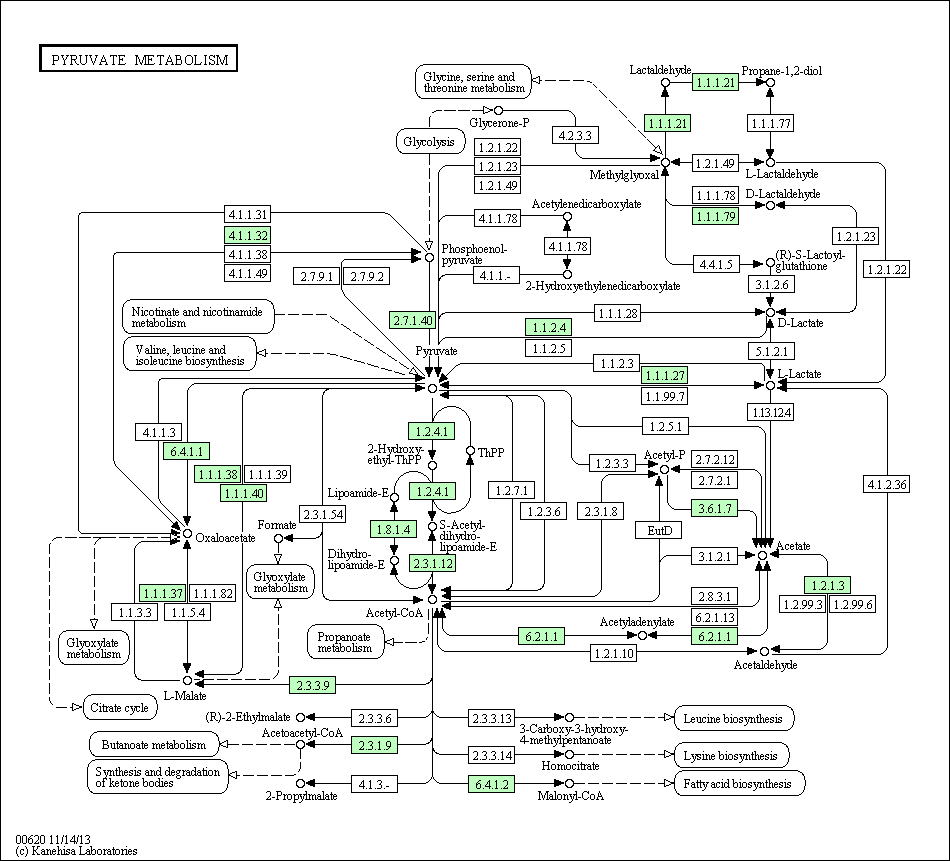

Supplement: Supplemental Information 9 [file peerj-04-1616-s009.gz › map/map00620.png]

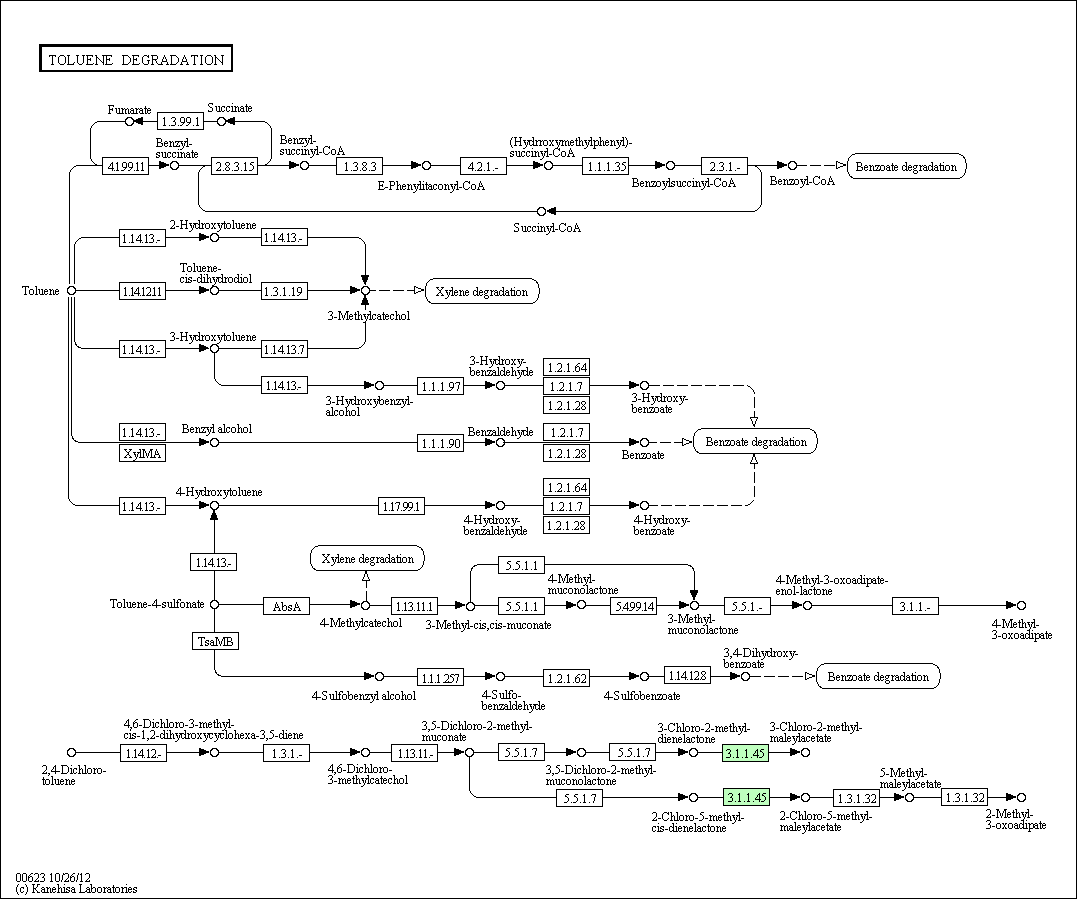

Supplement: Supplemental Information 9 [file peerj-04-1616-s009.gz › map/map00623.png]

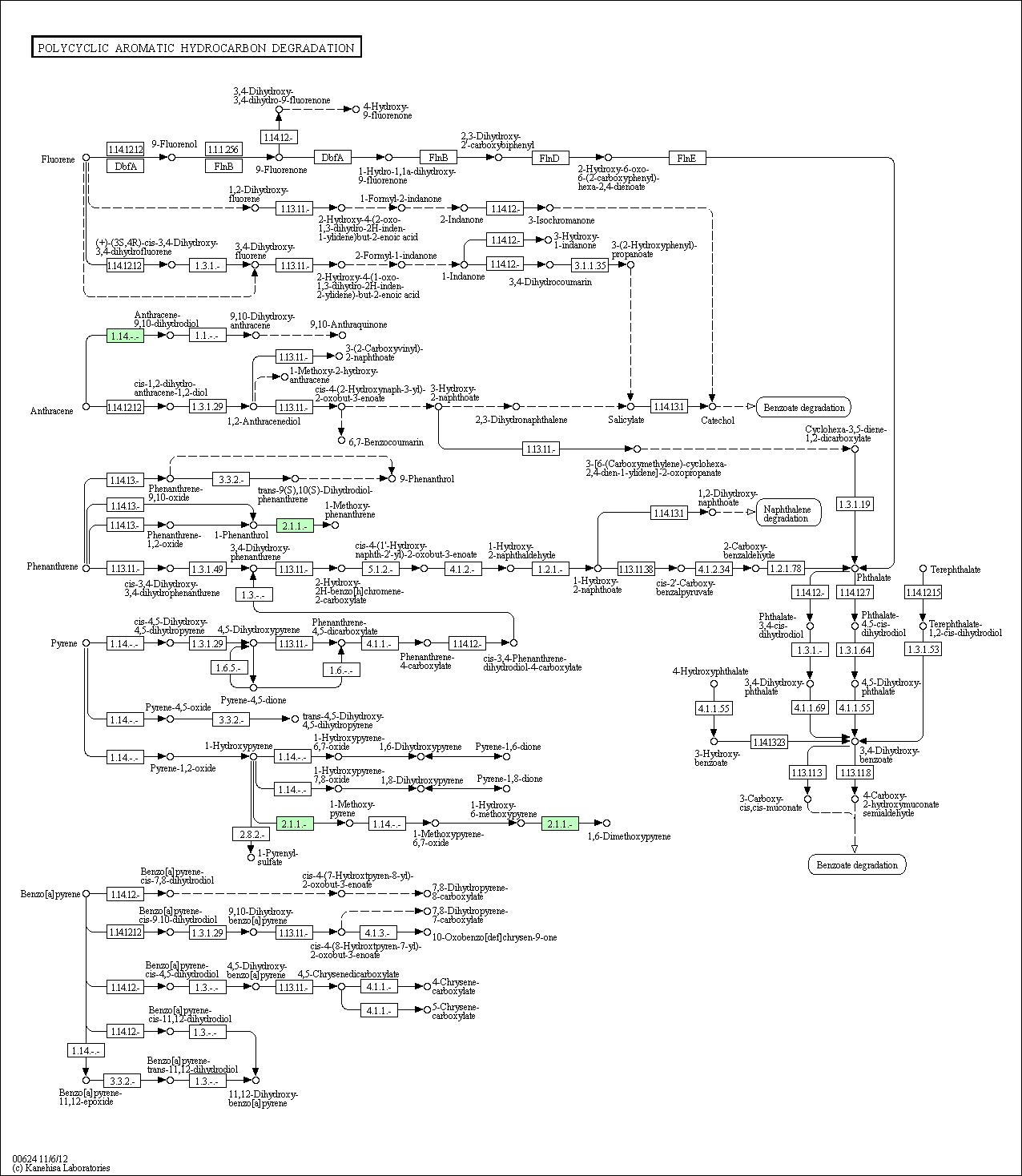

Supplement: Supplemental Information 9 [file peerj-04-1616-s009.gz › map/map00624.png]

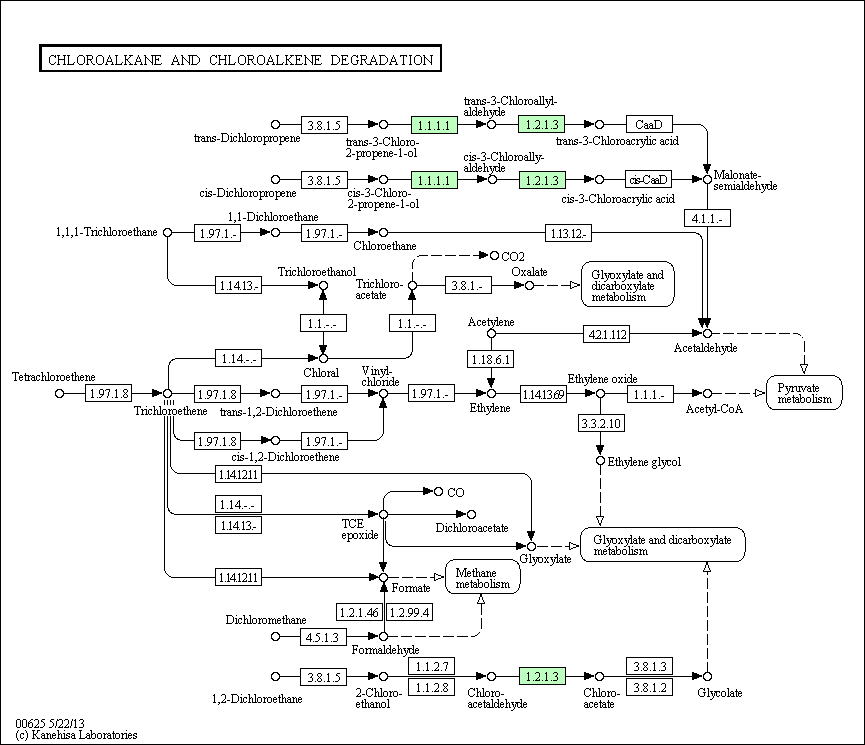

Supplement: Supplemental Information 9 [file peerj-04-1616-s009.gz › map/map00625.png]

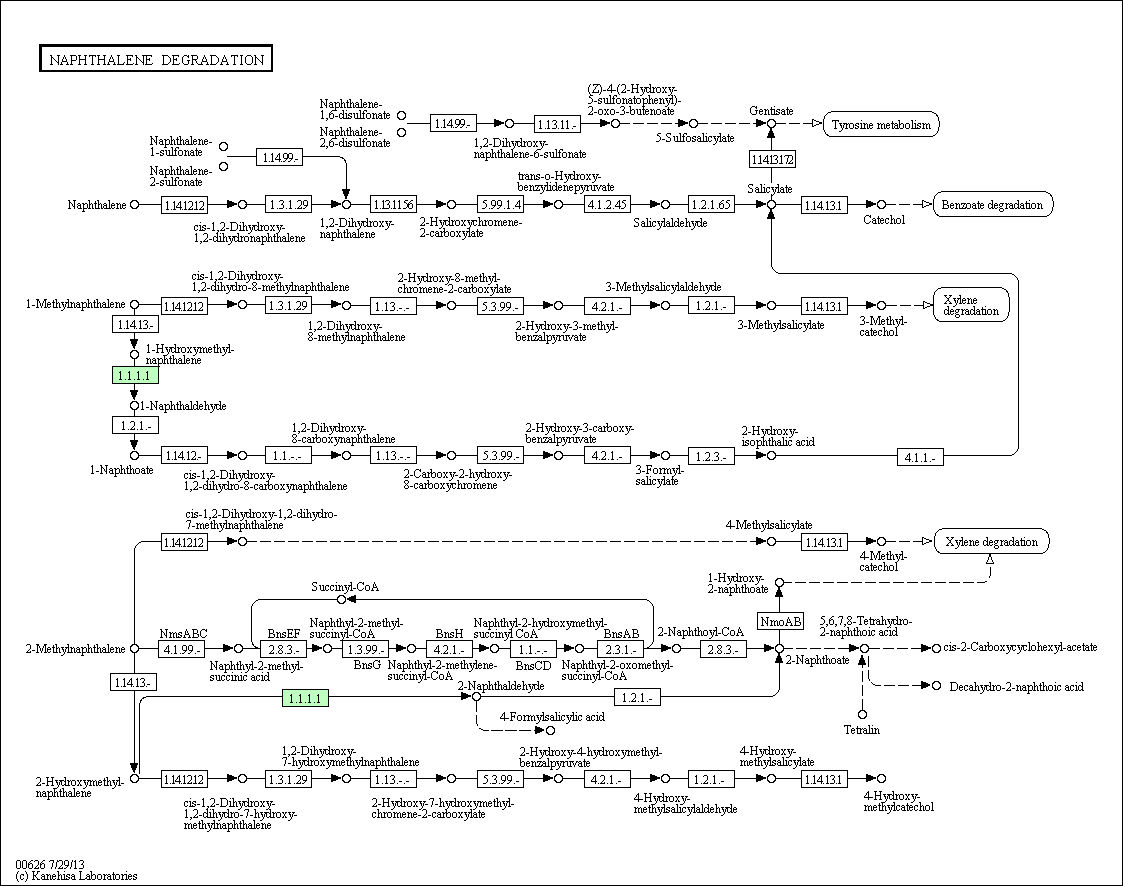

Supplement: Supplemental Information 9 [file peerj-04-1616-s009.gz › map/map00626.png]

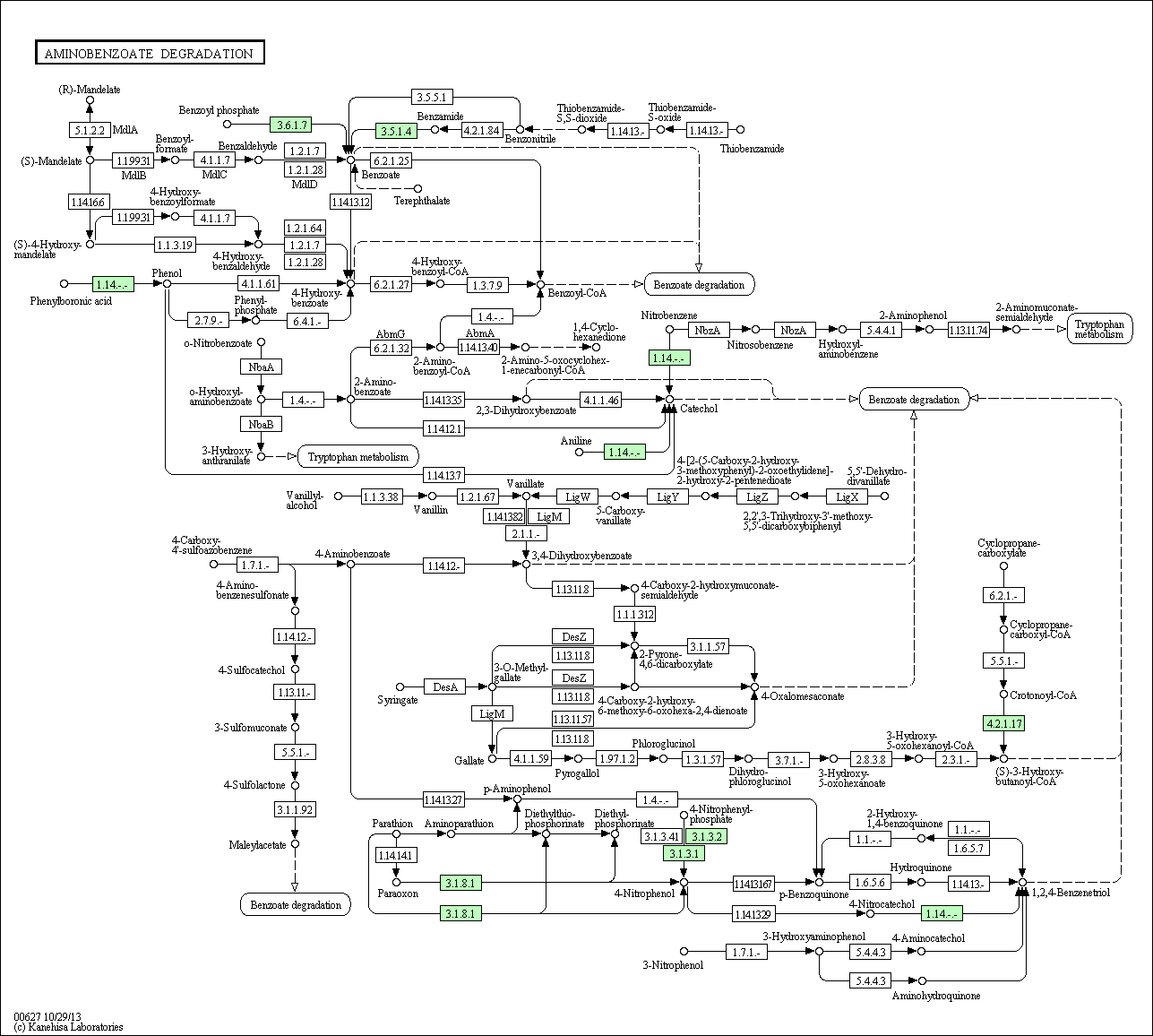

Supplement: Supplemental Information 9 [file peerj-04-1616-s009.gz › map/map00627.png]

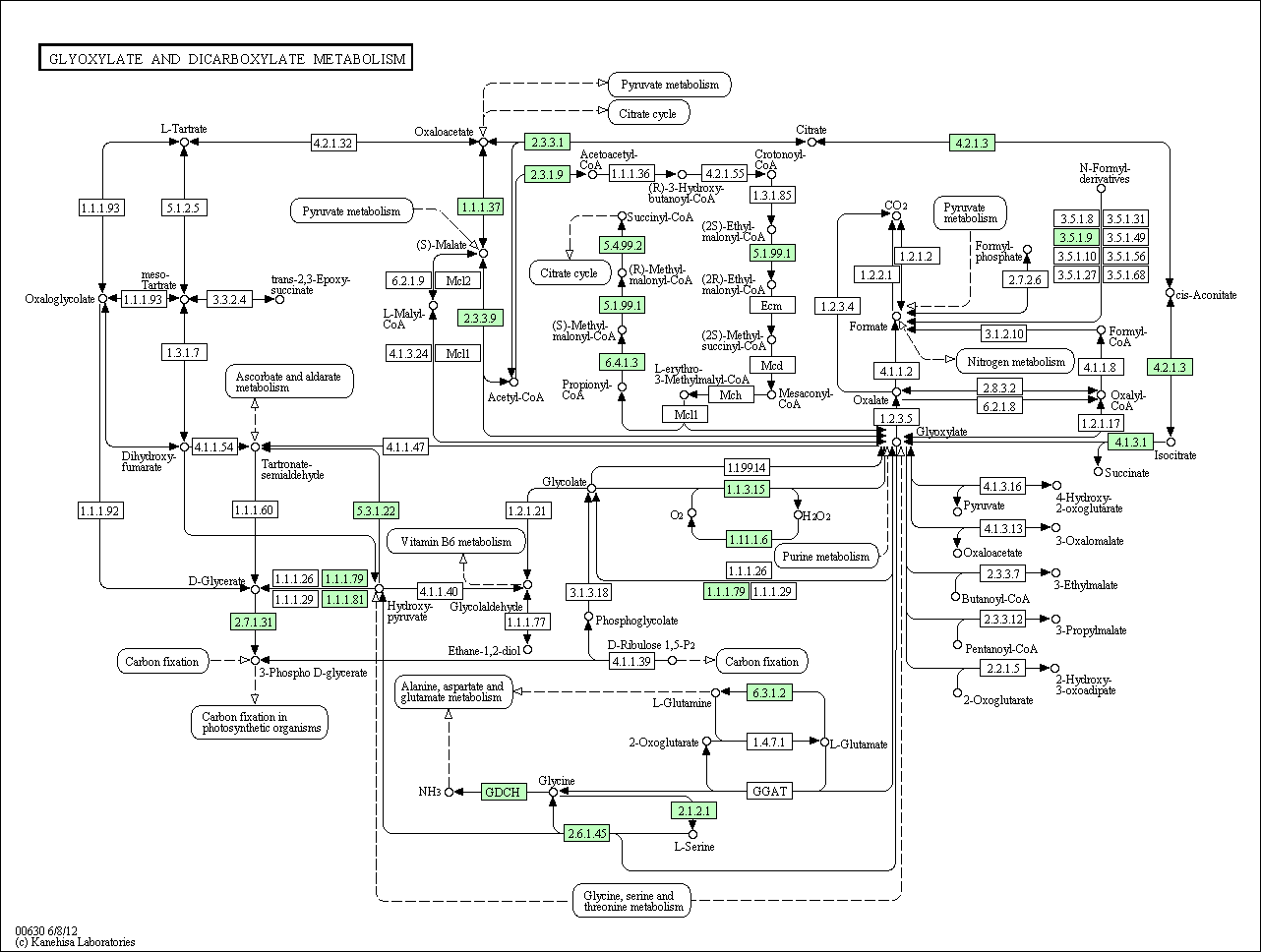

Supplement: Supplemental Information 9 [file peerj-04-1616-s009.gz › map/map00630.png]

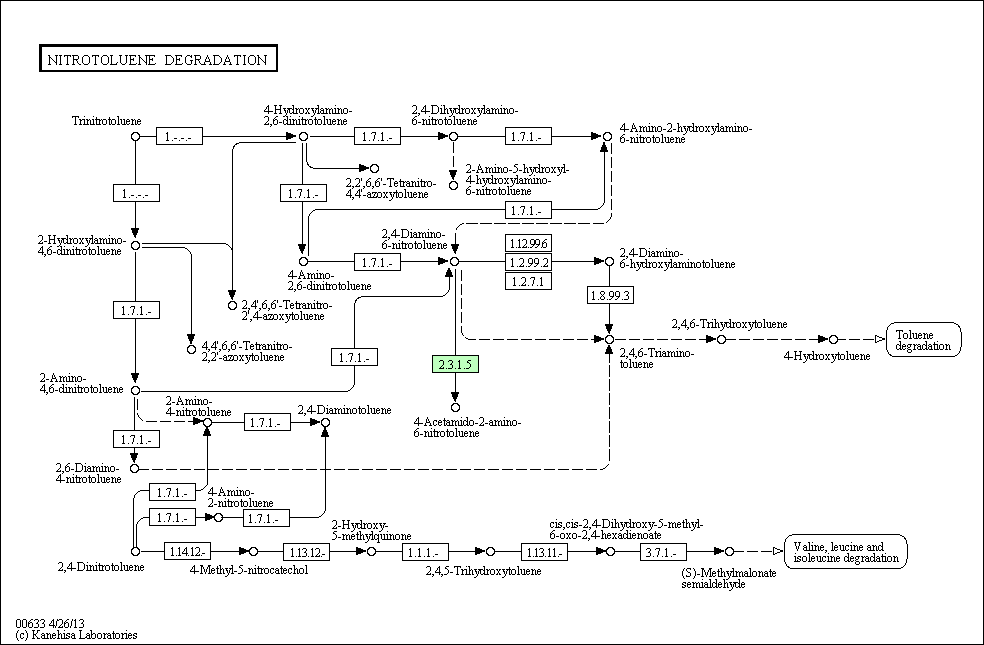

Supplement: Supplemental Information 9 [file peerj-04-1616-s009.gz › map/map00633.png]

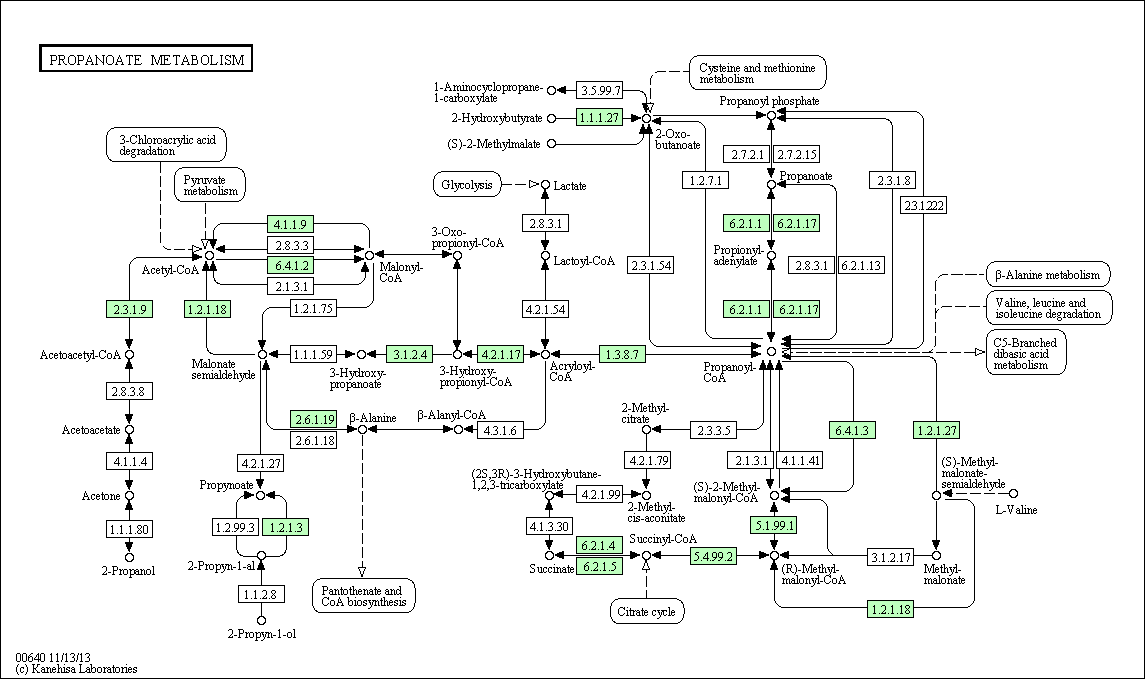

Supplement: Supplemental Information 9 [file peerj-04-1616-s009.gz › map/map00640.png]

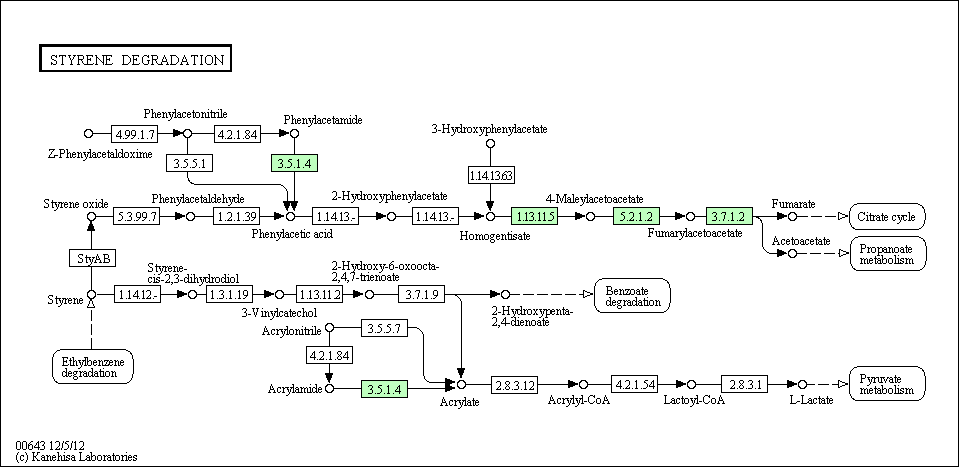

Supplement: Supplemental Information 9 [file peerj-04-1616-s009.gz › map/map00643.png]

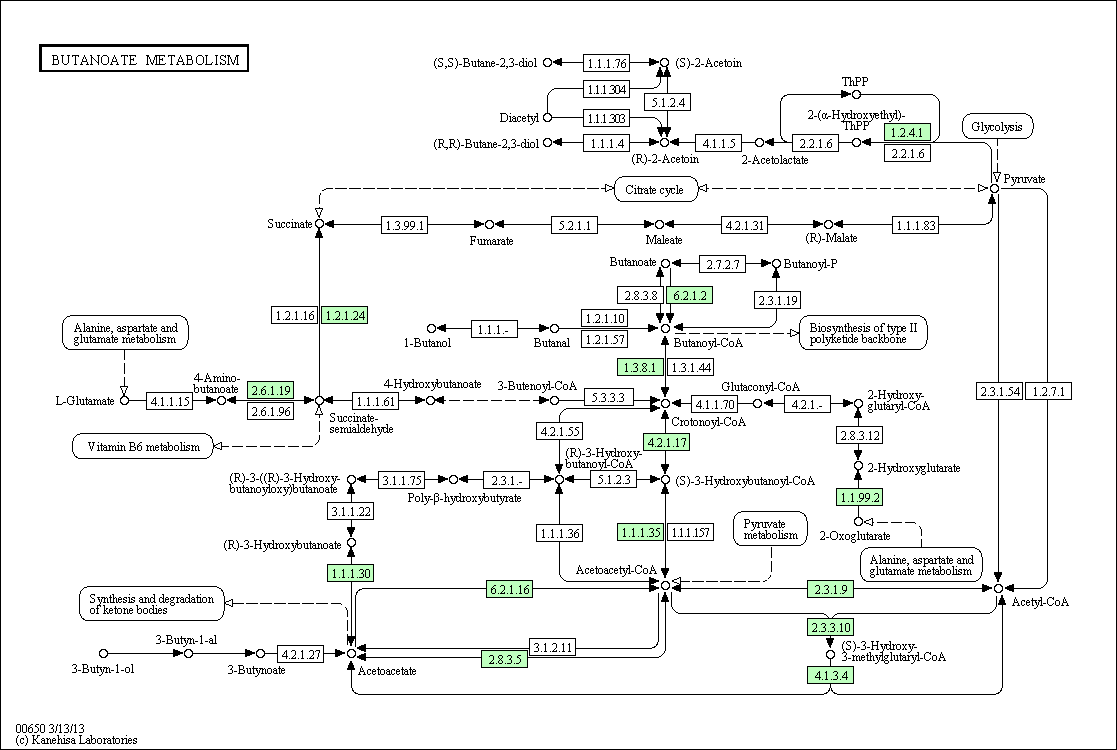

Supplement: Supplemental Information 9 [file peerj-04-1616-s009.gz › map/map00650.png]

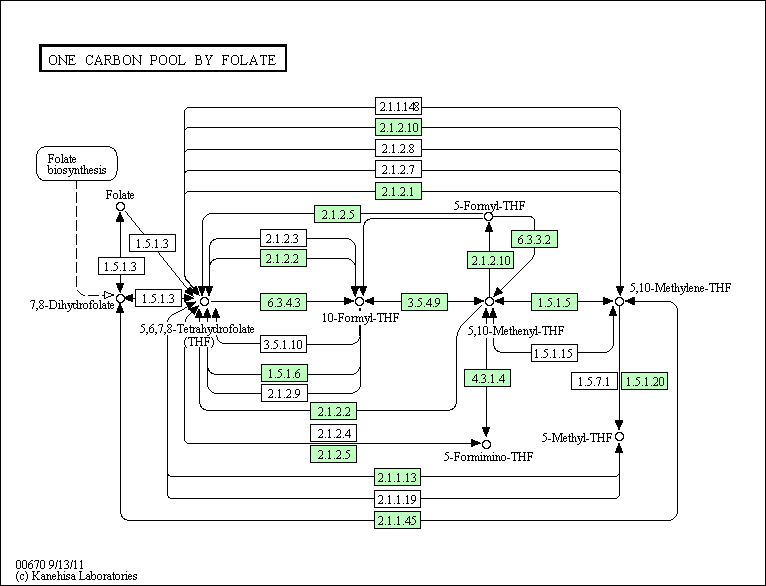

Supplement: Supplemental Information 9 [file peerj-04-1616-s009.gz › map/map00670.png]

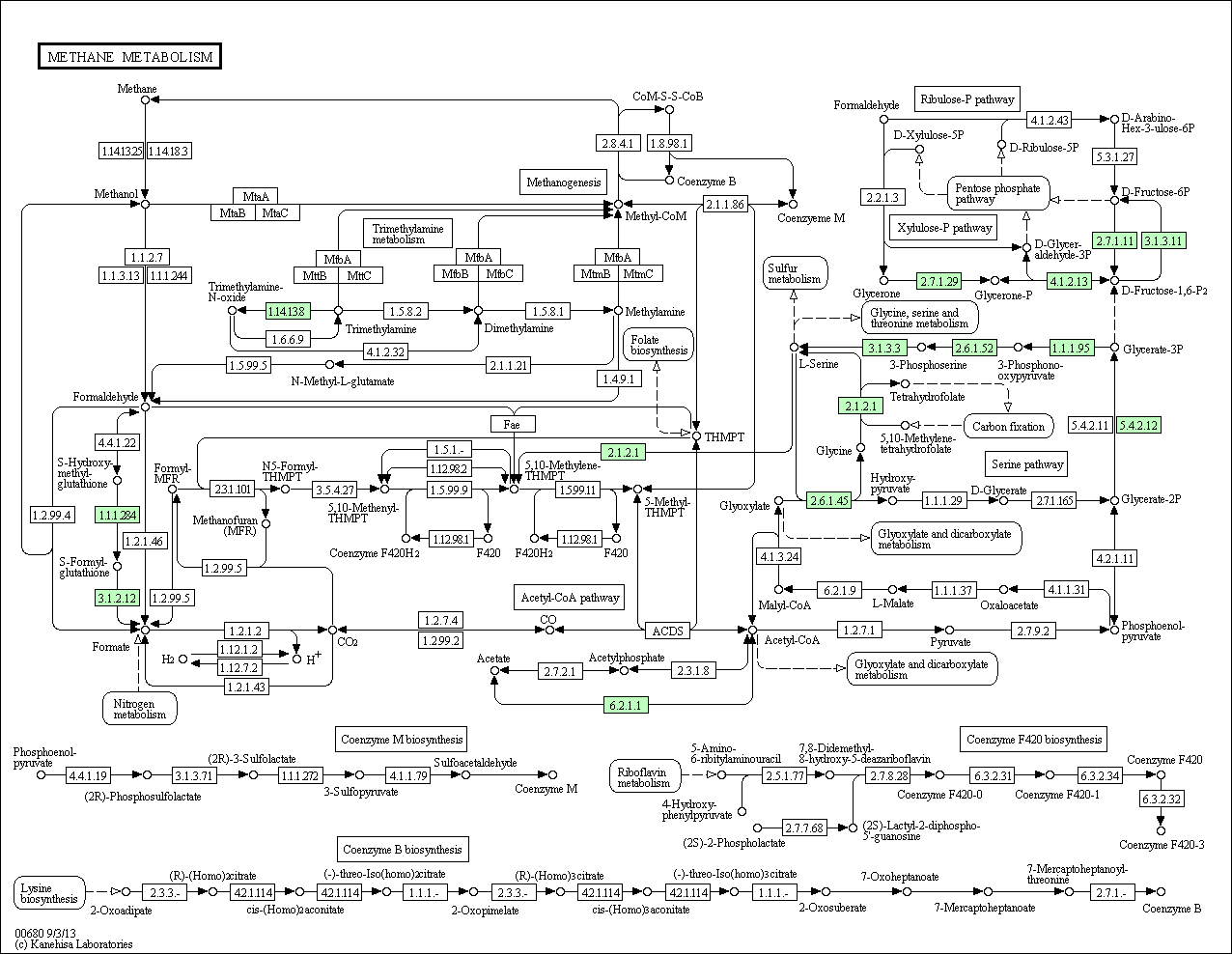

Supplement: Supplemental Information 9 [file peerj-04-1616-s009.gz › map/map00680.png]

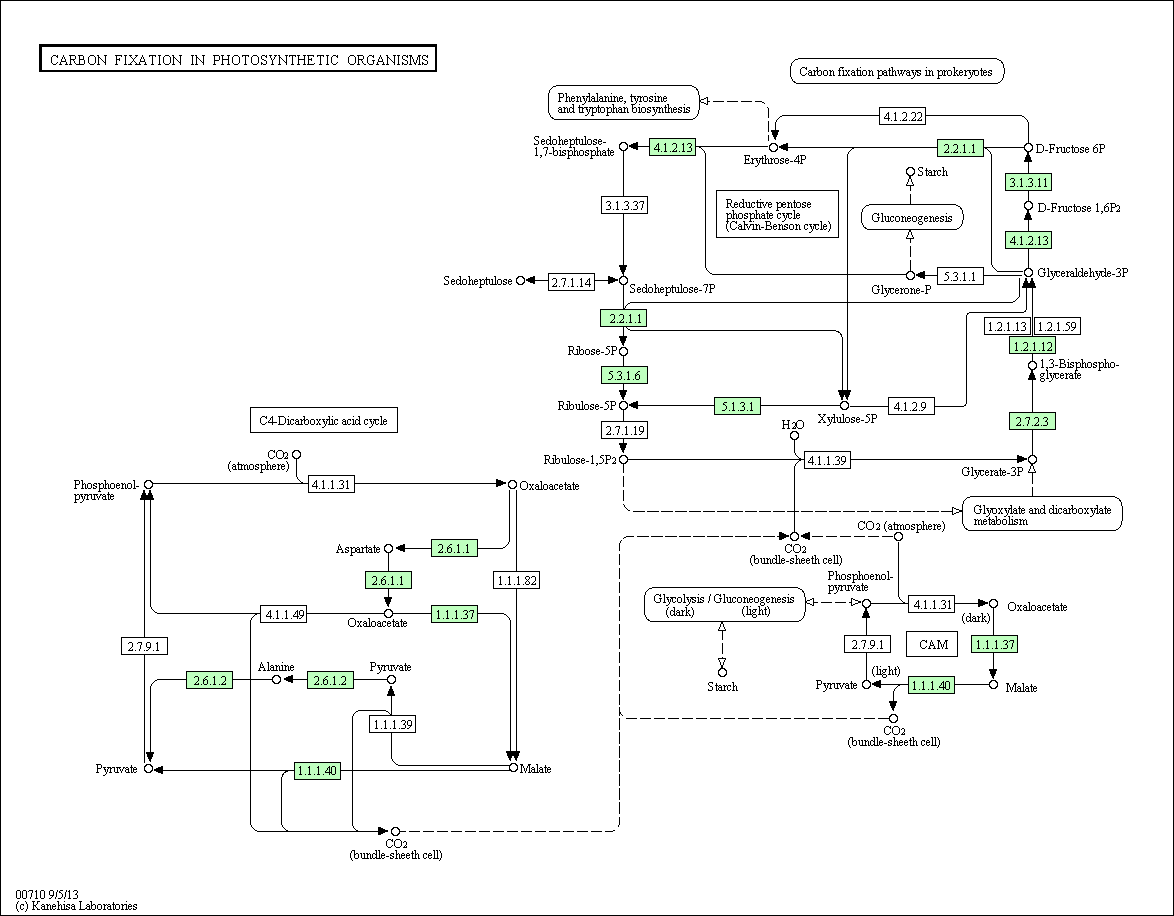

Supplement: Supplemental Information 9 [file peerj-04-1616-s009.gz › map/map00710.png]

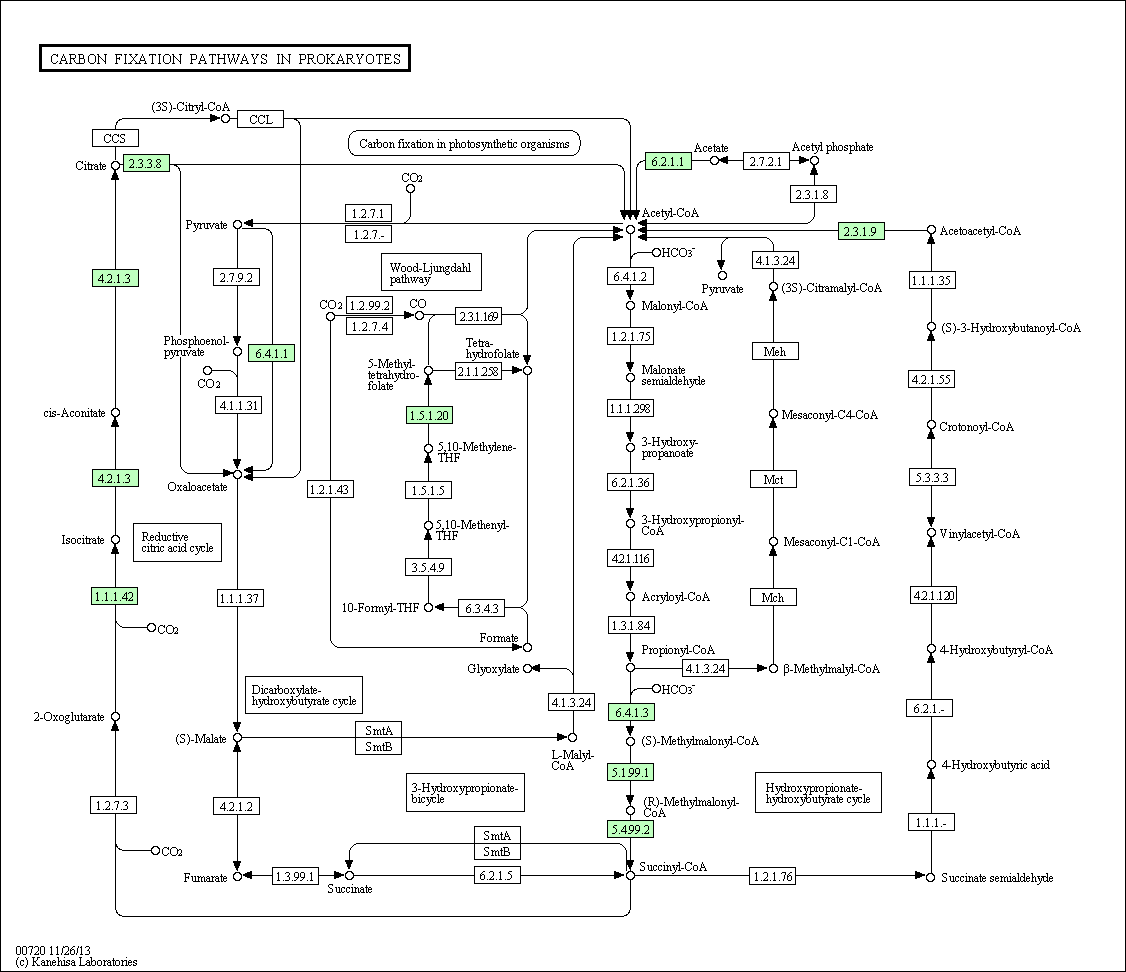

Supplement: Supplemental Information 9 [file peerj-04-1616-s009.gz › map/map00720.png]

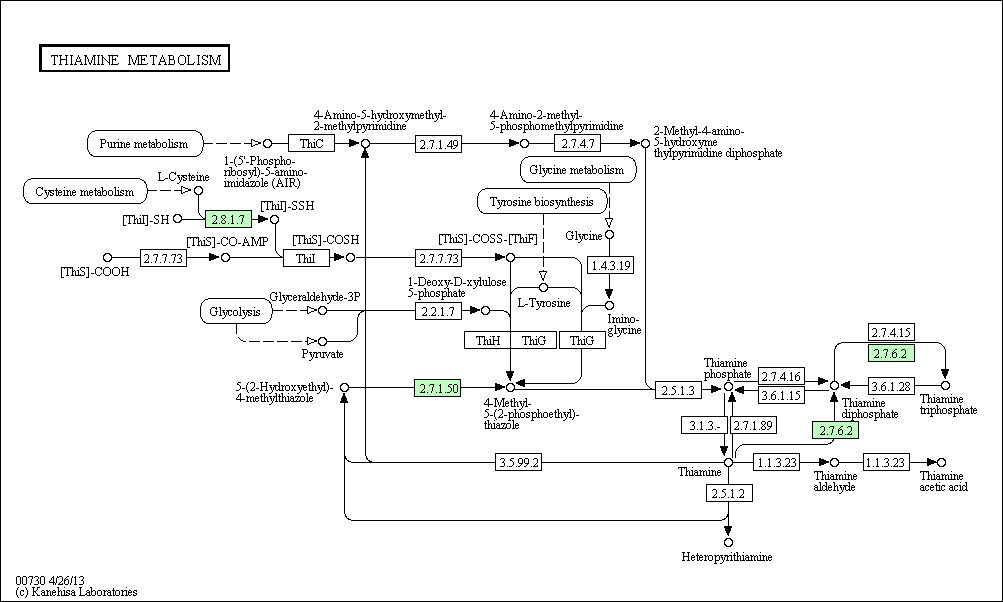

Supplement: Supplemental Information 9 [file peerj-04-1616-s009.gz › map/map00730.png]

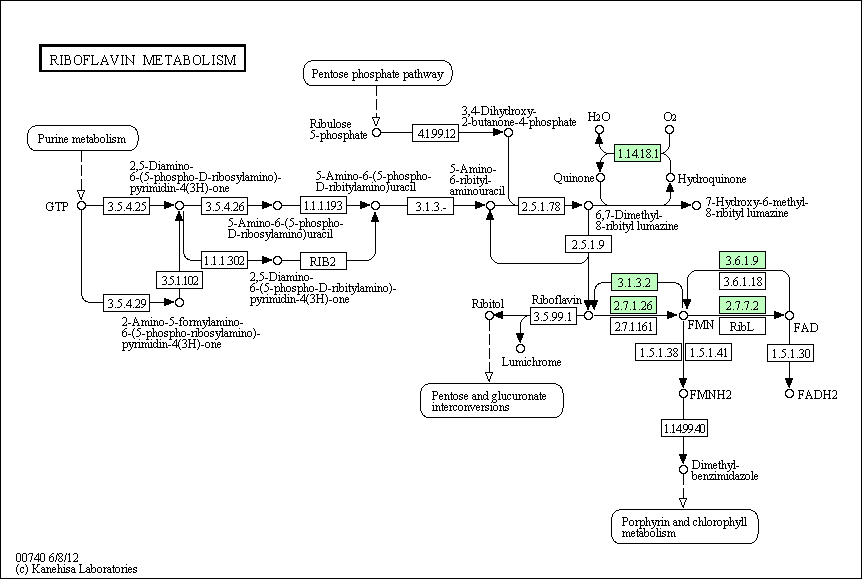

Supplement: Supplemental Information 9 [file peerj-04-1616-s009.gz › map/map00740.png]

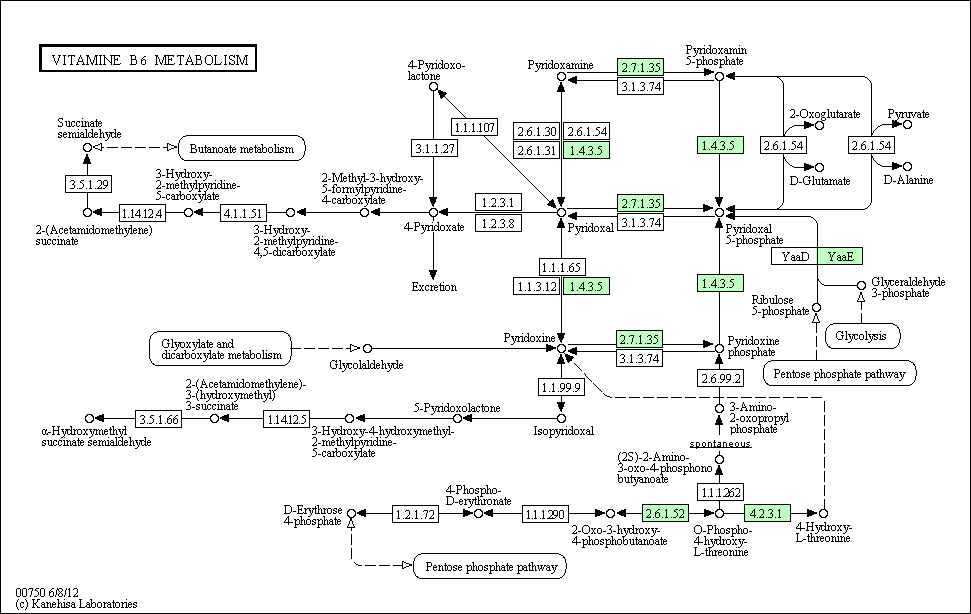

Supplement: Supplemental Information 9 [file peerj-04-1616-s009.gz › map/map00750.png]

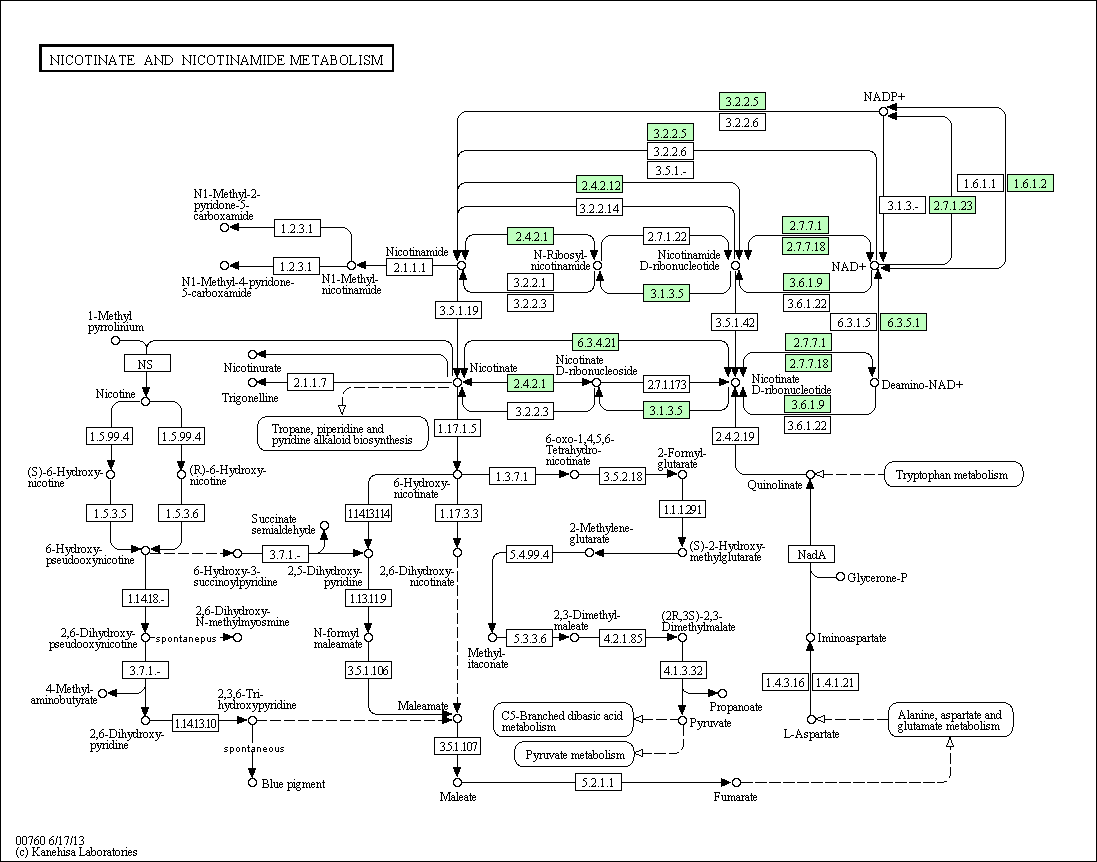

Supplement: Supplemental Information 9 [file peerj-04-1616-s009.gz › map/map00760.png]

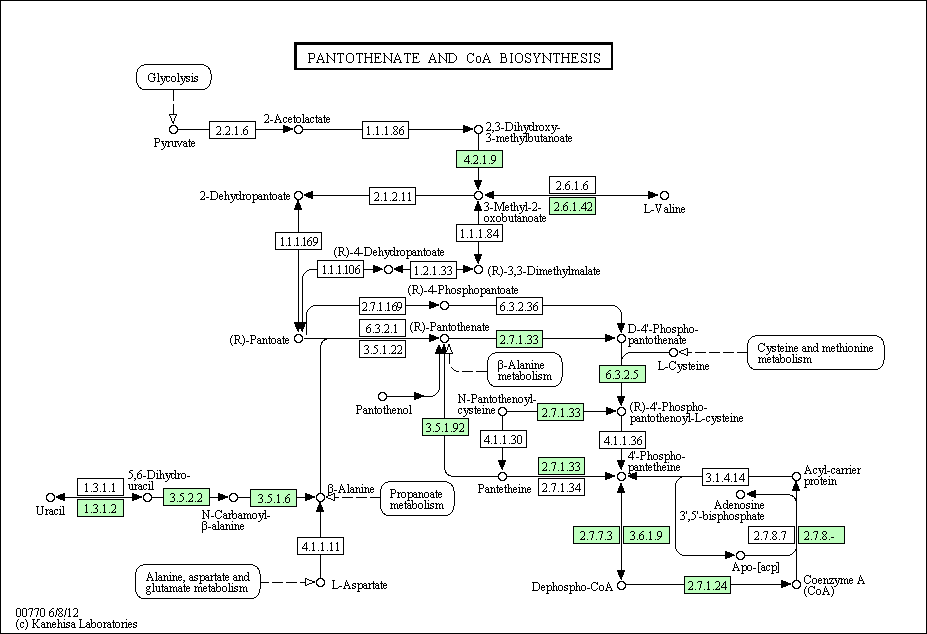

Supplement: Supplemental Information 9 [file peerj-04-1616-s009.gz › map/map00770.png]

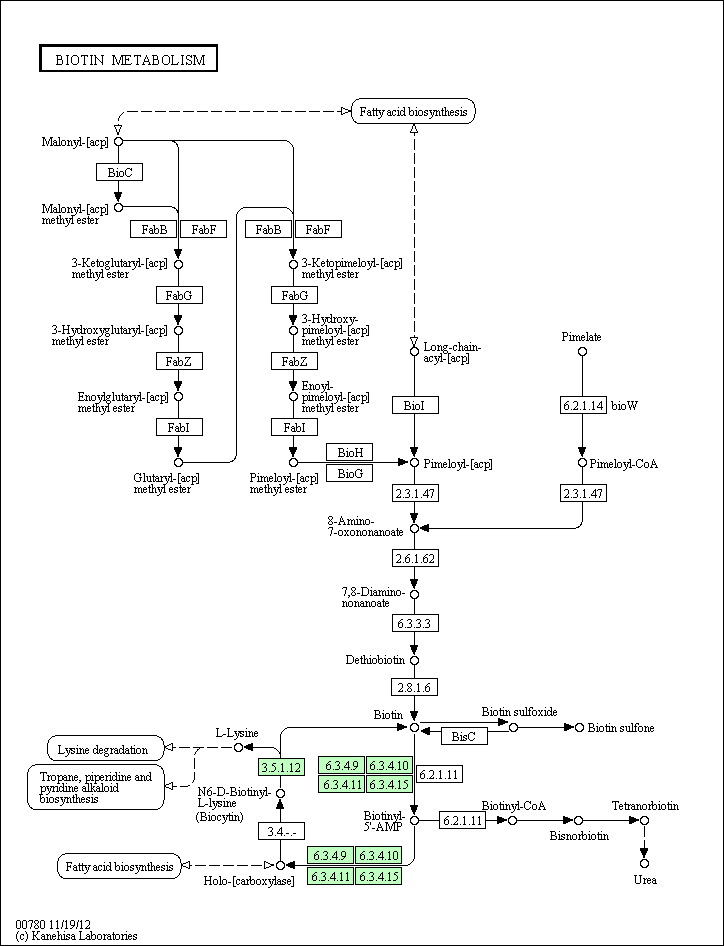

Supplement: Supplemental Information 9 [file peerj-04-1616-s009.gz › map/map00780.png]

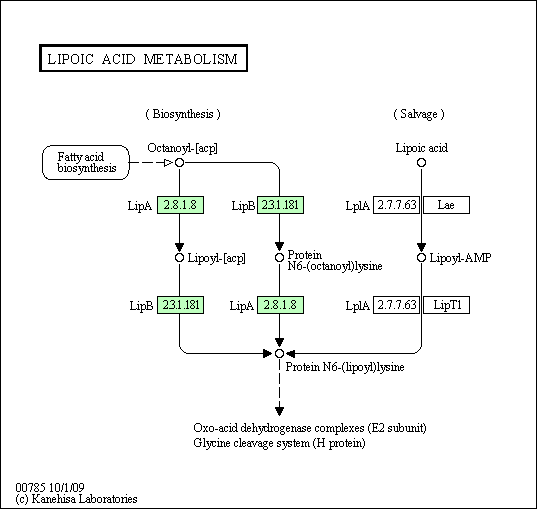

Supplement: Supplemental Information 9 [file peerj-04-1616-s009.gz › map/map00785.png]

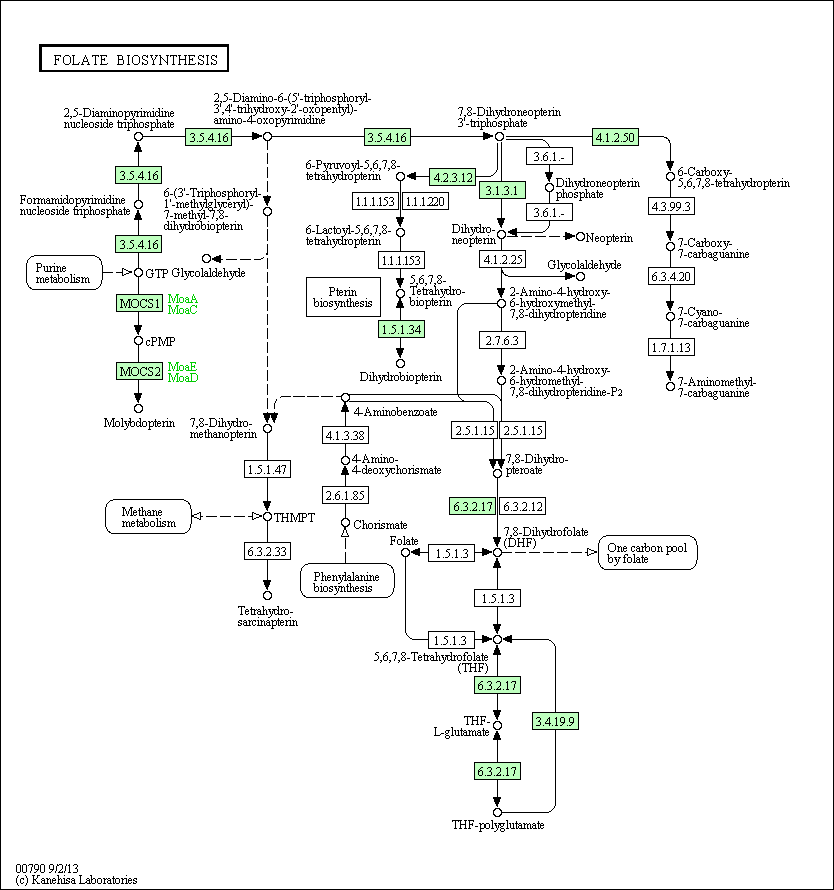

Supplement: Supplemental Information 9 [file peerj-04-1616-s009.gz › map/map00790.png]

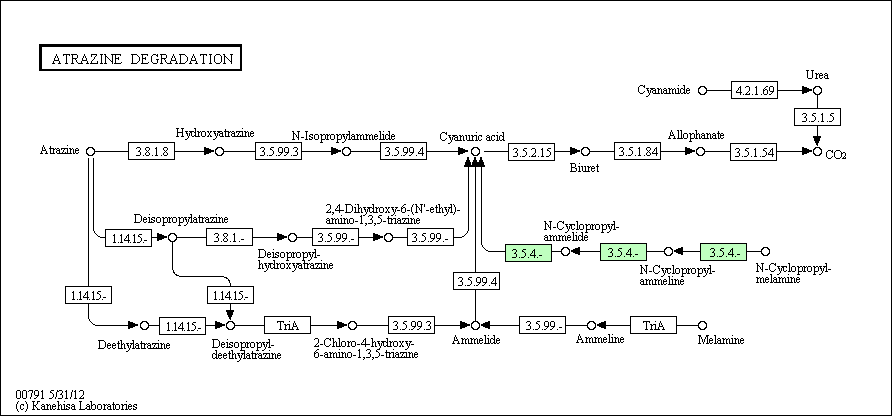

Supplement: Supplemental Information 9 [file peerj-04-1616-s009.gz › map/map00791.png]
